# Characterization of Esophageal Microbiota in Patients With Esophagitis and Esophageal Squamous Cell Carcinoma

**DOI:** 10.3389/fcimb.2021.774330

**Published:** 2021-11-11

**Authors:** Zongdan Jiang, Jun Wang, Ziyang Shen, Zhenyu Zhang, Shukui Wang

**Affiliations:** ^1^ Department of Gastroenterology, Nanjing First Hospital, Nanjing Medical University, Nanjing, China; ^2^ Department of Gastroenterology and Hepatology, Jinhu County People’s Hospital, Huaian, China; ^3^ Department of Endocrinology, Nanjing First Hospital, Nanjing Medical University, Nanjing, China; ^4^ General Clinical Research Center, Nanjing First Hospital, Nanjing Medical University, Nanjing, China

**Keywords:** esophageal microbiota, esophagitis patients, esophageal squamous cell carcinoma (ESCC), *Streptococcus anginosus*, Nanjing area

## Abstract

Microbial imbalances have been well elucidated in esophageal adenocarcinoma. However, few studies address the microbiota in esophageal squamous cell carcinoma (ESCC) and esophagitis (ES). We aimed to explore the association of esophageal microbiota with these patients. Esophageal tissues were obtained from healthy controls and ES and ESCC patients undergoing upper endoscopy. 16S rRNA gene sequencing was applied to analyze the microbiome. The α and β diversity differences were tested by Tukey test and partial least squares-discriminant analysis (PLS-DA), respectively. Linear discriminant analysis effect size (LEfSe) analysis was performed to assess taxonomic differences between groups. A total of 68 individuals were enrolled (control = 21, ES = 15, ESCC = 32). Microbial diversity was significantly different between the ESCC patients and healthy controls by Chao1 index, Shannon index, and PLS-DA. *Firmicutes*, *Proteobacteria*, *Bacteroidetes*, *Actinobacteria*, and *Fusobacteria* were the five dominant bacterial phyla among the three groups. *Megamonas*, *Collinsella*, *Roseburia*, and *Ruminococcus_2* showed a significantly continuous decreasing trend from the control group to the ESCC group at the genus level. When compared with the control group, decreased *Fusobacteria* at phylum level and *Faecalibacterium*, *Bacteroides*, *Curvibacter*, and *Blautia* at genus level were detected. ESCC samples also displayed a striking reduction of *Bacteroidetes*, *Faecalibacterium*, *Bacteroides*, and *Blautia* in comparison with the ES patients. LEfSe analysis indicated a greater abundance of *Streptococcus*, *Actinobacillus*, *Peptostreptococcus*, *Fusobacterium*, and *Prevotella* in the ESCC group. Our study suggests a potential association between esophageal microbiome dysbiosis and ESCC and provides insights into potential screening markers for esophageal cancer.

## Introduction

Esophageal cancer is a highly lethal malignancy with a rapidly increasing incidence globally. It has been ranked the fourth leading cause of cancer-related death, which brings a substantial public health burden (Feng et al., 2019). The main histological types of esophageal carcinoma are esophageal adenocarcinoma (EAC) and esophageal squamous cell carcinoma (ESCC), with the latter predominating in the Chinese population (Kelly, 2019). Although the treatment for ESCC has been notably improved, the prognosis of ESCC is still not satisfactory, with a 5-year overall survival rate of 30.3% (Baba et al., 2014; Torre et al., 2015). Thus, we still need to further advance our insights into ESCC and attempt to offer new therapeutic alternatives.

Esophagitis (ES), smoking, drinking, and heredity are the known risk factors related to ESCC (Kavin et al., 1996; Reichenbach et al., 2019). Recently, microorganisms have been considered to exert essential functions in the occurrence and progression of gastrointestinal diseases. There are at least 38 trillion microorganisms colonizing the human gastrointestinal tract, which associate with the immunological homeostasis. Increasing evidence suggests that an imbalance of certain species is involved in tumor onset and development *via* producing carcinogenic toxins, dampening the immunity, and damaging DNA structure. Previous studies reported that there was a significant decrease in bacterial counts and alterations in microbial communities in the gastroesophageal reflux disease (GERD) and Barrett’s esophagus groups (Blackett et al., 2013; Elliott et al., 2017) compared with the healthy group. Alterations of microbial diversity, including a lower level of *Veillonella* and *Streptococcus* and a higher level of *Lactobacillus*, *Enterobacteriaceae*, and *Akkermansia*, are associated with EAC (Lopetuso et al., 2020). However, the relationship between human esophageal microbiota and ESCC has not garnered sufficient scientific attention.

Hence, in the present study, we aimed to assess and compare the diversity and composition of the microbiota between ESCC, ES, and healthy tissues and complemented the clinical data in this area. This might further illustrate the role of microbiota in the pathogenesis of ESCC and bring light to the treatment of ESCC.

## Materials and Methods

### Study Participants

Samples were obtained from patients undergoing routine upper endoscopy for the screening of upper gastrointestinal cancer or clinical indications. Finally, we recruited 15 esophagitis patients (ES group) and 21 healthy volunteers (Control group) in Nanjing First Hospital from 2018 to 2019. A total of 32 ESCC patients (ESCC group) were enrolled from Jinhu People’s Hospital. Sampling from esophagectomy was done with a sterile scalpel blade (cutting down to submucosa) within 1 h of surgical resection. All samples were flash frozen in liquid nitrogen and stored at –80°C. The inclusion criteria were age older than 18 years and no contraindications for endoscopic examination. The ES and ESCC groups were composed of patients diagnosed as ES and ESCC, respectively, which were confirmed by both endoscopy and pathology. The control group was defined as individuals with normal esophagus confirmed by endoscopy and pathology and no digestive symptoms. To minimize the potential influence on the microbiota, all patients enrolled should not receive antibiotics, H_2_ receptor antagonists, proton pump inhibitors, and probiotics 1 month before sample collection. Patients who had received radiotherapy, chemotherapy, and/or prior surgery were excluded. The study protocol was approved by the institutional review board of Nanjing Medical University, and all experiments were performed in accordance with approved guidelines and regulations.

### DNA Extraction

Total genome DNA from samples was extracted using the QIAamp DNA Mini Kit (QIAGEN, Valencia, CA, USA) combined with the bead-beating method. The DNA concentrations of each sample were adjusted to 50 ng/μl for subsequent 16S rDNA gene analysis. The bacterial DNA samples were stored at –80°C for sequencing.

### PCR Amplification

16S rDNA genes of V3-V4 region were amplified using universal primers, namely, 338F (5′-ACTCCTACGGGAGGCAGCAG-3′) and 806R (5′ -GGACTACHVGGGTWTCTAAT-3′). All PCR reactions (including denaturation, annealing, and elongation) were carried out with Phusion^®^ High-Fidelity PCR Master Mix (New England Biolabs). After electrophoresis of PCR products, samples with bright main strip between 400 and 450 bp were chosen for next mixing and purification with Qiagen Gel Extraction Kit (Qiagen, Germany).

### Sequencing Processing and Analysis

The purified amplifications were paired-end sequenced (PE300) on an Illumina MiSeq platform (Illumina, San Diego, CA, USA). Barcodes and sequencing primers were trimmed before assembly. All raw reads were stored in NCBI Sequence Read Archive (SRA) database, and the accession number is PRJNA759579.

### Statistical Analysis

The raw data were filtered with QIIME (V1.8.0), discarding the reads that were dereplicated or shorter than 150 bp. Filtered reads were clustered into operational taxonomic units (OTUs) assuming 97% similarity. Compared with the SILVA database (version 128), the species classification information of each OTU was obtained. For continuous variables, independent t-test, White’s nonparametric t-test, and Mann–Whitney U test were applied. For categorical variables between groups, Pearson chi-square or Fisher’s exact test was used, depending on assumption validity. QIIME software was used to evaluate the α diversity by calculating the Shannon index and Simpson index. To compare the differences of diversity among groups, β diversity was tested by partial least squares-discriminant analysis (PLS-DA). Linear discriminant analysis (LDA) effect size (LEfSe) was performed to find key microbes associated with different groups with the LDA threshold of 3. We used Phylogenetic Investigation of Communities by Reconstruction of Unobserved States (PICRUSt) analysis to predict Kyoto Encyclopedia of Genes and Genomes (KEGG) biochemical pathways. Statistical analysis was performed using the SPSS V19.0 (SPSS Inc., Chicago, IL, USA) and STAMP V2.1.3. GraphPad Prism V6.0 (San Diego, CA, USA) was used for preparation of graphs. A *p* value <0.05 was considered statistically significant.

## Results

### Baseline Characteristics of Participants

A total of 21 healthy volunteers, 15 ES patients, and 32 ESCC patients were enrolled in this study. Demographic characteristics of all included individuals were shown in **Table 1**. Older age was observed in ESCC group compared with the healthy controls. There was no significant difference in sex, alcohol intake, smoking, diabetic background, and family history of cancer among the groups.

**Table 1:** Clinical characteristics of enrolled patients and healthy controls.

Characteristics	Control (n = 21)	ES (n = 15)	ESCC (n = 32)	*p* value
Age	47.85 ± 12.01	55.60 ± 11.53	55.97 ± 11.62	0.040*
BMI (kg/m^2^)	24.06 ± 3.78	24.57 ± 2.92	25.89 ± 4.06	0.200
Sex (male)	13 (61.9%)	9 9 (60.0%)	20 (62.5%)	0.986
Smoker (Yes)	11 (52.4%)	10 (66.7%)	18 (56.3%)	0.684
Alcohol consumption (Yes)	7 (33.3%)	6 (60.0%)	14 (43.8%)	0.750
Diabetes (Yes)	4 (19.0%)	7 (46.7%)	9 (28.1%)	0.214
Family history of cancer (Yes)	3 (14.3%)	2 (13.3%)	3 (9.4%)	0.796

*p < 0.05.

### Microbial Richness and Diversity Among the Three Groups

**Figure 1:**
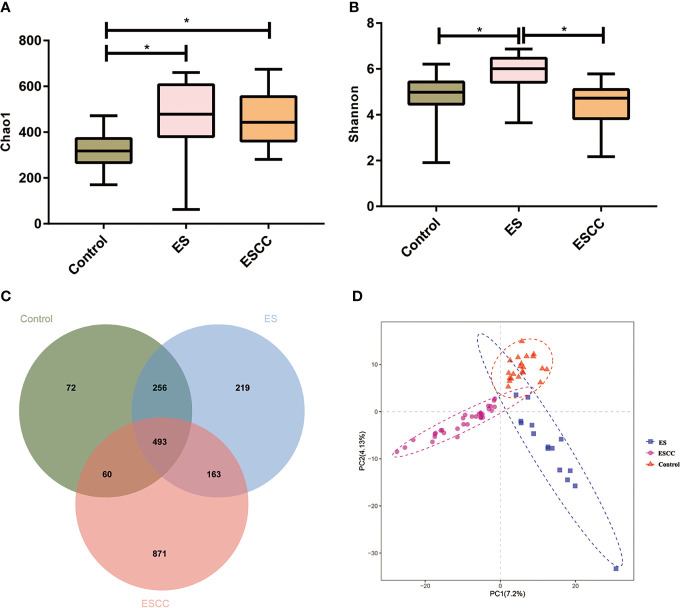
The microbial α diversity and β diversity analysis in different groups. **(A)** Chao 1 index was higher in the ESCC and ES group than in the control group. **(B)** The ES group had a significantly higher Shannon index, in comparison with the ESCC group and control group. Data were reported as minimum (min) to the maximum (max) with the line at median. **(C)** A Venn diagram displayed the overlaps among the groups. **(D)** PLS-DA revealed different microbial community structures in the three groups. *p < 0.05.

After sequencing and quality filtering, more than 3.2 million tags and a total of 2,134 OTUs were obtained with the dominant length of tags located among 400–440 bp (**Figure 1A**). To test the sequencing depth, we created the rarefaction curves and showed a reasonable amount of sampling (**Supplementary Figure S1**).

Each sample was reflected as a curve in the figure. The curve tends to be flat when the depth of sequencing increases, supporting the adequate volume of sequencing data.

The microbial α diversity and β diversity were applied to analyze the microbiota biodiversity and composition among the groups. We used Chao1 index and Shannon index to describe the community richness and diversity. A higher richness of microbiota was observed in the ESCC and ES groups than that in the control group according to the Chao1 index (ESCC vs. control, *p* = 0.0002, ES vs. control, *p* = 0.0012; **Figure 1A**). Compared with the control group, the Shannon index of the ESCC group showed a decreasing trend (*p* = 0.4171; **Figure 1B**), whereas the ES group owned a significantly higher Shannon index in comparison with the ESCC group (*p* < 0.0001) and the control group (*p* = 0.0022). Moreover, the Venn diagram indicated that 493 of the total 2,134 OTUs were shared among the three groups, with 72, 219, and 871 OTUs unique for the control, ES, and ESCC groups, respectively (**Figure 1C**). About β diversity, PLS-DA at the OTU level revealed a statistically significant clustering (**Figure 1D**), suggesting different microbial community structures.

### The Changes of Esophageal Microbiota Composition Among the Three Groups

**Figure 2:**
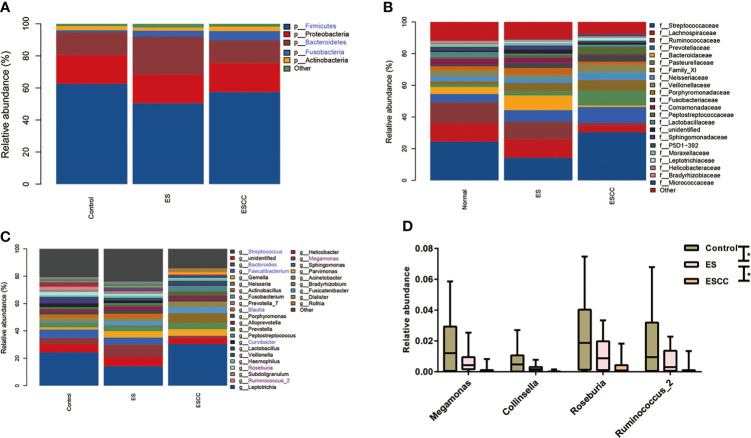
Comparison of relative abundance among each group Barplots of the relative abundance of the main bacterial taxa at **(A)** phylum, **(B)** family and **(C)** genus level for the control, ES and ESCC group. Significant taxa were highlighted in the blue font. **(D)** Mean relative abundance of continuous changing genera among the groups. Significant taxa were highlighted in the purple font. Data were reported as min to the max with the line at median. *p < 0.05.

As shown in **Figures 2A–C**, each group showed a different bacterial composition at the phylum, family, class, and genus levels. We explored taxa distribution at the phylum, family, and genus levels to reveal distinctive characteristics of each group. *Firmicutes*, *Proteobacteria*, *Bacteroidetes*, *Actinobacteria*, and *Fusobacteria* were the five dominant bacterial phyla in the three groups.

The healthy esophageal microbiota was composed mainly of *Firmicutes* (62.5%), *Proteobacteria* (18.2%), *Bacteroidetes* (13.9%), *Actinobacteria* (2.6%), and *Fusobacteria* (1.3%), with another ~1.5% of unidentified bacteria. At the genus level, *Streptococcus* (24.3%) was the main contributor to the microbiota profile, followed by *Faecalibacterium* and *Bacteroides* (6.1% and 4.3%, respectively); other subdominant genera were *Lactobacillus*, *Neisseria*, *Curvibacter*, and *Blautia*, accounting for about 3% each (**Figures 2A–C**).

The ES group showed a significant decrease of *Firmicutes* (*p* = 0.0370) together with a statistically significant robust increase of *Fusobacteria* (*p* = 0.0280) and *Bacteroidetes* (*p* = 0.0060) with its corresponding genus *Bacteroides* (*p* = 0.0240) as compared to the control group (**Figures 2A–C**).

The ESCC samples also displayed a striking reduction in its microbial composition, such as in *Fusobacteria* (*p* = 0.0010) at phylum level and *Faecalibacterium* (*p* = 0.0010), *Bacteroides* (*p* = 0.0090), *Curvibacter* (*p* = 0.0010), and *Blautia* (*p* = 0.0040) in comparison with the control group at the genus level. We observed an increasing tendency of *Streptococcus* in the ESCC group. When compared with the ES group, fewer *Bacteroidetes* (*p* = 0.0010), *Faecalibacterium* (*p* = 0.0010), *Bacteroides* (*p* = 0.0010), and *Blautia* (*p* = 0.0040) with more *Streptococcus* (*p* = 0.0070) in ESCC tissues were identified (**Figures 2A–C**). In addition, *Megamonas*, *Collinsella*, *Roseburia*, and *Ruminococcus_2* showed a significantly continuous decreasing trend from the control group to the ESCC group at the genus level (**Figure 2D**).

### Characterized Microbial Taxa Associated With Esophageal Squamous Cell Carcinoma Patients

**Figure 3:**
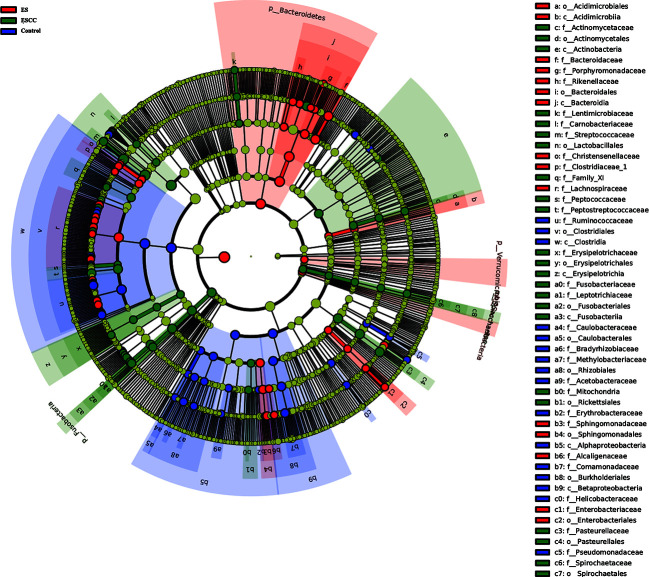
Linear discriminant analysis effect size (LEfSe) analysis showed the most abundant taxa from the phylum to the genus level among the control, esophagitis (ES), and esophageal squamous cell carcinoma (ESCC) groups.

We used multi-level LEfSe analysis to explore potential important microbe biomarkers for the groups in all taxa, and among the three groups, abundance of 138 bacterial species was significantly different. Here, 41, 45, and 52 taxa were abundant in healthy volunteers, ES patients, and ESCC patients, respectively. Given the large number of different bacterial species, we focused on the taxa with LDA scores >4.0. As shown in **Figure 3**, at the genus level, increased *Streptococcus* (LDA score = 4.9115, *p* = 0.0021), *Actinobacillus* (LDA score = 4.5193, *p* < 0.0001), *Peptostreptococcus* (LDA score = 4.3049, *p* < 0.0001), *Fusobacterium* (LDA score = 4.2109, *p* = 0.0004), and *Prevotella* (LDA score = 4.0768, *p* = 0.0020) were identified as powerful markers in ESCC patients. Particularly, *Streptococcus anginosus* at the species level (LDA score = 4.0115, *p* < 0.0001) showed greater abundance in the ESCC group. Besides, we observed a high level of *Roseburia* (LDA score = 4.0412, *p* = 0.0001), *Faecalibacterium* (LDA score = 4.4607, *p* < 0.0001), and *Curvibacter* (LDA score = 4.0812, *p* < 0.0001) at the genus level and *Alphaproteobacteria* (LDA score = 4.2618, *p* = 0.0002) at the class level in the control group. *Bacteroides* (LDA score = 4.6561, *p* = 0.0002) and *Blautia* (LDA score = 4.0883, *p* < 0.0001) at the genus level were abundant in ES patients (**Figure 3**).

### Functional Analysis of Esophageal Microbiota Across The Groups

Finally, PICRUSt was conducted to predict the metagenomes and identify the KEGG pathways involved in each group.

**Figure 4:**
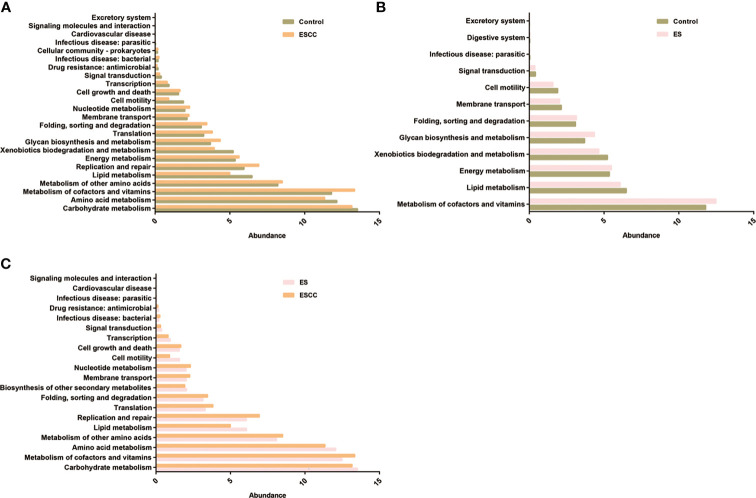
The function prediction of the three groups. Differential Kyoto Encyclopedia of Genes and Genomes (KEGG) pathways were analyzed using PICRUSt for the three groups. Significant differences between the control and esophageal squamous cell carcinoma (ESCC) groups **(A)**, the control and esophagitis (ES) groups **(B)**, and the ES and ESCC groups **(C)** were presented.

Compared with the control group, patients with ESCC showed a significant upregulation of microbial genes involved in signaling molecules and interaction, excretory system, cellular community, cell growth and death, membrane transport, energy metabolism, metabolism of other amino acids, nucleotide metabolism, folding, sorting and degradation, translation, glycan biosynthesis and metabolism, replication and repair, and metabolism of cofactors and vitamins, while there was a reduction of genes related to lipid metabolism, xenobiotics biodegradation and metabolism, cell motility, amino acid metabolism, carbohydrate metabolism, transcription, and signal transduction (**Figure 4A**). On the other hand, microbiota of the ES group was characterized by a higher potential for excretory system, digestive system, folding, sorting and degradation, energy metabolism, glycan biosynthesis and metabolism, and metabolism of cofactors and vitamins while showing reduced xenobiotic biodegradation and metabolism, lipid metabolism, cell motility, membrane transport, and signal transduction (**Figure 4B**).

Moreover, when comparing the ES and ESCC tissues, ESCC-associated microbiota showed significantly increased signaling molecules and interaction, infectious disease, cell growth and death, membrane transport, nucleotide metabolism, folding, sorting and degradation, metabolism of other amino acids, translation, metabolism of cofactors and vitamins, and replication and repair. Conversely, it displayed a consistently decreased lipid metabolism, amino acid metabolism, cell motility, carbohydrate metabolism, transcription, biosynthesis of other secondary metabolites, and signal transduction pathways (**Figure 4C**).

## Discussion

Increasing evidence has shown the crucial roles of bacterial imbalance in tumor development, including esophageal cancers (Garrett, 2015). In China, ESCC constitutes more than 90% of all esophageal cancers (Chen et al., 2021). Although imbalanced microbiome has been well elucidated in EAC, microbial alterations of ESCC were still inconclusive. In the present study, we profiled the structure of esophageal microbiota in the ES and ESCC patients and the matched controls through 16S rRNA gene sequencing and predicted the functional changes. We found a lower microbial diversity in the ESCC patients than in the healthy controls, which was supported by previous findings (Li M. et al., 2020), whereas other studies indicated a decreasing tendency or a higher diversity without significant difference (Shao et al., 2019; Li M. et al., 2020; Yang et al., 2021). Although studies conducted by Li D. et al. (2020) and Castaño-Rodríguez et al. (2017) suggested decreased richness of microbiota in the ESCC patients, our study observed a contrary trend. It could be partly explained by factors that could affect microbial structures such as geographic area or the organ studied. In accordance with previous research, the β diversity was statistically different between the ESCC and the control group (Shao et al., 2019; Li D. et al., 2020). The microbial dysbiosis of ESCC tissues was characterized by decreased *Faecalibacterium*, *Bacteroides*, *Curvibacter*, and *Blautia* and increased *Fusobacteria*.

*Bacteroides* is a predominant member of the gut microbiota, with *Bacteroides fragilis* as the most prevalent form, which was an opportunistic pathogen related to abdominal, soft tissue, and bloodstream infections (Gilbert et al., 2018). Later studies revealed that a subtype of *Bacteroides* could produce a heat-labile toxin named *Bacteroides fragilis* toxin. Secreted toxin promoted interleukin (IL)-18 production and cleared E-cadherin that led to profound inflammation and epithelial homeostasis (Valguarnera and Wardenburg, 2020). Indeed, higher levels of toxigenic *B. fragilis* strains have been reported in secretory diarrhea and various types of cancer including colorectal cancer and prostate cancer (Mármol et al., 2017; Sha et al., 2020). It seems to be inconsistent between our study and previous reports. Yang et al. (2021) have demonstrated significantly enriched *Bacteroides* in ESCC. This discrepancy might be explained by samples isolated from different sites and diverse diets of the individuals recruited. Meanwhile, recent findings also revealed the protective effect of *B. fragilis* in the development of colitis-related colorectal cancer. Thus, we proposed that the *Bacteroides* owned a bidirectional role in the oncogenesis, and a future functional and theoretical investigation is urgent to confirm its role in ESCC.

Specifically, a higher abundance of *S. anginosus* in the ESCC tissues was identified. *S. anginosus*, as an oral bacterium, was frequently found in the oral cavity, gastrointestinal tract, and genitourinary tract (Morita et al., 2003). It occupied up to 82% of patient-unique strains collected from hospitalized patients and was involved in purulent infections, including endocarditis (Siegman-Igra et al., 2012; Kawaguchi et al., 2014). In addition, the presence of *S. anginosus* has been reported in head and neck squamous cell carcinomas, gastric cancer, dysplasia of esophagus, and esophageal cancer tissues, which indicated the involvement of *S. anginosus* in the carcinogenic process (Sasaki et al., 1998; Tateda et al., 2000). Viable *S. anginosus* isolated from the esophageal cancer tissues could adhere to cultured epithelial cells and induce the mRNA expression of two CXC-chemokine genes, IL-8 and growth related oncogene (GRO). These results were supported by a higher content of inflammatory cytokines in esophageal cancer tissues (Narikiyo et al., 2004). Streptolysin S encoded by the sag gene cluster was supposed to be responsible for the cytotoxicity of *S. anginosus* (Asam et al., 2015). The involvement in sulfur metabolism might be the alternative strategy for *S. anginosus* in carcinogenesis (Coker et al., 2018). However, more clinical strains were urgent to better verify the pathogenic genes and cytotoxicity in future studies. Interestingly, increased abundance of *Fusobacterium*, another common elongated anaerobic Gram-negative bacterium of the oral cavity, was also observed in the ESCC tissues. Similar results have been confirmed in previous studies (Li D. et al., 2020; Yang et al., 2021). However, Li D. et al. (2020) found no significant difference of *Fusobacterium* between ESCC and healthy control. *Fusobacterium* caused periodontal disease and was related to the development of human cancers. Increased *Fusobacterium nucleatum* in esophageal cancer was a biomarker for predicting a poor clinical outcome (Yamamura et al., 2016). Experimental studies have shown that *F. nucleatum* could promote carcinogenesis by induction of chemokines and activation of β-catenin signaling pathway (Rubinstein et al., 2013; Yamamura et al., 2016). In our study, we failed to find the different abundance of *Porphyromonas gingivalis* among the three groups, which contributed to the development of ESCC *via* enhancing IL-6 secretion and promoting epithelial–mesenchymal transition (Chen et al., 2021). Because esophageal microbiome was partly shaped by the oral microbiome that linked the periodontal disease to ESCC (Norder et al., 2013), it suggested the possibility of protection against periodontal disease to prevent oncogenesis. This hypothesis was partly supported by the findings that numbers of lost teeth and lifestyle factors, including alcohol use and oral hygiene, were related to increased risk of ESCC (Chen et al., 2017).

Apart from compositional changes in bacterial taxa, we also predicted alterations in function across the groups. Metabolic reprogramming is a hallmark of cancer. Dysregulated metabolites including glucose, lipids, and amino acids have been reported in upper gastrointestinal cancers. For example, increased lactic acid, citrate, and glyceraldehyde were related to gastric cancer and esophageal cancer, although opposite changes were documented by other studies. In our study, we observed a reduction of carbohydrate and amino acid metabolism, which suggested a potential underlying mechanism of ESCC.

Nevertheless, two limitations about this study should be addressed. Firstly, the relatively small sample size of each group limited the generalizability of our findings, and larger studies will provide more credible results. Secondly, a cross-sectional study urged a follow-up prospective trial to fully demonstrate the role of microbiota in esophageal diseases.

## Conclusion

Taken together, our data investigated the microbiota spectrum of ESCC patients and demonstrated a significant difference in the microbial diversity and richness between the ESCC patients and the healthy subjects. Our results provided a potential association of *Streptococcus*, *Actinobacillus*, *Peptostreptococcus*, *Fusobacterium*, and *Prevotella* with ESCC. Further studies are required to confirm our results and elucidate mechanisms of the causal relationship.

## Data Availability Statement

The datasets presented in this study can be found in online repositories. The names of the repository/repositories and accession number(s) can be found below: https://www.ncbi.nlm.nih.gov/, PRJNA759579.

## Ethics Statement

The studies involving human participants were reviewed and approved by Nanjing first hospital. The patients/participants provided their written informed consent to participate in this study.

## Author Contributions

ZJ, ZZ, and SW conceived, organized, and supervised the project and proofread the article. ZJ and ZS collected and analyzed the data and drafted the article. JW supervised statistical analysis. All authors critically revised and approved the final version of the article.

## Funding

This study was supported by the Nanjing Health Science and Technology Development Special Fund Project Plan (YKK20108) and the Science and Technology Development Project of Nanjing Medical University (NMUB2019134).

## Conflict of Interest

The authors declare that the research was conducted in the absence of any commercial or financial relationships that could be construed as a potential conflict of interest.

## Publisher’s Note

All claims expressed in this article are solely those of the authors and do not necessarily represent those of their affiliated organizations, or those of the publisher, the editors and the reviewers. Any product that may be evaluated in this article, or claim that may be made by its manufacturer, is not guaranteed or endorsed by the publisher.

## Supplementary Material

Supplementary Figure 1 Quality Control and Basic Analysis
