# Supplementary figures and images for: Characterization of Esophageal Microbiota in Patients With Esophagitis and Esophageal Squamous Cell Carcinoma
Source: Front Cell Infect Microbiol. 2021 Nov 11;11:774330. doi: 10.3389/fcimb.2021.774330 (PMC8632060; doi:10.3389/fcimb.2021.774330)

# Multy samples Rarefaction Curves

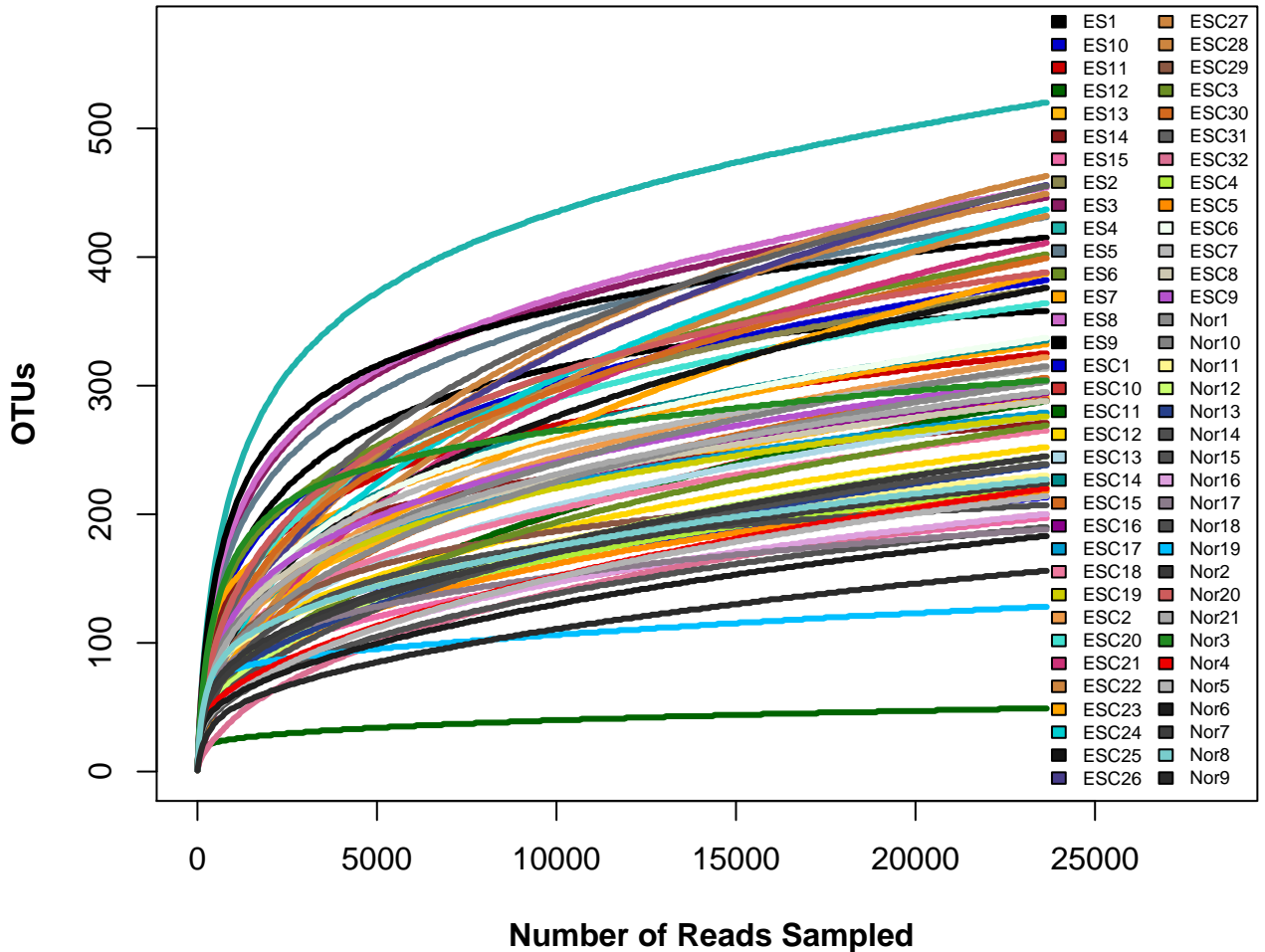

Supplement: Supplementary Figure 1 — Quality Control and Basic Analysis [file Image_1.pdf]

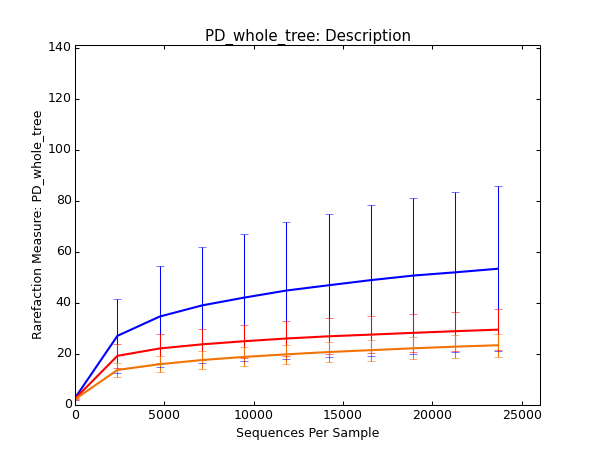

Supplement: Supplementary file 2 [file DataSheet_1.zip › 16S_V3_V4-68╕÷╤∙▒╛JZD 2020.7.21/2.Alpha_diversity_analysis/Alpha_diversity_index/Alpha_detail/alpha_rarefaction_plots/average_plots/PD_whole_treeDescription.png]

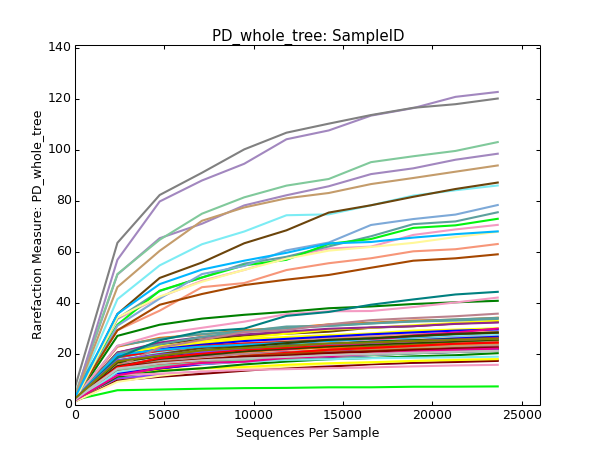

Supplement: Supplementary file 2 [file DataSheet_1.zip › 16S_V3_V4-68╕÷╤∙▒╛JZD 2020.7.21/2.Alpha_diversity_analysis/Alpha_diversity_index/Alpha_detail/alpha_rarefaction_plots/average_plots/PD_whole_treeSampleID.png]

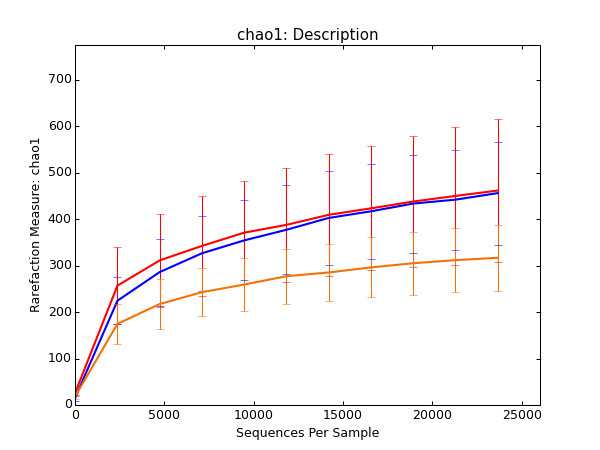

Supplement: Supplementary file 2 [file DataSheet_1.zip › 16S_V3_V4-68╕÷╤∙▒╛JZD 2020.7.21/2.Alpha_diversity_analysis/Alpha_diversity_index/Alpha_detail/alpha_rarefaction_plots/average_plots/chao1Description.png]

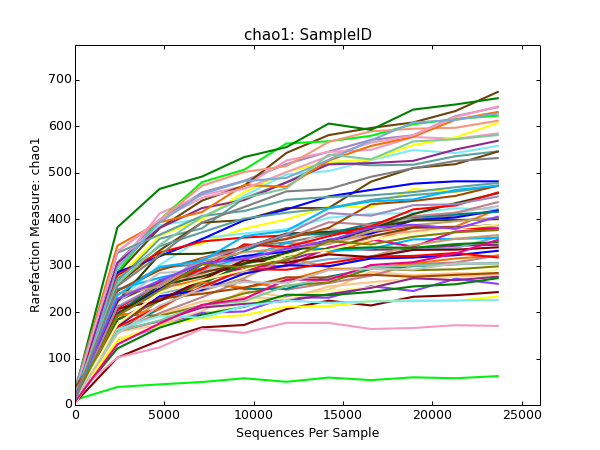

Supplement: Supplementary file 2 [file DataSheet_1.zip › 16S_V3_V4-68╕÷╤∙▒╛JZD 2020.7.21/2.Alpha_diversity_analysis/Alpha_diversity_index/Alpha_detail/alpha_rarefaction_plots/average_plots/chao1SampleID.png]

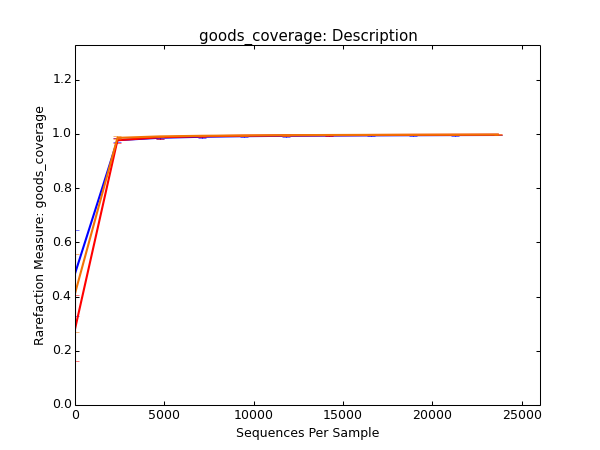

Supplement: Supplementary file 2 [file DataSheet_1.zip › 16S_V3_V4-68╕÷╤∙▒╛JZD 2020.7.21/2.Alpha_diversity_analysis/Alpha_diversity_index/Alpha_detail/alpha_rarefaction_plots/average_plots/goods_coverageDescription.png]

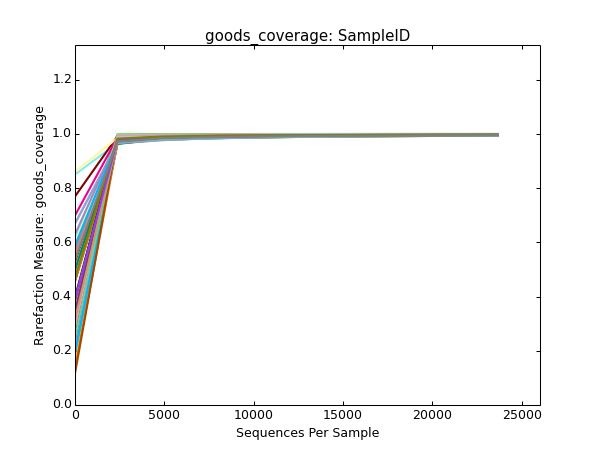

Supplement: Supplementary file 2 [file DataSheet_1.zip › 16S_V3_V4-68╕÷╤∙▒╛JZD 2020.7.21/2.Alpha_diversity_analysis/Alpha_diversity_index/Alpha_detail/alpha_rarefaction_plots/average_plots/goods_coverageSampleID.png]

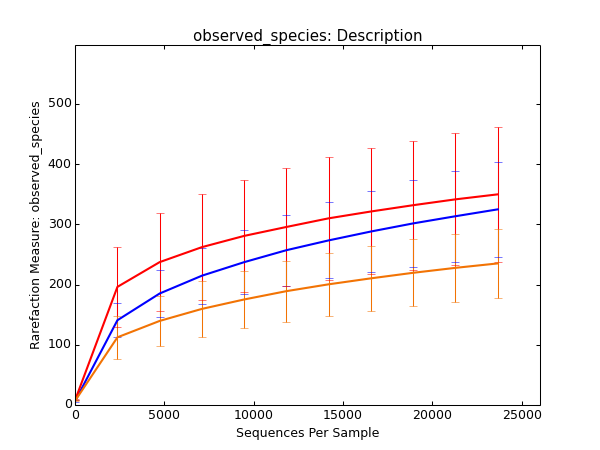

Supplement: Supplementary file 2 [file DataSheet_1.zip › 16S_V3_V4-68╕÷╤∙▒╛JZD 2020.7.21/2.Alpha_diversity_analysis/Alpha_diversity_index/Alpha_detail/alpha_rarefaction_plots/average_plots/observed_speciesDescription.png]

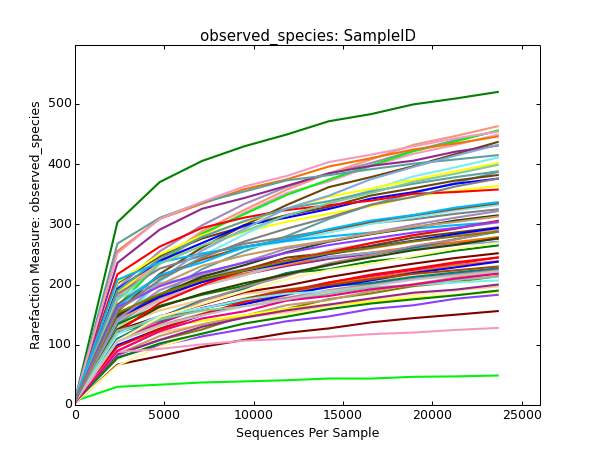

Supplement: Supplementary file 2 [file DataSheet_1.zip › 16S_V3_V4-68╕÷╤∙▒╛JZD 2020.7.21/2.Alpha_diversity_analysis/Alpha_diversity_index/Alpha_detail/alpha_rarefaction_plots/average_plots/observed_speciesSampleID.png]

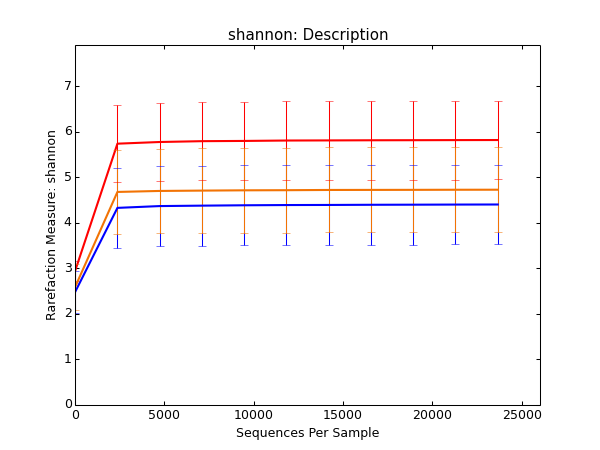

Supplement: Supplementary file 2 [file DataSheet_1.zip › 16S_V3_V4-68╕÷╤∙▒╛JZD 2020.7.21/2.Alpha_diversity_analysis/Alpha_diversity_index/Alpha_detail/alpha_rarefaction_plots/average_plots/shannonDescription.png]

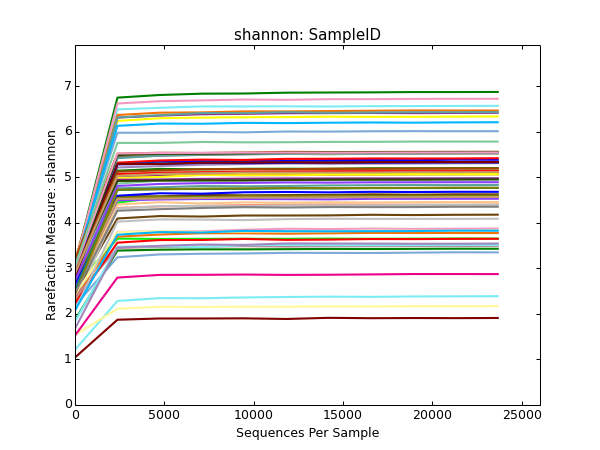

Supplement: Supplementary file 2 [file DataSheet_1.zip › 16S_V3_V4-68╕÷╤∙▒╛JZD 2020.7.21/2.Alpha_diversity_analysis/Alpha_diversity_index/Alpha_detail/alpha_rarefaction_plots/average_plots/shannonSampleID.png]

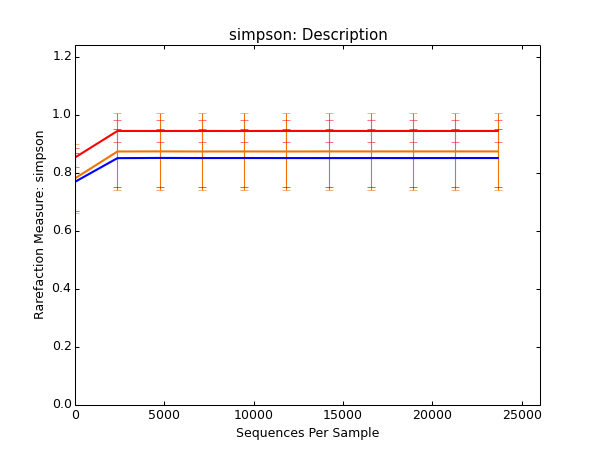

Supplement: Supplementary file 2 [file DataSheet_1.zip › 16S_V3_V4-68╕÷╤∙▒╛JZD 2020.7.21/2.Alpha_diversity_analysis/Alpha_diversity_index/Alpha_detail/alpha_rarefaction_plots/average_plots/simpsonDescription.png]

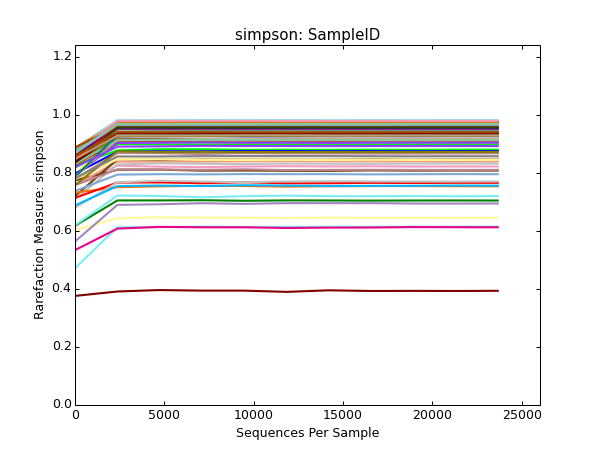

Supplement: Supplementary file 2 [file DataSheet_1.zip › 16S_V3_V4-68╕÷╤∙▒╛JZD 2020.7.21/2.Alpha_diversity_analysis/Alpha_diversity_index/Alpha_detail/alpha_rarefaction_plots/average_plots/simpsonSampleID.png]

PD\_whole\_tree

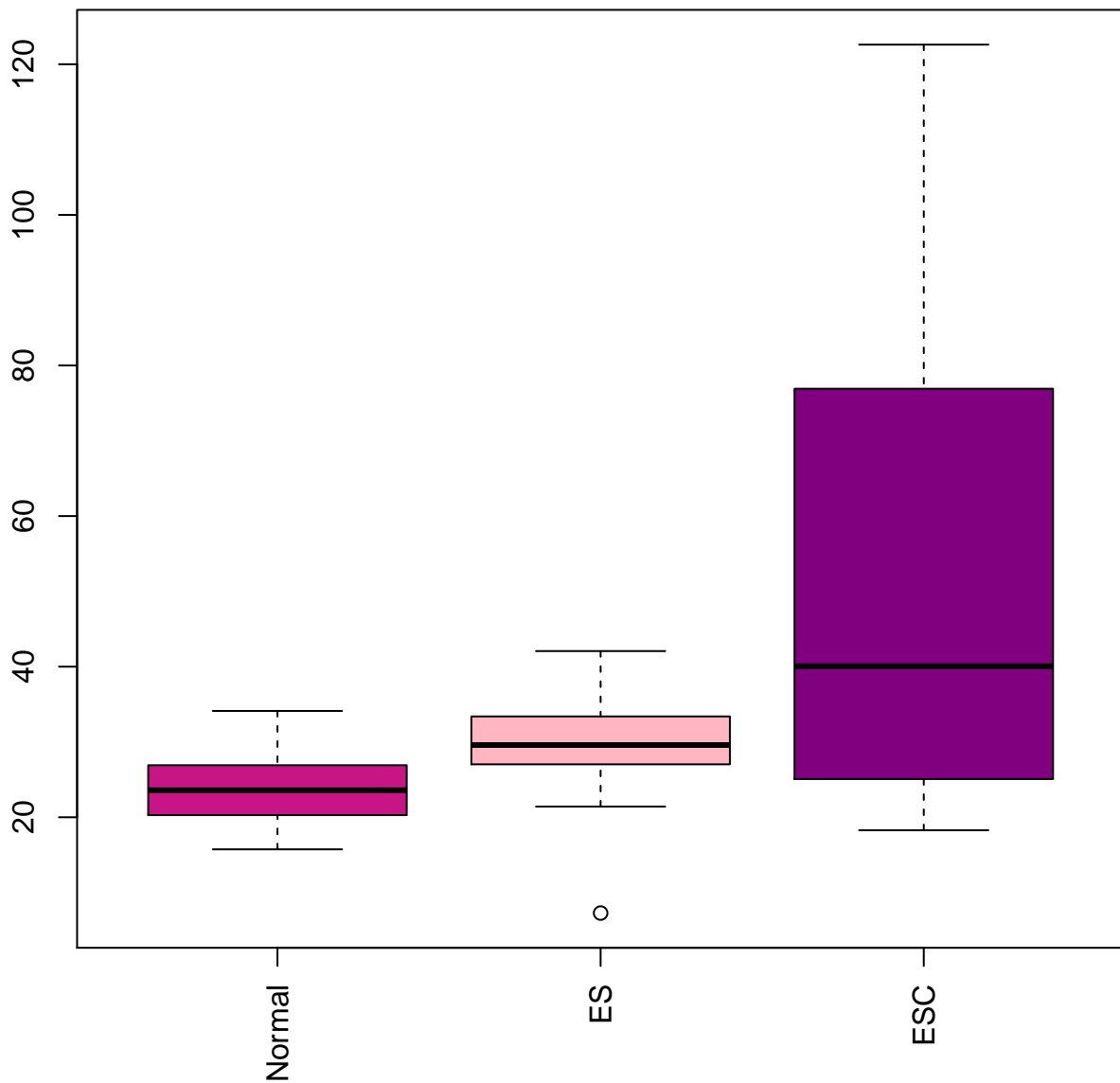

Supplement: Supplementary file 2 [file DataSheet_1.zip › 16S_V3_V4-68╕÷╤∙▒╛JZD 2020.7.21/2.Alpha_diversity_analysis/Alpha_diversity_index/Index_boxplot/PD_whole_tree.pdf]

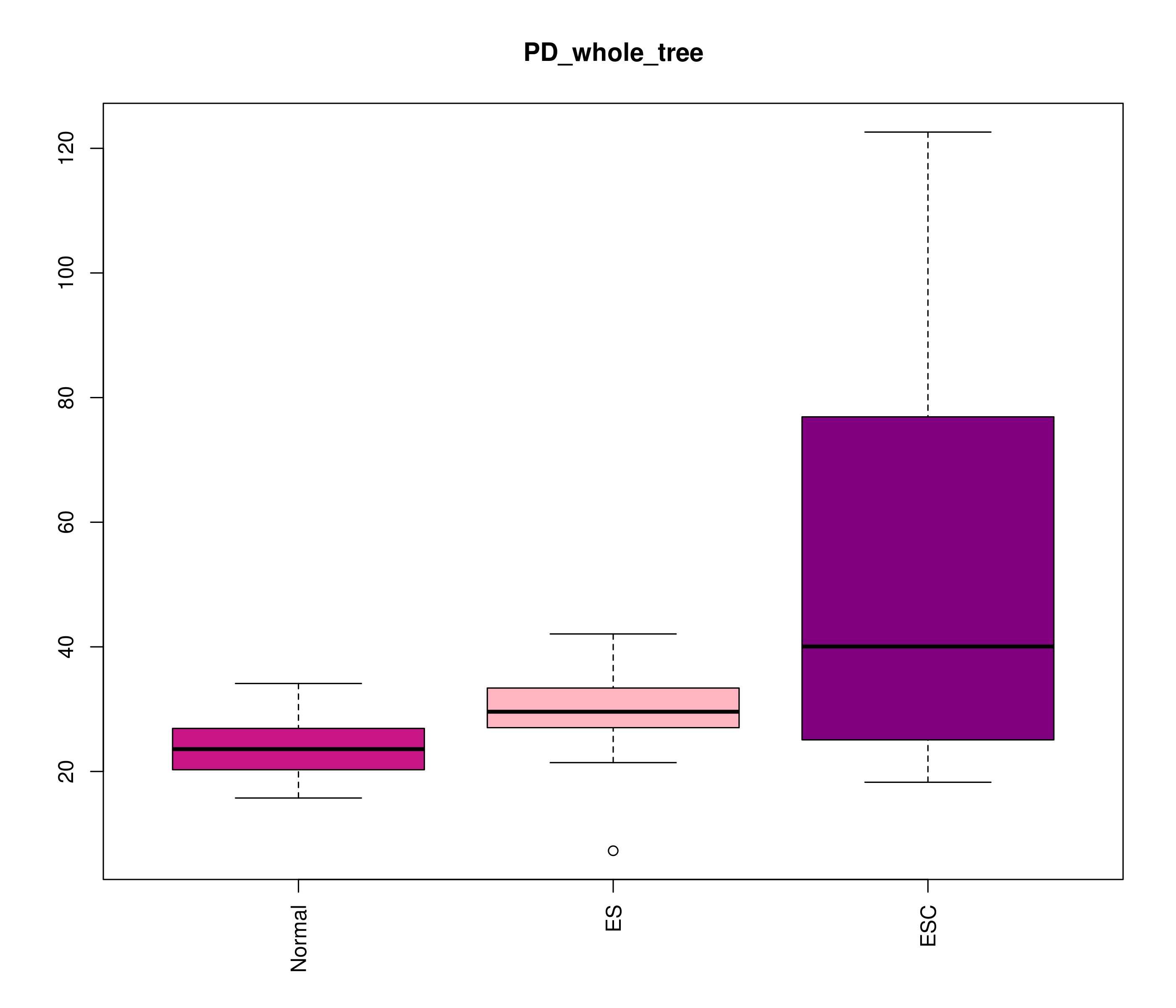

Supplement: Supplementary file 2 [file DataSheet_1.zip › 16S_V3_V4-68╕÷╤∙▒╛JZD 2020.7.21/2.Alpha_diversity_analysis/Alpha_diversity_index/Index_boxplot/PD_whole_tree.png]

**chao1**

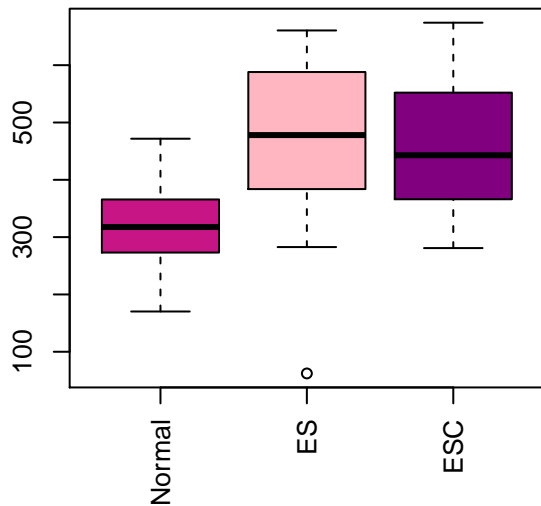

**observed\_species**

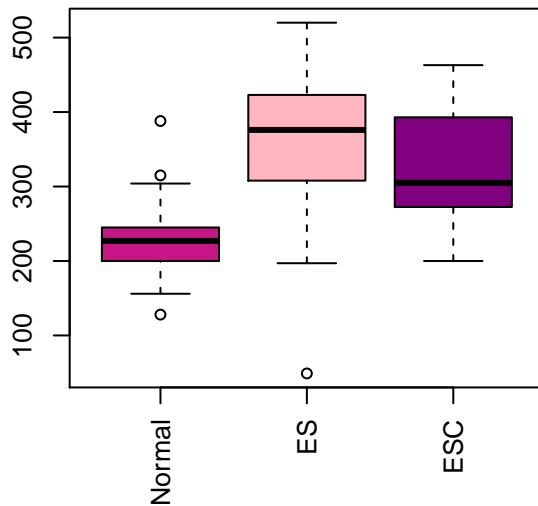

**PD\_whole\_tree**

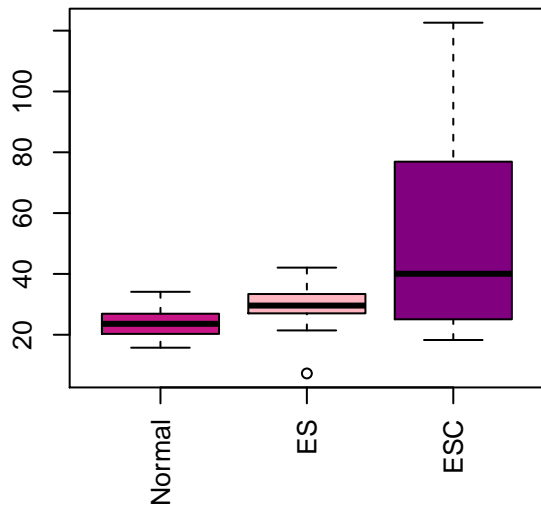

**shannon**

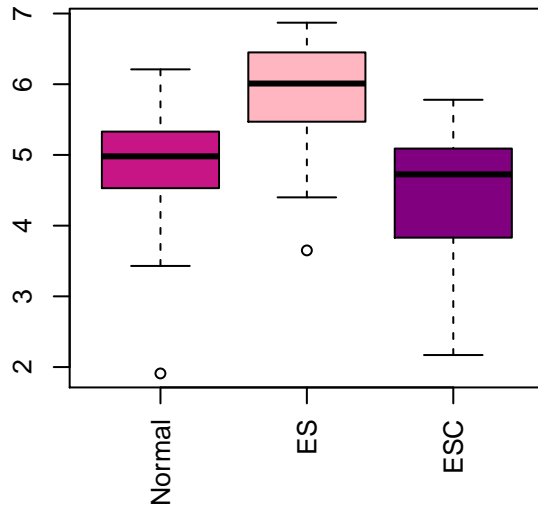

Supplement: Supplementary file 2 [file DataSheet_1.zip › 16S_V3_V4-68╕÷╤∙▒╛JZD 2020.7.21/2.Alpha_diversity_analysis/Alpha_diversity_index/Index_boxplot/alpha.pdf]

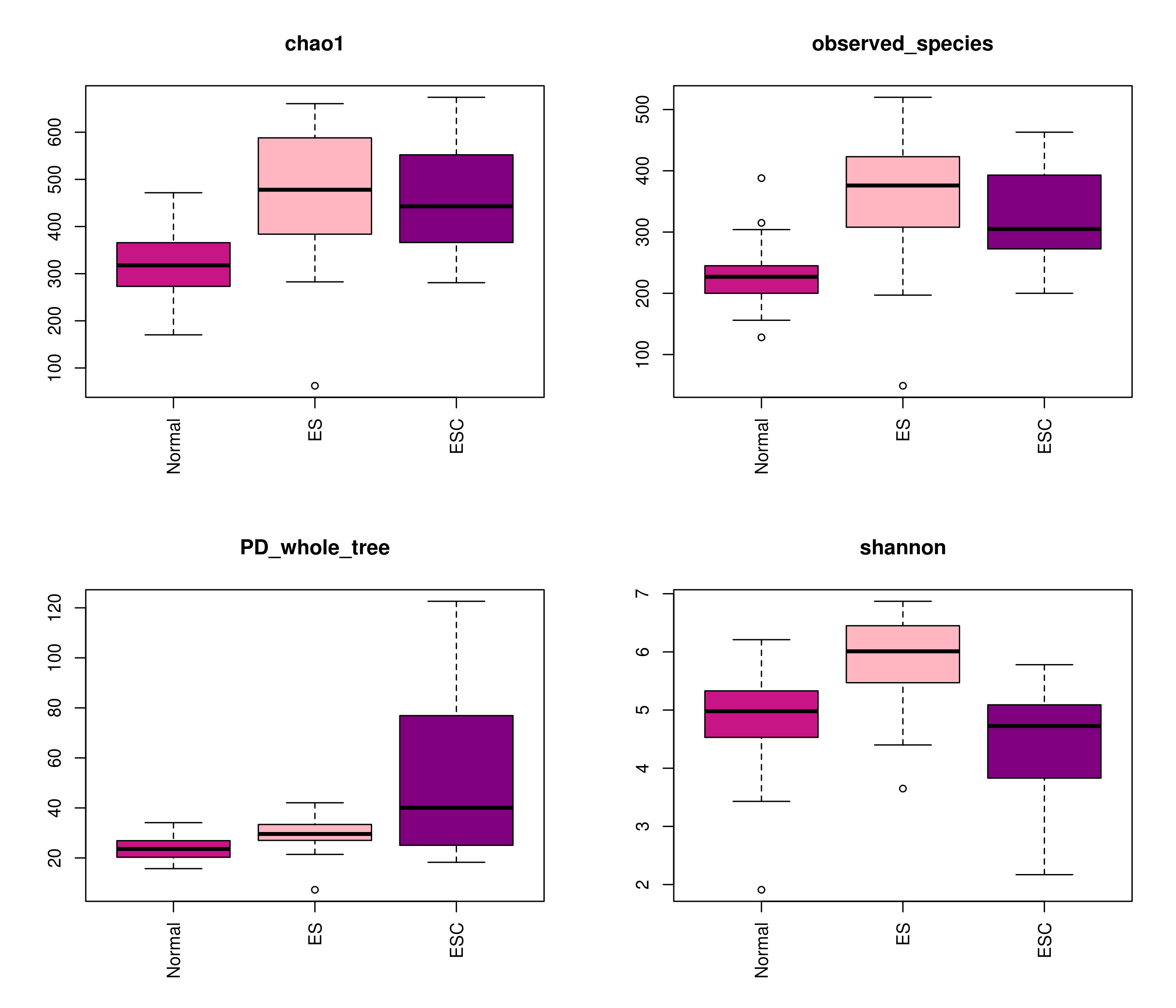

Supplement: Supplementary file 2 [file DataSheet_1.zip › 16S_V3_V4-68╕÷╤∙▒╛JZD 2020.7.21/2.Alpha_diversity_analysis/Alpha_diversity_index/Index_boxplot/alpha.png]

# chao1

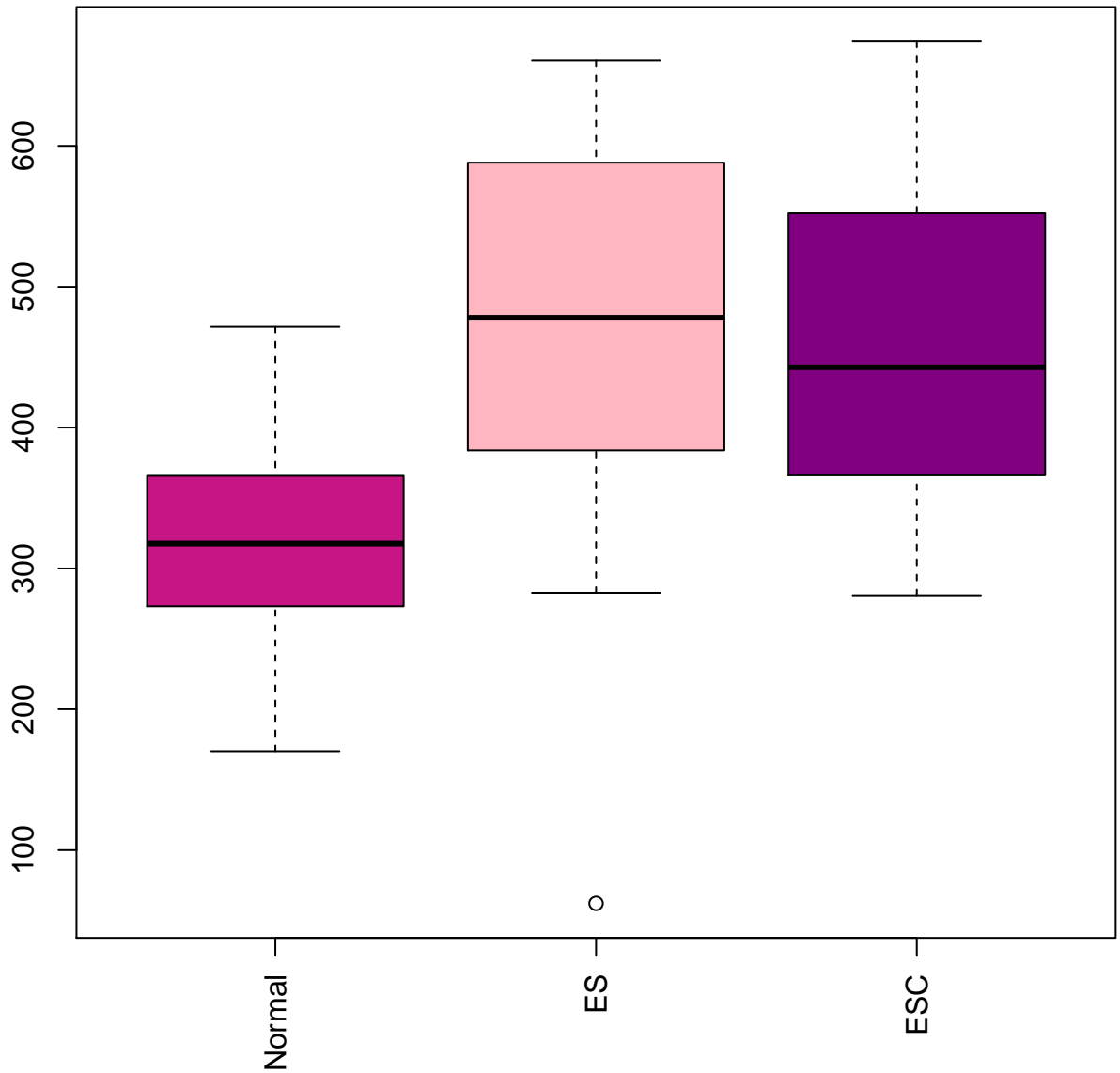

Supplement: Supplementary file 2 [file DataSheet_1.zip › 16S_V3_V4-68╕÷╤∙▒╛JZD 2020.7.21/2.Alpha_diversity_analysis/Alpha_diversity_index/Index_boxplot/chao1.pdf]

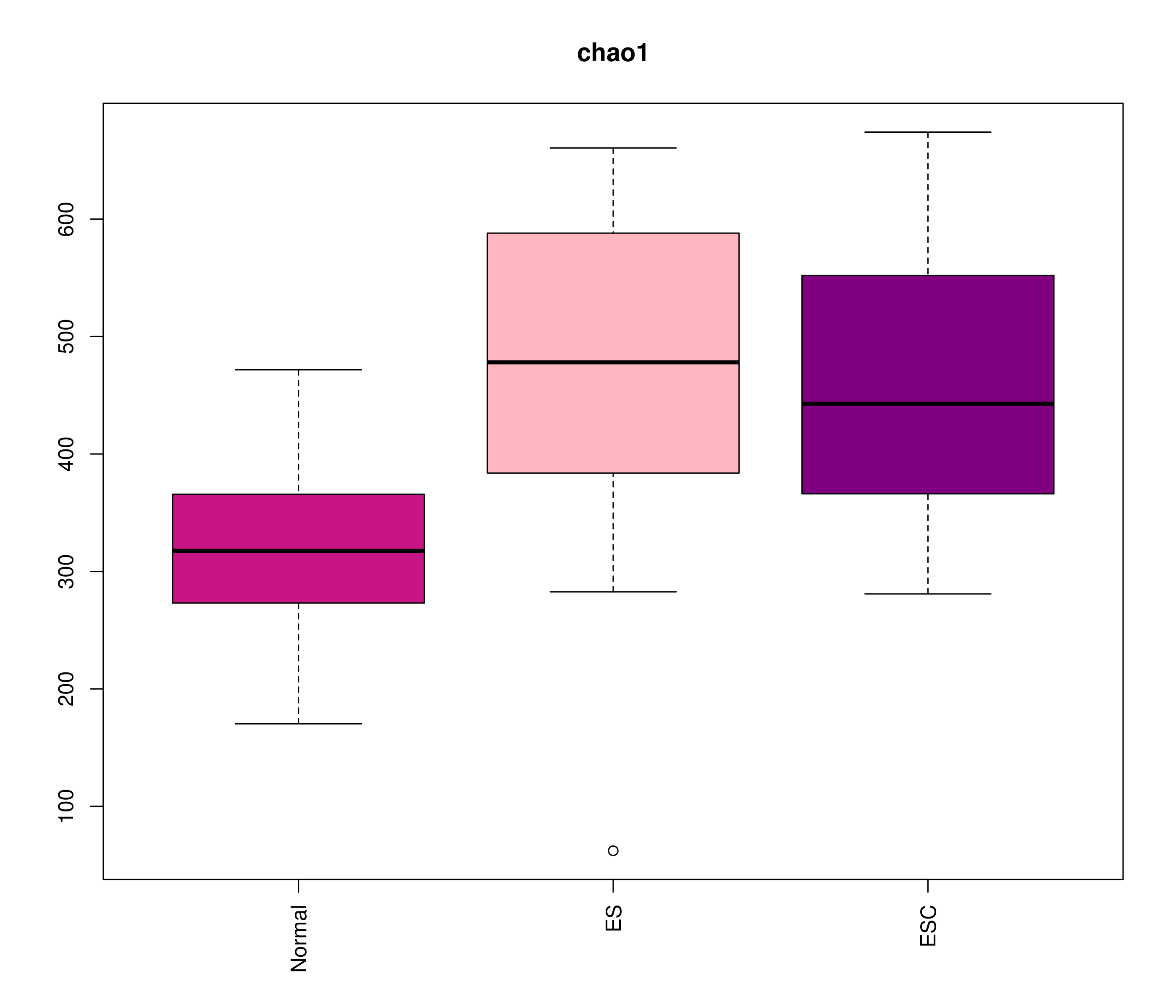

Supplement: Supplementary file 2 [file DataSheet_1.zip › 16S_V3_V4-68╕÷╤∙▒╛JZD 2020.7.21/2.Alpha_diversity_analysis/Alpha_diversity_index/Index_boxplot/chao1.png]

**observed\_species**

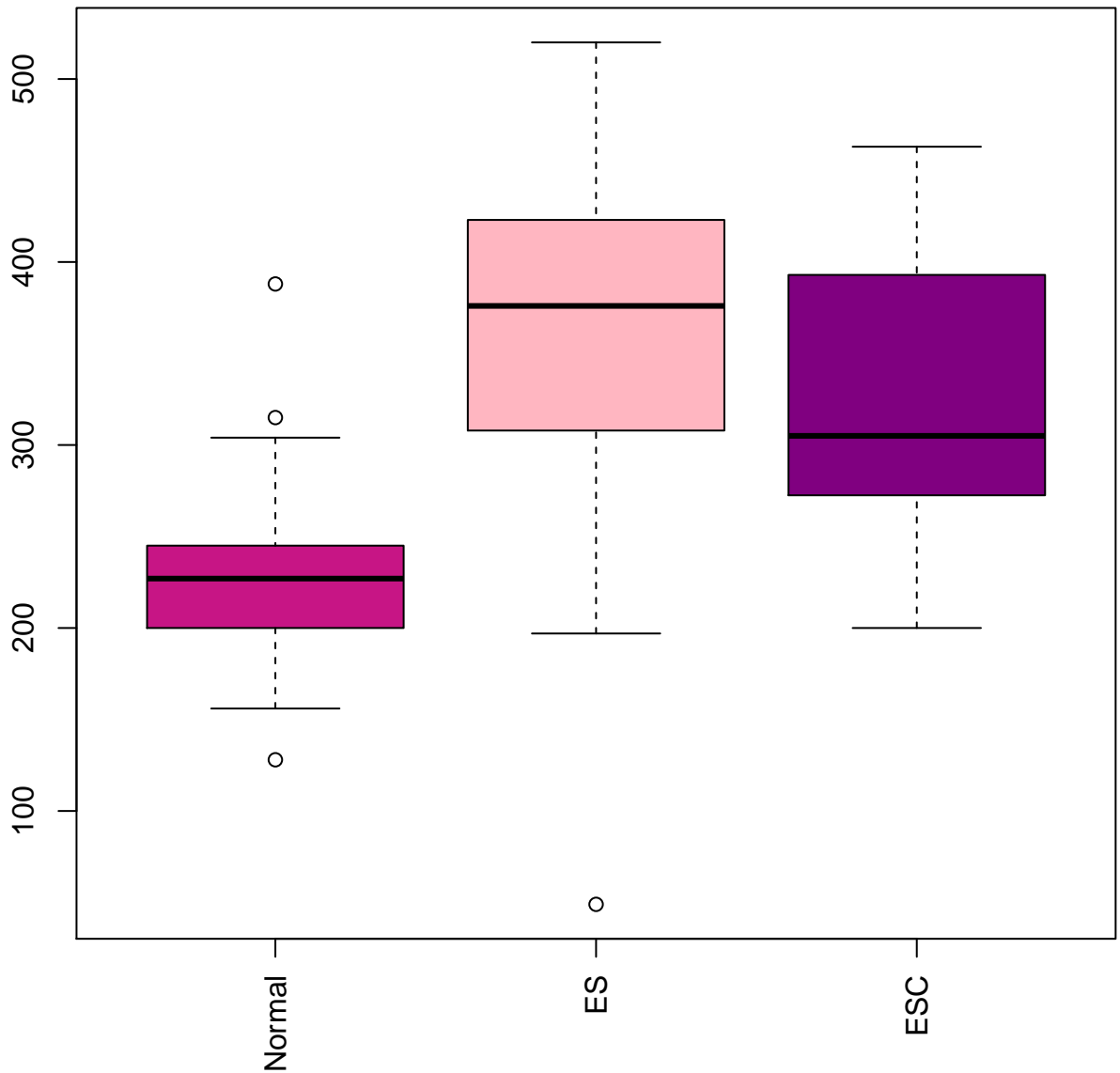

Supplement: Supplementary file 2 [file DataSheet_1.zip › 16S_V3_V4-68╕÷╤∙▒╛JZD 2020.7.21/2.Alpha_diversity_analysis/Alpha_diversity_index/Index_boxplot/observed_species.pdf]

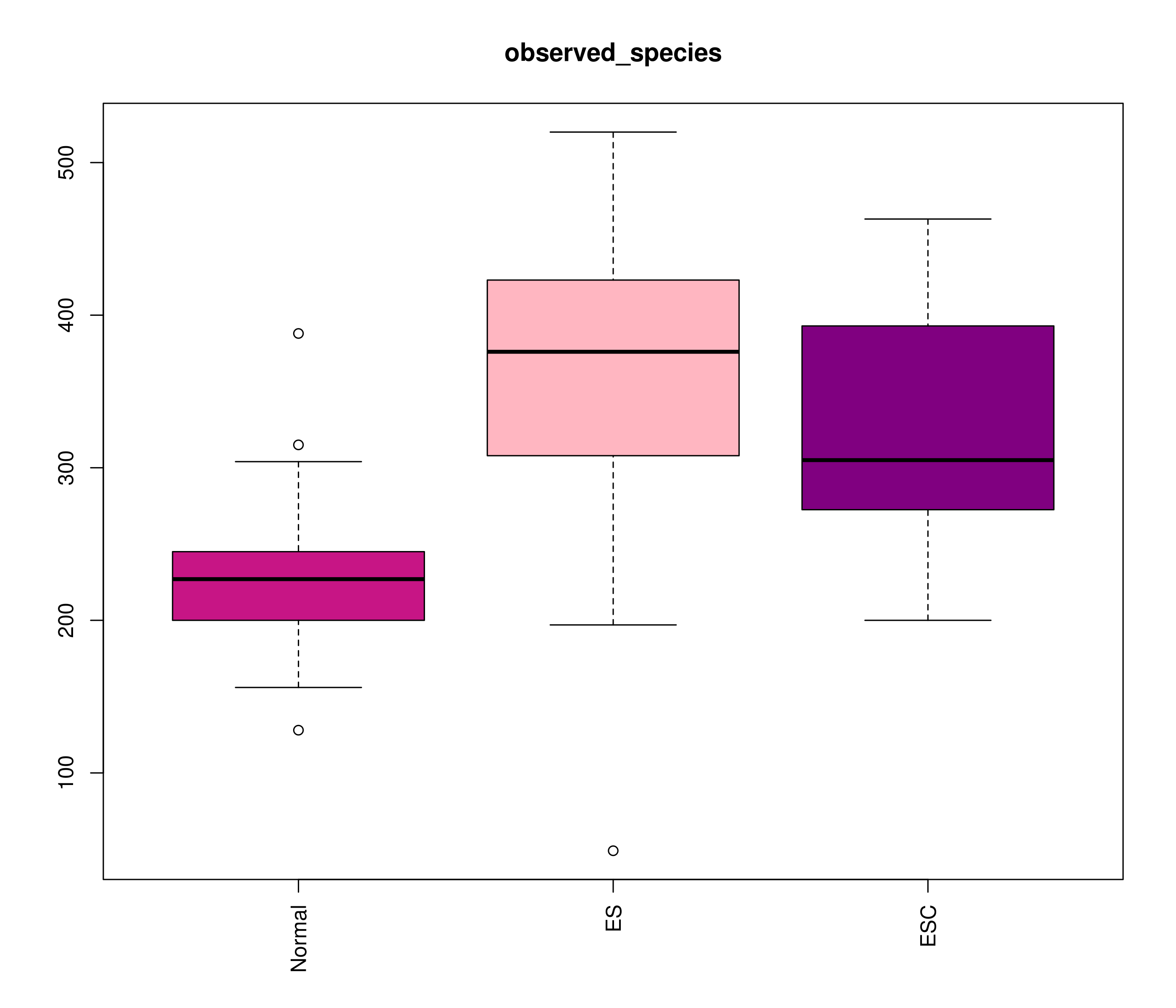

Supplement: Supplementary file 2 [file DataSheet_1.zip › 16S_V3_V4-68╕÷╤∙▒╛JZD 2020.7.21/2.Alpha_diversity_analysis/Alpha_diversity_index/Index_boxplot/observed_species.png]

# shannon

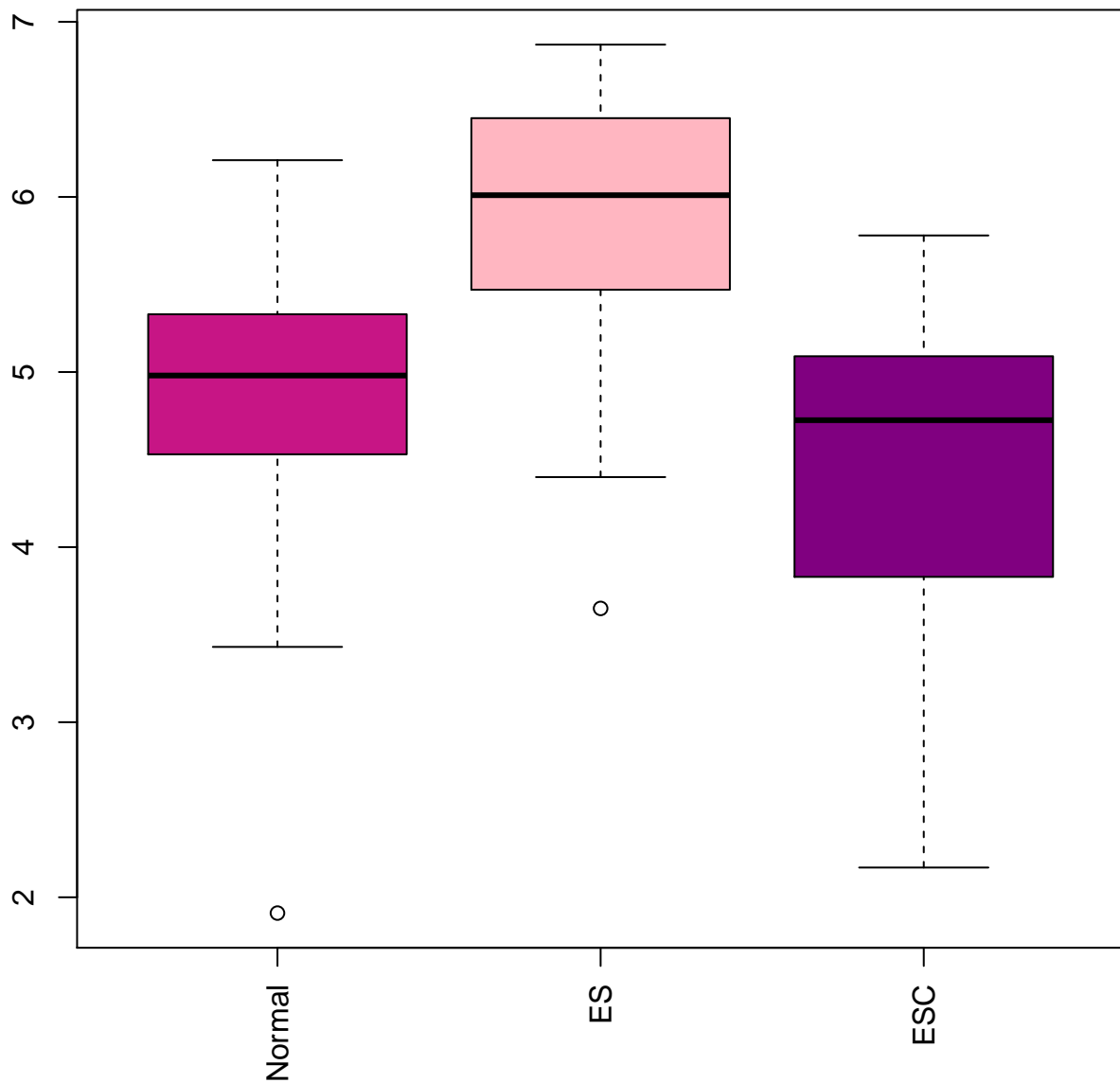

Supplement: Supplementary file 2 [file DataSheet_1.zip › 16S_V3_V4-68╕÷╤∙▒╛JZD 2020.7.21/2.Alpha_diversity_analysis/Alpha_diversity_index/Index_boxplot/shannon.pdf]

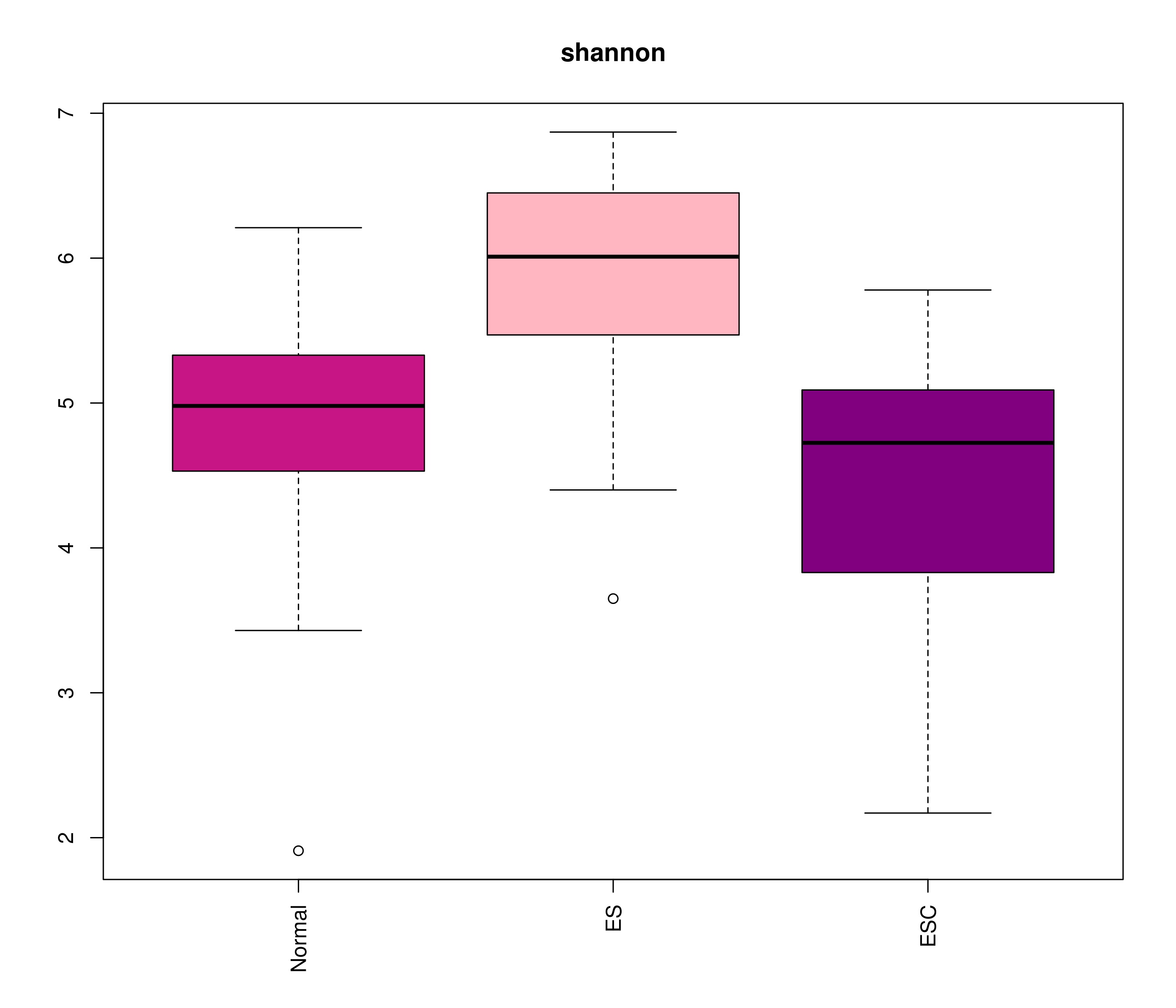

Supplement: Supplementary file 2 [file DataSheet_1.zip › 16S_V3_V4-68╕÷╤∙▒╛JZD 2020.7.21/2.Alpha_diversity_analysis/Alpha_diversity_index/Index_boxplot/shannon.png]

# Multy samples Rarefaction Curves

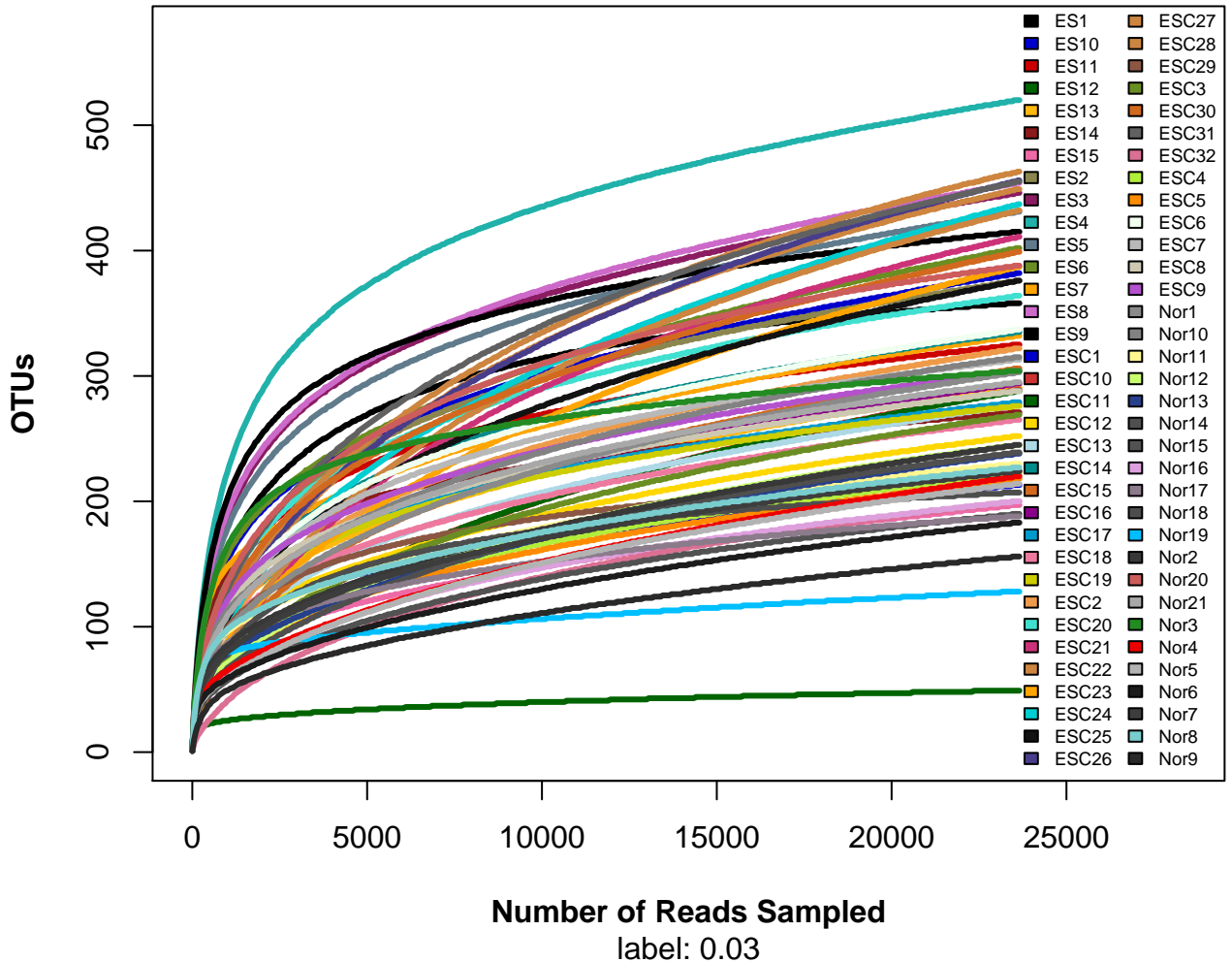

Supplement: Supplementary file 2 [file DataSheet_1.zip › 16S_V3_V4-68╕÷╤∙▒╛JZD 2020.7.21/2.Alpha_diversity_analysis/Rarefaction/rarefaction.All.0.03.pdf]
