# Supplementary material for: Characterization of Esophageal Microbiota in Patients With Esophagitis and Esophageal Squamous Cell Carcinoma
Source: Front Cell Infect Microbiol. 2021 Nov 11;11:774330. doi: 10.3389/fcimb.2021.774330 (PMC8632060; doi:10.3389/fcimb.2021.774330)
Supplement: Supplementary file 2 [file DataSheet_1.zip › 16S_V3_V4-68╕÷╤∙▒╛JZD 2020.7.21/2.Alpha_diversity_analysis/Alpha_diversity_index/Alpha_detail/alpha_rarefaction_plots/rarefaction_plots.html]

 
 
 
   
   Rarefaction Curves 
 
td.data{font-size:10px;border-spacing:0px 10px;text-align:center;}
td.headers{font-size:12px;font-weight:bold;text-align:center;}
table{border-spacing:0px;}
.removed{display:none;}
.expands{cursor:pointer; cursor:hand;}
.child1 td:first-child{padding-left: 3px;}
 
 

function show_hide_category(checkobject){
    var imagetype=document.getElementById('imagetype').value;
    img=document.getElementById(checkobject.name.replace('_raw'+imagetype,'_ave'+imagetype))
    if (checkobject.checked==false){
        img.style.display='none';
    }else{
        img.style.display='';
    }
}

function reset_tree(){
    var category=document.getElementById('category').value;
    var metric=document.getElementById('metric').value;
    var old_all_categories=document.getElementById('all_categories');
    var imagetype=document.getElementById('imagetype').value;
    cat_list=old_all_categories.value.split('$#!')
    if (metric!='' && category != ''){
    for (var i=1, il=cat_list.length; i 

 
 
 
 
 
 
 
 
  
  Select a Metric:  
 
 
   
 PD_whole_tree 
 chao1 
 goods_coverage 
 observed_species 
 shannon 
 simpson 
 
 
    Select a Category:  
 
 
   
 Description 
 SampleID 
 
 
 
 
 
  

 
      Show Categories: 
     
           
         All 
         None 
         Invert 
     
      
 Legend   
  ▶     ■    ES   
  ∟    ◆   ES1   
  ∟    ◆   ES2   
  ∟    ◆   ES3   
  ∟    ◆   ES4   
  ∟    ◆   ES5   
  ∟    ◆   ES6   
  ∟    ◆   ES7   
  ∟    ◆   ES8   
  ∟    ◆   ES9   
  ∟    ◆   ES10   
  ∟    ◆   ES11   
  ∟    ◆   ES12   
  ∟    ◆   ES13   
  ∟    ◆   ES14   
  ∟    ◆   ES15   
  ▶     ■    ESC   
  ∟    ◆   ESC1   
  ∟    ◆   ESC2   
  ∟    ◆   ESC3   
  ∟    ◆   ESC4   
  ∟    ◆   ESC5   
  ∟    ◆   ESC6   
  ∟    ◆   ESC7   
  ∟    ◆   ESC8   
  ∟    ◆   ESC9   
  ∟    ◆   ESC10   
  ∟    ◆   ESC11   
  ∟    ◆   ESC12   
  ∟    ◆   ESC13   
  ∟    ◆   ESC14   
  ∟    ◆   ESC15   
  ∟    ◆   ESC16   
  ∟    ◆   ESC17   
  ∟    ◆   ESC18   
  ∟    ◆   ESC19   
  ∟    ◆   ESC20   
  ∟    ◆   ESC21   
  ∟    ◆   ESC22   
  ∟    ◆   ESC23   
  ∟    ◆   ESC24   
  ∟    ◆   ESC25   
  ∟    ◆   ESC26   
  ∟    ◆   ESC27   
  ∟    ◆   ESC28   
  ∟    ◆   ESC29   
  ∟    ◆   ESC30   
  ∟    ◆   ESC31   
  ∟    ◆   ESC32   
  ▶     ■    Normal   
  ∟    ◆   Nor1   
  ∟    ◆   Nor2   
  ∟    ◆   Nor3   
  ∟    ◆   Nor4   
  ∟    ◆   Nor5   
  ∟    ◆   Nor6   
  ∟    ◆   Nor7   
  ∟    ◆   Nor8   
  ∟    ◆   Nor9   
  ∟    ◆   Nor10   
  ∟    ◆   Nor11   
  ∟    ◆   Nor12   
  ∟    ◆   Nor13   
  ∟    ◆   Nor14   
  ∟    ◆   Nor15   
  ∟    ◆   Nor16   
  ∟    ◆   Nor17   
  ∟    ◆   Nor18   
  ∟    ◆   Nor19   
  ∟    ◆   Nor20   
  ∟    ◆   Nor21   
  ▶     ■    ES1   
  ∟    ◆   ES1   
  ▶     ■    ES2   
  ∟    ◆   ES2   
  ▶     ■    ES3   
  ∟    ◆   ES3   
  ▶     ■    ES4   
  ∟    ◆   ES4   
  ▶     ■    ES5   
  ∟    ◆   ES5   
  ▶     ■    ES6   
  ∟    ◆   ES6   
  ▶     ■    ES7   
  ∟    ◆   ES7   
  ▶     ■    ES8   
  ∟    ◆   ES8   
  ▶     ■    ES9   
  ∟    ◆   ES9   
  ▶     ■    ES10   
  ∟    ◆   ES10   
  ▶     ■    ES11   
  ∟    ◆   ES11   
  ▶     ■    ES12   
  ∟    ◆   ES12   
  ▶     ■    ES13   
  ∟    ◆   ES13   
  ▶     ■    ES14   
  ∟    ◆   ES14   
  ▶     ■    ES15   
  ∟    ◆   ES15   
  ▶     ■    ESC1   
  ∟    ◆   ESC1   
  ▶     ■    ESC2   
  ∟    ◆   ESC2   
  ▶     ■    ESC3   
  ∟    ◆   ESC3   
  ▶     ■    ESC4   
  ∟    ◆   ESC4   
  ▶     ■    ESC5   
  ∟    ◆   ESC5   
  ▶     ■    ESC6   
  ∟    ◆   ESC6   
  ▶     ■    ESC7   
  ∟    ◆   ESC7   
  ▶     ■    ESC8   
  ∟    ◆   ESC8   
  ▶     ■    ESC9   
  ∟    ◆   ESC9   
  ▶     ■    ESC10   
  ∟    ◆   ESC10   
  ▶     ■    ESC11   
  ∟    ◆   ESC11   
  ▶     ■    ESC12   
  ∟    ◆   ESC12   
  ▶     ■    ESC13   
  ∟    ◆   ESC13   
  ▶     ■    ESC14   
  ∟    ◆   ESC14   
  ▶     ■    ESC15   
  ∟    ◆   ESC15   
  ▶     ■    ESC16   
  ∟    ◆   ESC16   
  ▶     ■    ESC17   
  ∟    ◆   ESC17   
  ▶     ■    ESC18   
  ∟    ◆   ESC18   
  ▶     ■    ESC19   
  ∟    ◆   ESC19   
  ▶     ■    ESC20   
  ∟    ◆   ESC20   
  ▶     ■    ESC21   
  ∟    ◆   ESC21   
  ▶     ■    ESC22   
  ∟    ◆   ESC22   
  ▶     ■    ESC23   
  ∟    ◆   ESC23   
  ▶     ■    ESC24   
  ∟    ◆   ESC24   
  ▶     ■    ESC25   
  ∟    ◆   ESC25   
  ▶     ■    ESC26   
  ∟    ◆   ESC26   
  ▶     ■    ESC27   
  ∟    ◆   ESC27   
  ▶     ■    ESC28   
  ∟    ◆   ESC28   
  ▶     ■    ESC29   
  ∟    ◆   ESC29   
  ▶     ■    ESC30   
  ∟    ◆   ESC30   
  ▶     ■    ESC31   
  ∟    ◆   ESC31   
  ▶     ■    ESC32   
  ∟    ◆   ESC32   
  ▶     ■    Nor1   
  ∟    ◆   Nor1   
  ▶     ■    Nor2   
  ∟    ◆   Nor2   
  ▶     ■    Nor3   
  ∟    ◆   Nor3   
  ▶     ■    Nor4   
  ∟    ◆   Nor4   
  ▶     ■    Nor5   
  ∟    ◆   Nor5   
  ▶     ■    Nor6   
  ∟    ◆   Nor6   
  ▶     ■    Nor7   
  ∟    ◆   Nor7   
  ▶     ■    Nor8   
  ∟    ◆   Nor8   
  ▶     ■    Nor9   
  ∟    ◆   Nor9   
  ▶     ■    Nor10   
  ∟    ◆   Nor10   
  ▶     ■    Nor11   
  ∟    ◆   Nor11   
  ▶     ■    Nor12   
  ∟    ◆   Nor12   
  ▶     ■    Nor13   
  ∟    ◆   Nor13   
  ▶     ■    Nor14   
  ∟    ◆   Nor14   
  ▶     ■    Nor15   
  ∟    ◆   Nor15   
  ▶     ■    Nor16   
  ∟    ◆   Nor16   
  ▶     ■    Nor17   
  ∟    ◆   Nor17   
  ▶     ■    Nor18   
  ∟    ◆   Nor18   
  ▶     ■    Nor19   
  ∟    ◆   Nor19   
  ▶     ■    Nor20   
  ∟    ◆   Nor20   
  ▶     ■    Nor21   
  ∟    ◆   Nor21   
  ▶     ■    ES   
  ∟    ◆   ES1   
  ∟    ◆   ES2   
  ∟    ◆   ES3   
  ∟    ◆   ES4   
  ∟    ◆   ES5   
  ∟    ◆   ES6   
  ∟    ◆   ES7   
  ∟    ◆   ES8   
  ∟    ◆   ES9   
  ∟    ◆   ES10   
  ∟    ◆   ES11   
  ∟    ◆   ES12   
  ∟    ◆   ES13   
  ∟    ◆   ES14   
  ∟    ◆   ES15   
  ▶     ■    ESC   
  ∟    ◆   ESC1   
  ∟    ◆   ESC2   
  ∟    ◆   ESC3   
  ∟    ◆   ESC4   
  ∟    ◆   ESC5   
  ∟    ◆   ESC6   
  ∟    ◆   ESC7   
  ∟    ◆   ESC8   
  ∟    ◆   ESC9   
  ∟    ◆   ESC10   
  ∟    ◆   ESC11   
  ∟    ◆   ESC12   
  ∟    ◆   ESC13   
  ∟    ◆   ESC14   
  ∟    ◆   ESC15   
  ∟    ◆   ESC16   
  ∟    ◆   ESC17   
  ∟    ◆   ESC18   
  ∟    ◆   ESC19   
  ∟    ◆   ESC20   
  ∟    ◆   ESC21   
  ∟    ◆   ESC22   
  ∟    ◆   ESC23   
  ∟    ◆   ESC24   
  ∟    ◆   ESC25   
  ∟    ◆   ESC26   
  ∟    ◆   ESC27   
  ∟    ◆   ESC28   
  ∟    ◆   ESC29   
  ∟    ◆   ESC30   
  ∟    ◆   ESC31   
  ∟    ◆   ESC32   
  ▶     ■    Normal   
  ∟    ◆   Nor1   
  ∟    ◆   Nor2   
  ∟    ◆   Nor3   
  ∟    ◆   Nor4   
  ∟    ◆   Nor5   
  ∟    ◆   Nor6   
  ∟    ◆   Nor7   
  ∟    ◆   Nor8   
  ∟    ◆   Nor9   
  ∟    ◆   Nor10   
  ∟    ◆   Nor11   
  ∟    ◆   Nor12   
  ∟    ◆   Nor13   
  ∟    ◆   Nor14   
  ∟    ◆   Nor15   
  ∟    ◆   Nor16   
  ∟    ◆   Nor17   
  ∟    ◆   Nor18   
  ∟    ◆   Nor19   
  ∟    ◆   Nor20   
  ∟    ◆   Nor21   
  ▶     ■    ES1   
  ∟    ◆   ES1   
  ▶     ■    ES2   
  ∟    ◆   ES2   
  ▶     ■    ES3   
  ∟    ◆   ES3   
  ▶     ■    ES4   
  ∟    ◆   ES4   
  ▶     ■    ES5   
  ∟    ◆   ES5   
  ▶     ■    ES6   
  ∟    ◆   ES6   
  ▶     ■    ES7   
  ∟    ◆   ES7   
  ▶     ■    ES8   
  ∟    ◆   ES8   
  ▶     ■    ES9   
  ∟    ◆   ES9   
  ▶     ■    ES10   
  ∟    ◆   ES10   
  ▶     ■    ES11   
  ∟    ◆   ES11   
  ▶     ■    ES12   
  ∟    ◆   ES12   
  ▶     ■    ES13   
  ∟    ◆   ES13   
  ▶     ■    ES14   
  ∟    ◆   ES14   
  ▶     ■    ES15   
  ∟    ◆   ES15   
  ▶     ■    ESC1   
  ∟    ◆   ESC1   
  ▶     ■    ESC2   
  ∟    ◆   ESC2   
  ▶     ■    ESC3   
  ∟    ◆   ESC3   
  ▶     ■    ESC4   
  ∟    ◆   ESC4   
  ▶     ■    ESC5   
  ∟    ◆   ESC5   
  ▶     ■    ESC6   
  ∟    ◆   ESC6   
  ▶     ■    ESC7   
  ∟    ◆   ESC7   
  ▶     ■    ESC8   
  ∟    ◆   ESC8   
  ▶     ■    ESC9   
  ∟    ◆   ESC9   
  ▶     ■    ESC10   
  ∟    ◆   ESC10   
  ▶     ■    ESC11   
  ∟    ◆   ESC11   
  ▶     ■    ESC12   
  ∟    ◆   ESC12   
  ▶     ■    ESC13   
  ∟    ◆   ESC13   
  ▶     ■    ESC14   
  ∟    ◆   ESC14   
  ▶     ■    ESC15   
  ∟    ◆   ESC15   
  ▶     ■    ESC16   
  ∟    ◆   ESC16   
  ▶     ■    ESC17   
  ∟    ◆   ESC17   
  ▶     ■    ESC18   
  ∟    ◆   ESC18   
  ▶     ■    ESC19   
  ∟    ◆   ESC19   
  ▶     ■    ESC20   
  ∟    ◆   ESC20   
  ▶     ■    ESC21   
  ∟    ◆   ESC21   
  ▶     ■    ESC22   
  ∟    ◆   ESC22   
  ▶     ■    ESC23   
  ∟    ◆   ESC23   
  ▶     ■    ESC24   
  ∟    ◆   ESC24   
  ▶     ■    ESC25   
  ∟    ◆   ESC25   
  ▶     ■    ESC26   
  ∟    ◆   ESC26   
  ▶     ■    ESC27   
  ∟    ◆   ESC27   
  ▶     ■    ESC28   
  ∟    ◆   ESC28   
  ▶     ■    ESC29   
  ∟    ◆   ESC29   
  ▶     ■    ESC30   
  ∟    ◆   ESC30   
  ▶     ■    ESC31   
  ∟    ◆   ESC31   
  ▶     ■    ESC32   
  ∟    ◆   ESC32   
  ▶     ■    Nor1   
  ∟    ◆   Nor1   
  ▶     ■    Nor2   
  ∟    ◆   Nor2   
  ▶     ■    Nor3   
  ∟    ◆   Nor3   
  ▶     ■    Nor4   
  ∟    ◆   Nor4   
  ▶     ■    Nor5   
  ∟    ◆   Nor5   
  ▶     ■    Nor6   
  ∟    ◆   Nor6   
  ▶     ■    Nor7   
  ∟    ◆   Nor7   
  ▶     ■    Nor8   
  ∟    ◆   Nor8   
  ▶     ■    Nor9   
  ∟    ◆   Nor9   
  ▶     ■    Nor10   
  ∟    ◆   Nor10   
  ▶     ■    Nor11   
  ∟    ◆   Nor11   
  ▶     ■    Nor12   
  ∟    ◆   Nor12   
  ▶     ■    Nor13   
  ∟    ◆   Nor13   
  ▶     ■    Nor14   
  ∟    ◆   Nor14   
  ▶     ■    Nor15   
  ∟    ◆   Nor15   
  ▶     ■    Nor16   
  ∟    ◆   Nor16   
  ▶     ■    Nor17   
  ∟    ◆   Nor17   
  ▶     ■    Nor18   
  ∟    ◆   Nor18   
  ▶     ■    Nor19   
  ∟    ◆   Nor19   
  ▶     ■    Nor20   
  ∟    ◆   Nor20   
  ▶     ■    Nor21   
  ∟    ◆   Nor21   
  ▶     ■    ES   
  ∟    ◆   ES1   
  ∟    ◆   ES2   
  ∟    ◆   ES3   
  ∟    ◆   ES4   
  ∟    ◆   ES5   
  ∟    ◆   ES6   
  ∟    ◆   ES7   
  ∟    ◆   ES8   
  ∟    ◆   ES9   
  ∟    ◆   ES10   
  ∟    ◆   ES11   
  ∟    ◆   ES12   
  ∟    ◆   ES13   
  ∟    ◆   ES14   
  ∟    ◆   ES15   
  ▶     ■    ESC   
  ∟    ◆   ESC1   
  ∟    ◆   ESC2   
  ∟    ◆   ESC3   
  ∟    ◆   ESC4   
  ∟    ◆   ESC5   
  ∟    ◆   ESC6   
  ∟    ◆   ESC7   
  ∟    ◆   ESC8   
  ∟    ◆   ESC9   
  ∟    ◆   ESC10   
  ∟    ◆   ESC11   
  ∟    ◆   ESC12   
  ∟    ◆   ESC13   
  ∟    ◆   ESC14   
  ∟    ◆   ESC15   
  ∟    ◆   ESC16   
  ∟    ◆   ESC17   
  ∟    ◆   ESC18   
  ∟    ◆   ESC19   
  ∟    ◆   ESC20   
  ∟    ◆   ESC21   
  ∟    ◆   ESC22   
  ∟    ◆   ESC23   
  ∟    ◆   ESC24   
  ∟    ◆   ESC25   
  ∟    ◆   ESC26   
  ∟    ◆   ESC27   
  ∟    ◆   ESC28   
  ∟    ◆   ESC29   
  ∟    ◆   ESC30   
  ∟    ◆   ESC31   
  ∟    ◆   ESC32   
  ▶     ■    Normal   
  ∟    ◆   Nor1   
  ∟    ◆   Nor2   
  ∟    ◆   Nor3   
  ∟    ◆   Nor4   
  ∟    ◆   Nor5   
  ∟    ◆   Nor6   
  ∟    ◆   Nor7   
  ∟    ◆   Nor8   
  ∟    ◆   Nor9   
  ∟    ◆   Nor10   
  ∟    ◆   Nor11   
  ∟    ◆   Nor12   
  ∟    ◆   Nor13   
  ∟    ◆   Nor14   
  ∟    ◆   Nor15   
  ∟    ◆   Nor16   
  ∟    ◆   Nor17   
  ∟    ◆   Nor18   
  ∟    ◆   Nor19   
  ∟    ◆   Nor20   
  ∟    ◆   Nor21   
  ▶     ■    ES1   
  ∟    ◆   ES1   
  ▶     ■    ES2   
  ∟    ◆   ES2   
  ▶     ■    ES3   
  ∟    ◆   ES3   
  ▶     ■    ES4   
  ∟    ◆   ES4   
  ▶     ■    ES5   
  ∟    ◆   ES5   
  ▶     ■    ES6   
  ∟    ◆   ES6   
  ▶     ■    ES7   
  ∟    ◆   ES7   
  ▶     ■    ES8   
  ∟    ◆   ES8   
  ▶     ■    ES9   
  ∟    ◆   ES9   
  ▶     ■    ES10   
  ∟    ◆   ES10   
  ▶     ■    ES11   
  ∟    ◆   ES11   
  ▶     ■    ES12   
  ∟    ◆   ES12   
  ▶     ■    ES13   
  ∟    ◆   ES13   
  ▶     ■    ES14   
  ∟    ◆   ES14   
  ▶     ■    ES15   
  ∟    ◆   ES15   
  ▶     ■    ESC1   
  ∟    ◆   ESC1   
  ▶     ■    ESC2   
  ∟    ◆   ESC2   
  ▶     ■    ESC3   
  ∟    ◆   ESC3   
  ▶     ■    ESC4   
  ∟    ◆   ESC4   
  ▶     ■    ESC5   
  ∟    ◆   ESC5   
  ▶     ■    ESC6   
  ∟    ◆   ESC6   
  ▶     ■    ESC7   
  ∟    ◆   ESC7   
  ▶     ■    ESC8   
  ∟    ◆   ESC8   
  ▶     ■    ESC9   
  ∟    ◆   ESC9   
  ▶     ■    ESC10   
  ∟    ◆   ESC10   
  ▶     ■    ESC11   
  ∟    ◆   ESC11   
  ▶     ■    ESC12   
  ∟    ◆   ESC12   
  ▶     ■    ESC13   
  ∟    ◆   ESC13   
  ▶     ■    ESC14   
  ∟    ◆   ESC14   
  ▶     ■    ESC15   
  ∟    ◆   ESC15   
  ▶     ■    ESC16   
  ∟    ◆   ESC16   
  ▶     ■    ESC17   
  ∟    ◆   ESC17   
  ▶     ■    ESC18   
  ∟    ◆   ESC18   
  ▶     ■    ESC19   
  ∟    ◆   ESC19   
  ▶     ■    ESC20   
  ∟    ◆   ESC20   
  ▶     ■    ESC21   
  ∟    ◆   ESC21   
  ▶     ■    ESC22   
  ∟    ◆   ESC22   
  ▶     ■    ESC23   
  ∟    ◆   ESC23   
  ▶     ■    ESC24   
  ∟    ◆   ESC24   
  ▶     ■    ESC25   
  ∟    ◆   ESC25   
  ▶     ■    ESC26   
  ∟    ◆   ESC26   
  ▶     ■    ESC27   
  ∟    ◆   ESC27   
  ▶     ■    ESC28   
  ∟    ◆   ESC28   
  ▶     ■    ESC29   
  ∟    ◆   ESC29   
  ▶     ■    ESC30   
  ∟    ◆   ESC30   
  ▶     ■    ESC31   
  ∟    ◆   ESC31   
  ▶     ■    ESC32   
  ∟    ◆   ESC32   
  ▶     ■    Nor1   
  ∟    ◆   Nor1   
  ▶     ■    Nor2   
  ∟    ◆   Nor2   
  ▶     ■    Nor3   
  ∟    ◆   Nor3   
  ▶     ■    Nor4   
  ∟    ◆   Nor4   
  ▶     ■    Nor5   
  ∟    ◆   Nor5   
  ▶     ■    Nor6   
  ∟    ◆   Nor6   
  ▶     ■    Nor7   
  ∟    ◆   Nor7   
  ▶     ■    Nor8   
  ∟    ◆   Nor8   
  ▶     ■    Nor9   
  ∟    ◆   Nor9   
  ▶     ■    Nor10   
  ∟    ◆   Nor10   
  ▶     ■    Nor11   
  ∟    ◆   Nor11   
  ▶     ■    Nor12   
  ∟    ◆   Nor12   
  ▶     ■    Nor13   
  ∟    ◆   Nor13   
  ▶     ■    Nor14   
  ∟    ◆   Nor14   
  ▶     ■    Nor15   
  ∟    ◆   Nor15   
  ▶     ■    Nor16   
  ∟    ◆   Nor16   
  ▶     ■    Nor17   
  ∟    ◆   Nor17   
  ▶     ■    Nor18   
  ∟    ◆   Nor18   
  ▶     ■    Nor19   
  ∟    ◆   Nor19   
  ▶     ■    Nor20   
  ∟    ◆   Nor20   
  ▶     ■    Nor21   
  ∟    ◆   Nor21   
  ▶     ■    ES   
  ∟    ◆   ES1   
  ∟    ◆   ES2   
  ∟    ◆   ES3   
  ∟    ◆   ES4   
  ∟    ◆   ES5   
  ∟    ◆   ES6   
  ∟    ◆   ES7   
  ∟    ◆   ES8   
  ∟    ◆   ES9   
  ∟    ◆   ES10   
  ∟    ◆   ES11   
  ∟    ◆   ES12   
  ∟    ◆   ES13   
  ∟    ◆   ES14   
  ∟    ◆   ES15   
  ▶     ■    ESC   
  ∟    ◆   ESC1   
  ∟    ◆   ESC2   
  ∟    ◆   ESC3   
  ∟    ◆   ESC4   
  ∟    ◆   ESC5   
  ∟    ◆   ESC6   
  ∟    ◆   ESC7   
  ∟    ◆   ESC8   
  ∟    ◆   ESC9   
  ∟    ◆   ESC10   
  ∟    ◆   ESC11   
  ∟    ◆   ESC12   
  ∟    ◆   ESC13   
  ∟    ◆   ESC14   
  ∟    ◆   ESC15   
  ∟    ◆   ESC16   
  ∟    ◆   ESC17   
  ∟    ◆   ESC18   
  ∟    ◆   ESC19   
  ∟    ◆   ESC20   
  ∟    ◆   ESC21   
  ∟    ◆   ESC22   
  ∟    ◆   ESC23   
  ∟    ◆   ESC24   
  ∟    ◆   ESC25   
  ∟    ◆   ESC26   
  ∟    ◆   ESC27   
  ∟    ◆   ESC28   
  ∟    ◆   ESC29   
  ∟    ◆   ESC30   
  ∟    ◆   ESC31   
  ∟    ◆   ESC32   
  ▶     ■    Normal   
  ∟    ◆   Nor1   
  ∟    ◆   Nor2   
  ∟    ◆   Nor3   
  ∟    ◆   Nor4   
  ∟    ◆   Nor5   
  ∟    ◆   Nor6   
  ∟    ◆   Nor7   
  ∟    ◆   Nor8   
  ∟    ◆   Nor9   
  ∟    ◆   Nor10   
  ∟    ◆   Nor11   
  ∟    ◆   Nor12   
  ∟    ◆   Nor13   
  ∟    ◆   Nor14   
  ∟    ◆   Nor15   
  ∟    ◆   Nor16   
  ∟    ◆   Nor17   
  ∟    ◆   Nor18   
  ∟    ◆   Nor19   
  ∟    ◆   Nor20   
  ∟    ◆   Nor21   
  ▶     ■    ES1   
  ∟    ◆   ES1   
  ▶     ■    ES2   
  ∟    ◆   ES2   
  ▶     ■    ES3   
  ∟    ◆   ES3   
  ▶     ■    ES4   
  ∟    ◆   ES4   
  ▶     ■    ES5   
  ∟    ◆   ES5   
  ▶     ■    ES6   
  ∟    ◆   ES6   
  ▶     ■    ES7   
  ∟    ◆   ES7   
  ▶     ■    ES8   
  ∟    ◆   ES8   
  ▶     ■    ES9   
  ∟    ◆   ES9   
  ▶     ■    ES10   
  ∟    ◆   ES10   
  ▶     ■    ES11   
  ∟    ◆   ES11   
  ▶     ■    ES12   
  ∟    ◆   ES12   
  ▶     ■    ES13   
  ∟    ◆   ES13   
  ▶     ■    ES14   
  ∟    ◆   ES14   
  ▶     ■    ES15   
  ∟    ◆   ES15   
  ▶     ■    ESC1   
  ∟    ◆   ESC1   
  ▶     ■    ESC2   
  ∟    ◆   ESC2   
  ▶     ■    ESC3   
  ∟    ◆   ESC3   
  ▶     ■    ESC4   
  ∟    ◆   ESC4   
  ▶     ■    ESC5   
  ∟    ◆   ESC5   
  ▶     ■    ESC6   
  ∟    ◆   ESC6   
  ▶     ■    ESC7   
  ∟    ◆   ESC7   
  ▶     ■    ESC8   
  ∟    ◆   ESC8   
  ▶     ■    ESC9   
  ∟    ◆   ESC9   
  ▶     ■    ESC10   
  ∟    ◆   ESC10   
  ▶     ■    ESC11   
  ∟    ◆   ESC11   
  ▶     ■    ESC12   
  ∟    ◆   ESC12   
  ▶     ■    ESC13   
  ∟    ◆   ESC13   
  ▶     ■    ESC14   
  ∟    ◆   ESC14   
  ▶     ■    ESC15   
  ∟    ◆   ESC15   
  ▶     ■    ESC16   
  ∟    ◆   ESC16   
  ▶     ■    ESC17   
  ∟    ◆   ESC17   
  ▶     ■    ESC18   
  ∟    ◆   ESC18   
  ▶     ■    ESC19   
  ∟    ◆   ESC19   
  ▶     ■    ESC20   
  ∟    ◆   ESC20   
  ▶     ■    ESC21   
  ∟    ◆   ESC21   
  ▶     ■    ESC22   
  ∟    ◆   ESC22   
  ▶     ■    ESC23   
  ∟    ◆   ESC23   
  ▶     ■    ESC24   
  ∟    ◆   ESC24   
  ▶     ■    ESC25   
  ∟    ◆   ESC25   
  ▶     ■    ESC26   
  ∟    ◆   ESC26   
  ▶     ■    ESC27   
  ∟    ◆   ESC27   
  ▶     ■    ESC28   
  ∟    ◆   ESC28   
  ▶     ■    ESC29   
  ∟    ◆   ESC29   
  ▶     ■    ESC30   
  ∟    ◆   ESC30   
  ▶     ■    ESC31   
  ∟    ◆   ESC31   
  ▶     ■    ESC32   
  ∟    ◆   ESC32   
  ▶     ■    Nor1   
  ∟    ◆   Nor1   
  ▶     ■    Nor2   
  ∟    ◆   Nor2   
  ▶     ■    Nor3   
  ∟    ◆   Nor3   
  ▶     ■    Nor4   
  ∟    ◆   Nor4   
  ▶     ■    Nor5   
  ∟    ◆   Nor5   
  ▶     ■    Nor6   
  ∟    ◆   Nor6   
  ▶     ■    Nor7   
  ∟    ◆   Nor7   
  ▶     ■    Nor8   
  ∟    ◆   Nor8   
  ▶     ■    Nor9   
  ∟    ◆   Nor9   
  ▶     ■    Nor10   
  ∟    ◆   Nor10   
  ▶     ■    Nor11   
  ∟    ◆   Nor11   
  ▶     ■    Nor12   
  ∟    ◆   Nor12   
  ▶     ■    Nor13   
  ∟    ◆   Nor13   
  ▶     ■    Nor14   
  ∟    ◆   Nor14   
  ▶     ■    Nor15   
  ∟    ◆   Nor15   
  ▶     ■    Nor16   
  ∟    ◆   Nor16   
  ▶     ■    Nor17   
  ∟    ◆   Nor17   
  ▶     ■    Nor18   
  ∟    ◆   Nor18   
  ▶     ■    Nor19   
  ∟    ◆   Nor19   
  ▶     ■    Nor20   
  ∟    ◆   Nor20   
  ▶     ■    Nor21   
  ∟    ◆   Nor21   
  ▶     ■    ES   
  ∟    ◆   ES1   
  ∟    ◆   ES2   
  ∟    ◆   ES3   
  ∟    ◆   ES4   
  ∟    ◆   ES5   
  ∟    ◆   ES6   
  ∟    ◆   ES7   
  ∟    ◆   ES8   
  ∟    ◆   ES9   
  ∟    ◆   ES10   
  ∟    ◆   ES11   
  ∟    ◆   ES12   
  ∟    ◆   ES13   
  ∟    ◆   ES14   
  ∟    ◆   ES15   
  ▶     ■    ESC   
  ∟    ◆   ESC1   
  ∟    ◆   ESC2   
  ∟    ◆   ESC3   
  ∟    ◆   ESC4   
  ∟    ◆   ESC5   
  ∟    ◆   ESC6   
  ∟    ◆   ESC7   
  ∟    ◆   ESC8   
  ∟    ◆   ESC9   
  ∟    ◆   ESC10   
  ∟    ◆   ESC11   
  ∟    ◆   ESC12   
  ∟    ◆   ESC13   
  ∟    ◆   ESC14   
  ∟    ◆   ESC15   
  ∟    ◆   ESC16   
  ∟    ◆   ESC17   
  ∟    ◆   ESC18   
  ∟    ◆   ESC19   
  ∟    ◆   ESC20   
  ∟    ◆   ESC21   
  ∟    ◆   ESC22   
  ∟    ◆   ESC23   
  ∟    ◆   ESC24   
  ∟    ◆   ESC25   
  ∟    ◆   ESC26   
  ∟    ◆   ESC27   
  ∟    ◆   ESC28   
  ∟    ◆   ESC29   
  ∟    ◆   ESC30   
  ∟    ◆   ESC31   
  ∟    ◆   ESC32   
  ▶     ■    Normal   
  ∟    ◆   Nor1   
  ∟    ◆   Nor2   
  ∟    ◆   Nor3   
  ∟    ◆   Nor4   
  ∟    ◆   Nor5   
  ∟    ◆   Nor6   
  ∟    ◆   Nor7   
  ∟    ◆   Nor8   
  ∟    ◆   Nor9   
  ∟    ◆   Nor10   
  ∟    ◆   Nor11   
  ∟    ◆   Nor12   
  ∟    ◆   Nor13   
  ∟    ◆   Nor14   
  ∟    ◆   Nor15   
  ∟    ◆   Nor16   
  ∟    ◆   Nor17   
  ∟    ◆   Nor18   
  ∟    ◆   Nor19   
  ∟    ◆   Nor20   
  ∟    ◆   Nor21   
  ▶     ■    ES1   
  ∟    ◆   ES1   
  ▶     ■    ES2   
  ∟    ◆   ES2   
  ▶     ■    ES3   
  ∟    ◆   ES3   
  ▶     ■    ES4   
  ∟    ◆   ES4   
  ▶     ■    ES5   
  ∟    ◆   ES5   
  ▶     ■    ES6   
  ∟    ◆   ES6   
  ▶     ■    ES7   
  ∟    ◆   ES7   
  ▶     ■    ES8   
  ∟    ◆   ES8   
  ▶     ■    ES9   
  ∟    ◆   ES9   
  ▶     ■    ES10   
  ∟    ◆   ES10   
  ▶     ■    ES11   
  ∟    ◆   ES11   
  ▶     ■    ES12   
  ∟    ◆   ES12   
  ▶     ■    ES13   
  ∟    ◆   ES13   
  ▶     ■    ES14   
  ∟    ◆   ES14   
  ▶     ■    ES15   
  ∟    ◆   ES15   
  ▶     ■    ESC1   
  ∟    ◆   ESC1   
  ▶     ■    ESC2   
  ∟    ◆   ESC2   
  ▶     ■    ESC3   
  ∟    ◆   ESC3   
  ▶     ■    ESC4   
  ∟    ◆   ESC4   
  ▶     ■    ESC5   
  ∟    ◆   ESC5   
  ▶     ■    ESC6   
  ∟    ◆   ESC6   
  ▶     ■    ESC7   
  ∟    ◆   ESC7   
  ▶     ■    ESC8   
  ∟    ◆   ESC8   
  ▶     ■    ESC9   
  ∟    ◆   ESC9   
  ▶     ■    ESC10   
  ∟    ◆   ESC10   
  ▶     ■    ESC11   
  ∟    ◆   ESC11   
  ▶     ■    ESC12   
  ∟    ◆   ESC12   
  ▶     ■    ESC13   
  ∟    ◆   ESC13   
  ▶     ■    ESC14   
  ∟    ◆   ESC14   
  ▶     ■    ESC15   
  ∟    ◆   ESC15   
  ▶     ■    ESC16   
  ∟    ◆   ESC16   
  ▶     ■    ESC17   
  ∟    ◆   ESC17   
  ▶     ■    ESC18   
  ∟    ◆   ESC18   
  ▶     ■    ESC19   
  ∟    ◆   ESC19   
  ▶     ■    ESC20   
  ∟    ◆   ESC20   
  ▶     ■    ESC21   
  ∟    ◆   ESC21   
  ▶     ■    ESC22   
  ∟    ◆   ESC22   
  ▶     ■    ESC23   
  ∟    ◆   ESC23   
  ▶     ■    ESC24   
  ∟    ◆   ESC24   
  ▶     ■    ESC25   
  ∟    ◆   ESC25   
  ▶     ■    ESC26   
  ∟    ◆   ESC26   
  ▶     ■    ESC27   
  ∟    ◆   ESC27   
  ▶     ■    ESC28   
  ∟    ◆   ESC28   
  ▶     ■    ESC29   
  ∟    ◆   ESC29   
  ▶     ■    ESC30   
  ∟    ◆   ESC30   
  ▶     ■    ESC31   
  ∟    ◆   ESC31   
  ▶     ■    ESC32   
  ∟    ◆   ESC32   
  ▶     ■    Nor1   
  ∟    ◆   Nor1   
  ▶     ■    Nor2   
  ∟    ◆   Nor2   
  ▶     ■    Nor3   
  ∟    ◆   Nor3   
  ▶     ■    Nor4   
  ∟    ◆   Nor4   
  ▶     ■    Nor5   
  ∟    ◆   Nor5   
  ▶     ■    Nor6   
  ∟    ◆   Nor6   
  ▶     ■    Nor7   
  ∟    ◆   Nor7   
  ▶     ■    Nor8   
  ∟    ◆   Nor8   
  ▶     ■    Nor9   
  ∟    ◆   Nor9   
  ▶     ■    Nor10   
  ∟    ◆   Nor10   
  ▶     ■    Nor11   
  ∟    ◆   Nor11   
  ▶     ■    Nor12   
  ∟    ◆   Nor12   
  ▶     ■    Nor13   
  ∟    ◆   Nor13   
  ▶     ■    Nor14   
  ∟    ◆   Nor14   
  ▶     ■    Nor15   
  ∟    ◆   Nor15   
  ▶     ■    Nor16   
  ∟    ◆   Nor16   
  ▶     ■    Nor17   
  ∟    ◆   Nor17   
  ▶     ■    Nor18   
  ∟    ◆   Nor18   
  ▶     ■    Nor19   
  ∟    ◆   Nor19   
  ▶     ■    Nor20   
  ∟    ◆   Nor20   
  ▶     ■    Nor21   
  ∟    ◆   Nor21   
  ▶     ■    ES   
  ∟    ◆   ES1   
  ∟    ◆   ES2   
  ∟    ◆   ES3   
  ∟    ◆   ES4   
  ∟    ◆   ES5   
  ∟    ◆   ES6   
  ∟    ◆   ES7   
  ∟    ◆   ES8   
  ∟    ◆   ES9   
  ∟    ◆   ES10   
  ∟    ◆   ES11   
  ∟    ◆   ES12   
  ∟    ◆   ES13   
  ∟    ◆   ES14   
  ∟    ◆   ES15   
  ▶     ■    ESC   
  ∟    ◆   ESC1   
  ∟    ◆   ESC2   
  ∟    ◆   ESC3   
  ∟    ◆   ESC4   
  ∟    ◆   ESC5   
  ∟    ◆   ESC6   
  ∟    ◆   ESC7   
  ∟    ◆   ESC8   
  ∟    ◆   ESC9   
  ∟    ◆   ESC10   
  ∟    ◆   ESC11   
  ∟    ◆   ESC12   
  ∟    ◆   ESC13   
  ∟    ◆   ESC14   
  ∟    ◆   ESC15   
  ∟    ◆   ESC16   
  ∟    ◆   ESC17   
  ∟    ◆   ESC18   
  ∟    ◆   ESC19   
  ∟    ◆   ESC20   
  ∟    ◆   ESC21   
  ∟    ◆   ESC22   
  ∟    ◆   ESC23   
  ∟    ◆   ESC24   
  ∟    ◆   ESC25   
  ∟    ◆   ESC26   
  ∟    ◆   ESC27   
  ∟    ◆   ESC28   
  ∟    ◆   ESC29   
  ∟    ◆   ESC30   
  ∟    ◆   ESC31   
  ∟    ◆   ESC32   
  ▶     ■    Normal   
  ∟    ◆   Nor1   
  ∟    ◆   Nor2   
  ∟    ◆   Nor3   
  ∟    ◆   Nor4   
  ∟    ◆   Nor5   
  ∟    ◆   Nor6   
  ∟    ◆   Nor7   
  ∟    ◆   Nor8   
  ∟    ◆   Nor9   
  ∟    ◆   Nor10   
  ∟    ◆   Nor11   
  ∟    ◆   Nor12   
  ∟    ◆   Nor13   
  ∟    ◆   Nor14   
  ∟    ◆   Nor15   
  ∟    ◆   Nor16   
  ∟    ◆   Nor17   
  ∟    ◆   Nor18   
  ∟    ◆   Nor19   
  ∟    ◆   Nor20   
  ∟    ◆   Nor21   
  ▶     ■    ES1   
  ∟    ◆   ES1   
  ▶     ■    ES2   
  ∟    ◆   ES2   
  ▶     ■    ES3   
  ∟    ◆   ES3   
  ▶     ■    ES4   
  ∟    ◆   ES4   
  ▶     ■    ES5   
  ∟    ◆   ES5   
  ▶     ■    ES6   
  ∟    ◆   ES6   
  ▶     ■    ES7   
  ∟    ◆   ES7   
  ▶     ■    ES8   
  ∟    ◆   ES8   
  ▶     ■    ES9   
  ∟    ◆   ES9   
  ▶     ■    ES10   
  ∟    ◆   ES10   
  ▶     ■    ES11   
  ∟    ◆   ES11   
  ▶     ■    ES12   
  ∟    ◆   ES12   
  ▶     ■    ES13   
  ∟    ◆   ES13   
  ▶     ■    ES14   
  ∟    ◆   ES14   
  ▶     ■    ES15   
  ∟    ◆   ES15   
  ▶     ■    ESC1   
  ∟    ◆   ESC1   
  ▶     ■    ESC2   
  ∟    ◆   ESC2   
  ▶     ■    ESC3   
  ∟    ◆   ESC3   
  ▶     ■    ESC4   
  ∟    ◆   ESC4   
  ▶     ■    ESC5   
  ∟    ◆   ESC5   
  ▶     ■    ESC6   
  ∟    ◆   ESC6   
  ▶     ■    ESC7   
  ∟    ◆   ESC7   
  ▶     ■    ESC8   
  ∟    ◆   ESC8   
  ▶     ■    ESC9   
  ∟    ◆   ESC9   
  ▶     ■    ESC10   
  ∟    ◆   ESC10   
  ▶     ■    ESC11   
  ∟    ◆   ESC11   
  ▶     ■    ESC12   
  ∟    ◆   ESC12   
  ▶     ■    ESC13   
  ∟    ◆   ESC13   
  ▶     ■    ESC14   
  ∟    ◆   ESC14   
  ▶     ■    ESC15   
  ∟    ◆   ESC15   
  ▶     ■    ESC16   
  ∟    ◆   ESC16   
  ▶     ■    ESC17   
  ∟    ◆   ESC17   
  ▶     ■    ESC18   
  ∟    ◆   ESC18   
  ▶     ■    ESC19   
  ∟    ◆   ESC19   
  ▶     ■    ESC20   
  ∟    ◆   ESC20   
  ▶     ■    ESC21   
  ∟    ◆   ESC21   
  ▶     ■    ESC22   
  ∟    ◆   ESC22   
  ▶     ■    ESC23   
  ∟    ◆   ESC23   
  ▶     ■    ESC24   
  ∟    ◆   ESC24   
  ▶     ■    ESC25   
  ∟    ◆   ESC25   
  ▶     ■    ESC26   
  ∟    ◆   ESC26   
  ▶     ■    ESC27   
  ∟    ◆   ESC27   
  ▶     ■    ESC28   
  ∟    ◆   ESC28   
  ▶     ■    ESC29   
  ∟    ◆   ESC29   
  ▶     ■    ESC30   
  ∟    ◆   ESC30   
  ▶     ■    ESC31   
  ∟    ◆   ESC31   
  ▶     ■    ESC32   
  ∟    ◆   ESC32   
  ▶     ■    Nor1   
  ∟    ◆   Nor1   
  ▶     ■    Nor2   
  ∟    ◆   Nor2   
  ▶     ■    Nor3   
  ∟    ◆   Nor3   
  ▶     ■    Nor4   
  ∟    ◆   Nor4   
  ▶     ■    Nor5   
  ∟    ◆   Nor5   
  ▶     ■    Nor6   
  ∟    ◆   Nor6   
  ▶     ■    Nor7   
  ∟    ◆   Nor7   
  ▶     ■    Nor8   
  ∟    ◆   Nor8   
  ▶     ■    Nor9   
  ∟    ◆   Nor9   
  ▶     ■    Nor10   
  ∟    ◆   Nor10   
  ▶     ■    Nor11   
  ∟    ◆   Nor11   
  ▶     ■    Nor12   
  ∟    ◆   Nor12   
  ▶     ■    Nor13   
  ∟    ◆   Nor13   
  ▶     ■    Nor14   
  ∟    ◆   Nor14   
  ▶     ■    Nor15   
  ∟    ◆   Nor15   
  ▶     ■    Nor16   
  ∟    ◆   Nor16   
  ▶     ■    Nor17   
  ∟    ◆   Nor17   
  ▶     ■    Nor18   
  ∟    ◆   Nor18   
  ▶     ■    Nor19   
  ∟    ◆   Nor19   
  ▶     ■    Nor20   
  ∟    ◆   Nor20   
  ▶     ■    Nor21   
  ∟    ◆   Nor21   
   
 
 
 If the lines for some categories do not extend all the way to the right end of the x-axis, that means that at least one of the samples in that category does not have that many samples. 
 
  
 
  Description  Seqs/Sample 
 PD_whole_tree Ave.  PD_whole_tree Err. 
 chao1 Ave.  chao1 Err. 
 goods_coverage Ave.  goods_coverage Err. 
 observed_species Ave.  observed_species Err. 
 shannon Ave.  shannon Err. 
 simpson Ave.  simpson Err. 
 
 
 ES  10.0 
      2.541       0.227 
     26.341       6.984 
      0.283       0.122 
      8.353       0.834 
      2.947       0.218 
      0.853       0.034 
 
 ES  2374.0 
     19.263       4.625 
    257.118      82.705 
      0.976       0.009 
    196.240      66.364 
      5.738       0.843 
      0.944       0.039 
 
 ES  4738.0 
     22.143       5.562 
    311.362      99.366 
      0.987       0.005 
    237.373      80.843 
      5.775       0.855 
      0.944       0.039 
 
 ES  7102.0 
     23.759       6.041 
    342.603     107.580 
      0.991       0.003 
    262.240      88.229 
      5.793       0.861 
      0.944       0.039 
 
 ES  9466.0 
     24.981       6.397 
    371.130     111.592 
      0.993       0.002 
    280.913      93.473 
      5.798       0.862 
      0.944       0.039 
 
 ES  11830.0 
     26.051       6.830 
    387.945     122.213 
      0.994       0.002 
    295.753      97.495 
      5.807       0.863 
      0.944       0.039 
 
 ES  14194.0 
     26.909       7.091 
    409.547     131.989 
      0.995       0.002 
    310.113     101.953 
      5.810       0.862 
      0.944       0.039 
 
 ES  16558.0 
     27.576       7.197 
    423.506     133.419 
      0.995       0.002 
    321.453     104.634 
      5.813       0.864 
      0.944       0.039 
 
 ES  18922.0 
     28.262       7.421 
    438.156     140.619 
      0.996       0.001 
    331.920     107.382 
      5.814       0.866 
      0.944       0.039 
 
 ES  21286.0 
     28.912       7.687 
    450.208     148.891 
      0.996       0.001 
    341.613     109.878 
      5.816       0.866 
      0.944       0.039 
 
 ES  23650.0 
     29.513       7.962 
    461.816     154.703 
      0.996       0.001 
    349.913     112.217 
      5.818       0.866 
      0.944       0.039 
 
 ESC  10.0 
      2.828       1.026 
     15.770       6.222 
      0.487       0.157 
      6.763       1.409 
      2.484       0.466 
      0.769       0.101 
 
 ESC  2374.0 
     27.091      14.458 
    224.585      51.112 
      0.976       0.007 
    140.844      28.279 
      4.328       0.878 
      0.851       0.099 
 
 ESC  4738.0 
     34.643      19.898 
    286.232      72.230 
      0.985       0.005 
    185.238      38.568 
      4.368       0.871 
      0.851       0.098 
 
 ESC  7102.0 
     38.995      22.708 
    326.764      80.809 
      0.989       0.004 
    214.819      46.267 
      4.377       0.877 
      0.851       0.099 
 
 ESC  9466.0 
     42.062      24.955 
    354.514      86.252 
      0.991       0.003 
    237.303      53.104 
      4.385       0.874 
      0.851       0.099 
 
 ESC  11830.0 
     44.840      26.968 
    377.308      95.958 
      0.992       0.003 
    257.169      58.991 
      4.390       0.875 
      0.851       0.099 
 
 ESC  14194.0 
     46.903      28.066 
    402.847     100.890 
      0.993       0.002 
    273.450      63.206 
      4.393       0.874 
      0.851       0.099 
 
 ESC  16558.0 
     48.916      29.561 
    417.101     101.826 
      0.994       0.002 
    288.206      67.678 
      4.396       0.875 
      0.851       0.099 
 
 ESC  18922.0 
     50.694      30.564 
    433.768     105.660 
      0.995       0.002 
    301.681      72.331 
      4.398       0.875 
      0.851       0.099 
 
 ESC  21286.0 
     51.971      31.390 
    442.198     107.822 
      0.995       0.002 
    313.569      75.328 
      4.401       0.874 
      0.851       0.099 
 
 ESC  23650.0 
     53.365      32.251 
    456.259     110.676 
      0.996       0.001 
    324.981      78.820 
      4.402       0.874 
      0.851       0.099 
 
 Normal  10.0 
      2.287       0.401 
     18.738       6.756 
      0.413       0.144 
      7.238       1.421 
      2.593       0.507 
      0.781       0.119 
 
 Normal  2374.0 
     13.731       2.802 
    174.691      43.649 
      0.985       0.005 
    112.443      35.727 
      4.678       0.930 
      0.874       0.134 
 
 Normal  4738.0 
     15.974       3.153 
    217.406      54.661 
      0.990       0.003 
    139.681      42.208 
      4.700       0.931 
      0.874       0.133 
 
 Normal  7102.0 
     17.617       3.539 
    242.955      51.686 
      0.993       0.002 
    159.652      45.920 
      4.708       0.932 
      0.874       0.134 
 
 Normal  9466.0 
     18.837       3.702 
    259.409      57.929 
      0.994       0.002 
    175.310      47.991 
      4.714       0.932 
      0.874       0.134 
 
 Normal  11830.0 
     19.845       3.826 
    277.316      58.857 
      0.995       0.001 
    189.133      50.835 
      4.717       0.935 
      0.874       0.134 
 
 Normal  14194.0 
     20.749       3.987 
    285.429      60.784 
      0.995       0.001 
    200.500      52.376 
      4.722       0.933 
      0.874       0.134 
 
 Normal  16558.0 
     21.481       4.115 
    296.321      64.398 
      0.996       0.001 
    210.414      53.825 
      4.723       0.934 
      0.874       0.134 
 
 Normal  18922.0 
     22.211       4.289 
    305.311      68.090 
      0.996       0.001 
    219.624      55.554 
      4.724       0.933 
      0.874       0.134 
 
 Normal  21286.0 
     22.852       4.459 
    311.917      69.494 
      0.997       0.001 
    227.833      57.016 
      4.726       0.934 
      0.874       0.134 
 
 Normal  23650.0 
     23.403       4.565 
    317.062      71.397 
      0.997       0.001 
    235.181      57.955 
      4.727       0.934 
      0.874       0.134 
 
  SampleID  Seqs/Sample 
 PD_whole_tree Ave.  PD_whole_tree Err. 
 chao1 Ave.  chao1 Err. 
 goods_coverage Ave.  goods_coverage Err. 
 observed_species Ave.  observed_species Err. 
 shannon Ave.  shannon Err. 
 simpson Ave.  simpson Err. 
 
 
 ES1  10.0 
      2.181         nan 
     25.100         nan 
      0.350         nan 
      7.800         nan 
      2.791         nan 
      0.828         nan 
 
 ES1  2374.0 
     18.903         nan 
    279.788         nan 
      0.971         nan 
    217.100         nan 
      5.320         nan 
      0.908         nan 
 
 ES1  4738.0 
     21.256         nan 
    328.078         nan 
      0.986         nan 
    263.100         nan 
      5.369         nan 
      0.907         nan 
 
 ES1  7102.0 
     23.115         nan 
    352.137         nan 
      0.990         nan 
    294.100         nan 
      5.388         nan 
      0.907         nan 
 
 ES1  9466.0 
     24.081         nan 
    360.619         nan 
      0.993         nan 
    311.200         nan 
      5.381         nan 
      0.906         nan 
 
 ES1  11830.0 
     24.563         nan 
    365.151         nan 
      0.995         nan 
    323.800         nan 
      5.404         nan 
      0.907         nan 
 
 ES1  14194.0 
     24.979         nan 
    369.859         nan 
      0.996         nan 
    331.900         nan 
      5.406         nan 
      0.907         nan 
 
 ES1  16558.0 
     25.371         nan 
    371.225         nan 
      0.997         nan 
    339.400         nan 
      5.413         nan 
      0.907         nan 
 
 ES1  18922.0 
     25.909         nan 
    385.669         nan 
      0.997         nan 
    350.800         nan 
      5.412         nan 
      0.907         nan 
 
 ES1  21286.0 
     26.017         nan 
    381.544         nan 
      0.998         nan 
    353.800         nan 
      5.413         nan 
      0.907         nan 
 
 ES1  23650.0 
     26.201         nan 
    380.567         nan 
      0.998         nan 
    357.900         nan 
      5.419         nan 
      0.907         nan 
 
 ES2  10.0 
      2.785         nan 
     21.300         nan 
      0.320         nan 
      8.200         nan 
      2.927         nan 
      0.854         nan 
 
 ES2  2374.0 
     19.129         nan 
    285.866         nan 
      0.969         nan 
    192.700         nan 
      5.293         nan 
      0.927         nan 
 
 ES2  4738.0 
     21.840         nan 
    320.285         nan 
      0.984         nan 
    239.500         nan 
      5.346         nan 
      0.928         nan 
 
 ES2  7102.0 
     23.620         nan 
    357.206         nan 
      0.989         nan 
    269.000         nan 
      5.359         nan 
      0.927         nan 
 
 ES2  9466.0 
     25.049         nan 
    397.989         nan 
      0.991         nan 
    298.700         nan 
      5.358         nan 
      0.926         nan 
 
 ES2  11830.0 
     25.907         nan 
    421.066         nan 
      0.993         nan 
    311.100         nan 
      5.362         nan 
      0.927         nan 
 
 ES2  14194.0 
     26.792         nan 
    449.286         nan 
      0.994         nan 
    325.600         nan 
      5.377         nan 
      0.927         nan 
 
 ES2  16558.0 
     27.697         nan 
    463.144         nan 
      0.994         nan 
    342.800         nan 
      5.378         nan 
      0.927         nan 
 
 ES2  18922.0 
     28.433         nan 
    476.658         nan 
      0.995         nan 
    353.500         nan 
      5.375         nan 
      0.927         nan 
 
 ES2  21286.0 
     29.125         nan 
    481.702         nan 
      0.995         nan 
    367.500         nan 
      5.387         nan 
      0.927         nan 
 
 ES2  23650.0 
     29.595         nan 
    481.622         nan 
      0.996         nan 
    376.000         nan 
      5.384         nan 
      0.927         nan 
 
 ES3  10.0 
      2.592         nan 
     34.800         nan 
      0.140         nan 
      9.300         nan 
      3.182         nan 
      0.886         nan 
 
 ES3  2374.0 
     22.837         nan 
    343.199         nan 
      0.965         nan 
    255.200         nan 
      6.368         nan 
      0.974         nan 
 
 ES3  4738.0 
     26.294         nan 
    393.366         nan 
      0.983         nan 
    309.000         nan 
      6.418         nan 
      0.974         nan 
 
 ES3  7102.0 
     27.605         nan 
    415.503         nan 
      0.989         nan 
    334.400         nan 
      6.425         nan 
      0.974         nan 
 
 ES3  9466.0 
     29.301         nan 
    473.653         nan 
      0.991         nan 
    358.400         nan 
      6.447         nan 
      0.974         nan 
 
 ES3  11830.0 
     30.205         nan 
    469.935         nan 
      0.993         nan 
    374.600         nan 
      6.446         nan 
      0.974         nan 
 
 ES3  14194.0 
     31.552         nan 
    526.634         nan 
      0.993         nan 
    396.100         nan 
      6.454         nan 
      0.974         nan 
 
 ES3  16558.0 
     32.228         nan 
    557.810         nan 
      0.994         nan 
    410.000         nan 
      6.461         nan 
      0.974         nan 
 
 ES3  18922.0 
     33.076         nan 
    577.692         nan 
      0.995         nan 
    424.300         nan 
      6.468         nan 
      0.974         nan 
 
 ES3  21286.0 
     33.423         nan 
    614.296         nan 
      0.995         nan 
    434.400         nan 
      6.466         nan 
      0.974         nan 
 
 ES3  23650.0 
     34.175         nan 
    631.000         nan 
      0.995         nan 
    446.000         nan 
      6.466         nan 
      0.974         nan 
 
 ES4  10.0 
      2.391         nan 
     30.800         nan 
      0.220         nan 
      8.900         nan 
      3.102         nan 
      0.878         nan 
 
 ES4  2374.0 
     27.086         nan 
    382.677         nan 
      0.962         nan 
    303.600         nan 
      6.749         nan 
      0.975         nan 
 
 ES4  4738.0 
     31.440         nan 
    465.433         nan 
      0.980         nan 
    370.300         nan 
      6.807         nan 
      0.975         nan 
 
 ES4  7102.0 
     33.830         nan 
    491.867         nan 
      0.987         nan 
    405.300         nan 
      6.836         nan 
      0.976         nan 
 
 ES4  9466.0 
     35.329         nan 
    533.823         nan 
      0.990         nan 
    429.700         nan 
      6.840         nan 
      0.975         nan 
 
 ES4  11830.0 
     36.491         nan 
    555.269         nan 
      0.992         nan 
    449.200         nan 
      6.858         nan 
      0.976         nan 
 
 ES4  14194.0 
     37.894         nan 
    606.092         nan 
      0.993         nan 
    471.300         nan 
      6.862         nan 
      0.976         nan 
 
 ES4  16558.0 
     38.597         nan 
    592.490         nan 
      0.994         nan 
    483.200         nan 
      6.865         nan 
      0.976         nan 
 
 ES4  18922.0 
     39.552         nan 
    636.149         nan 
      0.994         nan 
    499.300         nan 
      6.871         nan 
      0.976         nan 
 
 ES4  21286.0 
     40.230         nan 
    647.168         nan 
      0.995         nan 
    509.100         nan 
      6.871         nan 
      0.976         nan 
 
 ES4  23650.0 
     40.808         nan 
    660.591         nan 
      0.995         nan 
    520.000         nan 
      6.874         nan 
      0.976         nan 
 
 ES5  10.0 
      2.504         nan 
     27.000         nan 
      0.230         nan 
      8.800         nan 
      3.074         nan 
      0.874         nan 
 
 ES5  2374.0 
     20.497         nan 
    305.563         nan 
      0.971         nan 
    236.100         nan 
      6.310         nan 
      0.973         nan 
 
 ES5  4738.0 
     24.462         nan 
    381.355         nan 
      0.984         nan 
    291.300         nan 
      6.355         nan 
      0.973         nan 
 
 ES5  7102.0 
     26.625         nan 
    423.989         nan 
      0.988         nan 
    325.900         nan 
      6.385         nan 
      0.974         nan 
 
 ES5  9466.0 
     27.622         nan 
    441.468         nan 
      0.991         nan 
    343.800         nan 
      6.393         nan 
      0.973         nan 
 
 ES5  11830.0 
     28.744         nan 
    479.259         nan 
      0.992         nan 
    363.900         nan 
      6.407         nan 
      0.974         nan 
 
 ES5  14194.0 
     29.864         nan 
    518.155         nan 
      0.993         nan 
    384.900         nan 
      6.408         nan 
      0.974         nan 
 
 ES5  16558.0 
     30.390         nan 
    521.027         nan 
      0.994         nan 
    397.200         nan 
      6.412         nan 
      0.974         nan 
 
 ES5  18922.0 
     30.876         nan 
    525.755         nan 
      0.995         nan 
    405.800         nan 
      6.408         nan 
      0.974         nan 
 
 ES5  21286.0 
     31.773         nan 
    550.102         nan 
      0.995         nan 
    420.800         nan 
      6.412         nan 
      0.973         nan 
 
 ES5  23650.0 
     32.303         nan 
    568.968         nan 
      0.996         nan 
    431.000         nan 
      6.417         nan 
      0.974         nan 
 
 ES6  10.0 
      2.642         nan 
     33.200         nan 
      0.150         nan 
      9.200         nan 
      3.154         nan 
      0.882         nan 
 
 ES6  2374.0 
     19.730         nan 
    271.313         nan 
      0.976         nan 
    204.300         nan 
      6.237         nan 
      0.973         nan 
 
 ES6  4738.0 
     22.966         nan 
    365.084         nan 
      0.985         nan 
    249.900         nan 
      6.297         nan 
      0.974         nan 
 
 ES6  7102.0 
     24.969         nan 
    399.654         nan 
      0.989         nan 
    281.800         nan 
      6.310         nan 
      0.974         nan 
 
 ES6  9466.0 
     26.426         nan 
    458.224         nan 
      0.990         nan 
    303.900         nan 
      6.318         nan 
      0.974         nan 
 
 ES6  11830.0 
     27.916         nan 
    502.040         nan 
      0.992         nan 
    325.800         nan 
      6.321         nan 
      0.974         nan 
 
 ES6  14194.0 
     29.540         nan 
    523.319         nan 
      0.992         nan 
    345.900         nan 
      6.332         nan 
      0.974         nan 
 
 ES6  16558.0 
     30.225         nan 
    528.441         nan 
      0.993         nan 
    359.300         nan 
      6.326         nan 
      0.974         nan 
 
 ES6  18922.0 
     31.215         nan 
    559.597         nan 
      0.994         nan 
    374.100         nan 
      6.328         nan 
      0.974         nan 
 
 ES6  21286.0 
     32.145         nan 
    576.246         nan 
      0.994         nan 
    388.200         nan 
      6.332         nan 
      0.974         nan 
 
 ES6  23650.0 
     33.065         nan 
    607.154         nan 
      0.995         nan 
    402.000         nan 
      6.335         nan 
      0.974         nan 
 
 ES7  10.0 
      2.794         nan 
     34.800         nan 
      0.140         nan 
      9.300         nan 
      3.182         nan 
      0.886         nan 
 
 ES7  2374.0 
     17.550         nan 
    223.162         nan 
      0.985         nan 
    176.100         nan 
      6.491         nan 
      0.981         nan 
 
 ES7  4738.0 
     20.373         nan 
    301.465         nan 
      0.989         nan 
    211.500         nan 
      6.526         nan 
      0.981         nan 
 
 ES7  7102.0 
     22.196         nan 
    358.949         nan 
      0.990         nan 
    241.500         nan 
      6.556         nan 
      0.982         nan 
 
 ES7  9466.0 
     23.493         nan 
    369.304         nan 
      0.992         nan 
    260.200         nan 
      6.555         nan 
      0.982         nan 
 
 ES7  11830.0 
     24.120         nan 
    378.422         nan 
      0.993         nan 
    271.800         nan 
      6.559         nan 
      0.982         nan 
 
 ES7  14194.0 
     25.051         nan 
    403.123         nan 
      0.994         nan 
    286.200         nan 
      6.554         nan 
      0.981         nan 
 
 ES7  16558.0 
     25.848         nan 
    411.657         nan 
      0.995         nan 
    300.900         nan 
      6.562         nan 
      0.981         nan 
 
 ES7  18922.0 
     26.529         nan 
    413.240         nan 
      0.995         nan 
    311.300         nan 
      6.564         nan 
      0.982         nan 
 
 ES7  21286.0 
     27.337         nan 
    423.599         nan 
      0.996         nan 
    323.000         nan 
      6.567         nan 
      0.982         nan 
 
 ES7  23650.0 
     27.857         nan 
    421.000         nan 
      0.996         nan 
    332.000         nan 
      6.569         nan 
      0.982         nan 
 
 ES8  10.0 
      2.775         nan 
     28.000         nan 
      0.210         nan 
      8.900         nan 
      3.094         nan 
      0.876         nan 
 
 ES8  2374.0 
     23.138         nan 
    331.040         nan 
      0.969         nan 
    252.300         nan 
      6.620         nan 
      0.979         nan 
 
 ES8  4738.0 
     27.906         nan 
    399.364         nan 
      0.983         nan 
    309.100         nan 
      6.671         nan 
      0.980         nan 
 
 ES8  7102.0 
     30.237         nan 
    446.106         nan 
      0.988         nan 
    337.200         nan 
      6.688         nan 
      0.980         nan 
 
 ES8  9466.0 
     32.638         nan 
    465.679         nan 
      0.991         nan 
    363.000         nan 
      6.707         nan 
      0.980         nan 
 
 ES8  11830.0 
     35.572         nan 
    501.496         nan 
      0.992         nan 
    380.300         nan 
      6.701         nan 
      0.979         nan 
 
 ES8  14194.0 
     36.683         nan 
    536.052         nan 
      0.993         nan 
    403.500         nan 
      6.715         nan 
      0.980         nan 
 
 ES8  16558.0 
     36.815         nan 
    564.930         nan 
      0.994         nan 
    415.800         nan 
      6.716         nan 
      0.979         nan 
 
 ES8  18922.0 
     38.365         nan 
    580.640         nan 
      0.995         nan 
    429.600         nan 
      6.721         nan 
      0.980         nan 
 
 ES8  21286.0 
     40.165         nan 
    620.015         nan 
      0.995         nan 
    443.000         nan 
      6.723         nan 
      0.980         nan 
 
 ES8  23650.0 
     42.065         nan 
    642.900         nan 
      0.995         nan 
    454.000         nan 
      6.724         nan 
      0.979         nan 
 
 ES9  10.0 
      2.688         nan 
     37.700         nan 
      0.190         nan 
      8.800         nan 
      3.039         nan 
      0.864         nan 
 
 ES9  2374.0 
     23.169         nan 
    327.682         nan 
      0.970         nan 
    268.100         nan 
      6.314         nan 
      0.954         nan 
 
 ES9  4738.0 
     25.927         nan 
    366.740         nan 
      0.987         nan 
    310.900         nan 
      6.363         nan 
      0.954         nan 
 
 ES9  7102.0 
     27.770         nan 
    407.061         nan 
      0.991         nan 
    336.400         nan 
      6.411         nan 
      0.955         nan 
 
 ES9  9466.0 
     28.860         nan 
    417.713         nan 
      0.993         nan 
    354.600         nan 
      6.408         nan 
      0.955         nan 
 
 ES9  11830.0 
     30.742         nan 
    441.886         nan 
      0.994         nan 
    373.100         nan 
      6.431         nan 
      0.955         nan 
 
 ES9  14194.0 
     31.013         nan 
    447.887         nan 
      0.995         nan 
    381.800         nan 
      6.417         nan 
      0.955         nan 
 
 ES9  16558.0 
     32.017         nan 
    451.375         nan 
      0.996         nan 
    391.600         nan 
      6.423         nan 
      0.954         nan 
 
 ES9  18922.0 
     32.659         nan 
    458.502         nan 
      0.997         nan 
    400.900         nan 
      6.429         nan 
      0.955         nan 
 
 ES9  21286.0 
     33.073         nan 
    468.989         nan 
      0.997         nan 
    407.900         nan 
      6.429         nan 
      0.955         nan 
 
 ES9  23650.0 
     33.721         nan 
    478.030         nan 
      0.997         nan 
    415.000         nan 
      6.433         nan 
      0.955         nan 
 
 ES10  10.0 
      2.659         nan 
     23.200         nan 
      0.310         nan 
      8.000         nan 
      2.831         nan 
      0.832         nan 
 
 ES10  2374.0 
     20.586         nan 
    259.759         nan 
      0.976         nan 
    202.300         nan 
      5.475         nan 
      0.916         nan 
 
 ES10  4738.0 
     23.799         nan 
    333.049         nan 
      0.986         nan 
    246.900         nan 
      5.508         nan 
      0.916         nan 
 
 ES10  7102.0 
     25.674         nan 
    392.654         nan 
      0.989         nan 
    276.500         nan 
      5.519         nan 
      0.915         nan 
 
 ES10  9466.0 
     27.179         nan 
    398.373         nan 
      0.992         nan 
    296.800         nan 
      5.517         nan 
      0.914         nan 
 
 ES10  11830.0 
     27.943         nan 
    424.213         nan 
      0.993         nan 
    315.400         nan 
      5.534         nan 
      0.915         nan 
 
 ES10  14194.0 
     28.706         nan 
    424.207         nan 
      0.994         nan 
    329.100         nan 
      5.535         nan 
      0.914         nan 
 
 ES10  16558.0 
     30.281         nan 
    480.918         nan 
      0.994         nan 
    348.000         nan 
      5.537         nan 
      0.914         nan 
 
 ES10  18922.0 
     30.860         nan 
    510.300         nan 
      0.995         nan 
    360.000         nan 
      5.535         nan 
      0.914         nan 
 
 ES10  21286.0 
     31.908         nan 
    518.639         nan 
      0.995         nan 
    372.900         nan 
      5.541         nan 
      0.914         nan 
 
 ES10  23650.0 
     32.529         nan 
    545.676         nan 
      0.996         nan 
    382.000         nan 
      5.540         nan 
      0.914         nan 
 
 ES11  10.0 
      2.516         nan 
     22.150         nan 
      0.350         nan 
      8.000         nan 
      2.879         nan 
      0.848         nan 
 
 ES11  2374.0 
     18.561         nan 
    245.686         nan 
      0.975         nan 
    184.700         nan 
      5.436         nan 
      0.935         nan 
 
 ES11  4738.0 
     21.272         nan 
    295.798         nan 
      0.987         nan 
    226.400         nan 
      5.473         nan 
      0.935         nan 
 
 ES11  7102.0 
     22.865         nan 
    303.431         nan 
      0.992         nan 
    248.500         nan 
      5.490         nan 
      0.935         nan 
 
 ES11  9466.0 
     23.955         nan 
    340.773         nan 
      0.994         nan 
    267.800         nan 
      5.505         nan 
      0.936         nan 
 
 ES11  11830.0 
     24.696         nan 
    350.074         nan 
      0.995         nan 
    278.700         nan 
      5.514         nan 
      0.935         nan 
 
 ES11  14194.0 
     25.547         nan 
    367.282         nan 
      0.996         nan 
    291.000         nan 
      5.506         nan 
      0.935         nan 
 
 ES11  16558.0 
     26.363         nan 
    385.471         nan 
      0.996         nan 
    301.900         nan 
      5.518         nan 
      0.935         nan 
 
 ES11  18922.0 
     26.719         nan 
    400.559         nan 
      0.996         nan 
    308.400         nan 
      5.514         nan 
      0.935         nan 
 
 ES11  21286.0 
     27.398         nan 
    408.227         nan 
      0.997         nan 
    317.700         nan 
      5.518         nan 
      0.935         nan 
 
 ES11  23650.0 
     27.960         nan 
    416.286         nan 
      0.997         nan 
    325.000         nan 
      5.520         nan 
      0.935         nan 
 
 ES12  10.0 
      2.065         nan 
     11.120         nan 
      0.570         nan 
      6.800         nan 
      2.619         nan 
      0.818         nan 
 
 ES12  2374.0 
      5.786         nan 
     38.800         nan 
      0.998         nan 
     30.200         nan 
      3.654         nan 
      0.899         nan 
 
 ES12  4738.0 
      6.060         nan 
     44.408         nan 
      0.998         nan 
     33.800         nan 
      3.646         nan 
      0.898         nan 
 
 ES12  7102.0 
      6.376         nan 
     49.508         nan 
      0.999         nan 
     37.400         nan 
      3.648         nan 
      0.898         nan 
 
 ES12  9466.0 
      6.610         nan 
     57.598         nan 
      0.999         nan 
     39.200         nan 
      3.651         nan 
      0.898         nan 
 
 ES12  11830.0 
      6.701         nan 
     49.962         nan 
      0.999         nan 
     41.100         nan 
      3.650         nan 
      0.898         nan 
 
 ES12  14194.0 
      6.923         nan 
     59.079         nan 
      0.999         nan 
     44.000         nan 
      3.655         nan 
      0.898         nan 
 
 ES12  16558.0 
      6.935         nan 
     53.589         nan 
      0.999         nan 
     43.900         nan 
      3.651         nan 
      0.898         nan 
 
 ES12  18922.0 
      7.155         nan 
     59.593         nan 
      0.999         nan 
     46.800         nan 
      3.653         nan 
      0.899         nan 
 
 ES12  21286.0 
      7.180         nan 
     57.653         nan 
      0.999         nan 
     47.500         nan 
      3.656         nan 
      0.899         nan 
 
 ES12  23650.0 
      7.280         nan 
     62.200         nan 
      0.999         nan 
     49.000         nan 
      3.654         nan 
      0.898         nan 
 
 ES13  10.0 
      2.321         nan 
     16.850         nan 
      0.490         nan 
      6.500         nan 
      2.397         nan 
      0.758         nan 
 
 ES13  2374.0 
     18.392         nan 
    191.599         nan 
      0.983         nan 
    140.300         nan 
      4.328         nan 
      0.839         nan 
 
 ES13  4738.0 
     21.592         nan 
    262.612         nan 
      0.988         nan 
    176.700         nan 
      4.361         nan 
      0.839         nan 
 
 ES13  7102.0 
     22.984         nan 
    282.180         nan 
      0.992         nan 
    198.400         nan 
      4.375         nan 
      0.839         nan 
 
 ES13  9466.0 
     24.008         nan 
    316.644         nan 
      0.993         nan 
    215.900         nan 
      4.389         nan 
      0.840         nan 
 
 ES13  11830.0 
     25.352         nan 
    347.781         nan 
      0.994         nan 
    234.200         nan 
      4.401         nan 
      0.841         nan 
 
 ES13  14194.0 
     26.317         nan 
    350.114         nan 
      0.994         nan 
    250.700         nan 
      4.403         nan 
      0.841         nan 
 
 ES13  16558.0 
     26.880         nan 
    365.069         nan 
      0.995         nan 
    260.000         nan 
      4.399         nan 
      0.840         nan 
 
 ES13  18922.0 
     27.667         nan 
    373.629         nan 
      0.995         nan 
    271.400         nan 
      4.399         nan 
      0.840         nan 
 
 ES13  21286.0 
     28.141         nan 
    383.281         nan 
      0.996         nan 
    281.400         nan 
      4.396         nan 
      0.839         nan 
 
 ES13  23650.0 
     28.692         nan 
    387.007         nan 
      0.996         nan 
    290.800         nan 
      4.401         nan 
      0.840         nan 
 
 ES14  10.0 
      2.834         nan 
     26.400         nan 
      0.220         nan 
      8.900         nan 
      3.102         nan 
      0.878         nan 
 
 ES14  2374.0 
     19.529         nan 
    213.030         nan 
      0.985         nan 
    179.800         nan 
      5.978         nan 
      0.967         nan 
 
 ES14  4738.0 
     21.088         nan 
    240.812         nan 
      0.993         nan 
    201.800         nan 
      5.980         nan 
      0.966         nan 
 
 ES14  7102.0 
     21.785         nan 
    256.453         nan 
      0.995         nan 
    214.300         nan 
      5.994         nan 
      0.966         nan 
 
 ES14  9466.0 
     22.316         nan 
    289.237         nan 
      0.996         nan 
    225.400         nan 
      5.987         nan 
      0.966         nan 
 
 ES14  11830.0 
     23.250         nan 
    299.963         nan 
      0.996         nan 
    239.000         nan 
      6.005         nan 
      0.966         nan 
 
 ES14  14194.0 
     23.711         nan 
    314.121         nan 
      0.997         nan 
    245.400         nan 
      6.003         nan 
      0.966         nan 
 
 ES14  16558.0 
     24.026         nan 
    318.519         nan 
      0.997         nan 
    252.600         nan 
      6.010         nan 
      0.966         nan 
 
 ES14  18922.0 
     24.375         nan 
    334.916         nan 
      0.997         nan 
    259.100         nan 
      6.013         nan 
      0.966         nan 
 
 ES14  21286.0 
     24.840         nan 
    337.876         nan 
      0.997         nan 
    266.500         nan 
      6.011         nan 
      0.966         nan 
 
 ES14  23650.0 
     25.023         nan 
    361.588         nan 
      0.998         nan 
    271.000         nan 
      6.012         nan 
      0.966         nan 
 
 ES15  10.0 
      2.363         nan 
     22.700         nan 
      0.350         nan 
      7.900         nan 
      2.836         nan 
      0.838         nan 
 
 ES15  2374.0 
     14.053         nan 
    157.598         nan 
      0.989         nan 
    100.800         nan 
      5.497         nan 
      0.965         nan 
 
 ES15  4738.0 
     15.876         nan 
    172.589         nan 
      0.993         nan 
    120.400         nan 
      5.502         nan 
      0.965         nan 
 
 ES15  7102.0 
     16.727         nan 
    202.343         nan 
      0.994         nan 
    132.900         nan 
      5.511         nan 
      0.965         nan 
 
 ES15  9466.0 
     17.846         nan 
    245.851         nan 
      0.995         nan 
    145.100         nan 
      5.517         nan 
      0.965         nan 
 
 ES15  11830.0 
     18.564         nan 
    232.652         nan 
      0.996         nan 
    154.300         nan 
      5.515         nan 
      0.965         nan 
 
 ES15  14194.0 
     19.061         nan 
    247.990         nan 
      0.996         nan 
    164.300         nan 
      5.518         nan 
      0.965         nan 
 
 ES15  16558.0 
     19.964         nan 
    286.926         nan 
      0.996         nan 
    175.200         nan 
      5.518         nan 
      0.965         nan 
 
 ES15  18922.0 
     20.534         nan 
    279.444         nan 
      0.997         nan 
    183.500         nan 
      5.523         nan 
      0.965         nan 
 
 ES15  21286.0 
     20.926         nan 
    283.776         nan 
      0.997         nan 
    190.500         nan 
      5.524         nan 
      0.965         nan 
 
 ES15  23650.0 
     21.424         nan 
    282.647         nan 
      0.997         nan 
    197.000         nan 
      5.523         nan 
      0.965         nan 
 
 ESC1  10.0 
      2.617         nan 
     29.300         nan 
      0.250         nan 
      8.700         nan 
      3.054         nan 
      0.872         nan 
 
 ESC1  2374.0 
     13.989         nan 
    158.859         nan 
      0.990         nan 
    123.500         nan 
      5.755         nan 
      0.969         nan 
 
 ESC1  4738.0 
     15.319         nan 
    181.955         nan 
      0.994         nan 
    142.100         nan 
      5.755         nan 
      0.968         nan 
 
 ESC1  7102.0 
     16.488         nan 
    215.153         nan 
      0.995         nan 
    156.600         nan 
      5.775         nan 
      0.968         nan 
 
 ESC1  9466.0 
     17.532         nan 
    223.629         nan 
      0.996         nan 
    164.000         nan 
      5.767         nan 
      0.968         nan 
 
 ESC1  11830.0 
     18.017         nan 
    256.390         nan 
      0.996         nan 
    173.500         nan 
      5.770         nan 
      0.968         nan 
 
 ESC1  14194.0 
     19.107         nan 
    271.520         nan 
      0.996         nan 
    184.800         nan 
      5.777         nan 
      0.969         nan 
 
 ESC1  16558.0 
     19.539         nan 
    297.321         nan 
      0.997         nan 
    195.000         nan 
      5.778         nan 
      0.968         nan 
 
 ESC1  18922.0 
     20.235         nan 
    299.979         nan 
      0.997         nan 
    199.200         nan 
      5.784         nan 
      0.969         nan 
 
 ESC1  21286.0 
     20.903         nan 
    303.248         nan 
      0.997         nan 
    208.100         nan 
      5.784         nan 
      0.968         nan 
 
 ESC1  23650.0 
     21.182         nan 
    304.500         nan 
      0.997         nan 
    213.000         nan 
      5.784         nan 
      0.968         nan 
 
 ESC2  10.0 
      2.910         nan 
     10.400         nan 
      0.630         nan 
      4.800         nan 
      1.684         nan 
      0.564         nan 
 
 ESC2  2374.0 
     56.887         nan 
    211.733         nan 
      0.977         nan 
    142.900         nan 
      3.456         nan 
      0.690         nan 
 
 ESC2  4738.0 
     79.717         nan 
    259.896         nan 
      0.986         nan 
    190.800         nan 
      3.495         nan 
      0.692         nan 
 
 ESC2  7102.0 
     87.935         nan 
    314.851         nan 
      0.990         nan 
    217.000         nan 
      3.524         nan 
      0.695         nan 
 
 ESC2  9466.0 
     94.479         nan 
    337.826         nan 
      0.992         nan 
    237.200         nan 
      3.515         nan 
      0.693         nan 
 
 ESC2  11830.0 
    104.082         nan 
    367.316         nan 
      0.993         nan 
    259.700         nan 
      3.547         nan 
      0.696         nan 
 
 ESC2  14194.0 
    107.536         nan 
    413.384         nan 
      0.994         nan 
    272.100         nan 
      3.546         nan 
      0.696         nan 
 
 ESC2  16558.0 
    113.340         nan 
    407.628         nan 
      0.995         nan 
    285.700         nan 
      3.544         nan 
      0.696         nan 
 
 ESC2  18922.0 
    116.282         nan 
    430.252         nan 
      0.995         nan 
    299.200         nan 
      3.540         nan 
      0.694         nan 
 
 ESC2  21286.0 
    120.694         nan 
    432.289         nan 
      0.995         nan 
    311.200         nan 
      3.541         nan 
      0.694         nan 
 
 ESC2  23650.0 
    122.611         nan 
    448.833         nan 
      0.996         nan 
    322.000         nan 
      3.545         nan 
      0.695         nan 
 
 ESC3  10.0 
      1.981         nan 
      9.133         nan 
      0.600         nan 
      6.300         nan 
      2.443         nan 
      0.784         nan 
 
 ESC3  2374.0 
     34.257         nan 
    173.768         nan 
      0.981         nan 
    106.600         nan 
      3.793         nan 
      0.847         nan 
 
 ESC3  4738.0 
     42.490         nan 
    242.275         nan 
      0.987         nan 
    146.000         nan 
      3.816         nan 
      0.847         nan 
 
 ESC3  7102.0 
     48.275         nan 
    268.609         nan 
      0.990         nan 
    167.500         nan 
      3.800         nan 
      0.845         nan 
 
 ESC3  9466.0 
     52.906         nan 
    290.935         nan 
      0.992         nan 
    189.100         nan 
      3.810         nan 
      0.844         nan 
 
 ESC3  11830.0 
     57.309         nan 
    322.657         nan 
      0.993         nan 
    208.700         nan 
      3.819         nan 
      0.845         nan 
 
 ESC3  14194.0 
     60.455         nan 
    334.180         nan 
      0.994         nan 
    224.200         nan 
      3.817         nan 
      0.844         nan 
 
 ESC3  16558.0 
     61.900         nan 
    356.018         nan 
      0.995         nan 
    234.400         nan 
      3.826         nan 
      0.845         nan 
 
 ESC3  18922.0 
     63.496         nan 
    369.612         nan 
      0.995         nan 
    247.300         nan 
      3.825         nan 
      0.845         nan 
 
 ESC3  21286.0 
     65.775         nan 
    379.997         nan 
      0.996         nan 
    259.700         nan 
      3.825         nan 
      0.844         nan 
 
 ESC3  23650.0 
     67.832         nan 
    388.385         nan 
      0.996         nan 
    269.000         nan 
      3.830         nan 
      0.845         nan 
 
 ESC4  10.0 
      2.045         nan 
     14.850         nan 
      0.540         nan 
      5.800         nan 
      2.098         nan 
      0.680         nan 
 
 ESC4  2374.0 
     14.303         nan 
    153.642         nan 
      0.988         nan 
    105.400         nan 
      4.026         nan 
      0.767         nan 
 
 ESC4  4738.0 
     16.308         nan 
    213.970         nan 
      0.991         nan 
    131.900         nan 
      4.074         nan 
      0.773         nan 
 
 ESC4  7102.0 
     17.528         nan 
    251.475         nan 
      0.993         nan 
    148.900         nan 
      4.071         nan 
      0.770         nan 
 
 ESC4  9466.0 
     18.244         nan 
    264.811         nan 
      0.994         nan 
    164.000         nan 
      4.060         nan 
      0.768         nan 
 
 ESC4  11830.0 
     18.825         nan 
    261.943         nan 
      0.995         nan 
    175.200         nan 
      4.081         nan 
      0.770         nan 
 
 ESC4  14194.0 
     19.569         nan 
    290.287         nan 
      0.995         nan 
    189.000         nan 
      4.089         nan 
      0.771         nan 
 
 ESC4  16558.0 
     19.991         nan 
    293.369         nan 
      0.996         nan 
    198.100         nan 
      4.087         nan 
      0.770         nan 
 
 ESC4  18922.0 
     20.520         nan 
    291.613         nan 
      0.996         nan 
    208.200         nan 
      4.087         nan 
      0.770         nan 
 
 ESC4  21286.0 
     20.865         nan 
    304.914         nan 
      0.997         nan 
    214.900         nan 
      4.086         nan 
      0.770         nan 
 
 ESC4  23650.0 
     21.245         nan 
    304.966         nan 
      0.997         nan 
    222.000         nan 
      4.090         nan 
      0.770         nan 
 
 ESC5  10.0 
      1.827         nan 
      7.400         nan 
      0.700         nan 
      4.300         nan 
      1.532         nan 
      0.534         nan 
 
 ESC5  2374.0 
     11.601         nan 
    130.460         nan 
      0.986         nan 
     89.800         nan 
      2.798         nan 
      0.608         nan 
 
 ESC5  4738.0 
     14.530         nan 
    175.616         nan 
      0.991         nan 
    122.600         nan 
      2.857         nan 
      0.614         nan 
 
 ESC5  7102.0 
     16.481         nan 
    215.568         nan 
      0.993         nan 
    143.700         nan 
      2.859         nan 
      0.612         nan 
 
 ESC5  9466.0 
     16.935         nan 
    228.483         nan 
      0.994         nan 
    155.600         nan 
      2.864         nan 
      0.612         nan 
 
 ESC5  11830.0 
     18.479         nan 
    269.951         nan 
      0.995         nan 
    173.400         nan 
      2.857         nan 
      0.610         nan 
 
 ESC5  14194.0 
     19.102         nan 
    269.593         nan 
      0.996         nan 
    181.100         nan 
      2.860         nan 
      0.611         nan 
 
 ESC5  16558.0 
     20.030         nan 
    301.349         nan 
      0.996         nan 
    192.100         nan 
      2.867         nan 
      0.612         nan 
 
 ESC5  18922.0 
     20.910         nan 
    307.479         nan 
      0.996         nan 
    200.000         nan 
      2.876         nan 
      0.613         nan 
 
 ESC5  21286.0 
     21.548         nan 
    326.191         nan 
      0.997         nan 
    209.900         nan 
      2.875         nan 
      0.613         nan 
 
 ESC5  23650.0 
     22.082         nan 
    355.521         nan 
      0.997         nan 
    218.000         nan 
      2.875         nan 
      0.612         nan 
 
 ESC6  10.0 
      3.059         nan 
     11.250         nan 
      0.590         nan 
      5.600         nan 
      2.082         nan 
      0.688         nan 
 
 ESC6  2374.0 
     35.690         nan 
    236.425         nan 
      0.973         nan 
    163.600         nan 
      3.739         nan 
      0.755         nan 
 
 ESC6  4738.0 
     47.346         nan 
    297.565         nan 
      0.985         nan 
    215.900         nan 
      3.797         nan 
      0.756         nan 
 
 ESC6  7102.0 
     53.042         nan 
    309.896         nan 
      0.990         nan 
    236.700         nan 
      3.788         nan 
      0.755         nan 
 
 ESC6  9466.0 
     56.520         nan 
    363.632         nan 
      0.992         nan 
    262.400         nan 
      3.823         nan 
      0.757         nan 
 
 ESC6  11830.0 
     59.612         nan 
    374.991         nan 
      0.993         nan 
    274.800         nan 
      3.816         nan 
      0.756         nan 
 
 ESC6  14194.0 
     63.291         nan 
    423.764         nan 
      0.994         nan 
    292.100         nan 
      3.814         nan 
      0.755         nan 
 
 ESC6  16558.0 
     63.850         nan 
    438.482         nan 
      0.995         nan 
    306.300         nan 
      3.817         nan 
      0.755         nan 
 
 ESC6  18922.0 
     65.570         nan 
    442.640         nan 
      0.995         nan 
    315.600         nan 
      3.819         nan 
      0.755         nan 
 
 ESC6  21286.0 
     66.923         nan 
    463.011         nan 
      0.996         nan 
    327.900         nan 
      3.829         nan 
      0.756         nan 
 
 ESC6  23650.0 
     67.998         nan 
    472.423         nan 
      0.996         nan 
    336.900         nan 
      3.827         nan 
      0.756         nan 
 
 ESC7  10.0 
      3.407         nan 
     20.300         nan 
      0.320         nan 
      8.100         nan 
      2.892         nan 
      0.848         nan 
 
 ESC7  2374.0 
     46.173         nan 
    239.578         nan 
      0.975         nan 
    158.500         nan 
      5.031         nan 
      0.929         nan 
 
 ESC7  4738.0 
     60.384         nan 
    254.678         nan 
      0.988         nan 
    199.700         nan 
      5.075         nan 
      0.929         nan 
 
 ESC7  7102.0 
     72.154         nan 
    309.803         nan 
      0.990         nan 
    231.200         nan 
      5.088         nan 
      0.929         nan 
 
 ESC7  9466.0 
     77.375         nan 
    329.135         nan 
      0.993         nan 
    248.500         nan 
      5.104         nan 
      0.929         nan 
 
 ESC7  11830.0 
     80.954         nan 
    337.273         nan 
      0.994         nan 
    262.500         nan 
      5.104         nan 
      0.929         nan 
 
 ESC7  14194.0 
     83.034         nan 
    346.411         nan 
      0.995         nan 
    273.200         nan 
      5.104         nan 
      0.929         nan 
 
 ESC7  16558.0 
     86.530         nan 
    373.701         nan 
      0.996         nan 
    284.600         nan 
      5.105         nan 
      0.929         nan 
 
 ESC7  18922.0 
     88.910         nan 
    387.000         nan 
      0.996         nan 
    295.800         nan 
      5.112         nan 
      0.929         nan 
 
 ESC7  21286.0 
     91.378         nan 
    395.740         nan 
      0.996         nan 
    304.000         nan 
      5.110         nan 
      0.929         nan 
 
 ESC7  23650.0 
     93.823         nan 
    403.732         nan 
      0.997         nan 
    313.000         nan 
      5.114         nan 
      0.929         nan 
 
 ESC8  10.0 
      2.609         nan 
     17.250         nan 
      0.470         nan 
      6.700         nan 
      2.440         nan 
      0.758         nan 
 
 ESC8  2374.0 
     17.133         nan 
    192.503         nan 
      0.982         nan 
    153.000         nan 
      4.728         nan 
      0.874         nan 
 
 ESC8  4738.0 
     19.278         nan 
    246.543         nan 
      0.989         nan 
    184.500         nan 
      4.739         nan 
      0.872         nan 
 
 ESC8  7102.0 
     21.381         nan 
    302.812         nan 
      0.992         nan 
    207.900         nan 
      4.750         nan 
      0.872         nan 
 
 ESC8  9466.0 
     22.324         nan 
    296.183         nan 
      0.994         nan 
    223.300         nan 
      4.756         nan 
      0.872         nan 
 
 ESC8  11830.0 
     23.109         nan 
    312.216         nan 
      0.995         nan 
    239.000         nan 
      4.763         nan 
      0.872         nan 
 
 ESC8  14194.0 
     24.088         nan 
    353.155         nan 
      0.995         nan 
    251.800         nan 
      4.761         nan 
      0.872         nan 
 
 ESC8  16558.0 
     24.487         nan 
    345.664         nan 
      0.996         nan 
    261.300         nan 
      4.766         nan 
      0.872         nan 
 
 ESC8  18922.0 
     25.021         nan 
    362.245         nan 
      0.996         nan 
    271.900         nan 
      4.767         nan 
      0.872         nan 
 
 ESC8  21286.0 
     25.474         nan 
    371.961         nan 
      0.996         nan 
    280.100         nan 
      4.771         nan 
      0.872         nan 
 
 ESC8  23650.0 
     25.949         nan 
    376.667         nan 
      0.997         nan 
    288.000         nan 
      4.770         nan 
      0.872         nan 
 
 ESC9  10.0 
      2.626         nan 
     19.950         nan 
      0.390         nan 
      7.600         nan 
      2.742         nan 
      0.820         nan 
 
 ESC9  2374.0 
     17.479         nan 
    228.607         nan 
      0.978         nan 
    162.600         nan 
      4.809         nan 
      0.890         nan 
 
 ESC9  4738.0 
     19.725         nan 
    267.865         nan 
      0.988         nan 
    197.200         nan 
      4.854         nan 
      0.891         nan 
 
 ESC9  7102.0 
     20.917         nan 
    304.692         nan 
      0.991         nan 
    219.800         nan 
      4.871         nan 
      0.893         nan 
 
 ESC9  9466.0 
     22.214         nan 
    314.684         nan 
      0.993         nan 
    236.400         nan 
      4.876         nan 
      0.893         nan 
 
 ESC9  11830.0 
     23.208         nan 
    336.633         nan 
      0.994         nan 
    252.600         nan 
      4.886         nan 
      0.892         nan 
 
 ESC9  14194.0 
     23.904         nan 
    353.613         nan 
      0.995         nan 
    265.600         nan 
      4.883         nan 
      0.892         nan 
 
 ESC9  16558.0 
     24.320         nan 
    381.848         nan 
      0.995         nan 
    276.100         nan 
      4.889         nan 
      0.892         nan 
 
 ESC9  18922.0 
     24.958         nan 
    388.154         nan 
      0.996         nan 
    286.300         nan 
      4.890         nan 
      0.893         nan 
 
 ESC9  21286.0 
     25.500         nan 
    379.349         nan 
      0.996         nan 
    294.100         nan 
      4.892         nan 
      0.892         nan 
 
 ESC9  23650.0 
     26.016         nan 
    405.250         nan 
      0.997         nan 
    304.000         nan 
      4.892         nan 
      0.892         nan 
 
 ESC10  10.0 
      2.366         nan 
     11.450         nan 
      0.570         nan 
      6.200         nan 
      2.357         nan 
      0.758         nan 
 
 ESC10  2374.0 
     15.034         nan 
    192.836         nan 
      0.979         nan 
    107.000         nan 
      3.451         nan 
      0.812         nan 
 
 ESC10  4738.0 
     22.475         nan 
    243.963         nan 
      0.987         nan 
    141.500         nan 
      3.469         nan 
      0.812         nan 
 
 ESC10  7102.0 
     25.523         nan 
    288.843         nan 
      0.989         nan 
    172.500         nan 
      3.477         nan 
      0.810         nan 
 
 ESC10  9466.0 
     28.565         nan 
    341.723         nan 
      0.991         nan 
    195.800         nan 
      3.488         nan 
      0.812         nan 
 
 ESC10  11830.0 
     29.319         nan 
    358.396         nan 
      0.992         nan 
    214.300         nan 
      3.480         nan 
      0.810         nan 
 
 ESC10  14194.0 
     31.577         nan 
    393.566         nan 
      0.993         nan 
    235.300         nan 
      3.486         nan 
      0.810         nan 
 
 ESC10  16558.0 
     33.239         nan 
    388.110         nan 
      0.994         nan 
    249.100         nan 
      3.488         nan 
      0.811         nan 
 
 ESC10  18922.0 
     34.048         nan 
    406.542         nan 
      0.994         nan 
    264.000         nan 
      3.492         nan 
      0.810         nan 
 
 ESC10  21286.0 
     34.761         nan 
    413.741         nan 
      0.995         nan 
    274.900         nan 
      3.490         nan 
      0.810         nan 
 
 ESC10  23650.0 
     35.778         nan 
    436.867         nan 
      0.995         nan 
    288.900         nan 
      3.495         nan 
      0.810         nan 
 
 ESC11  10.0 
      2.500         nan 
     15.150         nan 
      0.500         nan 
      6.800         nan 
      2.528         nan 
      0.786         nan 
 
 ESC11  2374.0 
     18.524         nan 
    193.857         nan 
      0.980         nan 
    110.100         nan 
      4.721         nan 
      0.931         nan 
 
 ESC11  4738.0 
     25.357         nan 
    268.883         nan 
      0.986         nan 
    145.500         nan 
      4.739         nan 
      0.931         nan 
 
 ESC11  7102.0 
     28.793         nan 
    292.906         nan 
      0.989         nan 
    173.800         nan 
      4.745         nan 
      0.930         nan 
 
 ESC11  9466.0 
     29.907         nan 
    331.898         nan 
      0.991         nan 
    193.400         nan 
      4.746         nan 
      0.931         nan 
 
 ESC11  11830.0 
     34.904         nan 
    366.089         nan 
      0.992         nan 
    219.000         nan 
      4.757         nan 
      0.931         nan 
 
 ESC11  14194.0 
     36.439         nan 
    369.080         nan 
      0.993         nan 
    231.900         nan 
      4.756         nan 
      0.931         nan 
 
 ESC11  16558.0 
     39.278         nan 
    390.727         nan 
      0.994         nan 
    249.200         nan 
      4.762         nan 
      0.931         nan 
 
 ESC11  18922.0 
     41.342         nan 
    404.950         nan 
      0.994         nan 
    261.500         nan 
      4.762         nan 
      0.931         nan 
 
 ESC11  21286.0 
     43.294         nan 
    410.627         nan 
      0.995         nan 
    275.400         nan 
      4.765         nan 
      0.931         nan 
 
 ESC11  23650.0 
     44.326         nan 
    416.227         nan 
      0.995         nan 
    287.800         nan 
      4.766         nan 
      0.931         nan 
 
 ESC12  10.0 
      2.620         nan 
     18.233         nan 
      0.390         nan 
      7.700         nan 
      2.799         nan 
      0.836         nan 
 
 ESC12  2374.0 
     14.823         nan 
    207.811         nan 
      0.986         nan 
    124.100         nan 
      5.292         nan 
      0.937         nan 
 
 ESC12  4738.0 
     16.485         nan 
    224.462         nan 
      0.991         nan 
    147.100         nan 
      5.293         nan 
      0.936         nan 
 
 ESC12  7102.0 
     17.851         nan 
    269.952         nan 
      0.993         nan 
    166.400         nan 
      5.320         nan 
      0.937         nan 
 
 ESC12  9466.0 
     18.956         nan 
    311.477         nan 
      0.993         nan 
    187.500         nan 
      5.323         nan 
      0.936         nan 
 
 ESC12  11830.0 
     19.358         nan 
    306.148         nan 
      0.994         nan 
    198.200         nan 
      5.329         nan 
      0.937         nan 
 
 ESC12  14194.0 
     20.325         nan 
    324.337         nan 
      0.995         nan 
    212.400         nan 
      5.323         nan 
      0.936         nan 
 
 ESC12  16558.0 
     20.904         nan 
    318.627         nan 
      0.996         nan 
    224.000         nan 
      5.331         nan 
      0.936         nan 
 
 ESC12  18922.0 
     21.532         nan 
    334.002         nan 
      0.996         nan 
    234.800         nan 
      5.334         nan 
      0.937         nan 
 
 ESC12  21286.0 
     22.041         nan 
    334.475         nan 
      0.996         nan 
    244.200         nan 
      5.335         nan 
      0.936         nan 
 
 ESC12  23650.0 
     22.425         nan 
    339.568         nan 
      0.997         nan 
    252.000         nan 
      5.335         nan 
      0.936         nan 
 
 ESC13  10.0 
      2.461         nan 
     19.050         nan 
      0.320         nan 
      8.300         nan 
      2.967         nan 
      0.862         nan 
 
 ESC13  2374.0 
     15.182         nan 
    194.241         nan 
      0.981         nan 
    125.700         nan 
      4.919         nan 
      0.939         nan 
 
 ESC13  4738.0 
     18.080         nan 
    257.577         nan 
      0.987         nan 
    162.800         nan 
      4.929         nan 
      0.938         nan 
 
 ESC13  7102.0 
     19.518         nan 
    276.684         nan 
      0.991         nan 
    185.000         nan 
      4.933         nan 
      0.937         nan 
 
 ESC13  9466.0 
     21.035         nan 
    304.493         nan 
      0.993         nan 
    202.200         nan 
      4.931         nan 
      0.938         nan 
 
 ESC13  11830.0 
     22.078         nan 
    327.136         nan 
      0.994         nan 
    216.900         nan 
      4.941         nan 
      0.938         nan 
 
 ESC13  14194.0 
     22.900         nan 
    353.163         nan 
      0.995         nan 
    232.800         nan 
      4.947         nan 
      0.938         nan 
 
 ESC13  16558.0 
     23.653         nan 
    378.718         nan 
      0.995         nan 
    245.200         nan 
      4.949         nan 
      0.938         nan 
 
 ESC13  18922.0 
     24.341         nan 
    411.125         nan 
      0.995         nan 
    257.100         nan 
      4.949         nan 
      0.938         nan 
 
 ESC13  21286.0 
     24.932         nan 
    434.525         nan 
      0.996         nan 
    265.800         nan 
      4.952         nan 
      0.938         nan 
 
 ESC13  23650.0 
     25.676         nan 
    456.077         nan 
      0.996         nan 
    277.000         nan 
      4.954         nan 
      0.938         nan 
 
 ESC14  10.0 
      2.997         nan 
     14.950         nan 
      0.390         nan 
      8.000         nan 
      2.914         nan 
      0.858         nan 
 
 ESC14  2374.0 
     29.269         nan 
    246.070         nan 
      0.972         nan 
    166.800         nan 
      5.122         nan 
      0.940         nan 
 
 ESC14  4738.0 
     39.265         nan 
    290.473         nan 
      0.985         nan 
    213.900         nan 
      5.150         nan 
      0.940         nan 
 
 ESC14  7102.0 
     43.476         nan 
    316.376         nan 
      0.990         nan 
    241.600         nan 
      5.179         nan 
      0.941         nan 
 
 ESC14  9466.0 
     46.962         nan 
    340.843         nan 
      0.992         nan 
    262.500         nan 
      5.181         nan 
      0.940         nan 
 
 ESC14  11830.0 
     49.084         nan 
    357.315         nan 
      0.994         nan 
    274.800         nan 
      5.176         nan 
      0.940         nan 
 
 ESC14  14194.0 
     50.933         nan 
    381.621         nan 
      0.995         nan 
    290.300         nan 
      5.189         nan 
      0.940         nan 
 
 ESC14  16558.0 
     53.754         nan 
    432.147         nan 
      0.995         nan 
    304.500         nan 
      5.187         nan 
      0.940         nan 
 
 ESC14  18922.0 
     56.514         nan 
    439.333         nan 
      0.996         nan 
    314.800         nan 
      5.193         nan 
      0.941         nan 
 
 ESC14  21286.0 
     57.452         nan 
    450.257         nan 
      0.996         nan 
    325.700         nan 
      5.195         nan 
      0.940         nan 
 
 ESC14  23650.0 
     59.022         nan 
    472.284         nan 
      0.996         nan 
    335.000         nan 
      5.195         nan 
      0.940         nan 
 
 ESC15  10.0 
      2.079         nan 
     15.900         nan 
      0.540         nan 
      6.100         nan 
      2.240         nan 
      0.714         nan 
 
 ESC15  2374.0 
     14.756         nan 
    208.155         nan 
      0.976         nan 
    121.300         nan 
      3.564         nan 
      0.767         nan 
 
 ESC15  4738.0 
     18.402         nan 
    262.281         nan 
      0.985         nan 
    169.300         nan 
      3.622         nan 
      0.767         nan 
 
 ESC15  7102.0 
     20.168         nan 
    292.030         nan 
      0.989         nan 
    199.100         nan 
      3.624         nan 
      0.764         nan 
 
 ESC15  9466.0 
     21.716         nan 
    346.002         nan 
      0.991         nan 
    224.600         nan 
      3.647         nan 
      0.768         nan 
 
 ESC15  11830.0 
     22.513         nan 
    340.219         nan 
      0.993         nan 
    240.000         nan 
      3.626         nan 
      0.764         nan 
 
 ESC15  14194.0 
     23.485         nan 
    363.280         nan 
      0.994         nan 
    253.900         nan 
      3.632         nan 
      0.764         nan 
 
 ESC15  16558.0 
     24.309         nan 
    384.551         nan 
      0.995         nan 
    268.900         nan 
      3.644         nan 
      0.766         nan 
 
 ESC15  18922.0 
     25.028         nan 
    421.217         nan 
      0.995         nan 
    282.100         nan 
      3.642         nan 
      0.765         nan 
 
 ESC15  21286.0 
     25.544         nan 
    429.607         nan 
      0.995         nan 
    292.900         nan 
      3.648         nan 
      0.766         nan 
 
 ESC15  23650.0 
     26.184         nan 
    457.667         nan 
      0.996         nan 
    306.000         nan 
      3.649         nan 
      0.766         nan 
 
 ESC16  10.0 
      2.241         nan 
     21.200         nan 
      0.380         nan 
      7.400         nan 
      2.648         nan 
      0.798         nan 
 
 ESC16  2374.0 
     16.922         nan 
    196.548         nan 
      0.981         nan 
    144.600         nan 
      4.595         nan 
      0.873         nan 
 
 ESC16  4738.0 
     19.150         nan 
    250.358         nan 
      0.989         nan 
    179.500         nan 
      4.649         nan 
      0.875         nan 
 
 ESC16  7102.0 
     20.801         nan 
    303.335         nan 
      0.991         nan 
    204.100         nan 
      4.640         nan 
      0.874         nan 
 
 ESC16  9466.0 
     22.319         nan 
    320.978         nan 
      0.993         nan 
    222.700         nan 
      4.671         nan 
      0.875         nan 
 
 ESC16  11830.0 
     22.934         nan 
    329.864         nan 
      0.994         nan 
    236.000         nan 
      4.678         nan 
      0.875         nan 
 
 ESC16  14194.0 
     23.832         nan 
    355.689         nan 
      0.995         nan 
    250.700         nan 
      4.669         nan 
      0.875         nan 
 
 ESC16  16558.0 
     24.784         nan 
    369.290         nan 
      0.995         nan 
    262.100         nan 
      4.681         nan 
      0.875         nan 
 
 ESC16  18922.0 
     25.271         nan 
    397.811         nan 
      0.996         nan 
    272.800         nan 
      4.676         nan 
      0.875         nan 
 
 ESC16  21286.0 
     26.058         nan 
    403.882         nan 
      0.996         nan 
    284.000         nan 
      4.681         nan 
      0.875         nan 
 
 ESC16  23650.0 
     26.715         nan 
    419.156         nan 
      0.996         nan 
    294.000         nan 
      4.681         nan 
      0.875         nan 
 
 ESC17  10.0 
      2.786         nan 
     30.000         nan 
      0.230         nan 
      8.700         nan 
      3.039         nan 
      0.868         nan 
 
 ESC17  2374.0 
     15.840         nan 
    199.089         nan 
      0.980         nan 
    143.900         nan 
      5.047         nan 
      0.930         nan 
 
 ESC17  4738.0 
     18.198         nan 
    250.379         nan 
      0.988         nan 
    180.500         nan 
      5.089         nan 
      0.931         nan 
 
 ESC17  7102.0 
     19.774         nan 
    270.838         nan 
      0.992         nan 
    204.900         nan 
      5.117         nan 
      0.932         nan 
 
 ESC17  9466.0 
     20.788         nan 
    283.327         nan 
      0.994         nan 
    218.400         nan 
      5.108         nan 
      0.931         nan 
 
 ESC17  11830.0 
     21.736         nan 
    301.470         nan 
      0.995         nan 
    232.800         nan 
      5.123         nan 
      0.932         nan 
 
 ESC17  14194.0 
     22.475         nan 
    327.308         nan 
      0.996         nan 
    246.100         nan 
      5.122         nan 
      0.931         nan 
 
 ESC17  16558.0 
     22.937         nan 
    352.631         nan 
      0.996         nan 
    253.200         nan 
      5.121         nan 
      0.931         nan 
 
 ESC17  18922.0 
     23.447         nan 
    381.996         nan 
      0.996         nan 
    262.400         nan 
      5.121         nan 
      0.931         nan 
 
 ESC17  21286.0 
     23.947         nan 
    384.180         nan 
      0.997         nan 
    270.500         nan 
      5.124         nan 
      0.931         nan 
 
 ESC17  23650.0 
     24.466         nan 
    421.500         nan 
      0.997         nan 
    279.000         nan 
      5.125         nan 
      0.931         nan 
 
 ESC18  10.0 
      2.675         nan 
     22.100         nan 
      0.270         nan 
      8.600         nan 
      3.034         nan 
      0.870         nan 
 
 ESC18  2374.0 
     17.281         nan 
    183.766         nan 
      0.983         nan 
    134.500         nan 
      5.144         nan 
      0.935         nan 
 
 ESC18  4738.0 
     19.552         nan 
    224.452         nan 
      0.990         nan 
    164.800         nan 
      5.184         nan 
      0.936         nan 
 
 ESC18  7102.0 
     21.014         nan 
    261.030         nan 
      0.993         nan 
    183.100         nan 
      5.174         nan 
      0.935         nan 
 
 ESC18  9466.0 
     21.921         nan 
    304.196         nan 
      0.994         nan 
    198.000         nan 
      5.186         nan 
      0.936         nan 
 
 ESC18  11830.0 
     23.455         nan 
    321.136         nan 
      0.994         nan 
    219.000         nan 
      5.188         nan 
      0.936         nan 
 
 ESC18  14194.0 
     23.926         nan 
    336.354         nan 
      0.995         nan 
    225.400         nan 
      5.186         nan 
      0.935         nan 
 
 ESC18  16558.0 
     24.724         nan 
    339.511         nan 
      0.996         nan 
    238.700         nan 
      5.198         nan 
      0.936         nan 
 
 ESC18  18922.0 
     25.350         nan 
    338.519         nan 
      0.996         nan 
    245.500         nan 
      5.196         nan 
      0.936         nan 
 
 ESC18  21286.0 
     25.732         nan 
    346.507         nan 
      0.996         nan 
    256.400         nan 
      5.199         nan 
      0.936         nan 
 
 ESC18  23650.0 
     26.320         nan 
    350.583         nan 
      0.997         nan 
    265.000         nan 
      5.201         nan 
      0.936         nan 
 
 ESC19  10.0 
      2.157         nan 
     11.700         nan 
      0.500         nan 
      7.300         nan 
      2.742         nan 
      0.834         nan 
 
 ESC19  2374.0 
     15.642         nan 
    201.817         nan 
      0.981         nan 
    145.200         nan 
      5.004         nan 
      0.933         nan 
 
 ESC19  4738.0 
     18.000         nan 
    257.272         nan 
      0.989         nan 
    180.000         nan 
      5.035         nan 
      0.932         nan 
 
 ESC19  7102.0 
     19.352         nan 
    290.652         nan 
      0.992         nan 
    204.400         nan 
      5.053         nan 
      0.932         nan 
 
 ESC19  9466.0 
     19.926         nan 
    290.575         nan 
      0.994         nan 
    216.700         nan 
      5.046         nan 
      0.932         nan 
 
 ESC19  11830.0 
     20.763         nan 
    310.957         nan 
      0.995         nan 
    230.900         nan 
      5.048         nan 
      0.932         nan 
 
 ESC19  14194.0 
     21.276         nan 
    337.694         nan 
      0.995         nan 
    242.600         nan 
      5.054         nan 
      0.932         nan 
 
 ESC19  16558.0 
     21.801         nan 
    334.234         nan 
      0.996         nan 
    251.100         nan 
      5.059         nan 
      0.932         nan 
 
 ESC19  18922.0 
     22.051         nan 
    335.593         nan 
      0.996         nan 
    259.400         nan 
      5.064         nan 
      0.932         nan 
 
 ESC19  21286.0 
     22.642         nan 
    343.034         nan 
      0.997         nan 
    268.600         nan 
      5.065         nan 
      0.932         nan 
 
 ESC19  23650.0 
     23.048         nan 
    345.000         nan 
      0.997         nan 
    276.000         nan 
      5.067         nan 
      0.932         nan 
 
 ESC20  10.0 
      2.420         nan 
     15.000         nan 
      0.530         nan 
      6.600         nan 
      2.510         nan 
      0.794         nan 
 
 ESC20  2374.0 
     19.452         nan 
    260.956         nan 
      0.970         nan 
    186.700         nan 
      4.987         nan 
      0.929         nan 
 
 ESC20  4738.0 
     22.731         nan 
    330.054         nan 
      0.984         nan 
    237.800         nan 
      5.007         nan 
      0.928         nan 
 
 ESC20  7102.0 
     24.125         nan 
    347.999         nan 
      0.990         nan 
    262.900         nan 
      5.051         nan 
      0.929         nan 
 
 ESC20  9466.0 
     25.829         nan 
    379.714         nan 
      0.992         nan 
    287.700         nan 
      5.045         nan 
      0.928         nan 
 
 ESC20  11830.0 
     26.641         nan 
    399.649         nan 
      0.993         nan 
    304.300         nan 
      5.049         nan 
      0.928         nan 
 
 ESC20  14194.0 
     27.386         nan 
    427.085         nan 
      0.994         nan 
    317.500         nan 
      5.056         nan 
      0.928         nan 
 
 ESC20  16558.0 
     28.039         nan 
    426.021         nan 
      0.995         nan 
    330.200         nan 
      5.057         nan 
      0.928         nan 
 
 ESC20  18922.0 
     29.130         nan 
    465.010         nan 
      0.995         nan 
    346.400         nan 
      5.062         nan 
      0.928         nan 
 
 ESC20  21286.0 
     29.532         nan 
    458.946         nan 
      0.996         nan 
    355.800         nan 
      5.063         nan 
      0.928         nan 
 
 ESC20  23650.0 
     30.056         nan 
    471.333         nan 
      0.996         nan 
    364.000         nan 
      5.063         nan 
      0.928         nan 
 
 ESC21  10.0 
      3.309         nan 
      4.700         nan 
      0.850         nan 
      3.200         nan 
      1.217         nan 
      0.472         nan 
 
 ESC21  2374.0 
     41.442         nan 
    280.528         nan 
      0.966         nan 
    132.200         nan 
      2.284         nan 
      0.613         nan 
 
 ESC21  4738.0 
     54.680         nan 
    386.988         nan 
      0.976         nan 
    205.300         nan 
      2.347         nan 
      0.613         nan 
 
 ESC21  7102.0 
     62.960         nan 
    400.966         nan 
      0.983         nan 
    247.400         nan 
      2.344         nan 
      0.615         nan 
 
 ESC21  9466.0 
     67.951         nan 
    450.803         nan 
      0.986         nan 
    283.400         nan 
      2.361         nan 
      0.614         nan 
 
 ESC21  11830.0 
     74.320         nan 
    496.420         nan 
      0.988         nan 
    315.300         nan 
      2.372         nan 
      0.614         nan 
 
 ESC21  14194.0 
     74.665         nan 
    503.721         nan 
      0.990         nan 
    334.200         nan 
      2.379         nan 
      0.614         nan 
 
 ESC21  16558.0 
     78.220         nan 
    527.246         nan 
      0.991         nan 
    357.100         nan 
      2.375         nan 
      0.614         nan 
 
 ESC21  18922.0 
     82.014         nan 
    549.018         nan 
      0.992         nan 
    379.700         nan 
      2.383         nan 
      0.615         nan 
 
 ESC21  21286.0 
     84.108         nan 
    542.475         nan 
      0.993         nan 
    394.900         nan 
      2.386         nan 
      0.614         nan 
 
 ESC21  23650.0 
     85.980         nan 
    558.000         nan 
      0.994         nan 
    411.000         nan 
      2.389         nan 
      0.615         nan 
 
 ESC22  10.0 
      2.214         nan 
      9.103         nan 
      0.670         nan 
      5.700         nan 
      2.281         nan 
      0.760         nan 
 
 ESC22  2374.0 
     33.667         nan 
    297.102         nan 
      0.964         nan 
    164.600         nan 
      3.801         nan 
      0.825         nan 
 
 ESC22  4738.0 
     42.254         nan 
    412.722         nan 
      0.976         nan 
    234.400         nan 
      3.810         nan 
      0.821         nan 
 
 ESC22  7102.0 
     48.639         nan 
    452.017         nan 
      0.982         nan 
    282.000         nan 
      3.816         nan 
      0.820         nan 
 
 ESC22  9466.0 
     52.900         nan 
    470.933         nan 
      0.986         nan 
    319.400         nan 
      3.848         nan 
      0.822         nan 
 
 ESC22  11830.0 
     57.909         nan 
    526.533         nan 
      0.988         nan 
    352.000         nan 
      3.869         nan 
      0.823         nan 
 
 ESC22  14194.0 
     61.279         nan 
    545.058         nan 
      0.990         nan 
    371.200         nan 
      3.864         nan 
      0.822         nan 
 
 ESC22  16558.0 
     62.025         nan 
    549.461         nan 
      0.991         nan 
    396.600         nan 
      3.873         nan 
      0.823         nan 
 
 ESC22  18922.0 
     66.610         nan 
    577.083         nan 
      0.992         nan 
    417.200         nan 
      3.866         nan 
      0.822         nan 
 
 ESC22  21286.0 
     68.770         nan 
    573.221         nan 
      0.993         nan 
    432.000         nan 
      3.870         nan 
      0.822         nan 
 
 ESC22  23650.0 
     70.545         nan 
    584.835         nan 
      0.994         nan 
    449.000         nan 
      3.873         nan 
      0.822         nan 
 
 ESC23  10.0 
      4.740         nan 
     14.750         nan 
      0.470         nan 
      7.300         nan 
      2.716         nan 
      0.826         nan 
 
 ESC23  2374.0 
     33.870         nan 
    266.503         nan 
      0.973         nan 
    134.800         nan 
      4.757         nan 
      0.933         nan 
 
 ESC23  4738.0 
     44.642         nan 
    358.060         nan 
      0.982         nan 
    181.800         nan 
      4.781         nan 
      0.933         nan 
 
 ESC23  7102.0 
     49.938         nan 
    380.349         nan 
      0.985         nan 
    225.600         nan 
      4.807         nan 
      0.933         nan 
 
 ESC23  9466.0 
     55.361         nan 
    445.359         nan 
      0.987         nan 
    255.200         nan 
      4.803         nan 
      0.933         nan 
 
 ESC23  11830.0 
     58.046         nan 
    479.785         nan 
      0.989         nan 
    280.400         nan 
      4.809         nan 
      0.933         nan 
 
 ESC23  14194.0 
     62.110         nan 
    522.651         nan 
      0.990         nan 
    309.000         nan 
      4.822         nan 
      0.933         nan 
 
 ESC23  16558.0 
     66.084         nan 
    516.157         nan 
      0.991         nan 
    332.500         nan 
      4.826         nan 
      0.933         nan 
 
 ESC23  18922.0 
     70.839         nan 
    517.421         nan 
      0.992         nan 
    352.000         nan 
      4.830         nan 
      0.933         nan 
 
 ESC23  21286.0 
     71.902         nan 
    535.682         nan 
      0.993         nan 
    368.900         nan 
      4.828         nan 
      0.933         nan 
 
 ESC23  23650.0 
     75.490         nan 
    546.203         nan 
      0.993         nan 
    387.000         nan 
      4.831         nan 
      0.933         nan 
 
 ESC24  10.0 
      2.552         nan 
     18.750         nan 
      0.460         nan 
      6.800         nan 
      2.489         nan 
      0.774         nan 
 
 ESC24  2374.0 
     35.637         nan 
    283.792         nan 
      0.968         nan 
    154.900         nan 
      4.095         nan 
      0.811         nan 
 
 ESC24  4738.0 
     49.767         nan 
    381.103         nan 
      0.979         nan 
    217.800         nan 
      4.149         nan 
      0.811         nan 
 
 ESC24  7102.0 
     55.810         nan 
    440.088         nan 
      0.983         nan 
    261.500         nan 
      4.138         nan 
      0.807         nan 
 
 ESC24  9466.0 
     63.340         nan 
    472.241         nan 
      0.986         nan 
    298.600         nan 
      4.161         nan 
      0.808         nan 
 
 ESC24  11830.0 
     68.420         nan 
    542.896         nan 
      0.988         nan 
    332.100         nan 
      4.161         nan 
      0.807         nan 
 
 ESC24  14194.0 
     75.366         nan 
    581.185         nan 
      0.989         nan 
    361.900         nan 
      4.163         nan 
      0.807         nan 
 
 ESC24  16558.0 
     78.226         nan 
    596.600         nan 
      0.991         nan 
    378.600         nan 
      4.176         nan 
      0.808         nan 
 
 ESC24  18922.0 
     81.546         nan 
    608.490         nan 
      0.992         nan 
    396.600         nan 
      4.170         nan 
      0.808         nan 
 
 ESC24  21286.0 
     84.601         nan 
    633.035         nan 
      0.992         nan 
    416.800         nan 
      4.177         nan 
      0.808         nan 
 
 ESC24  23650.0 
     87.128         nan 
    674.112         nan 
      0.993         nan 
    436.900         nan 
      4.179         nan 
      0.808         nan 
 
 ESC25  10.0 
      7.151         nan 
     13.200         nan 
      0.540         nan 
      6.500         nan 
      2.463         nan 
      0.782         nan 
 
 ESC25  2374.0 
     63.530         nan 
    255.935         nan 
      0.971         nan 
    157.100         nan 
      4.265         nan 
      0.857         nan 
 
 ESC25  4738.0 
     82.258         nan 
    322.605         nan 
      0.983         nan 
    207.900         nan 
      4.300         nan 
      0.856         nan 
 
 ESC25  7102.0 
     90.964         nan 
    393.132         nan 
      0.987         nan 
    244.500         nan 
      4.327         nan 
      0.858         nan 
 
 ESC25  9466.0 
    100.116         nan 
    426.353         nan 
      0.989         nan 
    271.500         nan 
      4.344         nan 
      0.858         nan 
 
 ESC25  11830.0 
    106.658         nan 
    460.005         nan 
      0.991         nan 
    292.900         nan 
      4.336         nan 
      0.858         nan 
 
 ESC25  14194.0 
    110.192         nan 
    464.944         nan 
      0.992         nan 
    311.700         nan 
      4.347         nan 
      0.858         nan 
 
 ESC25  16558.0 
    113.566         nan 
    491.435         nan 
      0.993         nan 
    332.600         nan 
      4.345         nan 
      0.857         nan 
 
 ESC25  18922.0 
    116.402         nan 
    509.861         nan 
      0.994         nan 
    345.600         nan 
      4.346         nan 
      0.857         nan 
 
 ESC25  21286.0 
    117.819         nan 
    525.529         nan 
      0.994         nan 
    363.000         nan 
      4.353         nan 
      0.857         nan 
 
 ESC25  23650.0 
    120.039         nan 
    531.774         nan 
      0.995         nan 
    376.000         nan 
      4.353         nan 
      0.857         nan 
 
 ESC26  10.0 
      3.416         nan 
     14.900         nan 
      0.460         nan 
      7.300         nan 
      2.712         nan 
      0.826         nan 
 
 ESC26  2374.0 
     31.453         nan 
    286.671         nan 
      0.967         nan 
    164.400         nan 
      4.444         nan 
      0.878         nan 
 
 ESC26  4738.0 
     44.775         nan 
    398.316         nan 
      0.977         nan 
    235.700         nan 
      4.557         nan 
      0.882         nan 
 
 ESC26  7102.0 
     49.936         nan 
    479.926         nan 
      0.982         nan 
    285.000         nan 
      4.581         nan 
      0.882         nan 
 
 ESC26  9466.0 
     54.694         nan 
    506.726         nan 
      0.986         nan 
    317.000         nan 
      4.566         nan 
      0.880         nan 
 
 ESC26  11830.0 
     56.871         nan 
    563.134         nan 
      0.988         nan 
    348.700         nan 
      4.577         nan 
      0.880         nan 
 
 ESC26  14194.0 
     63.112         nan 
    567.911         nan 
      0.989         nan 
    374.300         nan 
      4.576         nan 
      0.880         nan 
 
 ESC26  16558.0 
     65.012         nan 
    579.692         nan 
      0.991         nan 
    397.700         nan 
      4.587         nan 
      0.880         nan 
 
 ESC26  18922.0 
     69.372         nan 
    604.645         nan 
      0.992         nan 
    421.500         nan 
      4.585         nan 
      0.880         nan 
 
 ESC26  21286.0 
     70.372         nan 
    615.888         nan 
      0.992         nan 
    437.800         nan 
      4.593         nan 
      0.880         nan 
 
 ESC26  23650.0 
     72.932         nan 
    622.981         nan 
      0.993         nan 
    456.000         nan 
      4.595         nan 
      0.880         nan 
 
 ESC27  10.0 
      2.977         nan 
     20.000         nan 
      0.330         nan 
      8.200         nan 
      2.934         nan 
      0.856         nan 
 
 ESC27  2374.0 
     29.288         nan 
    329.715         nan 
      0.964         nan 
    179.700         nan 
      5.044         nan 
      0.931         nan 
 
 ESC27  4738.0 
     36.948         nan 
    398.684         nan 
      0.978         nan 
    239.400         nan 
      5.113         nan 
      0.934         nan 
 
 ESC27  7102.0 
     46.143         nan 
    472.054         nan 
      0.982         nan 
    288.600         nan 
      5.122         nan 
      0.933         nan 
 
 ESC27  9466.0 
     47.764         nan 
    501.854         nan 
      0.986         nan 
    324.000         nan 
      5.141         nan 
      0.934         nan 
 
 ESC27  11830.0 
     52.860         nan 
    514.326         nan 
      0.988         nan 
    357.500         nan 
      5.152         nan 
      0.934         nan 
 
 ESC27  14194.0 
     55.531         nan 
    566.615         nan 
      0.989         nan 
    384.600         nan 
      5.145         nan 
      0.933         nan 
 
 ESC27  16558.0 
     57.480         nan 
    589.252         nan 
      0.991         nan 
    408.000         nan 
      5.150         nan 
      0.933         nan 
 
 ESC27  18922.0 
     60.193         nan 
    594.965         nan 
      0.992         nan 
    432.100         nan 
      5.160         nan 
      0.933         nan 
 
 ESC27  21286.0 
     61.016         nan 
    596.983         nan 
      0.993         nan 
    446.900         nan 
      5.157         nan 
      0.933         nan 
 
 ESC27  23650.0 
     63.057         nan 
    612.127         nan 
      0.993         nan 
    463.000         nan 
      5.161         nan 
      0.933         nan 
 
 ESC28  10.0 
      2.032         nan 
      7.883         nan 
      0.670         nan 
      5.500         nan 
      2.187         nan 
      0.738         nan 
 
 ESC28  2374.0 
     31.044         nan 
    295.640         nan 
      0.965         nan 
    138.300         nan 
      3.243         nan 
      0.794         nan 
 
 ESC28  4738.0 
     41.675         nan 
    393.411         nan 
      0.976         nan 
    208.200         nan 
      3.306         nan 
      0.796         nan 
 
 ESC28  7102.0 
     51.272         nan 
    454.485         nan 
      0.982         nan 
    256.600         nan 
      3.320         nan 
      0.795         nan 
 
 ESC28  9466.0 
     54.463         nan 
    481.813         nan 
      0.985         nan 
    292.600         nan 
      3.327         nan 
      0.796         nan 
 
 ESC28  11830.0 
     60.571         nan 
    489.165         nan 
      0.988         nan 
    326.300         nan 
      3.341         nan 
      0.796         nan 
 
 ESC28  14194.0 
     63.623         nan 
    528.016         nan 
      0.990         nan 
    347.600         nan 
      3.340         nan 
      0.796         nan 
 
 ESC28  16558.0 
     70.509         nan 
    569.838         nan 
      0.991         nan 
    375.100         nan 
      3.337         nan 
      0.795         nan 
 
 ESC28  18922.0 
     72.829         nan 
    608.338         nan 
      0.991         nan 
    395.400         nan 
      3.345         nan 
      0.795         nan 
 
 ESC28  21286.0 
     74.586         nan 
    613.814         nan 
      0.992         nan 
    414.300         nan 
      3.353         nan 
      0.796         nan 
 
 ESC28  23650.0 
     78.331         nan 
    627.743         nan 
      0.993         nan 
    432.000         nan 
      3.351         nan 
      0.795         nan 
 
 ESC29  10.0 
      2.568         nan 
     15.833         nan 
      0.500         nan 
      6.600         nan 
      2.464         nan 
      0.778         nan 
 
 ESC29  2374.0 
     15.276         nan 
    168.825         nan 
      0.984         nan 
    131.200         nan 
      4.403         nan 
      0.847         nan 
 
 ESC29  4738.0 
     17.259         nan 
    194.197         nan 
      0.992         nan 
    157.000         nan 
      4.452         nan 
      0.848         nan 
 
 ESC29  7102.0 
     18.456         nan 
    223.193         nan 
      0.994         nan 
    174.700         nan 
      4.426         nan 
      0.846         nan 
 
 ESC29  9466.0 
     19.308         nan 
    232.427         nan 
      0.996         nan 
    184.500         nan 
      4.448         nan 
      0.846         nan 
 
 ESC29  11830.0 
     19.714         nan 
    228.759         nan 
      0.997         nan 
    192.000         nan 
      4.442         nan 
      0.846         nan 
 
 ESC29  14194.0 
     20.234         nan 
    255.240         nan 
      0.997         nan 
    201.300         nan 
      4.451         nan 
      0.846         nan 
 
 ESC29  16558.0 
     20.680         nan 
    264.329         nan 
      0.997         nan 
    207.400         nan 
      4.447         nan 
      0.846         nan 
 
 ESC29  18922.0 
     21.033         nan 
    271.868         nan 
      0.998         nan 
    213.300         nan 
      4.452         nan 
      0.846         nan 
 
 ESC29  21286.0 
     21.493         nan 
    276.009         nan 
      0.998         nan 
    220.100         nan 
      4.456         nan 
      0.846         nan 
 
 ESC29  23650.0 
     21.734         nan 
    280.895         nan 
      0.998         nan 
    224.000         nan 
      4.455         nan 
      0.846         nan 
 
 ESC30  10.0 
      4.360         nan 
     21.450         nan 
      0.350         nan 
      7.900         nan 
      2.824         nan 
      0.832         nan 
 
 ESC30  2374.0 
     51.296         nan 
    260.591         nan 
      0.971         nan 
    174.600         nan 
      4.860         nan 
      0.912         nan 
 
 ESC30  4738.0 
     64.728         nan 
    344.870         nan 
      0.982         nan 
    234.500         nan 
      4.928         nan 
      0.913         nan 
 
 ESC30  7102.0 
     74.928         nan 
    405.234         nan 
      0.987         nan 
    264.800         nan 
      4.924         nan 
      0.913         nan 
 
 ESC30  9466.0 
     81.336         nan 
    445.658         nan 
      0.990         nan 
    288.900         nan 
      4.932         nan 
      0.912         nan 
 
 ESC30  11830.0 
     85.926         nan 
    466.378         nan 
      0.991         nan 
    314.700         nan 
      4.938         nan 
      0.912         nan 
 
 ESC30  14194.0 
     88.519         nan 
    539.392         nan 
      0.992         nan 
    335.000         nan 
      4.945         nan 
      0.912         nan 
 
 ESC30  16558.0 
     95.119         nan 
    528.881         nan 
      0.993         nan 
    352.100         nan 
      4.946         nan 
      0.912         nan 
 
 ESC30  18922.0 
     97.424         nan 
    567.726         nan 
      0.993         nan 
    370.600         nan 
      4.948         nan 
      0.912         nan 
 
 ESC30  21286.0 
     99.511         nan 
    572.324         nan 
      0.994         nan 
    384.200         nan 
      4.950         nan 
      0.912         nan 
 
 ESC30  23650.0 
    102.979         nan 
    581.300         nan 
      0.994         nan 
    398.900         nan 
      4.954         nan 
      0.912         nan 
 
 ESC31  10.0 
      3.369         nan 
     25.400         nan 
      0.300         nan 
      8.200         nan 
      2.912         nan 
      0.850         nan 
 
 ESC31  2374.0 
     51.100         nan 
    307.212         nan 
      0.965         nan 
    194.000         nan 
      5.216         nan 
      0.925         nan 
 
 ESC31  4738.0 
     65.330         nan 
    398.518         nan 
      0.978         nan 
    255.200         nan 
      5.247         nan 
      0.925         nan 
 
 ESC31  7102.0 
     70.996         nan 
    457.753         nan 
      0.983         nan 
    298.400         nan 
      5.275         nan 
      0.926         nan 
 
 ESC31  9466.0 
     78.172         nan 
    482.928         nan 
      0.987         nan 
    333.800         nan 
      5.291         nan 
      0.925         nan 
 
 ESC31  11830.0 
     82.152         nan 
    518.687         nan 
      0.989         nan 
    362.200         nan 
      5.303         nan 
      0.925         nan 
 
 ESC31  14194.0 
     85.671         nan 
    545.586         nan 
      0.991         nan 
    384.100         nan 
      5.296         nan 
      0.925         nan 
 
 ESC31  16558.0 
     90.438         nan 
    570.843         nan 
      0.992         nan 
    401.900         nan 
      5.300         nan 
      0.925         nan 
 
 ESC31  18922.0 
     92.695         nan 
    580.863         nan 
      0.993         nan 
    422.200         nan 
      5.302         nan 
      0.925         nan 
 
 ESC31  21286.0 
     96.067         nan 
    622.081         nan 
      0.993         nan 
    440.000         nan 
      5.306         nan 
      0.925         nan 
 
 ESC31  23650.0 
     98.427         nan 
    641.294         nan 
      0.994         nan 
    455.000         nan 
      5.310         nan 
      0.925         nan 
 
 ESC32  10.0 
      1.419         nan 
      4.100         nan 
      0.860         nan 
      3.600         nan 
      1.542         nan 
      0.602         nan 
 
 ESC32  2374.0 
      9.078         nan 
    143.488         nan 
      0.984         nan 
     65.400         nan 
      2.112         nan 
      0.644         nan 
 
 ESC32  4738.0 
     11.464         nan 
    169.426         nan 
      0.990         nan 
     97.000         nan 
      2.157         nan 
      0.647         nan 
 
 ESC32  7102.0 
     13.217         nan 
    193.740         nan 
      0.992         nan 
    118.000         nan 
      2.154         nan 
      0.646         nan 
 
 ESC32  9466.0 
     14.126         nan 
    222.812         nan 
      0.993         nan 
    134.800         nan 
      2.159         nan 
      0.645         nan 
 
 ESC32  11830.0 
     15.045         nan 
    230.028         nan 
      0.994         nan 
    149.700         nan 
      2.158         nan 
      0.645         nan 
 
 ESC32  14194.0 
     15.961         nan 
    245.704         nan 
      0.995         nan 
    162.700         nan 
      2.165         nan 
      0.646         nan 
 
 ESC32  16558.0 
     16.549         nan 
    253.559         nan 
      0.996         nan 
    173.200         nan 
      2.163         nan 
      0.645         nan 
 
 ESC32  18922.0 
     17.298         nan 
    275.243         nan 
      0.996         nan 
    183.300         nan 
      2.169         nan 
      0.645         nan 
 
 ESC32  21286.0 
     17.841         nan 
    276.801         nan 
      0.997         nan 
    191.200         nan 
      2.169         nan 
      0.645         nan 
 
 ESC32  23650.0 
     18.270         nan 
    292.495         nan 
      0.997         nan 
    200.000         nan 
      2.171         nan 
      0.645         nan 
 
 Nor1  10.0 
      1.824         nan 
     18.950         nan 
      0.480         nan 
      6.300         nan 
      2.253         nan 
      0.710         nan 
 
 Nor1  2374.0 
     15.337         nan 
    221.003         nan 
      0.980         nan 
    131.300         nan 
      4.311         nan 
      0.829         nan 
 
 Nor1  4738.0 
     18.795         nan 
    284.445         nan 
      0.986         nan 
    174.200         nan 
      4.367         nan 
      0.833         nan 
 
 Nor1  7102.0 
     20.535         nan 
    289.682         nan 
      0.990         nan 
    197.700         nan 
      4.372         nan 
      0.831         nan 
 
 Nor1  9466.0 
     21.599         nan 
    312.118         nan 
      0.992         nan 
    215.100         nan 
      4.362         nan 
      0.831         nan 
 
 Nor1  11830.0 
     23.167         nan 
    333.112         nan 
      0.993         nan 
    237.400         nan 
      4.380         nan 
      0.832         nan 
 
 Nor1  14194.0 
     24.221         nan 
    379.151         nan 
      0.994         nan 
    256.800         nan 
      4.381         nan 
      0.832         nan 
 
 Nor1  16558.0 
     25.107         nan 
    371.563         nan 
      0.995         nan 
    269.200         nan 
      4.382         nan 
      0.831         nan 
 
 Nor1  18922.0 
     25.651         nan 
    404.237         nan 
      0.995         nan 
    281.400         nan 
      4.389         nan 
      0.832         nan 
 
 Nor1  21286.0 
     26.446         nan 
    414.484         nan 
      0.995         nan 
    294.000         nan 
      4.395         nan 
      0.832         nan 
 
 Nor1  23650.0 
     26.915         nan 
    428.079         nan 
      0.996         nan 
    303.000         nan 
      4.393         nan 
      0.832         nan 
 
 Nor2  10.0 
      1.909         nan 
     19.400         nan 
      0.390         nan 
      7.800         nan 
      2.844         nan 
      0.846         nan 
 
 Nor2  2374.0 
     14.011         nan 
    183.819         nan 
      0.985         nan 
    109.700         nan 
      5.062         nan 
      0.941         nan 
 
 Nor2  4738.0 
     16.873         nan 
    228.536         nan 
      0.990         nan 
    136.100         nan 
      5.077         nan 
      0.940         nan 
 
 Nor2  7102.0 
     19.813         nan 
    263.078         nan 
      0.992         nan 
    160.800         nan 
      5.087         nan 
      0.940         nan 
 
 Nor2  9466.0 
     21.448         nan 
    298.560         nan 
      0.993         nan 
    175.500         nan 
      5.087         nan 
      0.940         nan 
 
 Nor2  11830.0 
     21.807         nan 
    298.974         nan 
      0.994         nan 
    188.600         nan 
      5.089         nan 
      0.940         nan 
 
 Nor2  14194.0 
     23.436         nan 
    338.888         nan 
      0.995         nan 
    203.700         nan 
      5.097         nan 
      0.940         nan 
 
 Nor2  16558.0 
     25.311         nan 
    356.697         nan 
      0.995         nan 
    213.900         nan 
      5.096         nan 
      0.940         nan 
 
 Nor2  18922.0 
     27.258         nan 
    340.782         nan 
      0.996         nan 
    224.300         nan 
      5.101         nan 
      0.940         nan 
 
 Nor2  21286.0 
     28.407         nan 
    373.583         nan 
      0.996         nan 
    234.600         nan 
      5.097         nan 
      0.940         nan 
 
 Nor2  23650.0 
     29.928         nan 
    379.610         nan 
      0.996         nan 
    245.000         nan 
      5.100         nan 
      0.940         nan 
 
 Nor3  10.0 
      2.354         nan 
     29.600         nan 
      0.200         nan 
      8.900         nan 
      3.087         nan 
      0.874         nan 
 
 Nor3  2374.0 
     19.656         nan 
    249.297         nan 
      0.980         nan 
    208.000         nan 
      6.129         nan 
      0.966         nan 
 
 Nor3  4738.0 
     21.591         nan 
    273.896         nan 
      0.992         nan 
    236.900         nan 
      6.179         nan 
      0.967         nan 
 
 Nor3  7102.0 
     22.868         nan 
    294.220         nan 
      0.994         nan 
    251.700         nan 
      6.176         nan 
      0.966         nan 
 
 Nor3  9466.0 
     23.615         nan 
    314.247         nan 
      0.995         nan 
    263.100         nan 
      6.195         nan 
      0.966         nan 
 
 Nor3  11830.0 
     24.302         nan 
    349.618         nan 
      0.996         nan 
    272.300         nan 
      6.190         nan 
      0.966         nan 
 
 Nor3  14194.0 
     25.017         nan 
    331.400         nan 
      0.997         nan 
    279.900         nan 
      6.198         nan 
      0.966         nan 
 
 Nor3  16558.0 
     25.527         nan 
    336.438         nan 
      0.997         nan 
    286.400         nan 
      6.203         nan 
      0.966         nan 
 
 Nor3  18922.0 
     25.922         nan 
    356.584         nan 
      0.997         nan 
    292.800         nan 
      6.199         nan 
      0.966         nan 
 
 Nor3  21286.0 
     26.540         nan 
    357.733         nan 
      0.998         nan 
    298.800         nan 
      6.206         nan 
      0.966         nan 
 
 Nor3  23650.0 
     26.908         nan 
    360.000         nan 
      0.998         nan 
    304.000         nan 
      6.209         nan 
      0.966         nan 
 
 Nor4  10.0 
      2.176         nan 
     11.733         nan 
      0.560         nan 
      6.500         nan 
      2.483         nan 
      0.788         nan 
 
 Nor4  2374.0 
     12.691         nan 
    157.131         nan 
      0.988         nan 
     84.600         nan 
      4.523         nan 
      0.914         nan 
 
 Nor4  4738.0 
     15.266         nan 
    226.000         nan 
      0.990         nan 
    114.600         nan 
      4.534         nan 
      0.913         nan 
 
 Nor4  7102.0 
     16.441         nan 
    245.844         nan 
      0.992         nan 
    133.900         nan 
      4.546         nan 
      0.913         nan 
 
 Nor4  9466.0 
     17.934         nan 
    273.300         nan 
      0.993         nan 
    148.000         nan 
      4.554         nan 
      0.914         nan 
 
 Nor4  11830.0 
     19.286         nan 
    316.559         nan 
      0.994         nan 
    165.800         nan 
      4.554         nan 
      0.913         nan 
 
 Nor4  14194.0 
     20.064         nan 
    296.938         nan 
      0.995         nan 
    174.400         nan 
      4.559         nan 
      0.914         nan 
 
 Nor4  16558.0 
     21.415         nan 
    319.862         nan 
      0.995         nan 
    189.000         nan 
      4.563         nan 
      0.914         nan 
 
 Nor4  18922.0 
     22.150         nan 
    344.277         nan 
      0.995         nan 
    200.600         nan 
      4.563         nan 
      0.914         nan 
 
 Nor4  21286.0 
     23.124         nan 
    357.820         nan 
      0.996         nan 
    211.600         nan 
      4.568         nan 
      0.914         nan 
 
 Nor4  23650.0 
     23.807         nan 
    365.700         nan 
      0.996         nan 
    220.000         nan 
      4.567         nan 
      0.914         nan 
 
 Nor5  10.0 
      1.800         nan 
     19.250         nan 
      0.390         nan 
      7.600         nan 
      2.751         nan 
      0.824         nan 
 
 Nor5  2374.0 
     11.306         nan 
    196.043         nan 
      0.985         nan 
     83.700         nan 
      4.550         nan 
      0.927         nan 
 
 Nor5  4738.0 
     14.079         nan 
    191.923         nan 
      0.990         nan 
    107.900         nan 
      4.572         nan 
      0.928         nan 
 
 Nor5  7102.0 
     16.689         nan 
    214.221         nan 
      0.992         nan 
    125.400         nan 
      4.562         nan 
      0.927         nan 
 
 Nor5  9466.0 
     18.487         nan 
    240.293         nan 
      0.993         nan 
    148.100         nan 
      4.584         nan 
      0.927         nan 
 
 Nor5  11830.0 
     19.815         nan 
    256.584         nan 
      0.994         nan 
    159.500         nan 
      4.578         nan 
      0.927         nan 
 
 Nor5  14194.0 
     21.577         nan 
    264.210         nan 
      0.995         nan 
    175.800         nan 
      4.593         nan 
      0.928         nan 
 
 Nor5  16558.0 
     22.916         nan 
    293.483         nan 
      0.995         nan 
    185.400         nan 
      4.586         nan 
      0.927         nan 
 
 Nor5  18922.0 
     23.648         nan 
    290.308         nan 
      0.996         nan 
    197.400         nan 
      4.594         nan 
      0.928         nan 
 
 Nor5  21286.0 
     24.846         nan 
    292.251         nan 
      0.996         nan 
    204.300         nan 
      4.591         nan 
      0.927         nan 
 
 Nor5  23650.0 
     25.464         nan 
    298.515         nan 
      0.997         nan 
    213.900         nan 
      4.594         nan 
      0.928         nan 
 
 Nor6  10.0 
      2.752         nan 
     14.300         nan 
      0.460         nan 
      7.000         nan 
      2.572         nan 
      0.792         nan 
 
 Nor6  2374.0 
     10.389         nan 
    138.277         nan 
      0.989         nan 
     79.500         nan 
      4.495         nan 
      0.903         nan 
 
 Nor6  4738.0 
     11.911         nan 
    168.425         nan 
      0.992         nan 
     98.900         nan 
      4.513         nan 
      0.904         nan 
 
 Nor6  7102.0 
     13.064         nan 
    198.424         nan 
      0.994         nan 
    113.900         nan 
      4.523         nan 
      0.905         nan 
 
 Nor6  9466.0 
     13.847         nan 
    201.438         nan 
      0.995         nan 
    126.200         nan 
      4.522         nan 
      0.904         nan 
 
 Nor6  11830.0 
     14.801         nan 
    232.029         nan 
      0.995         nan 
    139.100         nan 
      4.520         nan 
      0.904         nan 
 
 Nor6  14194.0 
     15.416         nan 
    231.002         nan 
      0.996         nan 
    147.100         nan 
      4.515         nan 
      0.903         nan 
 
 Nor6  16558.0 
     16.120         nan 
    256.077         nan 
      0.996         nan 
    159.500         nan 
      4.527         nan 
      0.904         nan 
 
 Nor6  18922.0 
     16.507         nan 
    245.461         nan 
      0.997         nan 
    165.600         nan 
      4.528         nan 
      0.904         nan 
 
 Nor6  21286.0 
     17.214         nan 
    271.951         nan 
      0.997         nan 
    176.500         nan 
      4.528         nan 
      0.904         nan 
 
 Nor6  23650.0 
     17.647         nan 
    260.903         nan 
      0.997         nan 
    183.000         nan 
      4.529         nan 
      0.904         nan 
 
 Nor7  10.0 
      2.699         nan 
     16.050         nan 
      0.460         nan 
      6.900         nan 
      2.524         nan 
      0.780         nan 
 
 Nor7  2374.0 
     15.436         nan 
    156.992         nan 
      0.985         nan 
    106.100         nan 
      4.542         nan 
      0.869         nan 
 
 Nor7  4738.0 
     17.306         nan 
    197.869         nan 
      0.991         nan 
    132.500         nan 
      4.559         nan 
      0.868         nan 
 
 Nor7  7102.0 
     18.768         nan 
    232.030         nan 
      0.993         nan 
    151.400         nan 
      4.542         nan 
      0.865         nan 
 
 Nor7  9466.0 
     20.044         nan 
    269.059         nan 
      0.994         nan 
    169.000         nan 
      4.565         nan 
      0.868         nan 
 
 Nor7  11830.0 
     20.556         nan 
    248.212         nan 
      0.995         nan 
    177.900         nan 
      4.565         nan 
      0.867         nan 
 
 Nor7  14194.0 
     21.585         nan 
    262.653         nan 
      0.996         nan 
    190.700         nan 
      4.571         nan 
      0.867         nan 
 
 Nor7  16558.0 
     22.144         nan 
    278.336         nan 
      0.996         nan 
    200.600         nan 
      4.568         nan 
      0.867         nan 
 
 Nor7  18922.0 
     22.670         nan 
    303.186         nan 
      0.996         nan 
    209.200         nan 
      4.569         nan 
      0.867         nan 
 
 Nor7  21286.0 
     23.164         nan 
    306.966         nan 
      0.997         nan 
    217.000         nan 
      4.573         nan 
      0.867         nan 
 
 Nor7  23650.0 
     23.594         nan 
    321.750         nan 
      0.997         nan 
    224.000         nan 
      4.575         nan 
      0.867         nan 
 
 Nor8  10.0 
      2.194         nan 
     21.500         nan 
      0.320         nan 
      8.200         nan 
      2.932         nan 
      0.856         nan 
 
 Nor8  2374.0 
     13.409         nan 
    160.790         nan 
      0.987         nan 
    120.700         nan 
      5.292         nan 
      0.951         nan 
 
 Nor8  4738.0 
     15.081         nan 
    207.239         nan 
      0.992         nan 
    139.400         nan 
      5.302         nan 
      0.951         nan 
 
 Nor8  7102.0 
     16.445         nan 
    266.666         nan 
      0.993         nan 
    160.700         nan 
      5.319         nan 
      0.951         nan 
 
 Nor8  9466.0 
     17.475         nan 
    250.024         nan 
      0.995         nan 
    172.500         nan 
      5.319         nan 
      0.951         nan 
 
 Nor8  11830.0 
     18.121         nan 
    250.099         nan 
      0.996         nan 
    184.400         nan 
      5.322         nan 
      0.951         nan 
 
 Nor8  14194.0 
     19.293         nan 
    276.287         nan 
      0.996         nan 
    197.100         nan 
      5.333         nan 
      0.951         nan 
 
 Nor8  16558.0 
     19.562         nan 
    293.124         nan 
      0.996         nan 
    202.700         nan 
      5.331         nan 
      0.951         nan 
 
 Nor8  18922.0 
     20.241         nan 
    298.943         nan 
      0.997         nan 
    212.600         nan 
      5.332         nan 
      0.951         nan 
 
 Nor8  21286.0 
     20.717         nan 
    303.194         nan 
      0.997         nan 
    220.000         nan 
      5.333         nan 
      0.951         nan 
 
 Nor8  23650.0 
     21.265         nan 
    305.964         nan 
      0.997         nan 
    227.000         nan 
      5.333         nan 
      0.951         nan 
 
 Nor9  10.0 
      1.380         nan 
      5.200         nan 
      0.770         nan 
      3.300         nan 
      1.047         nan 
      0.376         nan 
 
 Nor9  2374.0 
      9.580         nan 
    101.990         nan 
      0.990         nan 
     67.100         nan 
      1.872         nan 
      0.391         nan 
 
 Nor9  4738.0 
     11.045         nan 
    139.375         nan 
      0.994         nan 
     81.300         nan 
      1.900         nan 
      0.396         nan 
 
 Nor9  7102.0 
     12.208         nan 
    167.109         nan 
      0.995         nan 
     96.500         nan 
      1.900         nan 
      0.394         nan 
 
 Nor9  9466.0 
     13.435         nan 
    172.365         nan 
      0.996         nan 
    108.000         nan 
      1.903         nan 
      0.394         nan 
 
 Nor9  11830.0 
     14.571         nan 
    206.595         nan 
      0.996         nan 
    119.800         nan 
      1.889         nan 
      0.390         nan 
 
 Nor9  14194.0 
     15.023         nan 
    224.605         nan 
      0.996         nan 
    127.200         nan 
      1.914         nan 
      0.395         nan 
 
 Nor9  16558.0 
     15.950         nan 
    214.333         nan 
      0.997         nan 
    137.300         nan 
      1.906         nan 
      0.393         nan 
 
 Nor9  18922.0 
     16.534         nan 
    233.043         nan 
      0.997         nan 
    144.300         nan 
      1.910         nan 
      0.393         nan 
 
 Nor9  21286.0 
     16.839         nan 
    236.571         nan 
      0.997         nan 
    150.000         nan 
      1.907         nan 
      0.393         nan 
 
 Nor9  23650.0 
     17.280         nan 
    243.143         nan 
      0.997         nan 
    156.000         nan 
      1.911         nan 
      0.394         nan 
 
 Nor10  10.0 
      2.550         nan 
     15.500         nan 
      0.400         nan 
      7.700         nan 
      2.807         nan 
      0.838         nan 
 
 Nor10  2374.0 
     16.672         nan 
    222.599         nan 
      0.980         nan 
    148.800         nan 
      5.490         nan 
      0.956         nan 
 
 Nor10  4738.0 
     19.182         nan 
    324.594         nan 
      0.986         nan 
    183.700         nan 
      5.515         nan 
      0.956         nan 
 
 Nor10  7102.0 
     21.330         nan 
    325.628         nan 
      0.989         nan 
    213.200         nan 
      5.517         nan 
      0.956         nan 
 
 Nor10  9466.0 
     22.831         nan 
    337.963         nan 
      0.992         nan 
    232.200         nan 
      5.526         nan 
      0.956         nan 
 
 Nor10  11830.0 
     24.245         nan 
    369.215         nan 
      0.993         nan 
    253.700         nan 
      5.536         nan 
      0.956         nan 
 
 Nor10  14194.0 
     25.496         nan 
    372.761         nan 
      0.994         nan 
    271.200         nan 
      5.543         nan 
      0.956         nan 
 
 Nor10  16558.0 
     26.057         nan 
    381.527         nan 
      0.995         nan 
    283.700         nan 
      5.546         nan 
      0.956         nan 
 
 Nor10  18922.0 
     27.035         nan 
    397.222         nan 
      0.995         nan 
    294.600         nan 
      5.545         nan 
      0.956         nan 
 
 Nor10  21286.0 
     27.878         nan 
    398.324         nan 
      0.996         nan 
    306.400         nan 
      5.550         nan 
      0.956         nan 
 
 Nor10  23650.0 
     28.413         nan 
    400.312         nan 
      0.996         nan 
    315.000         nan 
      5.549         nan 
      0.956         nan 
 
 Nor11  10.0 
      2.629         nan 
     38.000         nan 
      0.120         nan 
      9.400         nan 
      3.202         nan 
      0.888         nan 
 
 Nor11  2374.0 
     15.064         nan 
    173.511         nan 
      0.987         nan 
    119.100         nan 
      5.517         nan 
      0.958         nan 
 
 Nor11  4738.0 
     16.788         nan 
    229.325         nan 
      0.991         nan 
    145.500         nan 
      5.538         nan 
      0.959         nan 
 
 Nor11  7102.0 
     17.647         nan 
    239.539         nan 
      0.993         nan 
    159.500         nan 
      5.534         nan 
      0.958         nan 
 
 Nor11  9466.0 
     19.127         nan 
    252.506         nan 
      0.994         nan 
    176.400         nan 
      5.545         nan 
      0.958         nan 
 
 Nor11  11830.0 
     20.011         nan 
    274.960         nan 
      0.995         nan 
    191.400         nan 
      5.558         nan 
      0.959         nan 
 
 Nor11  14194.0 
     20.544         nan 
    274.572         nan 
      0.996         nan 
    199.200         nan 
      5.553         nan 
      0.958         nan 
 
 Nor11  16558.0 
     21.143         nan 
    280.582         nan 
      0.996         nan 
    209.000         nan 
      5.556         nan 
      0.958         nan 
 
 Nor11  18922.0 
     21.807         nan 
    276.018         nan 
      0.997         nan 
    217.800         nan 
      5.557         nan 
      0.958         nan 
 
 Nor11  21286.0 
     22.134         nan 
    280.261         nan 
      0.997         nan 
    223.700         nan 
      5.559         nan 
      0.958         nan 
 
 Nor11  23650.0 
     22.581         nan 
    284.171         nan 
      0.997         nan 
    230.000         nan 
      5.560         nan 
      0.958         nan 
 
 Nor12  10.0 
      2.581         nan 
     22.150         nan 
      0.300         nan 
      8.300         nan 
      2.938         nan 
      0.852         nan 
 
 Nor12  2374.0 
     13.068         nan 
    168.062         nan 
      0.984         nan 
     96.800         nan 
      5.073         nan 
      0.957         nan 
 
 Nor12  4738.0 
     15.722         nan 
    211.274         nan 
      0.989         nan 
    125.800         nan 
      5.096         nan 
      0.957         nan 
 
 Nor12  7102.0 
     17.362         nan 
    253.657         nan 
      0.991         nan 
    150.400         nan 
      5.108         nan 
      0.957         nan 
 
 Nor12  9466.0 
     19.072         nan 
    292.466         nan 
      0.992         nan 
    171.500         nan 
      5.118         nan 
      0.958         nan 
 
 Nor12  11830.0 
     20.433         nan 
    291.290         nan 
      0.994         nan 
    186.800         nan 
      5.121         nan 
      0.958         nan 
 
 Nor12  14194.0 
     21.638         nan 
    305.575         nan 
      0.994         nan 
    203.100         nan 
      5.130         nan 
      0.958         nan 
 
 Nor12  16558.0 
     22.378         nan 
    319.868         nan 
      0.995         nan 
    216.500         nan 
      5.133         nan 
      0.958         nan 
 
 Nor12  18922.0 
     23.146         nan 
    321.048         nan 
      0.996         nan 
    226.200         nan 
      5.133         nan 
      0.958         nan 
 
 Nor12  21286.0 
     23.998         nan 
    327.324         nan 
      0.996         nan 
    237.100         nan 
      5.133         nan 
      0.958         nan 
 
 Nor12  23650.0 
     24.453         nan 
    317.625         nan 
      0.996         nan 
    245.000         nan 
      5.135         nan 
      0.958         nan 
 
 Nor13  10.0 
      2.618         nan 
     21.700         nan 
      0.300         nan 
      8.400         nan 
      2.987         nan 
      0.864         nan 
 
 Nor13  2374.0 
     12.388         nan 
    168.843         nan 
      0.987         nan 
     99.200         nan 
      5.284         nan 
      0.956         nan 
 
 Nor13  4738.0 
     14.354         nan 
    234.290         nan 
      0.990         nan 
    126.500         nan 
      5.295         nan 
      0.955         nan 
 
 Nor13  7102.0 
     16.084         nan 
    247.800         nan 
      0.992         nan 
    149.300         nan 
      5.315         nan 
      0.956         nan 
 
 Nor13  9466.0 
     17.203         nan 
    282.789         nan 
      0.993         nan 
    167.300         nan 
      5.310         nan 
      0.955         nan 
 
 Nor13  11830.0 
     18.231         nan 
    300.912         nan 
      0.994         nan 
    183.600         nan 
      5.319         nan 
      0.955         nan 
 
 Nor13  14194.0 
     19.128         nan 
    298.874         nan 
      0.995         nan 
    196.000         nan 
      5.324         nan 
      0.956         nan 
 
 Nor13  16558.0 
     19.592         nan 
    315.441         nan 
      0.995         nan 
    206.900         nan 
      5.323         nan 
      0.955         nan 
 
 Nor13  18922.0 
     20.674         nan 
    317.514         nan 
      0.996         nan 
    219.200         nan 
      5.326         nan 
      0.956         nan 
 
 Nor13  21286.0 
     21.234         nan 
    322.514         nan 
      0.996         nan 
    228.600         nan 
      5.328         nan 
      0.956         nan 
 
 Nor13  23650.0 
     21.878         nan 
    331.947         nan 
      0.996         nan 
    238.000         nan 
      5.327         nan 
      0.955         nan 
 
 Nor14  10.0 
      2.490         nan 
     20.150         nan 
      0.460         nan 
      6.500         nan 
      2.333         nan 
      0.732         nan 
 
 Nor14  2374.0 
     12.525         nan 
    191.463         nan 
      0.984         nan 
     90.100         nan 
      3.696         nan 
      0.751         nan 
 
 Nor14  4738.0 
     14.999         nan 
    205.990         nan 
      0.989         nan 
    122.800         nan 
      3.743         nan 
      0.753         nan 
 
 Nor14  7102.0 
     17.769         nan 
    263.825         nan 
      0.990         nan 
    150.700         nan 
      3.771         nan 
      0.755         nan 
 
 Nor14  9466.0 
     19.091         nan 
    252.677         nan 
      0.993         nan 
    167.800         nan 
      3.770         nan 
      0.755         nan 
 
 Nor14  11830.0 
     20.291         nan 
    267.931         nan 
      0.994         nan 
    182.500         nan 
      3.761         nan 
      0.753         nan 
 
 Nor14  14194.0 
     21.699         nan 
    292.751         nan 
      0.994         nan 
    201.200         nan 
      3.770         nan 
      0.754         nan 
 
 Nor14  16558.0 
     22.234         nan 
    294.612         nan 
      0.995         nan 
    210.700         nan 
      3.779         nan 
      0.755         nan 
 
 Nor14  18922.0 
     23.163         nan 
    304.791         nan 
      0.996         nan 
    221.000         nan 
      3.777         nan 
      0.755         nan 
 
 Nor14  21286.0 
     23.947         nan 
    315.418         nan 
      0.996         nan 
    232.400         nan 
      3.776         nan 
      0.754         nan 
 
 Nor14  23650.0 
     24.495         nan 
    321.850         nan 
      0.997         nan 
    238.900         nan 
      3.776         nan 
      0.754         nan 
 
 Nor15  10.0 
      2.225         nan 
     11.650         nan 
      0.580         nan 
      5.300         nan 
      1.876         nan 
      0.618         nan 
 
 Nor15  2374.0 
     11.068         nan 
    121.797         nan 
      0.988         nan 
     77.900         nan 
      3.390         nan 
      0.705         nan 
 
 Nor15  4738.0 
     13.232         nan 
    166.615         nan 
      0.991         nan 
    102.300         nan 
      3.410         nan 
      0.705         nan 
 
 Nor15  7102.0 
     14.380         nan 
    194.655         nan 
      0.993         nan 
    118.400         nan 
      3.418         nan 
      0.706         nan 
 
 Nor15  9466.0 
     15.907         nan 
    211.584         nan 
      0.994         nan 
    135.400         nan 
      3.418         nan 
      0.704         nan 
 
 Nor15  11830.0 
     17.017         nan 
    237.429         nan 
      0.995         nan 
    146.600         nan 
      3.422         nan 
      0.705         nan 
 
 Nor15  14194.0 
     17.931         nan 
    236.412         nan 
      0.996         nan 
    159.300         nan 
      3.427         nan 
      0.705         nan 
 
 Nor15  16558.0 
     18.409         nan 
    243.419         nan 
      0.996         nan 
    168.600         nan 
      3.425         nan 
      0.704         nan 
 
 Nor15  18922.0 
     19.068         nan 
    255.342         nan 
      0.997         nan 
    175.500         nan 
      3.428         nan 
      0.705         nan 
 
 Nor15  21286.0 
     19.509         nan 
    260.020         nan 
      0.997         nan 
    182.100         nan 
      3.431         nan 
      0.705         nan 
 
 Nor15  23650.0 
     20.283         nan 
    273.094         nan 
      0.997         nan 
    190.000         nan 
      3.431         nan 
      0.705         nan 
 
 Nor16  10.0 
      2.636         nan 
     18.900         nan 
      0.350         nan 
      8.100         nan 
      2.919         nan 
      0.856         nan 
 
 Nor16  2374.0 
     11.718         nan 
    159.141         nan 
      0.986         nan 
     83.900         nan 
      4.946         nan 
      0.952         nan 
 
 Nor16  4738.0 
     14.352         nan 
    180.640         nan 
      0.991         nan 
    108.000         nan 
      4.951         nan 
      0.951         nan 
 
 Nor16  7102.0 
     15.929         nan 
    209.789         nan 
      0.993         nan 
    129.900         nan 
      4.965         nan 
      0.952         nan 
 
 Nor16  9466.0 
     17.029         nan 
    217.679         nan 
      0.994         nan 
    145.000         nan 
      4.969         nan 
      0.951         nan 
 
 Nor16  11830.0 
     17.984         nan 
    223.883         nan 
      0.995         nan 
    157.200         nan 
      4.974         nan 
      0.951         nan 
 
 Nor16  14194.0 
     18.751         nan 
    238.551         nan 
      0.996         nan 
    166.800         nan 
      4.974         nan 
      0.952         nan 
 
 Nor16  16558.0 
     19.526         nan 
    252.124         nan 
      0.996         nan 
    177.000         nan 
      4.976         nan 
      0.951         nan 
 
 Nor16  18922.0 
     20.326         nan 
    273.030         nan 
      0.996         nan 
    186.500         nan 
      4.976         nan 
      0.951         nan 
 
 Nor16  21286.0 
     20.828         nan 
    268.794         nan 
      0.997         nan 
    191.600         nan 
      4.977         nan 
      0.951         nan 
 
 Nor16  23650.0 
     21.516         nan 
    276.241         nan 
      0.997         nan 
    200.000         nan 
      4.979         nan 
      0.952         nan 
 
 Nor17  10.0 
      1.932         nan 
     19.220         nan 
      0.370         nan 
      8.000         nan 
      2.899         nan 
      0.854         nan 
 
 Nor17  2374.0 
     11.742         nan 
    137.441         nan 
      0.990         nan 
    107.500         nan 
      5.065         nan 
      0.941         nan 
 
 Nor17  4738.0 
     13.533         nan 
    171.868         nan 
      0.993         nan 
    129.200         nan 
      5.075         nan 
      0.941         nan 
 
 Nor17  7102.0 
     14.362         nan 
    186.963         nan 
      0.995         nan 
    139.700         nan 
      5.088         nan 
      0.941         nan 
 
 Nor17  9466.0 
     14.884         nan 
    193.611         nan 
      0.996         nan 
    148.900         nan 
      5.082         nan 
      0.941         nan 
 
 Nor17  11830.0 
     15.756         nan 
    211.692         nan 
      0.997         nan 
    159.600         nan 
      5.091         nan 
      0.941         nan 
 
 Nor17  14194.0 
     16.361         nan 
    212.923         nan 
      0.997         nan 
    167.400         nan 
      5.091         nan 
      0.941         nan 
 
 Nor17  16558.0 
     16.767         nan 
    223.976         nan 
      0.997         nan 
    170.700         nan 
      5.087         nan 
      0.941         nan 
 
 Nor17  18922.0 
     17.248         nan 
    225.864         nan 
      0.998         nan 
    177.700         nan 
      5.086         nan 
      0.941         nan 
 
 Nor17  21286.0 
     17.589         nan 
    224.104         nan 
      0.998         nan 
    182.600         nan 
      5.089         nan 
      0.941         nan 
 
 Nor17  23650.0 
     17.951         nan 
    233.493         nan 
      0.998         nan 
    188.000         nan 
      5.090         nan 
      0.941         nan 
 
 Nor18  10.0 
      1.671         nan 
     10.900         nan 
      0.610         nan 
      5.100         nan 
      1.840         nan 
      0.620         nan 
 
 Nor18  2374.0 
     12.976         nan 
    162.425         nan 
      0.982         nan 
    118.300         nan 
      3.475         nan 
      0.722         nan 
 
 Nor18  4738.0 
     15.369         nan 
    191.793         nan 
      0.991         nan 
    148.100         nan 
      3.485         nan 
      0.720         nan 
 
 Nor18  7102.0 
     16.148         nan 
    188.128         nan 
      0.995         nan 
    160.100         nan 
      3.471         nan 
      0.716         nan 
 
 Nor18  9466.0 
     16.894         nan 
    212.965         nan 
      0.996         nan 
    175.000         nan 
      3.508         nan 
      0.720         nan 
 
 Nor18  11830.0 
     17.679         nan 
    224.812         nan 
      0.997         nan 
    185.000         nan 
      3.513         nan 
      0.722         nan 
 
 Nor18  14194.0 
     17.906         nan 
    220.125         nan 
      0.997         nan 
    189.000         nan 
      3.508         nan 
      0.720         nan 
 
 Nor18  16558.0 
     18.337         nan 
    223.136         nan 
      0.998         nan 
    194.800         nan 
      3.507         nan 
      0.720         nan 
 
 Nor18  18922.0 
     18.510         nan 
    223.835         nan 
      0.998         nan 
    199.600         nan 
      3.510         nan 
      0.720         nan 
 
 Nor18  21286.0 
     18.693         nan 
    224.879         nan 
      0.998         nan 
    203.500         nan 
      3.511         nan 
      0.719         nan 
 
 Nor18  23650.0 
     18.932         nan 
    226.375         nan 
      0.999         nan 
    207.000         nan 
      3.513         nan 
      0.720         nan 
 
 Nor19  10.0 
      2.904         nan 
     23.850         nan 
      0.290         nan 
      8.500         nan 
      3.014         nan 
      0.868         nan 
 
 Nor19  2374.0 
     11.735         nan 
    102.134         nan 
      0.996         nan 
     88.000         nan 
      5.522         nan 
      0.967         nan 
 
 Nor19  4738.0 
     12.285         nan 
    124.150         nan 
      0.998         nan 
     93.100         nan 
      5.533         nan 
      0.967         nan 
 
 Nor19  7102.0 
     13.098         nan 
    163.945         nan 
      0.998         nan 
    100.700         nan 
      5.536         nan 
      0.967         nan 
 
 Nor19  9466.0 
     13.740         nan 
    155.435         nan 
      0.998         nan 
    106.800         nan 
      5.541         nan 
      0.967         nan 
 
 Nor19  11830.0 
     14.029         nan 
    176.993         nan 
      0.998         nan 
    109.700         nan 
      5.542         nan 
      0.967         nan 
 
 Nor19  14194.0 
     14.409         nan 
    176.835         nan 
      0.998         nan 
    113.200         nan 
      5.539         nan 
      0.967         nan 
 
 Nor19  16558.0 
     14.722         nan 
    163.663         nan 
      0.998         nan 
    117.700         nan 
      5.542         nan 
      0.967         nan 
 
 Nor19  18922.0 
     15.125         nan 
    165.813         nan 
      0.999         nan 
    120.300         nan 
      5.543         nan 
      0.967         nan 
 
 Nor19  21286.0 
     15.495         nan 
    171.842         nan 
      0.999         nan 
    124.700         nan 
      5.545         nan 
      0.967         nan 
 
 Nor19  23650.0 
     15.736         nan 
    170.273         nan 
      0.999         nan 
    128.000         nan 
      5.545         nan 
      0.967         nan 
 
 Nor20  10.0 
      2.580         nan 
     21.550         nan 
      0.340         nan 
      8.100         nan 
      2.907         nan 
      0.852         nan 
 
 Nor20  2374.0 
     20.160         nan 
    271.577         nan 
      0.970         nan 
    190.100         nan 
      5.418         nan 
      0.943         nan 
 
 Nor20  4738.0 
     23.763         nan 
    341.871         nan 
      0.983         nan 
    243.200         nan 
      5.479         nan 
      0.944         nan 
 
 Nor20  7102.0 
     26.676         nan 
    371.928         nan 
      0.988         nan 
    279.100         nan 
      5.496         nan 
      0.944         nan 
 
 Nor20  9466.0 
     28.670         nan 
    401.389         nan 
      0.990         nan 
    305.400         nan 
      5.508         nan 
      0.944         nan 
 
 Nor20  11830.0 
     30.017         nan 
    414.036         nan 
      0.992         nan 
    326.100         nan 
      5.514         nan 
      0.944         nan 
 
 Nor20  14194.0 
     30.960         nan 
    423.870         nan 
      0.994         nan 
    338.500         nan 
      5.521         nan 
      0.945         nan 
 
 Nor20  16558.0 
     31.892         nan 
    441.782         nan 
      0.995         nan 
    354.600         nan 
      5.518         nan 
      0.944         nan 
 
 Nor20  18922.0 
     32.815         nan 
    452.054         nan 
      0.995         nan 
    367.500         nan 
      5.525         nan 
      0.945         nan 
 
 Nor20  21286.0 
     33.530         nan 
    459.771         nan 
      0.996         nan 
    378.500         nan 
      5.526         nan 
      0.945         nan 
 
 Nor20  23650.0 
     34.116         nan 
    471.694         nan 
      0.996         nan 
    388.000         nan 
      5.529         nan 
      0.945         nan 
 
 Nor21  10.0 
      2.114         nan 
     13.950         nan 
      0.530         nan 
      6.100         nan 
      2.242         nan 
      0.720         nan 
 
 Nor21  2374.0 
     17.416         nan 
    224.176         nan 
      0.978         nan 
    150.900         nan 
      4.588         nan 
      0.847         nan 
 
 Nor21  4738.0 
     19.924         nan 
    265.411         nan 
      0.988         nan 
    183.300         nan 
      4.581         nan 
      0.845         nan 
 
 Nor21  7102.0 
     22.347         nan 
    284.931         nan 
      0.991         nan 
    209.700         nan 
      4.612         nan 
      0.846         nan 
 
 Nor21  9466.0 
     23.245         nan 
    305.117         nan 
      0.993         nan 
    224.300         nan 
      4.605         nan 
      0.846         nan 
 
 Nor21  11830.0 
     24.632         nan 
    338.709         nan 
      0.994         nan 
    244.800         nan 
      4.609         nan 
      0.845         nan 
 
 Nor21  14194.0 
     25.279         nan 
    335.632         nan 
      0.995         nan 
    252.900         nan 
      4.611         nan 
      0.845         nan 
 
 Nor21  16558.0 
     26.000         nan 
    362.699         nan 
      0.995         nan 
    264.500         nan 
      4.626         nan 
      0.847         nan 
 
 Nor21  18922.0 
     26.937         nan 
    382.176         nan 
      0.996         nan 
    278.000         nan 
      4.617         nan 
      0.845         nan 
 
 Nor21  21286.0 
     27.767         nan 
    382.457         nan 
      0.996         nan 
    286.500         nan 
      4.626         nan 
      0.846         nan 
 
 Nor21  23650.0 
     28.292         nan 
    387.571         nan 
      0.997         nan 
    295.000         nan 
      4.625         nan 
      0.846         nan 
 
 
 
 
 
 
