# Supplementary material for: Characterization of Esophageal Microbiota in Patients With Esophagitis and Esophageal Squamous Cell Carcinoma
Source: Front Cell Infect Microbiol. 2021 Nov 11;11:774330. doi: 10.3389/fcimb.2021.774330 (PMC8632060; doi:10.3389/fcimb.2021.774330)
Supplement: Supplementary file 2 [file DataSheet_1.zip › 16S_V3_V4-68╕÷╤∙▒╛JZD 2020.7.21/3.Taxonomic_analysis/Barplot/Groups/bar.ALL.Class.pdf]

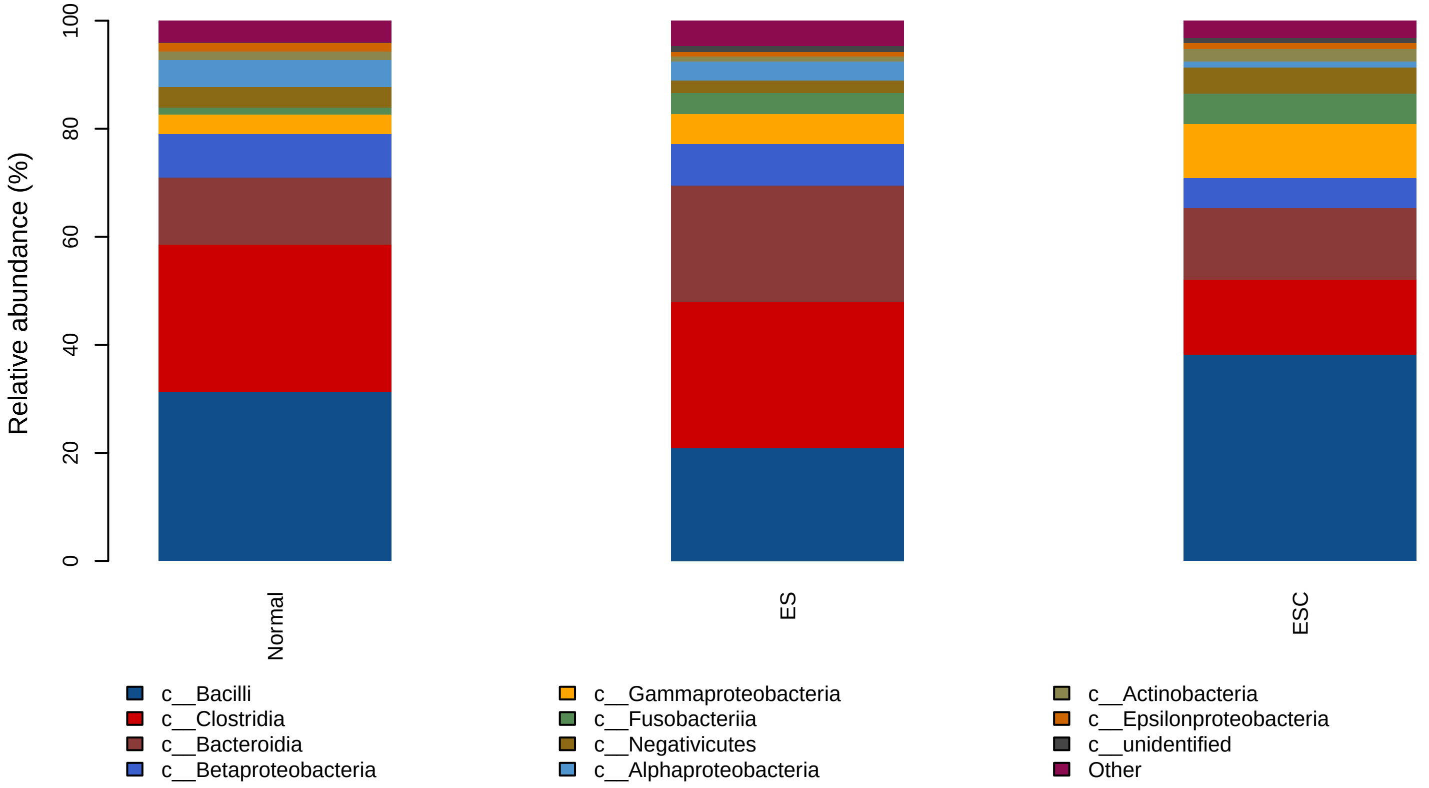
Relative abundance (%)
0
20
40
60
80
100
Normal
ES
ESC
c__Bacilli
c__Clostridia
c__Bacteroidia
c__Betaproteobacteria
c__Gammaproteobacteria
c__Fusobacteriia
c__Negativicutes
c__Alphaproteobacteria
c__Actinobacteria
c__Epsilonproteobacteria
c__unidentified
Other
