# Supplementary material for: Characterization of Esophageal Microbiota in Patients With Esophagitis and Esophageal Squamous Cell Carcinoma
Source: Front Cell Infect Microbiol. 2021 Nov 11;11:774330. doi: 10.3389/fcimb.2021.774330 (PMC8632060; doi:10.3389/fcimb.2021.774330)
Supplement: Supplementary file 2 [file DataSheet_1.zip › 16S_V3_V4-68╕÷╤∙▒╛JZD 2020.7.21/3.Taxonomic_analysis/Barplot/Groups/bar.ALL.Family.pdf]

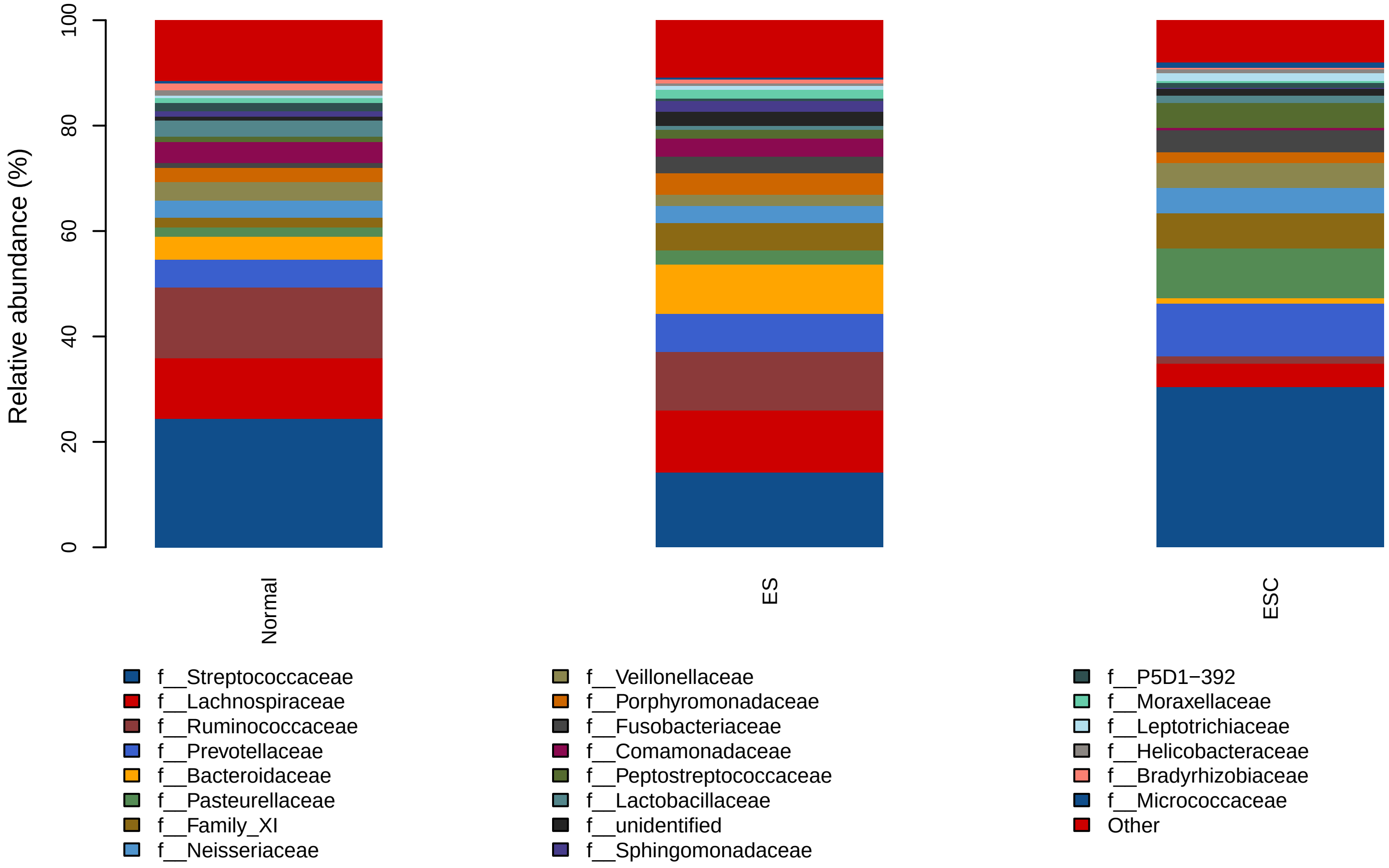

Relative abundance (%)
0
20
40
60
80
100
Normal
ES
ESC
f__Streptococcaceae
f__Lachnospiraceae
f__Ruminococcaceae
f__Prevotellaceae
f__Bacteroidaceae
f__Pasteurellaceae
f__Family_XI
f__Neisseriaceae
f__Veillonellaceae
f__Porphyromonadaceae
f__Fusobacteriaceae
f__Comamonadaceae
f__Peptostreptococcaceae
f__Lactobacillaceae
f__unidentified
f__Sphingomonadaceae
f__P5D1−392
f__Moraxellaceae
f__Leptotrichiaceae
f__Helicobacteraceae
f__Bradyrhizobiaceae
f__Micrococcaceae
Other
