# Supplementary material for: Characterization of Esophageal Microbiota in Patients With Esophagitis and Esophageal Squamous Cell Carcinoma
Source: Front Cell Infect Microbiol. 2021 Nov 11;11:774330. doi: 10.3389/fcimb.2021.774330 (PMC8632060; doi:10.3389/fcimb.2021.774330)
Supplement: Supplementary file 2 [file DataSheet_1.zip › 16S_V3_V4-68╕÷╤∙▒╛JZD 2020.7.21/3.Taxonomic_analysis/Barplot/Groups/bar.ALL.Genus.pdf]

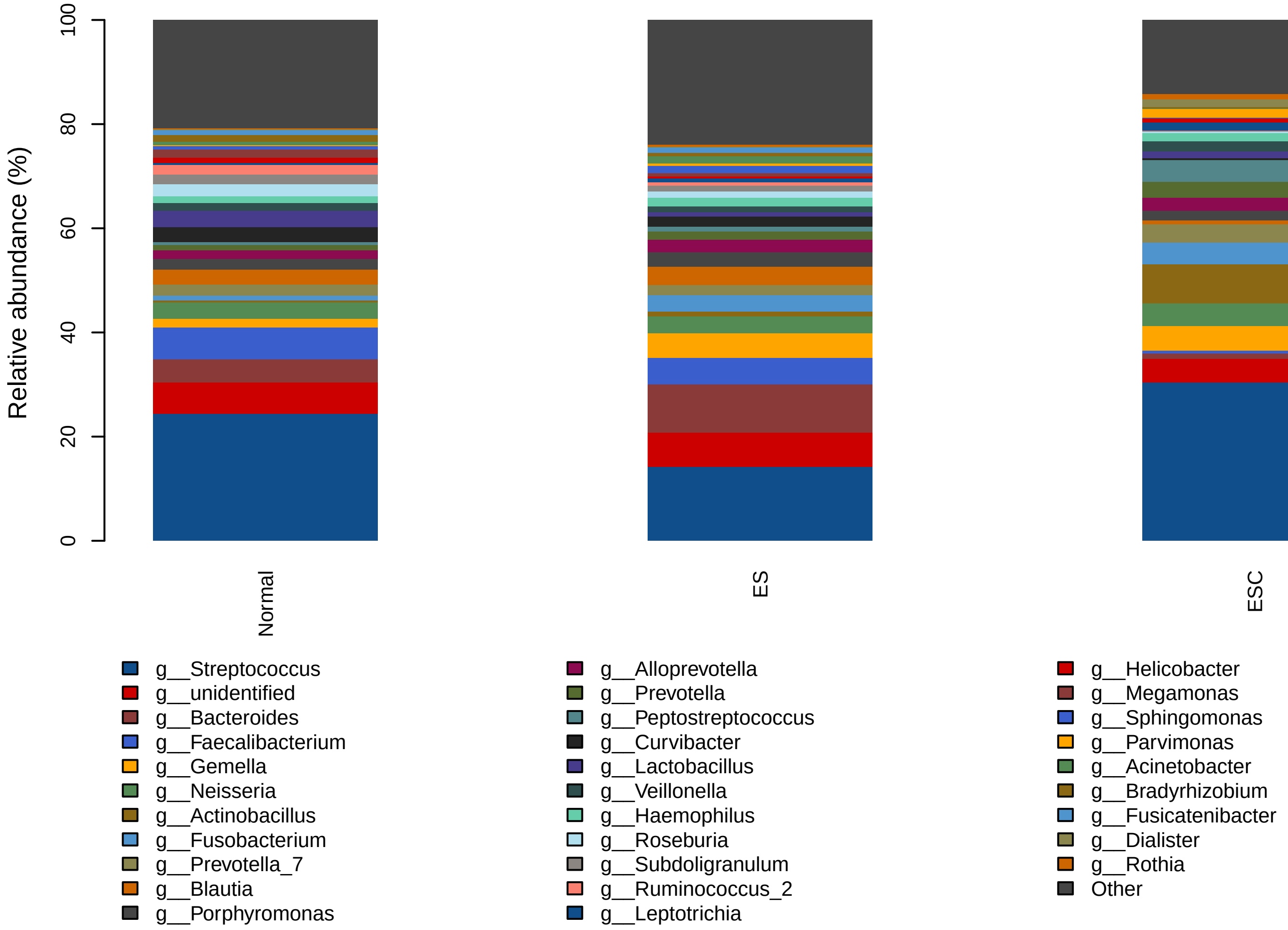
Relative abundance (%)
0
20
40
60
80
100
Normal
ES
ESC
g__Streptococcus
g__unidentified
g__Bacteroides
g__Faecalibacterium
g__Gemella
g__Neisseria
g__Actinobacillus
g__Fusobacterium
g__Prevotella_7
g__Blautia
g__Porphyromonas
g__Alloprevotella
g__Prevotella
g__Peptostreptococcus
g__Curvibacter
g__Lactobacillus
g__Veillonella
g__Haemophilus
g__Roseburia
g__Subdoligranulum
g__Ruminococcus_2
g__Leptotrichia
g__Helicobacter
g__Megamonas
g__Sphingomonas
g__Parvimonas
g__Acinetobacter
g__Bradyrhizobium
g__Fusicatenibacter
g__Dialister
g__Rothia
Other
