# Supplementary material for: Characterization of Esophageal Microbiota in Patients With Esophagitis and Esophageal Squamous Cell Carcinoma
Source: Front Cell Infect Microbiol. 2021 Nov 11;11:774330. doi: 10.3389/fcimb.2021.774330 (PMC8632060; doi:10.3389/fcimb.2021.774330)
Supplement: Supplementary file 2 [file DataSheet_1.zip › 16S_V3_V4-68╕÷╤∙▒╛JZD 2020.7.21/3.Taxonomic_analysis/Barplot/Groups/bar.ALL.Phylum.pdf]

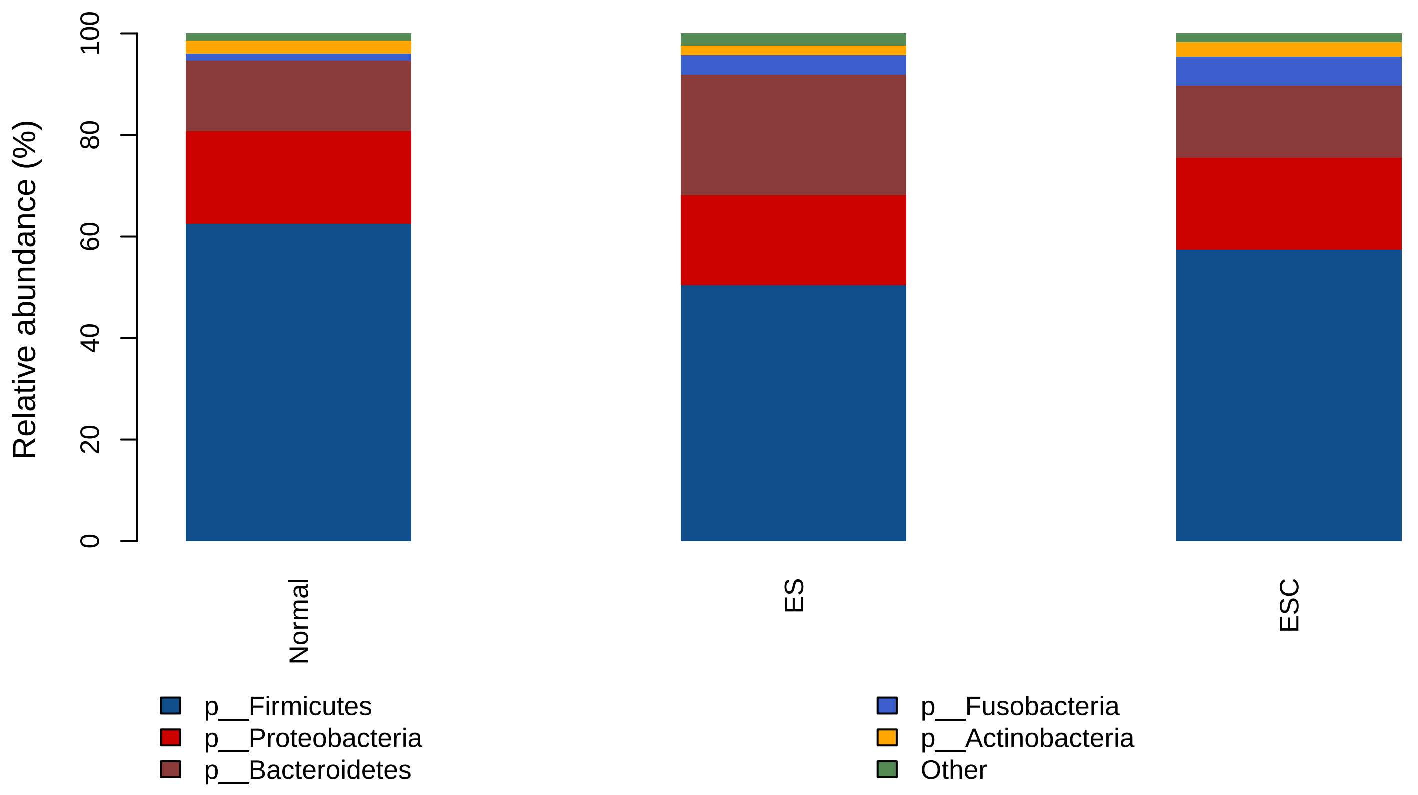

Relative abundance (%)
0
20
40
60
80
100
Normal
ES
ESC
p__Firmicutes
p__Proteobacteria
p__Bacteroidetes
p__Fusobacteria
p__Actinobacteria
Other
