# Supplementary material for: Characterization of Esophageal Microbiota in Patients With Esophagitis and Esophageal Squamous Cell Carcinoma
Source: Front Cell Infect Microbiol. 2021 Nov 11;11:774330. doi: 10.3389/fcimb.2021.774330 (PMC8632060; doi:10.3389/fcimb.2021.774330)
Supplement: Supplementary file 2 [file DataSheet_1.zip › 16S_V3_V4-68╕÷╤∙▒╛JZD 2020.7.21/3.Taxonomic_analysis/Barplot/Samples/bar.ALL.Family.pdf]

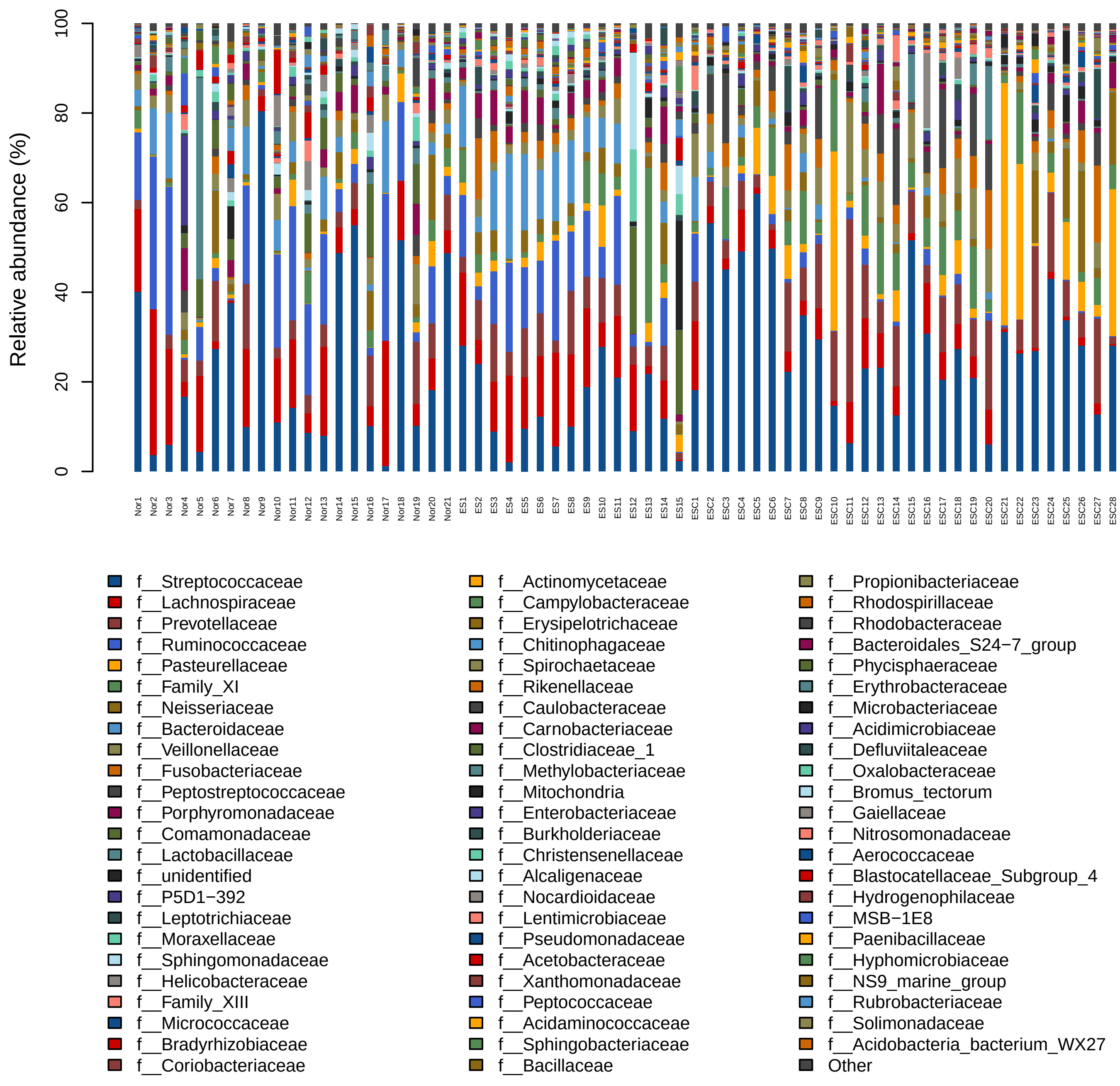

Relative abundance (%)
0
20
40
60
80
100
Nor1 Nor2 Nor3 Nor4 Nor5 Nor6 Nor7 Nor8 Nor9 Nor10 Nor11 Nor12 Nor13 Nor14 Nor15 Nor16 Nor17 Nor18 Nor19 Nor20 Nor21
ES1 ES2 ES3 ES4 ES5 ES6 ES7 ES8 ES9 ES10 ES11 ES12 ES13 ES14 ES15
ESC1 ESC2 ESC3 ESC4 ESC5 ESC6 ESC7 ESC8 ESC9 ESC10 ESC11 ESC12 ESC13 ESC14 ESC15 ESC16 ESC17 ESC18 ESC19 ESC20 ESC21 ESC22 ESC23 ESC24 ESC25 ESC26 ESC27 ESC28 ESC29 ESC30 ESC31 ESC32
f__Streptococcaceae
f__Lachnospiraceae
f__Prevotellaceae
f__Ruminococcaceae
f__Pasteurellaceae
f__Family_XI
f__Neisseriaceae
f__Bacteroidaceae
f__Veillonellaceae
f__Fusobacteriaceae
f__Peptostreptococcaceae
f__Porphyromonadaceae
f__Comamonadaceae
f__Lactobacillaceae
f__unidentified
f__P5D1−392
f__Leptotrichiaceae
f__Moraxellaceae
f__Sphingomonadaceae
f__Helicobacteraceae
f__Family_XIII
f__Micrococcaceae
f__Bradyrhizobiaceae
f__Coriobacteriaceae
f__Actinomycetaceae
f__Campylobacteraceae
f__Erysipelotrichaceae
f__Chitinophagaceae
f__Spirochaetaceae
f__Rikenellaceae
f__Caulobacteraceae
f__Carnobacteriaceae
f__Clostridiaceae_1
f__Methylobacteriaceae
f__Mitochondria
f__Enterobacteriaceae
f__Burkholderiaceae
f__Christensenellaceae
f__Alcaligenaceae
f__Nocardioidaceae
f__Lentimicrobiaceae
f__Pseudomonadaceae
f__Acetobacteraceae
f__Xanthomonadaceae
f__Peptococcaceae
f__Acidaminococcaceae
f__Sphingobacteriaceae
f__Bacillaceae
f__Propionibacteriaceae
f__Rhodospirillaceae
f__Rhodobacteraceae
f__Bacteroidales_S24−7_group
f__Phycisphaeraceae
f__Erythrobacteraceae
f__Microbacteriaceae
f__Acidimicrobiaceae
f__Defluviitaleaceae
f__Oxalobacteraceae
f__Bromus_tectorum
f__Gaiellaceae
f__Nitrosomonadaceae
f__Aerococcaceae
f__Blastocatellaceae_Subgroup_4
f__Hydrogenophilaceae
f__MSB−1E8
f__Paenibacillaceae
f__Hyphomicrobiaceae
f__NS9_marine_group
f__Rubrobacteriaceae
f__Solimonadaceae
f__Acidobacteria_bacterium_WX27
Other
