# Supplementary material for: Characterization of Esophageal Microbiota in Patients With Esophagitis and Esophageal Squamous Cell Carcinoma
Source: Front Cell Infect Microbiol. 2021 Nov 11;11:774330. doi: 10.3389/fcimb.2021.774330 (PMC8632060; doi:10.3389/fcimb.2021.774330)
Supplement: Supplementary file 2 [file DataSheet_1.zip › 16S_V3_V4-68╕÷╤∙▒╛JZD 2020.7.21/3.Taxonomic_analysis/Barplot/Samples/bar.ALL.Genus.pdf]

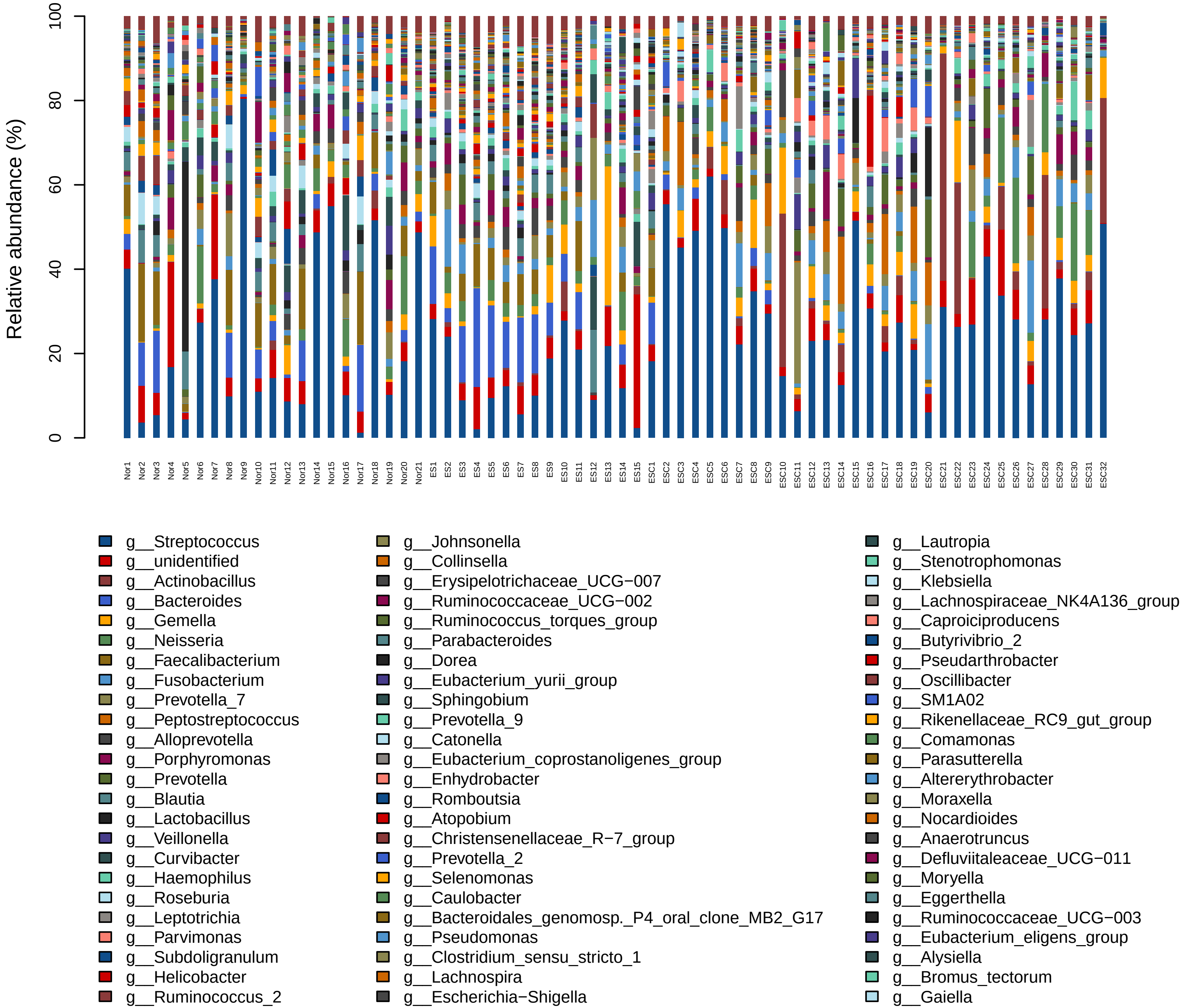

Relative abundance (%)
0
20
40
60
80
100
Nor1 Nor2 Nor3 Nor4 Nor5 Nor6 Nor7 Nor8 Nor9 Nor10 Nor11 Nor12 Nor13 Nor14 Nor15 Nor16 Nor17 Nor18 Nor19 Nor20 Nor21
ES1 ES2 ES3 ES4 ES5 ES6 ES7 ES8 ES9 ES10 ES11 ES12 ES13 ES14 ES15
ESC1 ESC2 ESC3 ESC4 ESC5 ESC6 ESC7 ESC8 ESC9 ESC10 ESC11 ESC12 ESC13 ESC14 ESC15 ESC16 ESC17 ESC18 ESC19 ESC20 ESC21 ESC22 ESC23 ESC24 ESC25 ESC26 ESC27 ESC28 ESC29 ESC30 ESC31 ESC32
g__Streptococcus
g__unidentified
g__Actinobacillus
g__Bacteroides
g__Gemella
g__Neisseria
g__Faecalibacterium
g__Fusobacterium
g__Prevotella_7
g__Peptostreptococcus
g__Alloprevotella
g__Porphyromonas
g__Prevotella
g__Blautia
g__Lactobacillus
g__Veillonella
g__Curvibacter
g__Haemophilus
g__Roseburia
g__Leptotrichia
g__Parvimonas
g__Subdoligranulum
g__Helicobacter
g__Ruminococcus_2
g__Johnsonella
g__Collinsella
g__Erysipelotrichaceae_UCG−007
g__Ruminococcaceae_UCG−002
g__Ruminococcus_torques_group
g__Parabacteroides
g__Dorea
g__Eubacterium_yurii_group
g__Sphingobium
g__Prevotella_9
g__Catonella
g__Eubacterium_coprostanoligenes_group
g__Enhydrobacter
g__Romboutsia
g__Atopobium
g__Christensenellaceae_R−7_group
g__Prevotella_2
g__Selenomonas
g__Caulobacter
g__Bacteroidales_genomosp._P4_oral_clone_MB2_G17
g__Pseudomonas
g__Clostridium_sensu_stricto_1
g__Lachnospira
g__Escherichia−Shigella
g__Lautropia
g__Stenotrophomonas
g__Klebsiella
g__Lachnospiraceae_NK4A136_group
g__Caproiciproducens
g__Butyrivibrio_2
g__Pseudarthrobacter
g__Oscillibacter
g__SM1A02
g__Rikenellaceae_RC9_gut_group
g__Comamonas
g__Parasutterella
g__Altererythrobacter
g__Moraxella
g__Nocardioides
g__Anaerotruncus
g__Defluviitaleaceae_UCG−011
g__Moryella
g__Eggerthella
g__Ruminococcaceae_UCG−003
g__Eubacterium_eligens_group
g__Alysiella
g__Bromus_tectorum
g__Gaiella
