# Supplementary material for: Characterization of Esophageal Microbiota in Patients With Esophagitis and Esophageal Squamous Cell Carcinoma
Source: Front Cell Infect Microbiol. 2021 Nov 11;11:774330. doi: 10.3389/fcimb.2021.774330 (PMC8632060; doi:10.3389/fcimb.2021.774330)
Supplement: Supplementary file 2 [file DataSheet_1.zip › 16S_V3_V4-68╕÷╤∙▒╛JZD 2020.7.21/3.Taxonomic_analysis/Core_microbiome/core_otu_size.pdf]

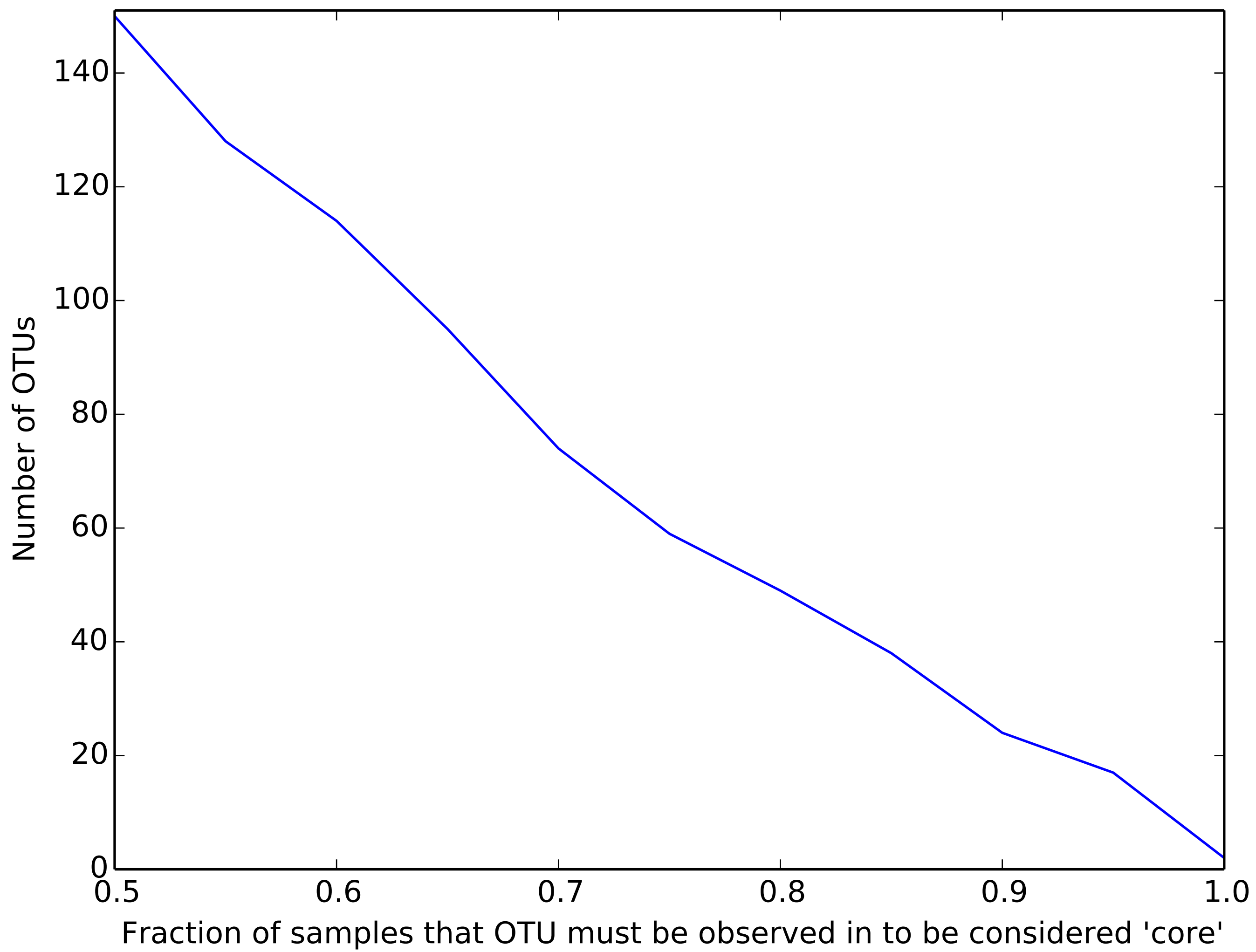
Number of OTUs
0
20
40
60
80
100
120
140
0.5
0.6
0.7
0.8
0.9
1.0
Fraction of samples that OTU must be observed in to be considered 'core'
