# Supplementary material for: Characterization of Esophageal Microbiota in Patients With Esophagitis and Esophageal Squamous Cell Carcinoma
Source: Front Cell Infect Microbiol. 2021 Nov 11;11:774330. doi: 10.3389/fcimb.2021.774330 (PMC8632060; doi:10.3389/fcimb.2021.774330)
Supplement: Supplementary file 2 [file DataSheet_1.zip › 16S_V3_V4-68╕÷╤∙▒╛JZD 2020.7.21/3.Taxonomic_analysis/Genus_phylogeny/phylogeny.pdf]

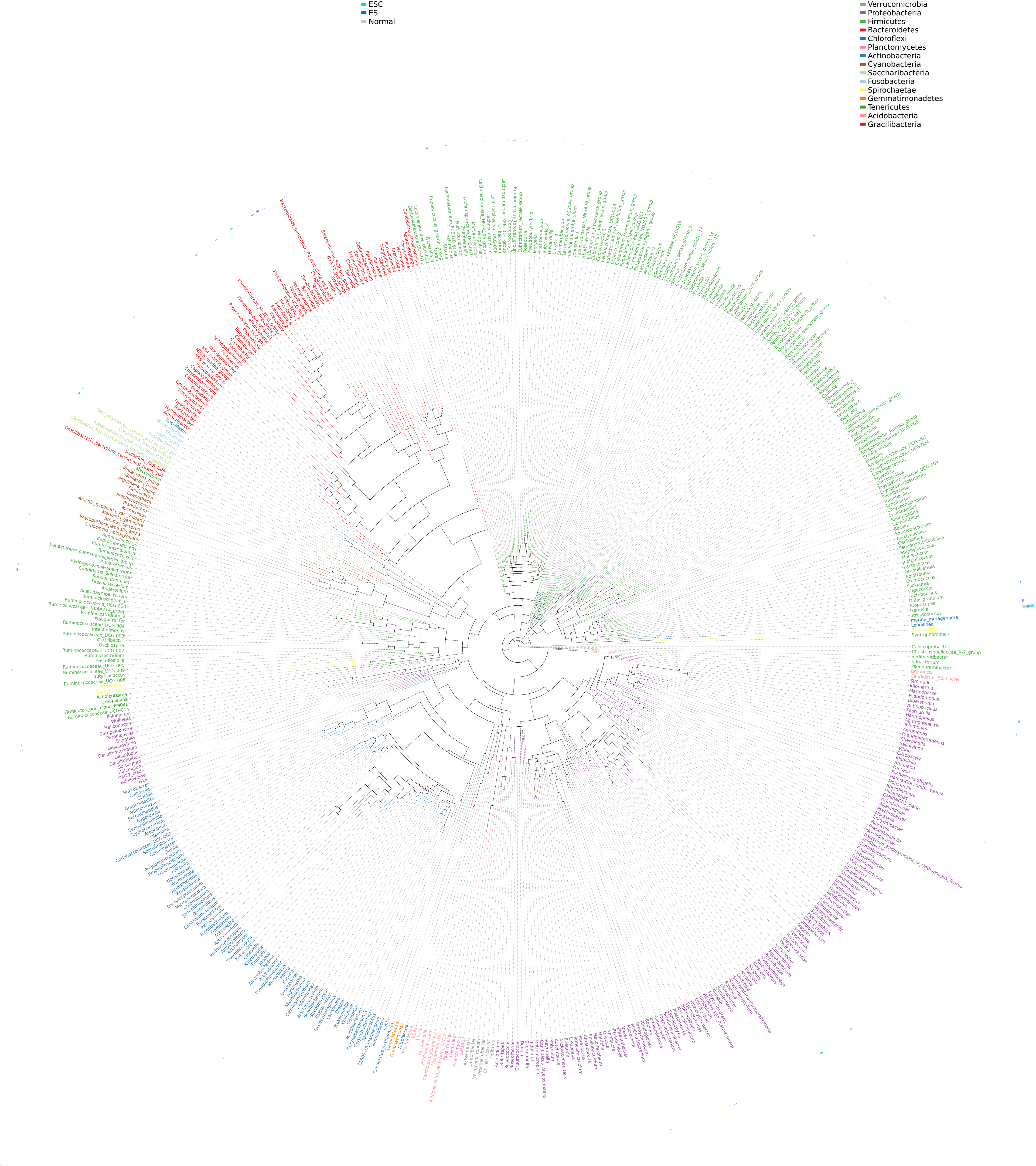
ESC
ES
Normal
Verrucomicrobia
Proteobacteria
Firmicutes
Bacteroidetes
Chloroflexi
Planctomycetes
Actinobacteria
Cyanobacteria
Saccharibacteria
Fusobacteria
Spirochaetae
Gemmatimonadetes
Tenericutes
Acidobacteria
Gracilibacteria
