# Supplementary material for: Characterization of Esophageal Microbiota in Patients With Esophagitis and Esophageal Squamous Cell Carcinoma
Source: Front Cell Infect Microbiol. 2021 Nov 11;11:774330. doi: 10.3389/fcimb.2021.774330 (PMC8632060; doi:10.3389/fcimb.2021.774330)
Supplement: Supplementary file 2 [file DataSheet_1.zip › 16S_V3_V4-68╕÷╤∙▒╛JZD 2020.7.21/3.Taxonomic_analysis/Heatmap/Level_taxonomy/Groups/Topclass.pdf]

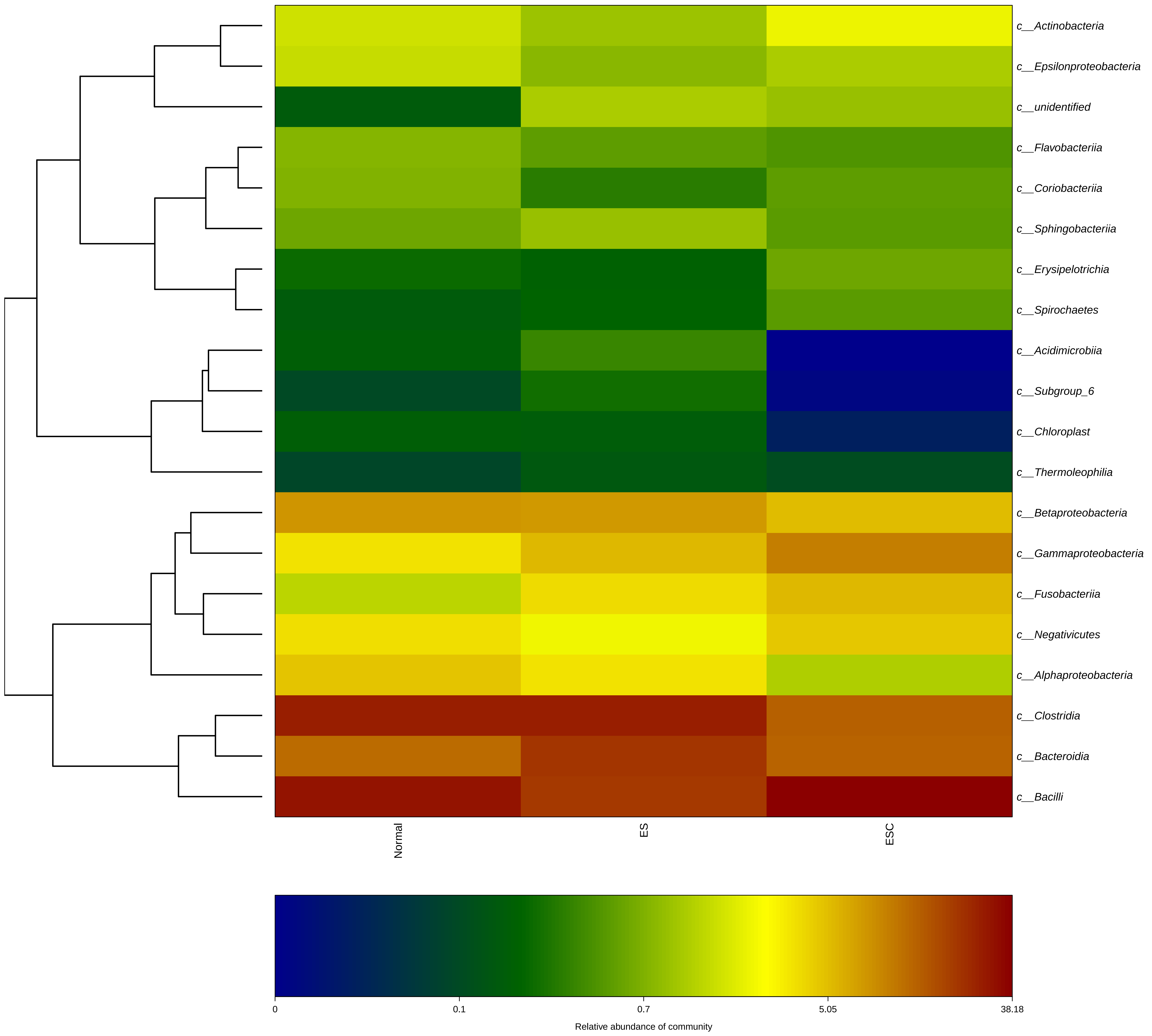

c__Actinobacteria
c__Epsilonproteobacteria
c__unidentified
c__Flavobacteriia
c__Coriobacteriia
c__Sphingobacteriia
c__Erysipelotrichia
c__Spirochaetes
c__Acidimicrobiia
c__Subgroup_6
c__Chloroplast
c__Thermoleophilia
c__Betaproteobacteria
c__Gammaproteobacteria
c__Fusobacteriia
c__Negativicutes
c__Alphaproteobacteria
c__Clostridia
c__Bacteroidia
c__Bacilli
Normal
ES
ESC
0
0.1
0.7
5.05
38.18
Relative abundance of community
