# Supplementary material for: Characterization of Esophageal Microbiota in Patients With Esophagitis and Esophageal Squamous Cell Carcinoma
Source: Front Cell Infect Microbiol. 2021 Nov 11;11:774330. doi: 10.3389/fcimb.2021.774330 (PMC8632060; doi:10.3389/fcimb.2021.774330)
Supplement: Supplementary file 2 [file DataSheet_1.zip › 16S_V3_V4-68╕÷╤∙▒╛JZD 2020.7.21/3.Taxonomic_analysis/Heatmap/Level_taxonomy/Groups/Toporder.pdf]

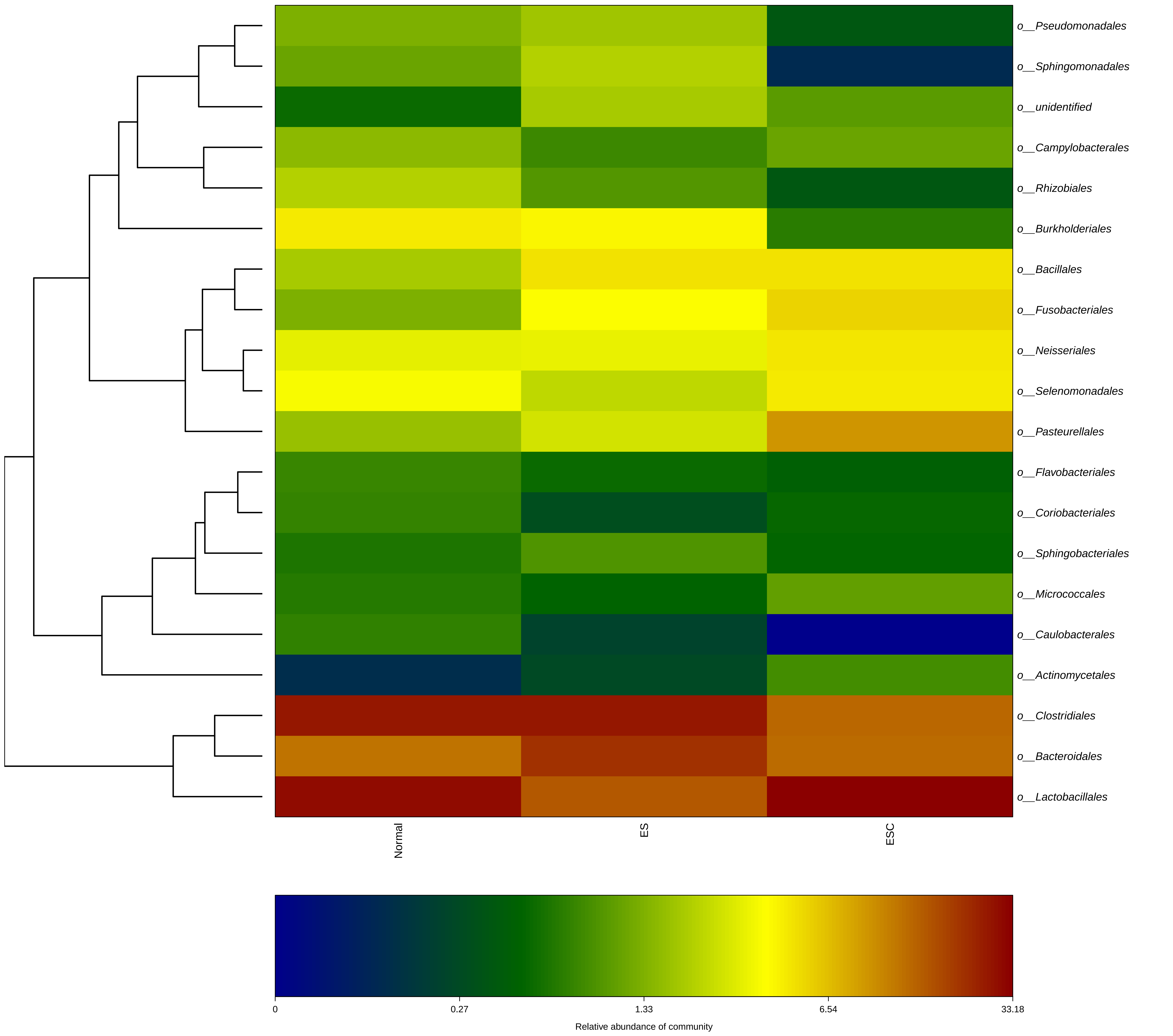
o__Pseudomonadales
o__Sphingomonadales
o__unidentified
o__Campylobacterales
o__Rhizobiales
o__Burkholderiales
o__Bacillales
o__Fusobacteriales
o__Neisseriales
o__Selenomonadales
o__Pasteurellales
o__Flavobacteriales
o__Coriobacteriales
o__Sphingobacteriales
o__Micrococcales
o__Caulobacterales
o__Actinomycetales
o__Clostridiales
o__Bacteroidales
o__Lactobacillales
Normal
ES
ESC
0
0.27
1.33
6.54
33.18
Relative abundance of community
