# Supplementary material for: Characterization of Esophageal Microbiota in Patients With Esophagitis and Esophageal Squamous Cell Carcinoma
Source: Front Cell Infect Microbiol. 2021 Nov 11;11:774330. doi: 10.3389/fcimb.2021.774330 (PMC8632060; doi:10.3389/fcimb.2021.774330)
Supplement: Supplementary file 2 [file DataSheet_1.zip › 16S_V3_V4-68╕÷╤∙▒╛JZD 2020.7.21/3.Taxonomic_analysis/Heatmap/Level_taxonomy/Groups/Topphylum.pdf]

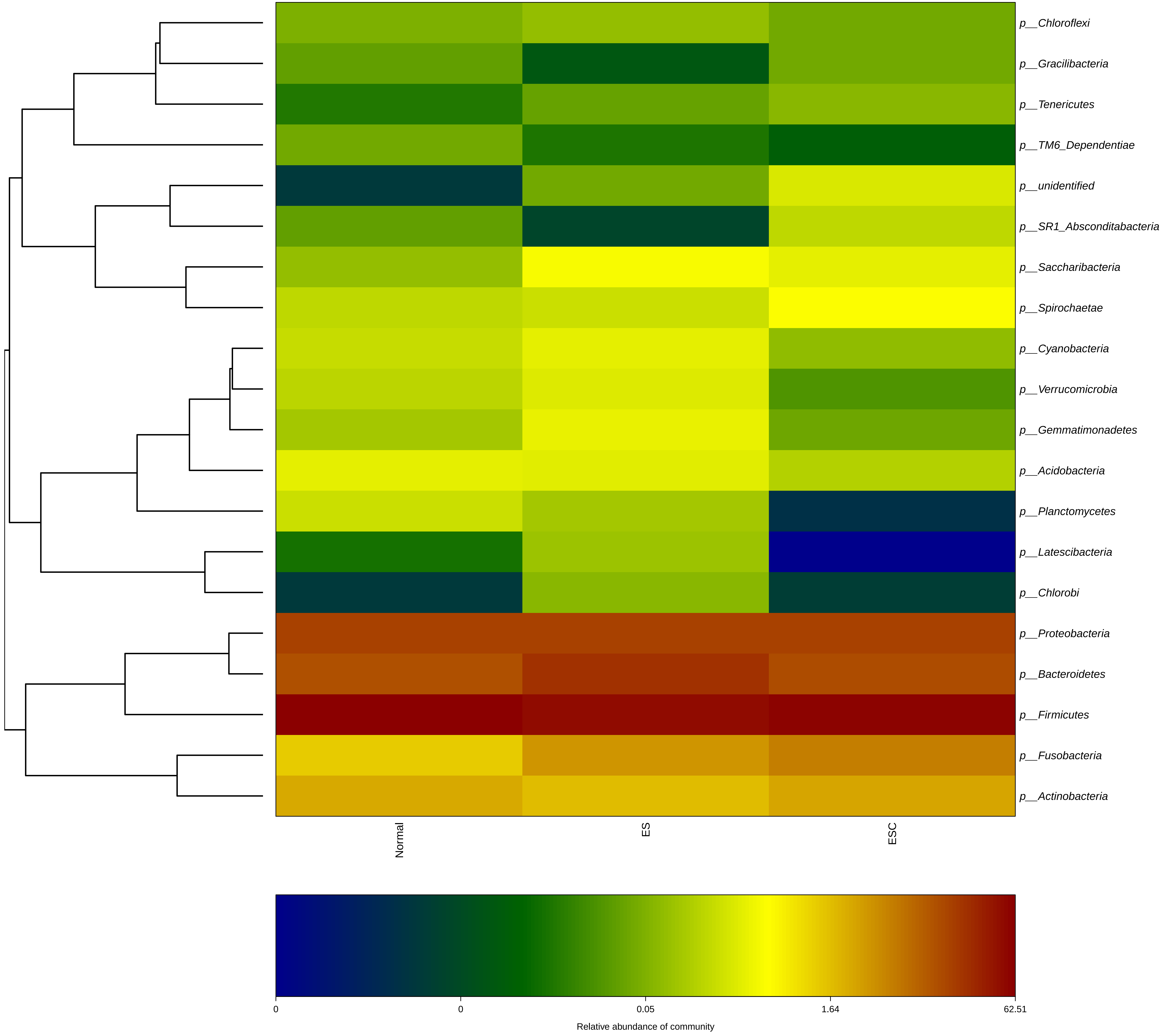
p__Chloroflexi
p__Gracilibacteria
p__Tenericutes
p__TM6_Dependentiae
p__unidentified
p__SR1_Absconditabacteria
p__Saccharibacteria
p__Spirochaetae
p__Cyanobacteria
p__Verrucomicrobia
p__Gemmatimonadetes
p__Acidobacteria
p__Planctomycetes
p__Latescibacteria
p__Chlorobi
p__Proteobacteria
p__Bacteroidetes
p__Firmicutes
p__Fusobacteria
p__Actinobacteria
Normal
ES
ESC
0
0
0.05
1.64
62.51
Relative abundance of community
