# Supplementary material for: Characterization of Esophageal Microbiota in Patients With Esophagitis and Esophageal Squamous Cell Carcinoma
Source: Front Cell Infect Microbiol. 2021 Nov 11;11:774330. doi: 10.3389/fcimb.2021.774330 (PMC8632060; doi:10.3389/fcimb.2021.774330)
Supplement: Supplementary file 2 [file DataSheet_1.zip › 16S_V3_V4-68╕÷╤∙▒╛JZD 2020.7.21/3.Taxonomic_analysis/Heatmap/Level_taxonomy/Samples/Topfamily.pdf]

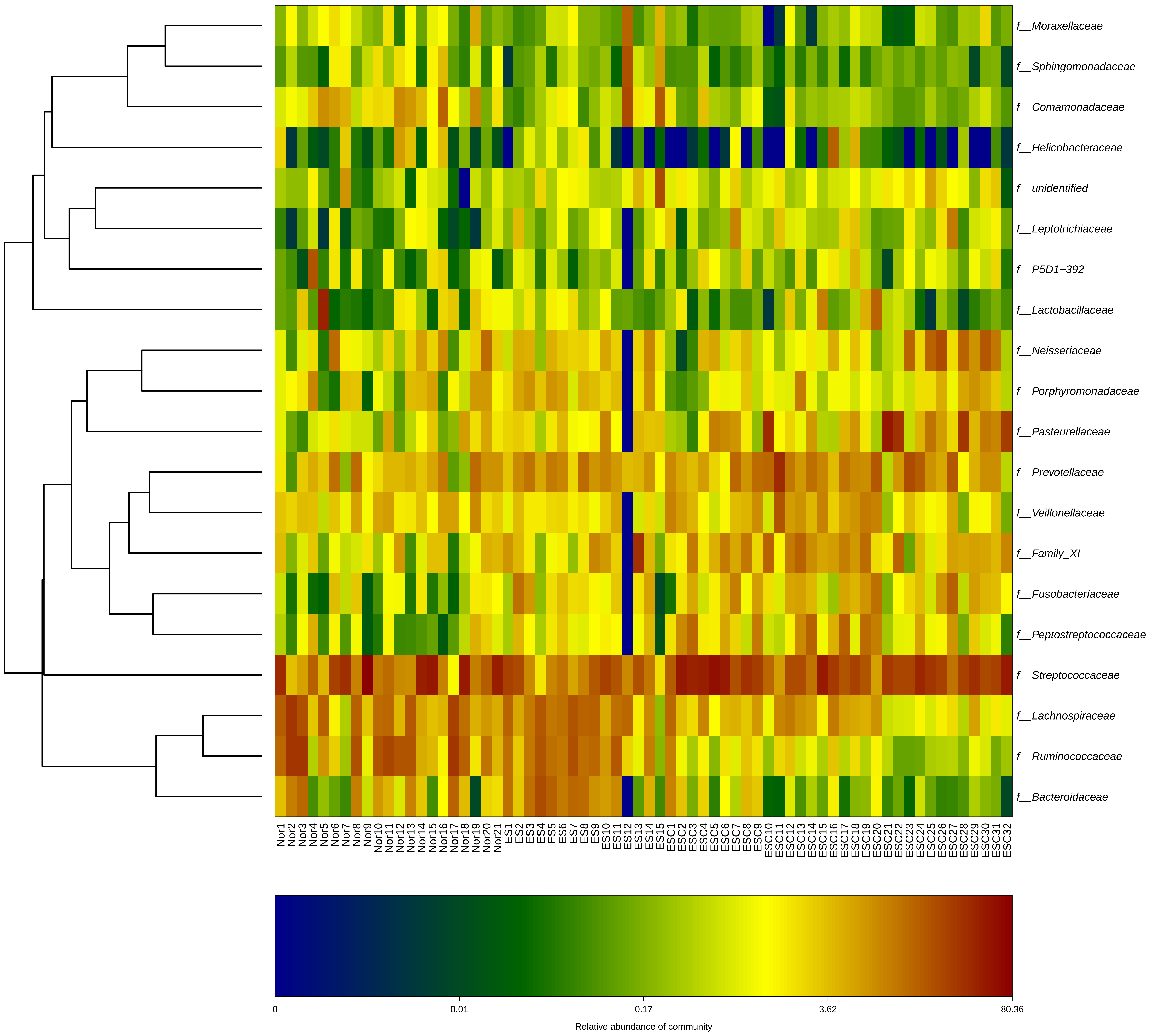
f__Moraxellaceae
f__Sphingomonadaceae
f__Comamonadaceae
f__Helicobacteraceae
f__unidentified
f__Leptotrichiaceae
f__P5D1−392
f__Lactobacillaceae
f__Neisseriaceae
f__Porphyromonadaceae
f__Pasteurellaceae
f__Prevotellaceae
f__Veillonellaceae
f__Family_XI
f__Fusobacteriaceae
f__Peptostreptococcaceae
f__Streptococcaceae
f__Lachnospiraceae
f__Ruminococcaceae
f__Bacteroidaceae
Nor1
Nor2
Nor3
Nor4
Nor5
Nor6
Nor7
Nor8
Nor9
Nor10
Nor11
Nor12
Nor13
Nor14
Nor15
Nor16
Nor17
Nor18
Nor19
Nor20
Nor21
ES1
ES2
ES3
ES4
ES5
ES6
ES7
ES8
ES9
ES10
ES11
ES12
ES13
ES14
ES15
ESC1
ESC2
ESC3
ESC4
ESC5
ESC6
ESC7
ESC8
ESC9
ESC10
ESC11
ESC12
ESC13
ESC14
ESC15
ESC16
ESC17
ESC18
ESC19
ESC20
ESC21
ESC22
ESC23
ESC24
ESC25
ESC26
ESC27
ESC28
ESC29
ESC30
ESC31
ESC32
0
0.01
0.17
3.62
80.36
Relative abundance of community
