# Supplementary material for: Characterization of Esophageal Microbiota in Patients With Esophagitis and Esophageal Squamous Cell Carcinoma
Source: Front Cell Infect Microbiol. 2021 Nov 11;11:774330. doi: 10.3389/fcimb.2021.774330 (PMC8632060; doi:10.3389/fcimb.2021.774330)
Supplement: Supplementary file 2 [file DataSheet_1.zip › 16S_V3_V4-68╕÷╤∙▒╛JZD 2020.7.21/3.Taxonomic_analysis/Heatmap/OTUs/Topgroups_OTUs.pdf]

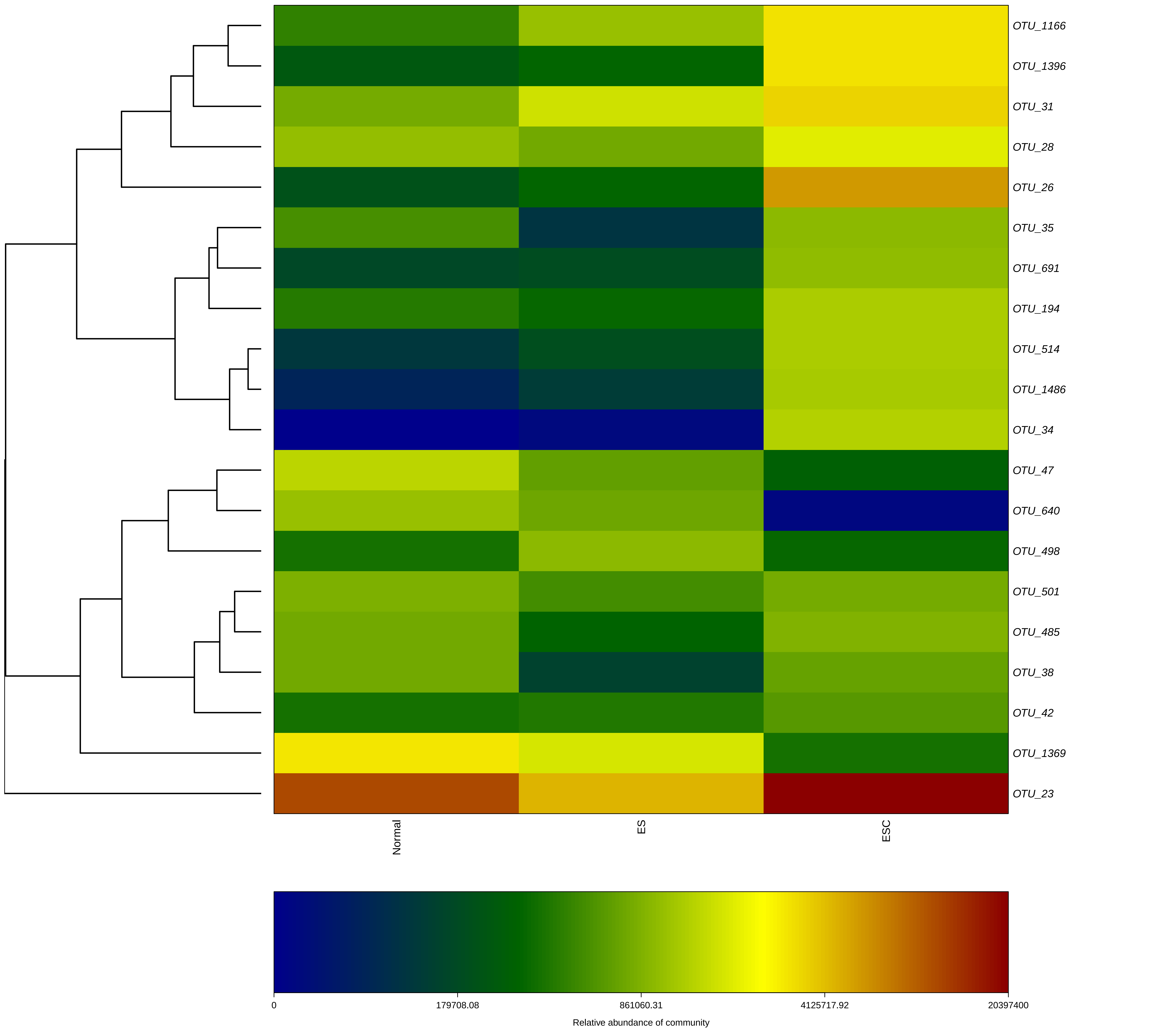
OTU_1166
OTU_1396
OTU_31
OTU_28
OTU_26
OTU_35
OTU_691
OTU_194
OTU_514
OTU_1486
OTU_34
OTU_47
OTU_640
OTU_498
OTU_501
OTU_485
OTU_38
OTU_42
OTU_1369
OTU_23
Normal
ES
ESC
0
179708.08
861060.31
4125717.92
20397400
Relative abundance of community
