# Supplementary material for: Characterization of Esophageal Microbiota in Patients With Esophagitis and Esophageal Squamous Cell Carcinoma
Source: Front Cell Infect Microbiol. 2021 Nov 11;11:774330. doi: 10.3389/fcimb.2021.774330 (PMC8632060; doi:10.3389/fcimb.2021.774330)
Supplement: Supplementary file 2 [file DataSheet_1.zip › 16S_V3_V4-68╕÷╤∙▒╛JZD 2020.7.21/3.Taxonomic_analysis/Heatmap/OTUs/Topsamples_OTUs.pdf]

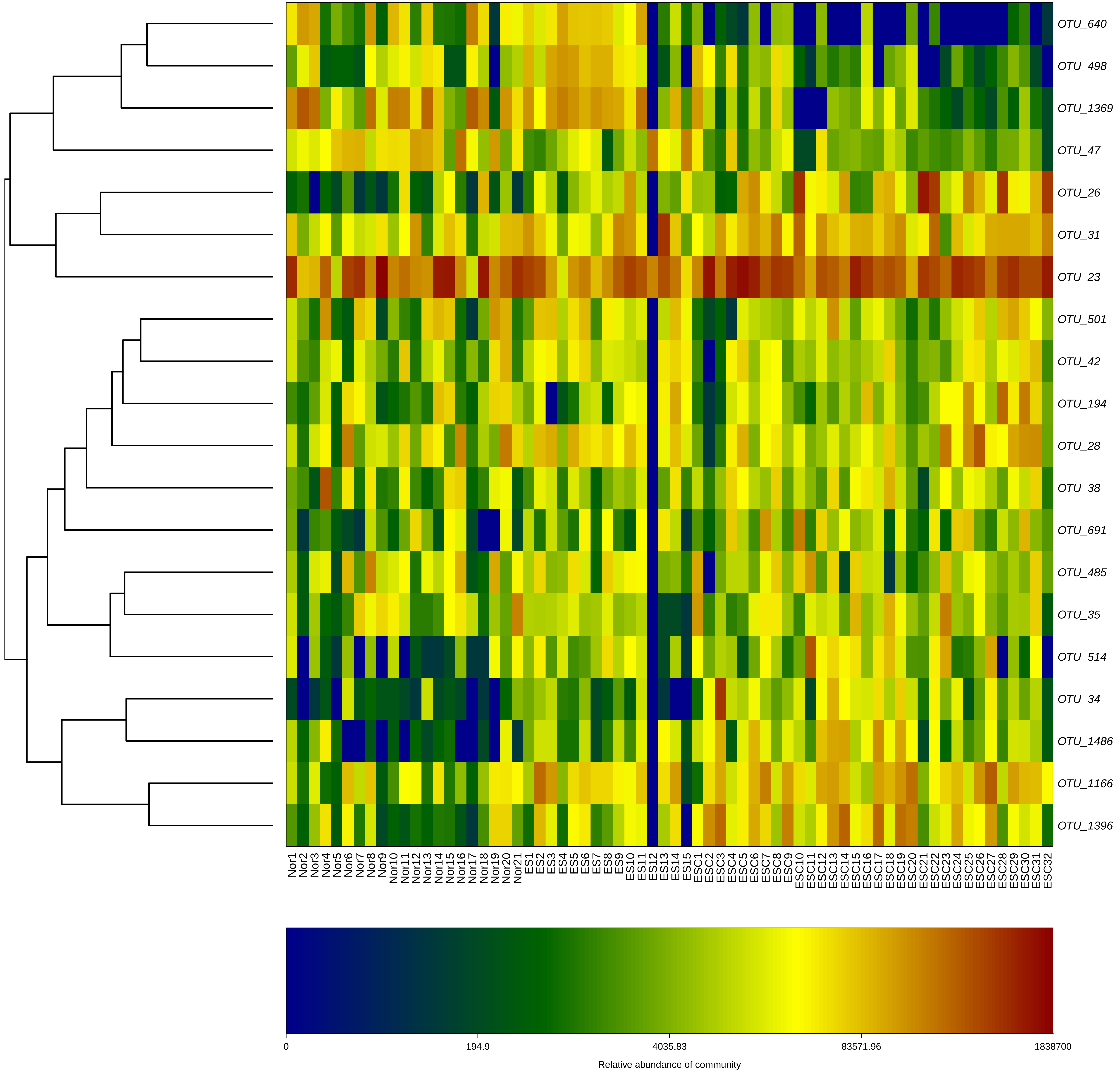
OTU_640
OTU_498
OTU_1369
OTU_47
OTU_26
OTU_31
OTU_23
OTU_501
OTU_42
OTU_194
OTU_28
OTU_38
OTU_691
OTU_485
OTU_35
OTU_514
OTU_34
OTU_1486
OTU_1166
OTU_1396
Nor1 Nor2 Nor3 Nor4 Nor5 Nor6 Nor7 Nor8 Nor9 Nor10 Nor11 Nor12 Nor13 Nor14 Nor15 Nor16 Nor17 Nor18 Nor19 Nor20 Nor21
ES1 ES2 ES3 ES4 ES5 ES6 ES7 ES8 ES9 ES10 ES11 ES12 ES13 ES14 ES15
ESC1 ESC2 ESC3 ESC4 ESC5 ESC6 ESC7 ESC8 ESC9 ESC10 ESC11 ESC12 ESC13 ESC14 ESC15 ESC16 ESC17 ESC18 ESC19 ESC20 ESC21 ESC22 ESC23 ESC24 ESC25 ESC26 ESC27 ESC28 ESC29 ESC30 ESC31 ESC32
0
194.9
4035.83
83571.96
1838700
Relative abundance of community
