# Supplementary material for: Characterization of Esophageal Microbiota in Patients With Esophagitis and Esophageal Squamous Cell Carcinoma
Source: Front Cell Infect Microbiol. 2021 Nov 11;11:774330. doi: 10.3389/fcimb.2021.774330 (PMC8632060; doi:10.3389/fcimb.2021.774330)
Supplement: Supplementary file 2 [file DataSheet_1.zip › 16S_V3_V4-68╕÷╤∙▒╛JZD 2020.7.21/3.Taxonomic_analysis/Levels_Bar_tree/Top_class.pdf]

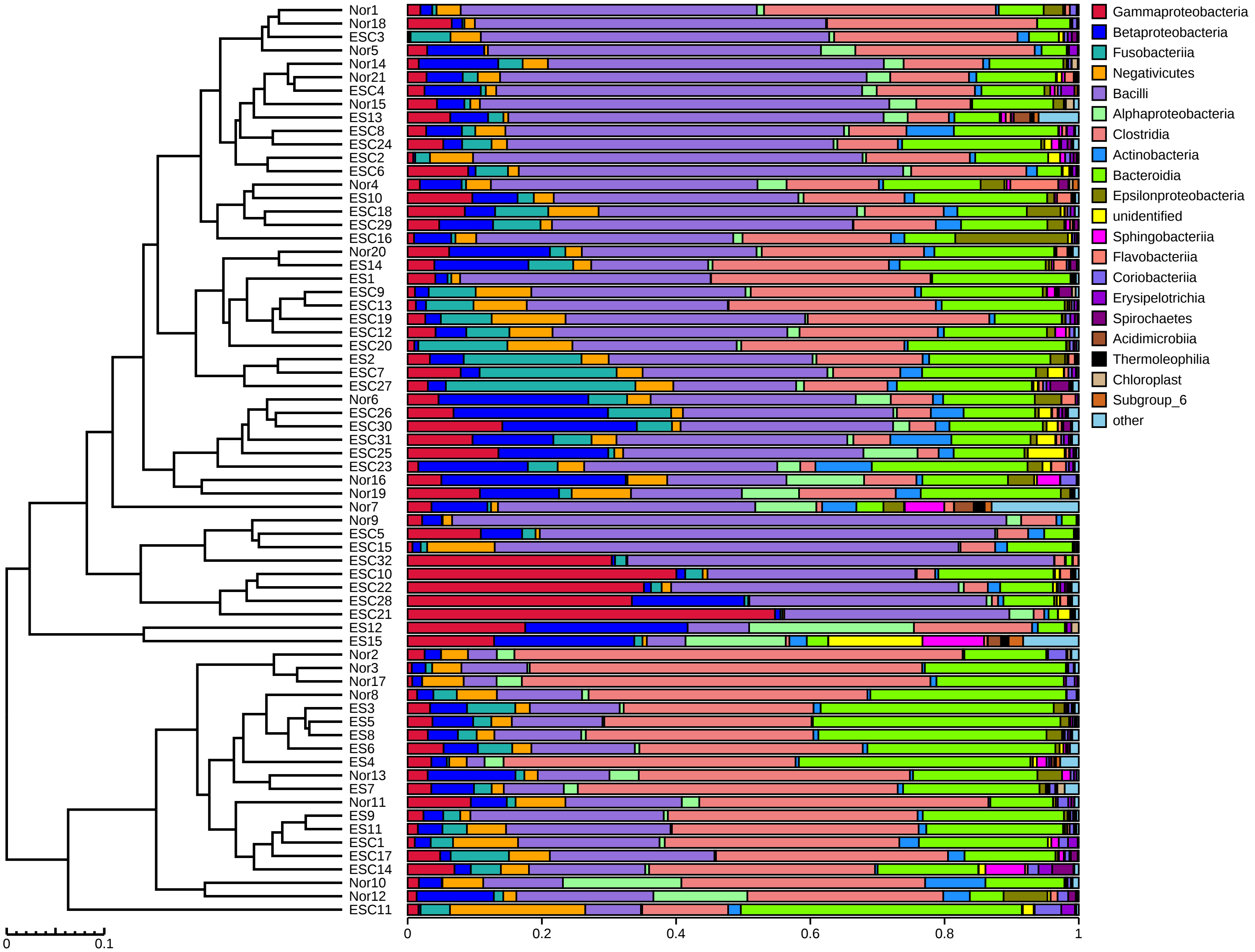

Nor1
Nor18
ESC3
Nor5
Nor14
Nor21
ESC4
Nor15
ES13
ESC8
ESC24
ESC2
ESC6
Nor4
ES10
ESC18
ESC29
ESC16
Nor20
ES14
ES1
ESC9
ESC13
ESC19
ESC12
ESC20
ES2
ESC7
ESC27
Nor6
ESC26
ESC30
ESC31
ESC25
ESC23
Nor16
Nor19
Nor7
Nor9
ESC5
ESC15
ESC32
ESC10
ESC22
ESC28
ESC21
ES12
ES15
Nor2
Nor3
Nor17
Nor8
ES3
ES5
ES8
ES6
ES4
Nor13
ES7
Nor11
ES9
ES11
ESC1
ESC17
ESC14
Nor10
Nor12
ESC11
0
0.1
0
0.2
0.4
0.6
0.8
1
Gammaproteobacteria
Betaproteobacteria
Fusobacteriia
Negativicutes
Bacilli
Alphaproteobacteria
Clostridia
Actinobacteria
Bacteroidia
Epsilonproteobacteria
unidentified
Sphingobacteriia
Flavobacteriia
Coriobacteriia
Erysipelotrichia
Spirochaetes
Acidimicrobiia
Thermoleophilia
Chloroplast
Subgroup_6
other
