# Supplementary material for: Characterization of Esophageal Microbiota in Patients With Esophagitis and Esophageal Squamous Cell Carcinoma
Source: Front Cell Infect Microbiol. 2021 Nov 11;11:774330. doi: 10.3389/fcimb.2021.774330 (PMC8632060; doi:10.3389/fcimb.2021.774330)
Supplement: Supplementary file 2 [file DataSheet_1.zip › 16S_V3_V4-68╕÷╤∙▒╛JZD 2020.7.21/3.Taxonomic_analysis/Levels_Bar_tree/Top_family.pdf]

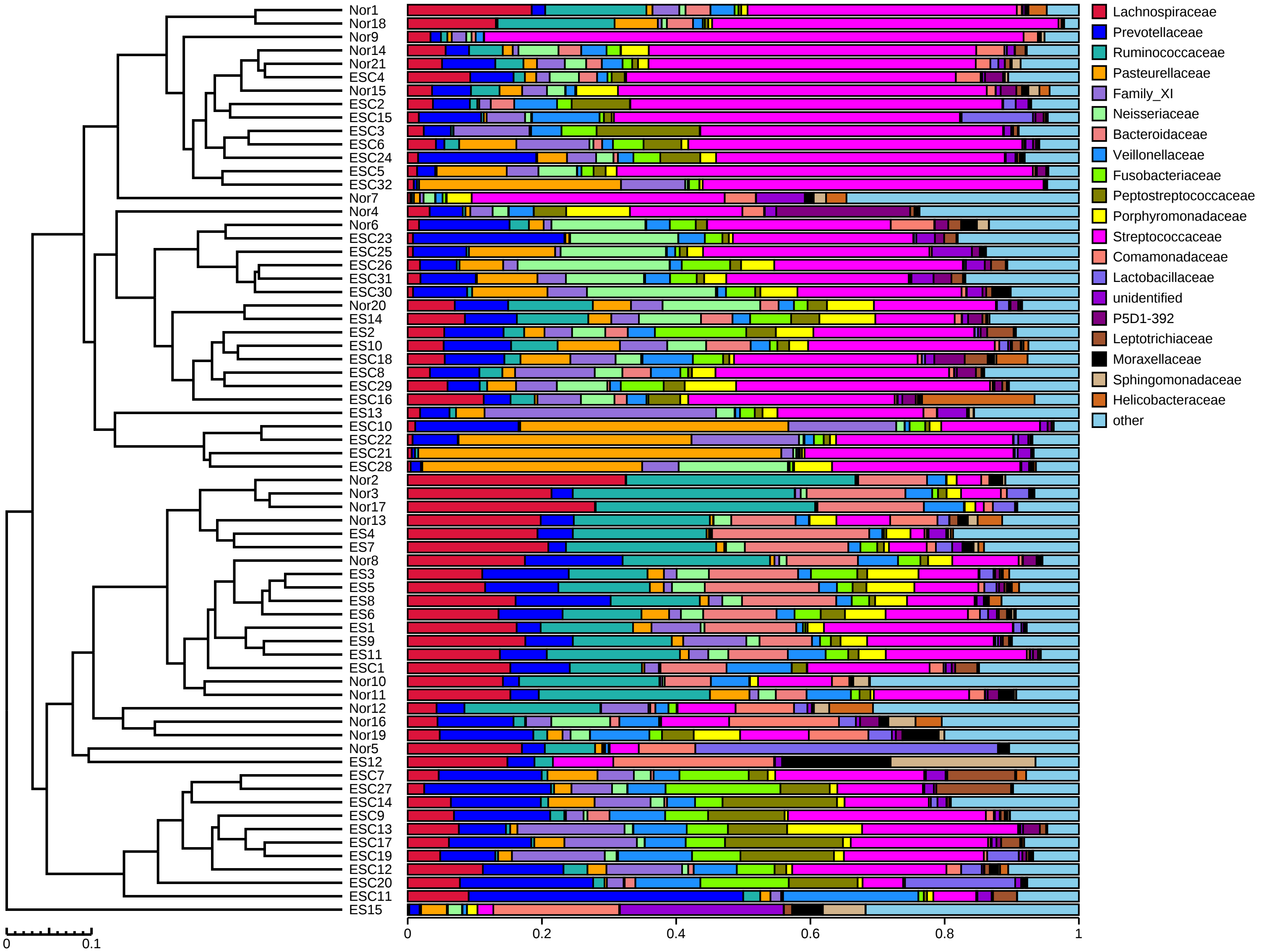
Nor1
Nor18
Nor9
Nor14
Nor21
ESC4
Nor15
ESC2
ESC15
ESC3
ESC6
ESC24
ESC5
ESC32
Nor7
Nor4
Nor6
ESC23
ESC25
ESC26
ESC31
ESC30
Nor20
ES14
ES2
ES10
ESC18
ESC8
ESC29
ESC16
ES13
ESC10
ESC22
ESC21
ESC28
Nor2
Nor3
Nor17
Nor13
ES4
ES7
Nor8
ES3
ES5
ES8
ES6
ES1
ES9
ES11
ESC1
Nor10
Nor11
Nor12
Nor16
Nor19
Nor5
ES12
ESC7
ESC27
ESC14
ESC9
ESC13
ESC17
ESC19
ESC12
ESC20
ESC11
ES15
0
0.1
0
0.2
0.4
0.6
0.8
1
Lachnospiraceae
Prevotellaceae
Ruminococcaceae
Pasteurellaceae
Family_XI
Neisseriaceae
Bacteroidaceae
Veillonellaceae
Fusobacteriaceae
Peptostreptococcaceae
Porphyromonadaceae
Streptococcaceae
Comamonadaceae
Lactobacillaceae
unidentified
P5D1-392
Leptotrichiaceae
Moraxellaceae
Sphingomonadaceae
Helicobacteraceae
other
