# Supplementary material for: Characterization of Esophageal Microbiota in Patients With Esophagitis and Esophageal Squamous Cell Carcinoma
Source: Front Cell Infect Microbiol. 2021 Nov 11;11:774330. doi: 10.3389/fcimb.2021.774330 (PMC8632060; doi:10.3389/fcimb.2021.774330)
Supplement: Supplementary file 2 [file DataSheet_1.zip › 16S_V3_V4-68╕÷╤∙▒╛JZD 2020.7.21/3.Taxonomic_analysis/Levels_Bar_tree/Top_genus.pdf]

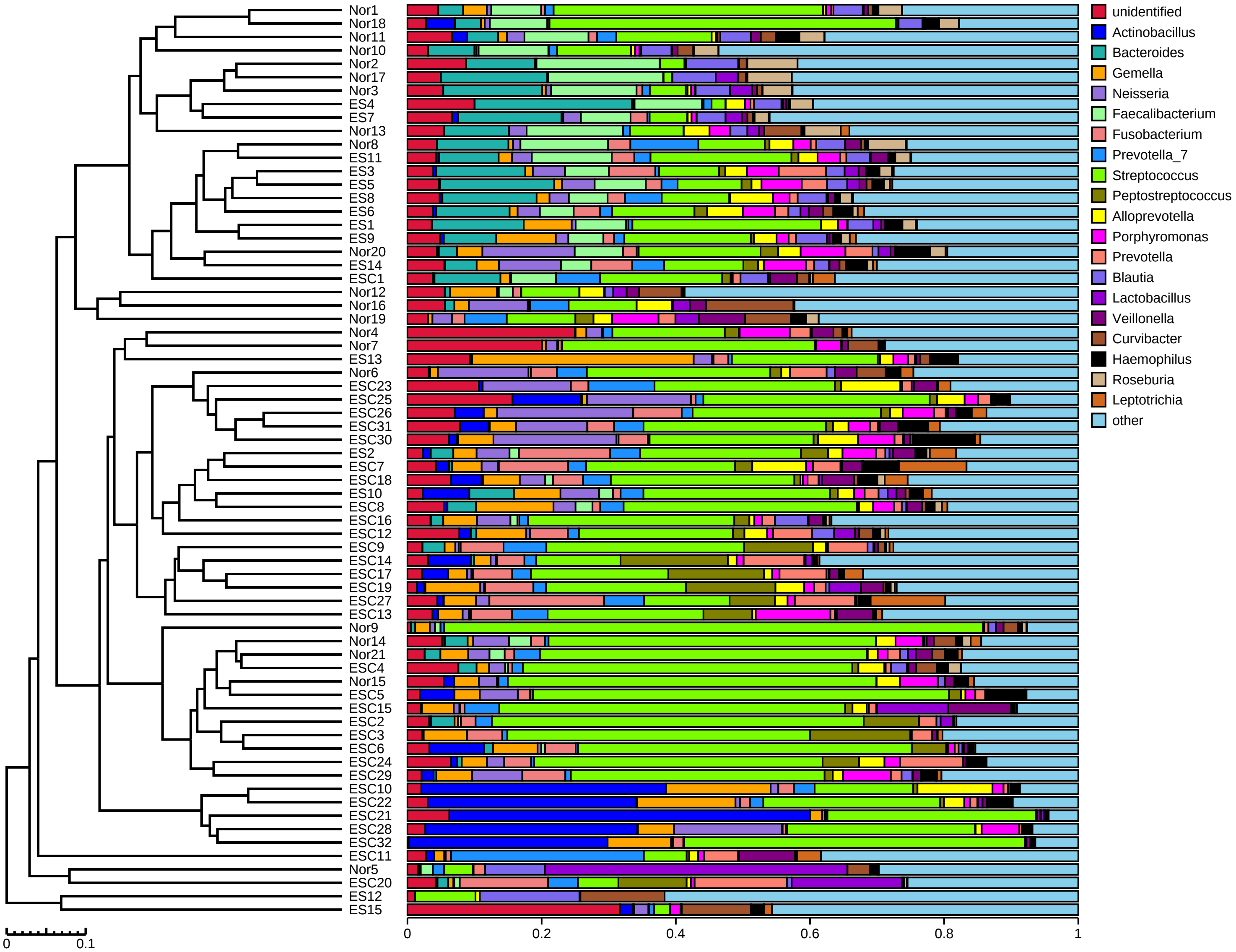

Nor1
Nor18
Nor11
Nor10
Nor2
Nor17
Nor3
ES4
ES7
Nor13
Nor8
ES11
ES3
ES5
ES8
ES6
ES1
ES9
Nor20
ES14
ESC1
Nor12
Nor16
Nor19
Nor4
Nor7
ES13
Nor6
ESC23
ESC25
ESC26
ESC31
ESC30
ES2
ESC7
ESC18
ES10
ESC8
ESC16
ESC12
ESC9
ESC14
ESC17
ESC19
ESC27
ESC13
Nor9
Nor14
Nor21
ESC4
Nor15
ESC5
ESC15
ESC2
ESC3
ESC6
ESC24
ESC29
ESC10
ESC22
ESC21
ESC28
ESC32
ESC11
Nor5
ESC20
ES12
ES15
0
0.1
0
0.2
0.4
0.6
0.8
1
unidentified
Actinobacillus
Bacteroides
Gemella
Neisseria
Faecalibacterium
Fusobacterium
Prevotella_7
Streptococcus
Peptostreptococcus
Alloprevotella
Porphyromonas
Prevotella
Blautia
Lactobacillus
Veillonella
Curvibacter
Haemophilus
Roseburia
Leptotrichia
other
