# Supplementary material for: Characterization of Esophageal Microbiota in Patients With Esophagitis and Esophageal Squamous Cell Carcinoma
Source: Front Cell Infect Microbiol. 2021 Nov 11;11:774330. doi: 10.3389/fcimb.2021.774330 (PMC8632060; doi:10.3389/fcimb.2021.774330)
Supplement: Supplementary file 2 [file DataSheet_1.zip › 16S_V3_V4-68╕÷╤∙▒╛JZD 2020.7.21/3.Taxonomic_analysis/Levels_Bar_tree/Top_order.pdf]

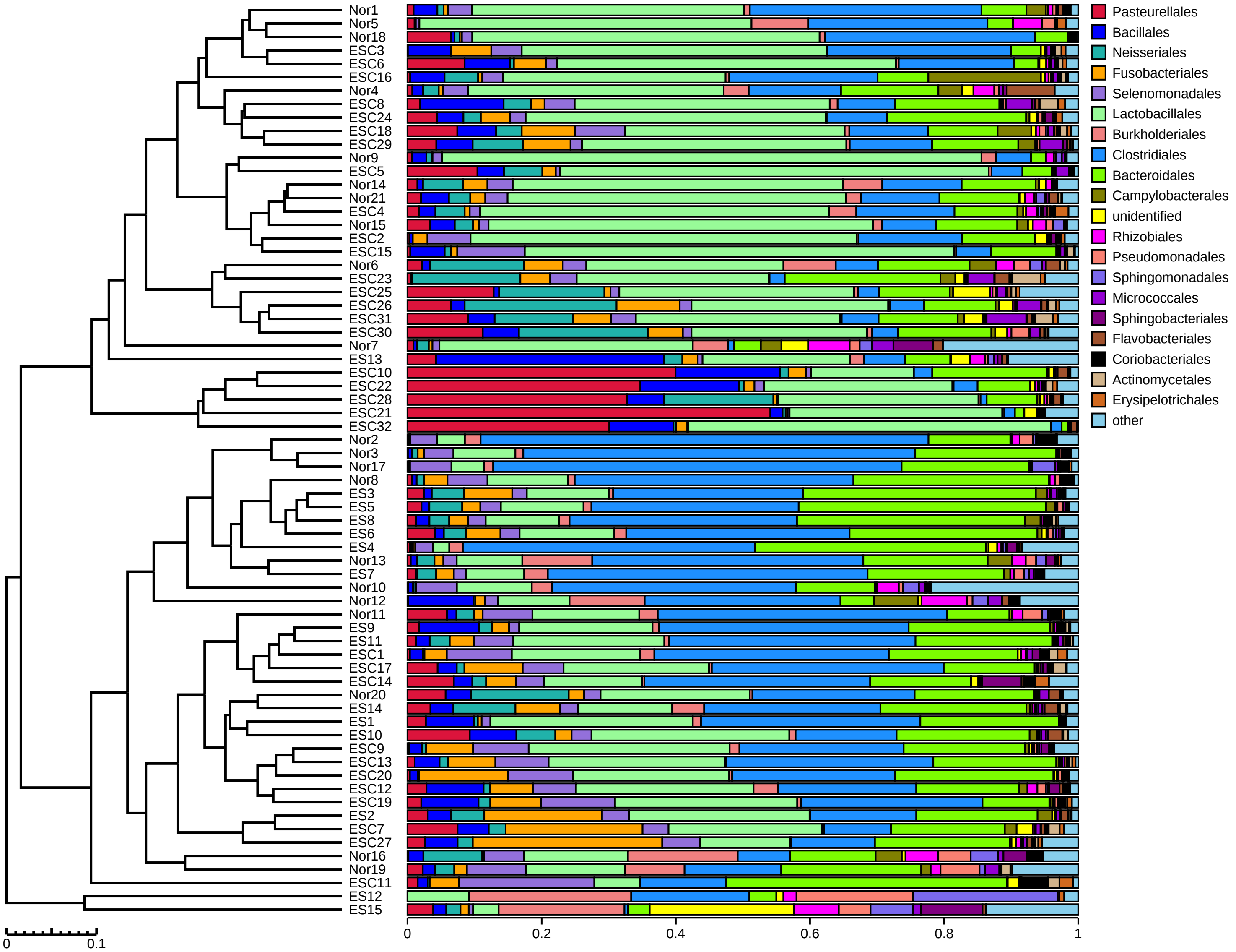
Nor1
Nor5
Nor18
ESC3
ESC6
ESC16
Nor4
ESC8
ESC24
ESC18
ESC29
Nor9
ESC5
Nor14
Nor21
ESC4
Nor15
ESC2
ESC15
Nor6
ESC23
ESC25
ESC26
ESC31
ESC30
Nor7
ES13
ESC10
ESC22
ESC28
ESC21
ESC32
Nor2
Nor3
Nor17
Nor8
ES3
ES5
ES8
ES6
ES4
Nor13
ES7
Nor10
Nor12
Nor11
ES9
ES11
ESC1
ESC17
ESC14
Nor20
ES14
ES1
ES10
ESC9
ESC13
ESC20
ESC12
ESC19
ES2
ESC7
ESC27
Nor16
Nor19
ESC11
ES12
ES15
0
0.1
0
0.2
0.4
0.6
0.8
1
Pasteurellales
Bacillales
Neisseriales
Fusobacteriales
Selenomonadales
Lactobacillales
Burkholderiales
Clostridiales
Bacteroidales
Campylobacterales
unidentified
Rhizobiales
Pseudomonadales
Sphingomonadales
Micrococcales
Sphingobacteriales
Flavobacteriales
Coriobacteriales
Actinomycetales
Erysipelotrichales
other
