# Supplementary material for: Characterization of Esophageal Microbiota in Patients With Esophagitis and Esophageal Squamous Cell Carcinoma
Source: Front Cell Infect Microbiol. 2021 Nov 11;11:774330. doi: 10.3389/fcimb.2021.774330 (PMC8632060; doi:10.3389/fcimb.2021.774330)
Supplement: Supplementary file 2 [file DataSheet_1.zip › 16S_V3_V4-68╕÷╤∙▒╛JZD 2020.7.21/3.Taxonomic_analysis/Levels_Bar_tree/Top_phylum.pdf]

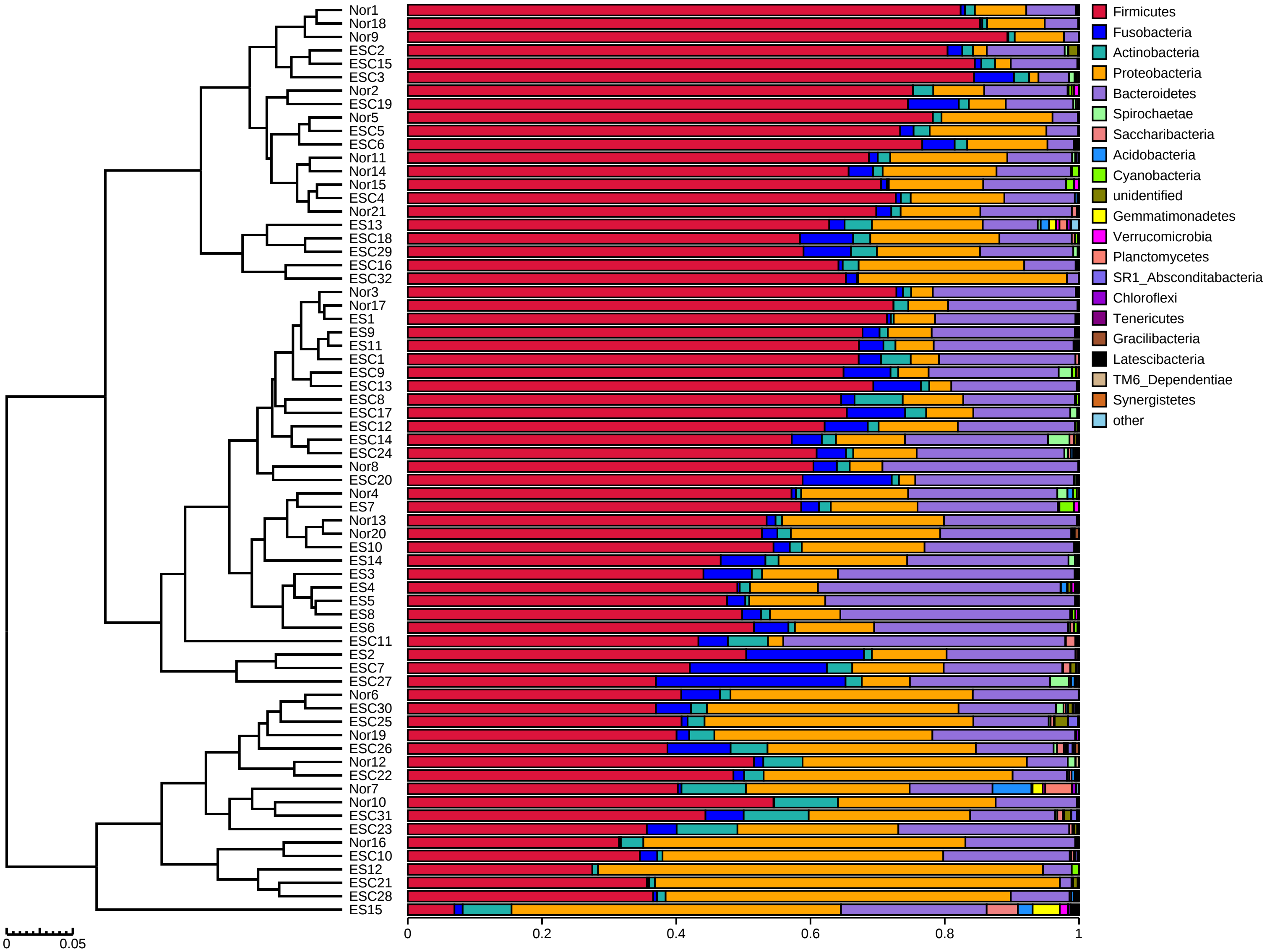
Nor1
Nor18
Nor9
ESC2
ESC15
ESC3
Nor2
ESC19
Nor5
ESC5
ESC6
Nor11
Nor14
Nor15
ESC4
Nor21
ES13
ESC18
ESC29
ESC16
ESC32
Nor3
Nor17
ES1
ES9
ES11
ESC1
ESC9
ESC13
ESC8
ESC17
ESC12
ESC14
ESC24
Nor8
ESC20
Nor4
ES7
Nor13
Nor20
ES10
ES14
ES3
ES4
ES5
ES8
ES6
ESC11
ES2
ESC7
ESC27
Nor6
ESC30
ESC25
Nor19
ESC26
Nor12
ESC22
Nor7
Nor10
ESC31
ESC23
Nor16
ESC10
ES12
ESC21
ESC28
ES15
0
0.05
0
0.2
0.4
0.6
0.8
1
Firmicutes
Fusobacteria
Actinobacteria
Proteobacteria
Bacteroidetes
Spirochaetae
Saccharibacteria
Acidobacteria
Cyanobacteria
unidentified
Gemmatimonadetes
Verrucomicrobia
Planctomycetes
SR1_Absconditabacteria
Chloroflexi
Tenericutes
Gracilibacteria
Latescibacteria
TM6_Dependentiae
Synergistetes
other
