# Supplementary material for: Characterization of Esophageal Microbiota in Patients With Esophagitis and Esophageal Squamous Cell Carcinoma
Source: Front Cell Infect Microbiol. 2021 Nov 11;11:774330. doi: 10.3389/fcimb.2021.774330 (PMC8632060; doi:10.3389/fcimb.2021.774330)
Supplement: Supplementary file 2 [file DataSheet_1.zip › 16S_V3_V4-68╕÷╤∙▒╛JZD 2020.7.21/4.Beta_diversity_analysis/(Un)Weighted_Unifrac_distance_boxplot/unweighted_unifrac.txt.pdf]

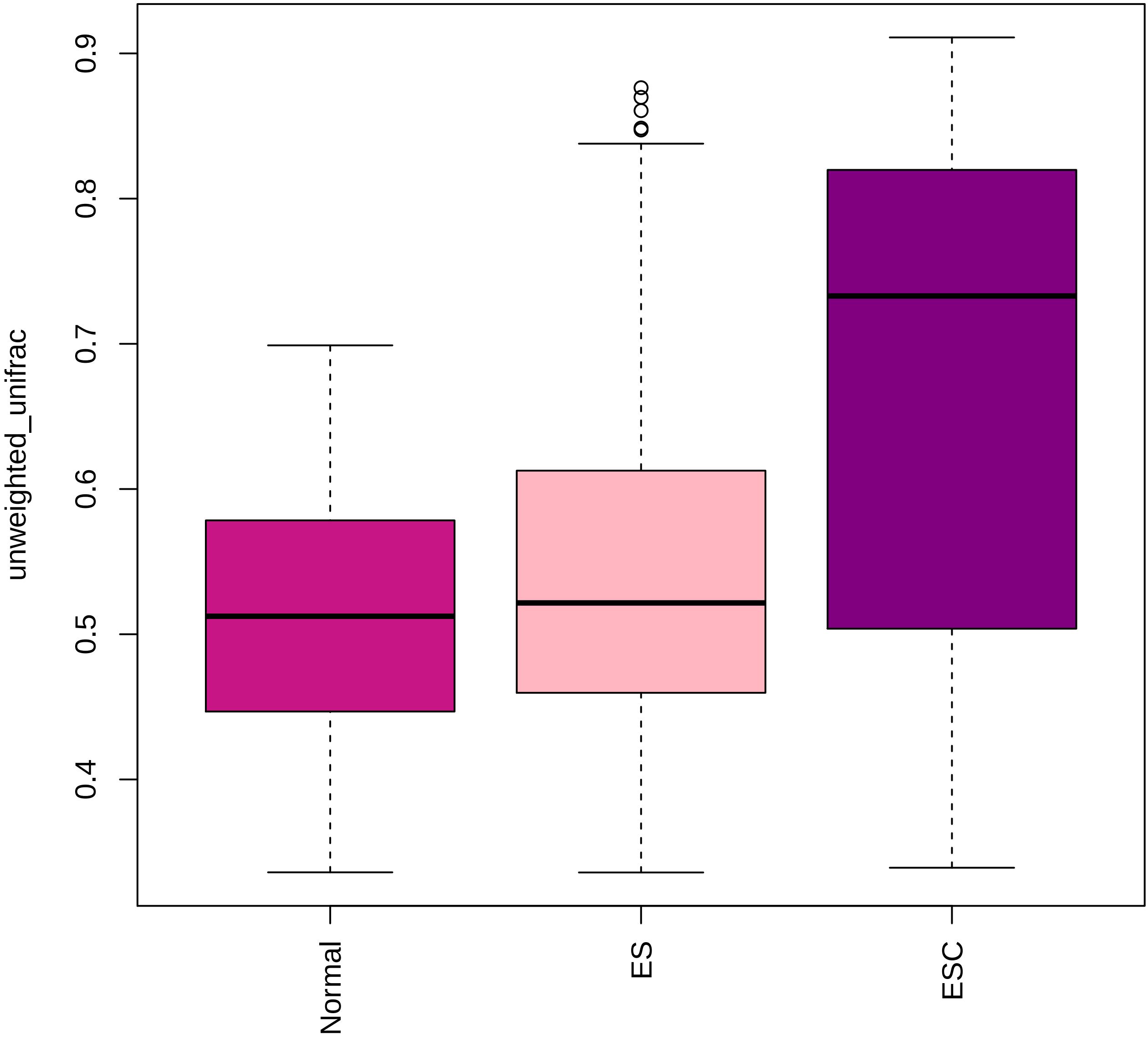

unweighted_unifrac
0.4
0.5
0.6
0.7
0.8
0.9
Normal
ES
ESC
