# Supplementary material for: Characterization of Esophageal Microbiota in Patients With Esophagitis and Esophageal Squamous Cell Carcinoma
Source: Front Cell Infect Microbiol. 2021 Nov 11;11:774330. doi: 10.3389/fcimb.2021.774330 (PMC8632060; doi:10.3389/fcimb.2021.774330)
Supplement: Supplementary file 2 [file DataSheet_1.zip › 16S_V3_V4-68╕÷╤∙▒╛JZD 2020.7.21/4.Beta_diversity_analysis/(Un)Weighted_Unifrac_distance_heatmap/unweighted_unifrac_distance.pdf]

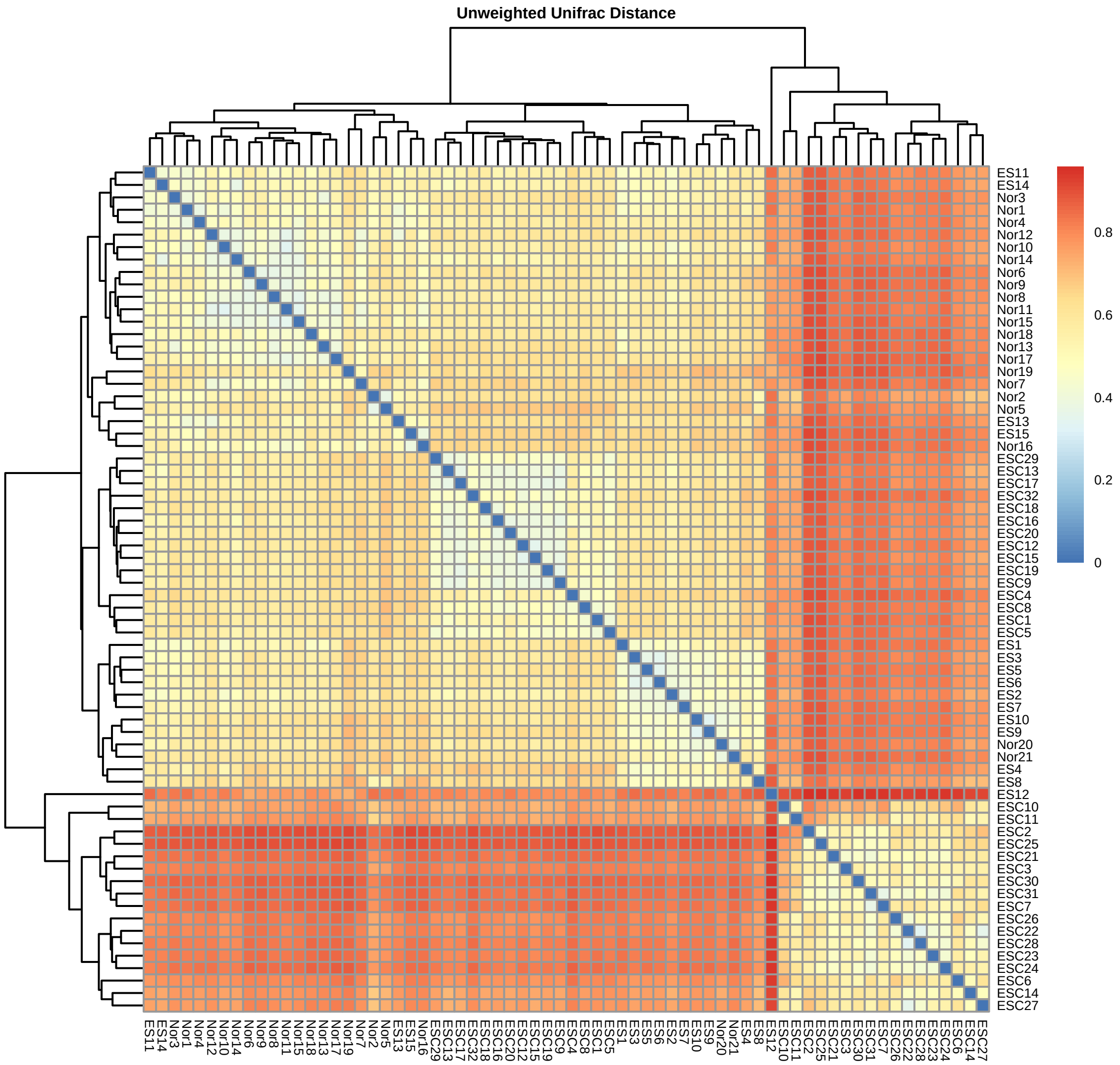
Unweighted Unifrac Distance
ES11
ES14
Nor3
Nor1
Nor4
Nor12
Nor10
Nor14
Nor6
Nor9
Nor8
Nor11
Nor15
Nor18
Nor13
Nor17
Nor19
Nor7
Nor2
Nor5
ES13
ES15
Nor16
ESC29
ESC13
ESC17
ESC32
ESC18
ESC16
ESC20
ESC12
ESC15
ESC19
ESC9
ESC4
ESC8
ESC1
ESC5
ES1
ES3
ES5
ES6
ES2
ES7
ES10
ES9
Nor20
Nor21
ES4
ES8
ES12
ESC10
ESC11
ESC2
ESC25
ESC21
ESC3
ESC30
ESC31
ESC7
ESC26
ESC22
ESC28
ESC23
ESC24
ESC6
ESC14
ESC27
0.8
0.6
0.4
0.2
0
