# Supplementary material for: Characterization of Esophageal Microbiota in Patients With Esophagitis and Esophageal Squamous Cell Carcinoma
Source: Front Cell Infect Microbiol. 2021 Nov 11;11:774330. doi: 10.3389/fcimb.2021.774330 (PMC8632060; doi:10.3389/fcimb.2021.774330)
Supplement: Supplementary file 2 [file DataSheet_1.zip › 16S_V3_V4-68╕÷╤∙▒╛JZD 2020.7.21/4.Beta_diversity_analysis/(Un)Weighted_Unifrac_distance_heatmap/weighted_unifrac_distance.pdf]

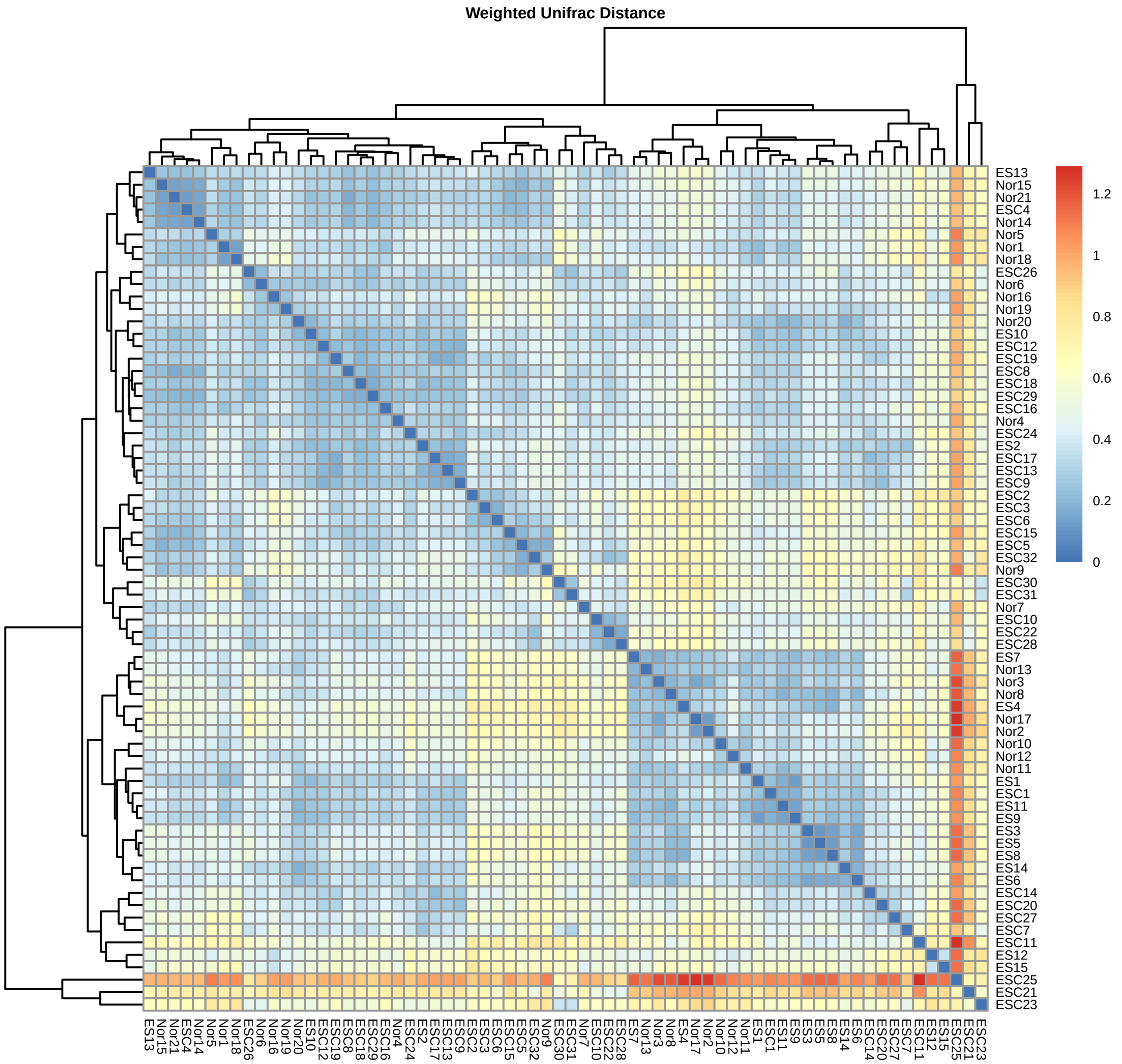
Weighted Unifrac Distance
1.2
1
0.8
0.6
0.4
0.2
0
ES13
Nor15
Nor21
ESC4
Nor14
Nor5
Nor1
Nor18
ESC26
Nor6
Nor16
Nor19
Nor20
ES10
ESC12
ESC19
ESC8
ESC18
ESC29
ESC16
Nor4
ESC24
ES2
ESC17
ESC13
ESC9
ESC2
ESC3
ESC6
ESC15
ESC5
ESC32
Nor9
ESC30
ESC31
Nor7
ESC10
ESC22
ESC28
ES7
Nor13
Nor3
Nor8
ES4
Nor17
Nor2
Nor10
Nor12
Nor11
ES1
ESC1
ES11
ES9
ES3
ES5
ES8
ES14
ES6
ESC14
ESC20
ESC27
ESC7
ESC11
ES12
ES15
ESC25
ESC21
ESC23
