# Supplementary material for: Characterization of Esophageal Microbiota in Patients With Esophagitis and Esophageal Squamous Cell Carcinoma
Source: Front Cell Infect Microbiol. 2021 Nov 11;11:774330. doi: 10.3389/fcimb.2021.774330 (PMC8632060; doi:10.3389/fcimb.2021.774330)
Supplement: Supplementary file 2 [file DataSheet_1.zip › 16S_V3_V4-68╕÷╤∙▒╛JZD 2020.7.21/4.Beta_diversity_analysis/Bray-Curtis_PCoA/OTUs_PCOA_notext.pdf]

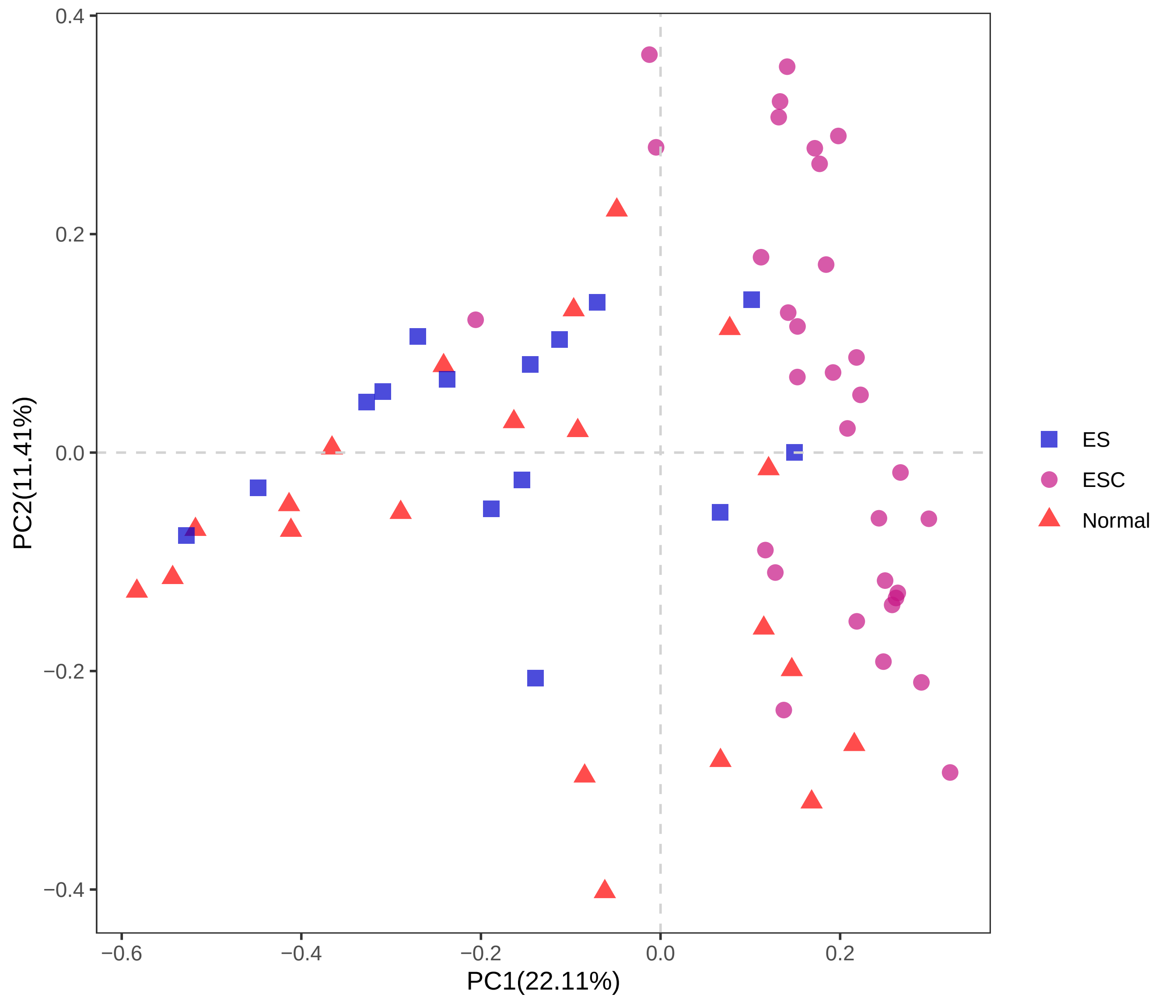
0.4
0.2
0.0
−0.2
−0.4
PC2(11.41%)
−0.6
−0.4
−0.2
0.0
0.2
PC1(22.11%)
ES
ESC
Normal
