# Supplementary material for: Characterization of Esophageal Microbiota in Patients With Esophagitis and Esophageal Squamous Cell Carcinoma
Source: Front Cell Infect Microbiol. 2021 Nov 11;11:774330. doi: 10.3389/fcimb.2021.774330 (PMC8632060; doi:10.3389/fcimb.2021.774330)
Supplement: Supplementary file 2 [file DataSheet_1.zip › 16S_V3_V4-68╕÷╤∙▒╛JZD 2020.7.21/4.Beta_diversity_analysis/Bray-Curtis_PCoA/OTUs_PCOA_text.pdf]

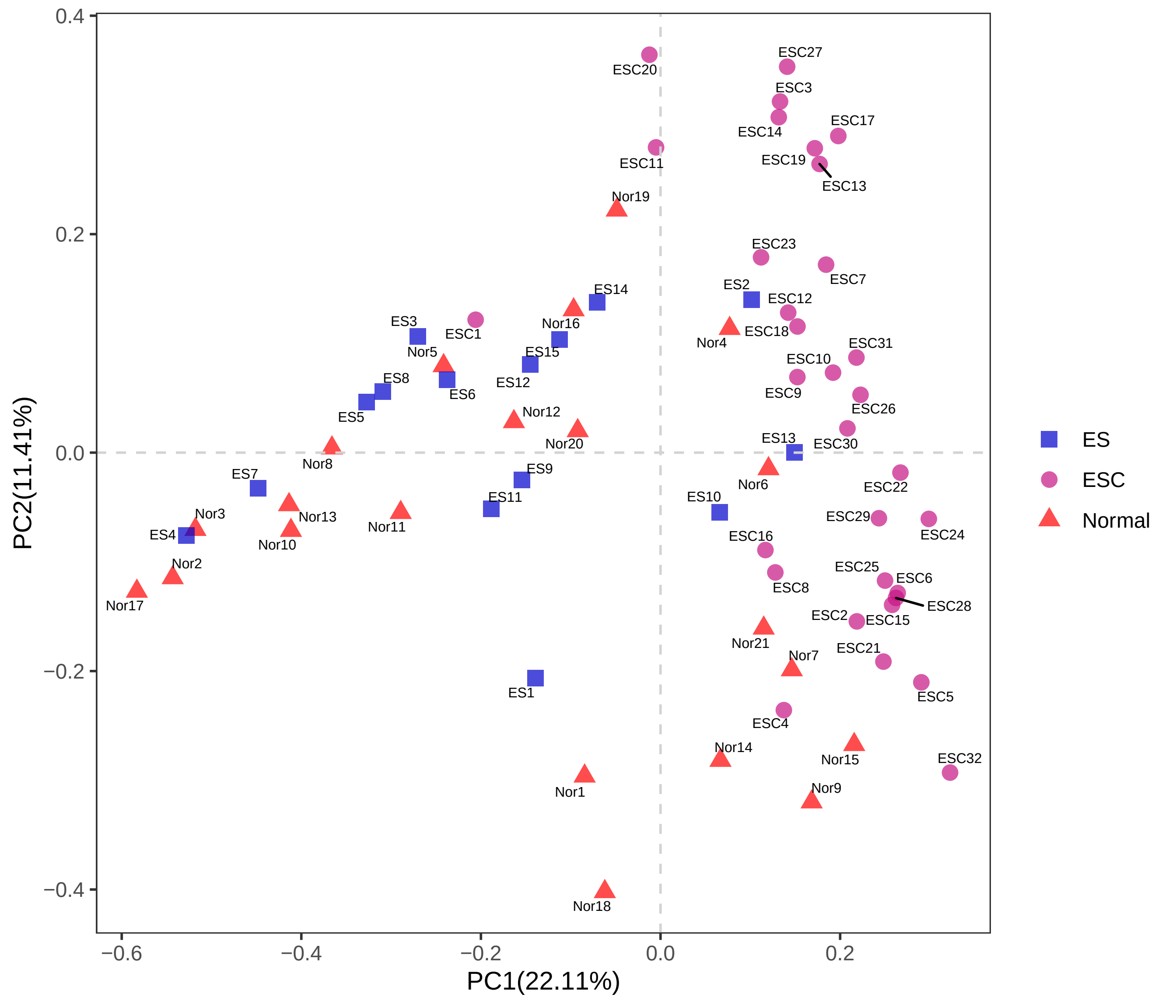

PC2(11.41%)
PC1(22.11%)
0.4
0.2
0.0
−0.2
−0.4
−0.6
−0.4
−0.2
0.0
0.2
ES
ESC
Normal
ESC20
ESC27
ESC3
ESC14
ESC17
ESC19
ESC13
ESC11
Nor19
ESC23
ESC7
ES2
ESC12
ESC18
Nor4
ES14
Nor16
ES3
ESC1
Nor5
ES15
ES12
ES8
ES6
ES5
Nor12
Nor20
Nor8
ESC31
ESC10
ESC9
ESC26
ESC30
ES13
Nor6
ESC22
ES7
ES9
ES11
Nor3
ES4
Nor13
Nor10
Nor11
ES10
ESC29
ESC24
ESC16
Nor2
Nor17
ESC25
ESC6
ESC8
ESC28
ESC2
ESC15
Nor21
Nor7
ESC21
ES1
ESC5
ESC4
Nor14
Nor15
ESC32
Nor1
Nor9
Nor18
