# Supplementary material for: Characterization of Esophageal Microbiota in Patients With Esophagitis and Esophageal Squamous Cell Carcinoma
Source: Front Cell Infect Microbiol. 2021 Nov 11;11:774330. doi: 10.3389/fcimb.2021.774330 (PMC8632060; doi:10.3389/fcimb.2021.774330)
Supplement: Supplementary file 2 [file DataSheet_1.zip › 16S_V3_V4-68╕÷╤∙▒╛JZD 2020.7.21/4.Beta_diversity_analysis/Hcluster/f_cluster.pdf]

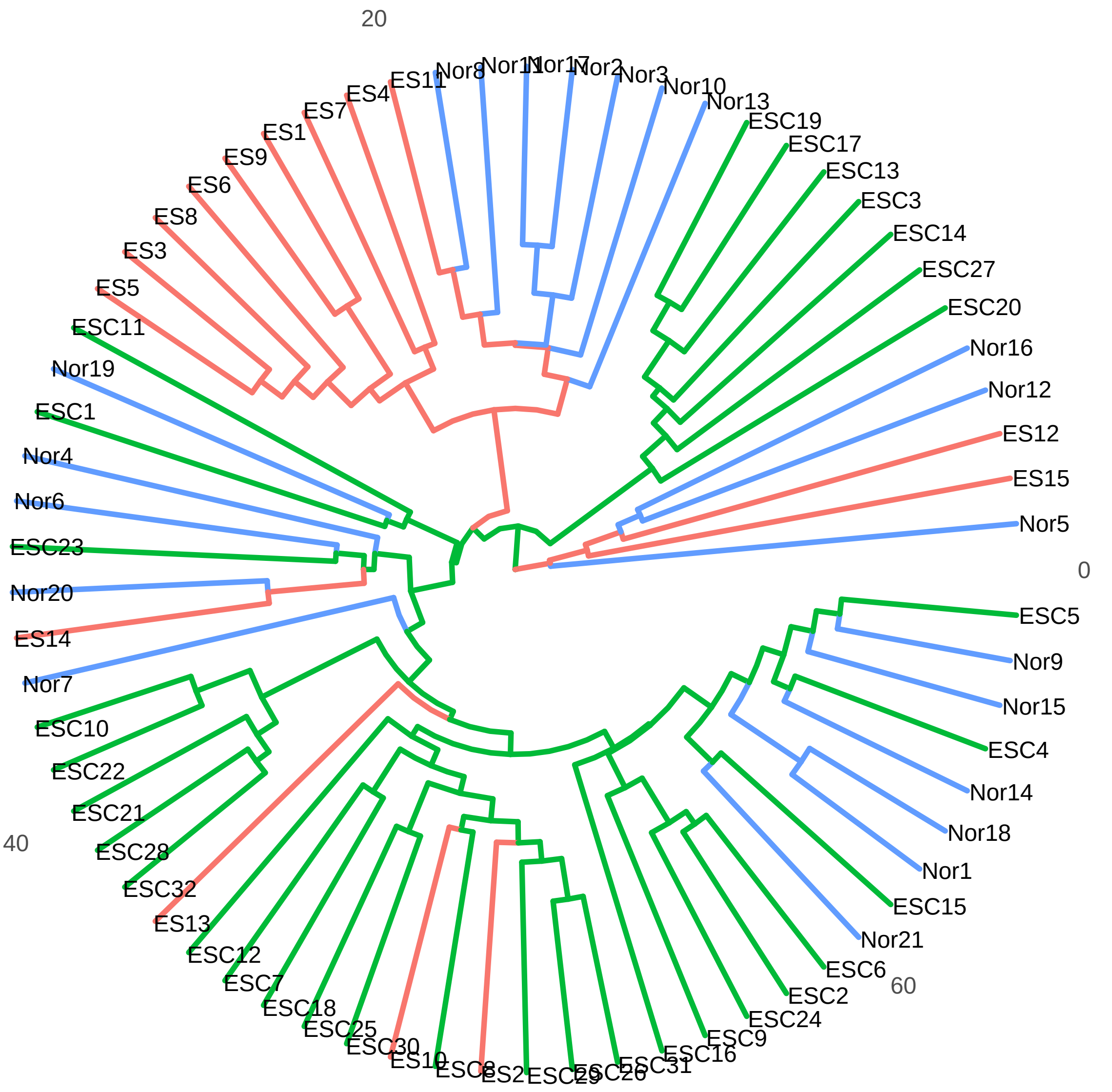
20
0
40
60
Nor5
ES15
ES12
Nor12
Nor16
ESC20
ESC27
ESC14
ESC3
ESC13
ESC17
ESC19
Nor13
Nor10
Nor3
Nor2
Nor17
Nor11
Nor8
ES11
ES4
ES7
ES1
ES9
ES6
ES8
ES3
ES5
ESC11
Nor19
ESC1
Nor4
Nor6
ESC23
Nor20
ES14
Nor7
ESC10
ESC22
ESC21
ESC28
ESC32
ES13
ESC12
ESC7
ESC18
ESC25
ESC30
ES10
ESC8
ES2
ESC29
ESC26
ESC31
ESC16
ESC9
ESC24
ESC2
ESC6
Nor21
ESC15
Nor1
Nor18
Nor14
ESC4
Nor15
Nor9
ESC5
