# Supplementary material for: Characterization of Esophageal Microbiota in Patients With Esophagitis and Esophageal Squamous Cell Carcinoma
Source: Front Cell Infect Microbiol. 2021 Nov 11;11:774330. doi: 10.3389/fcimb.2021.774330 (PMC8632060; doi:10.3389/fcimb.2021.774330)
Supplement: Supplementary file 2 [file DataSheet_1.zip › 16S_V3_V4-68╕÷╤∙▒╛JZD 2020.7.21/4.Beta_diversity_analysis/Hcluster/p_cluster.pdf]

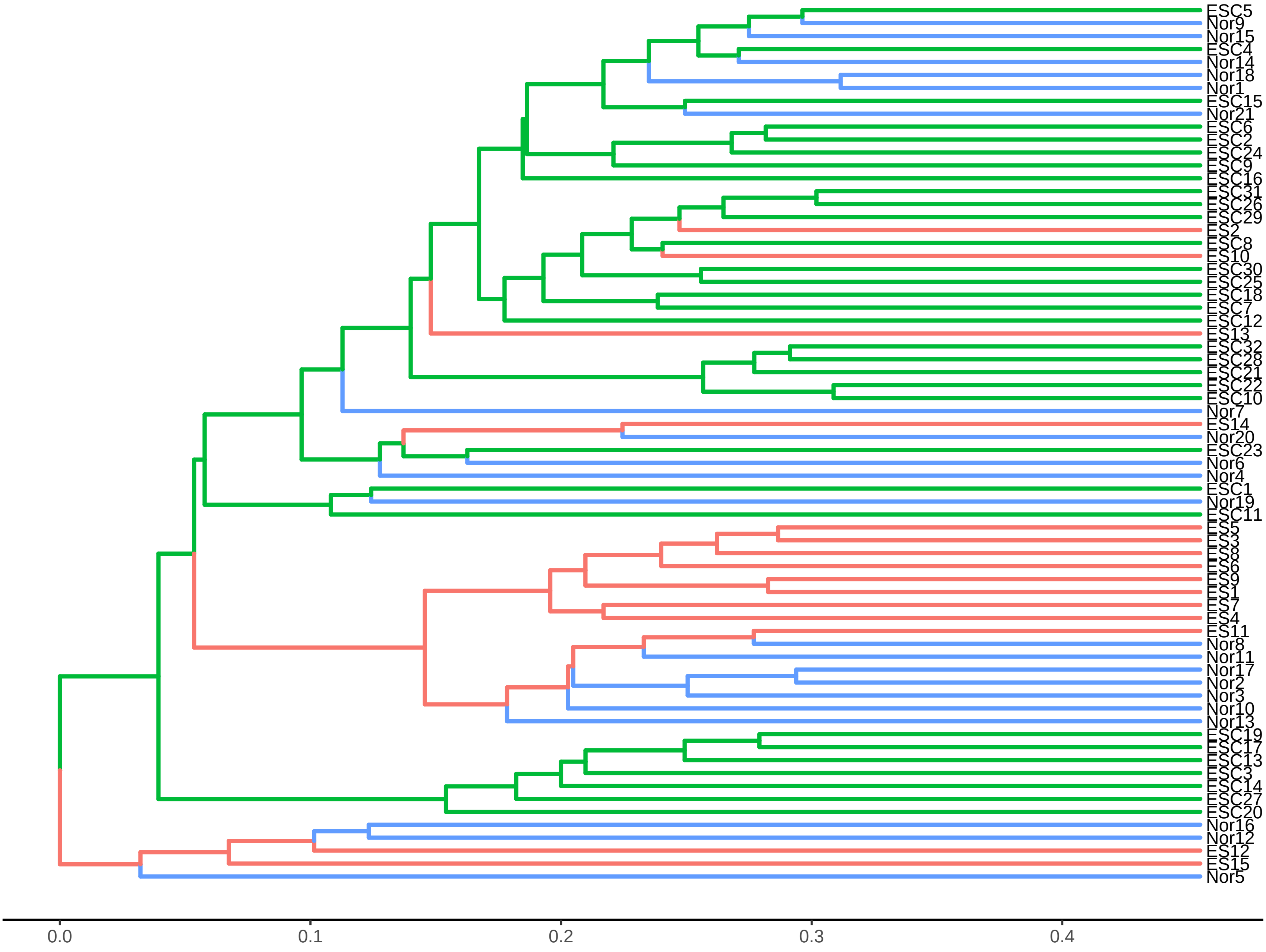
ESC5
Nor9
Nor15
ESC4
Nor14
Nor18
Nor1
ESC15
Nor21
ESC6
ESC2
ESC24
ESC9
ESC16
ESC31
ESC26
ESC29
ES2
ESC8
ES10
ESC30
ESC25
ESC18
ESC7
ESC12
ES13
ESC32
ESC28
ESC21
ESC22
ESC10
Nor7
ES14
Nor20
ESC23
Nor6
Nor4
ESC1
Nor19
ESC11
ES5
ES3
ES8
ES6
ES9
ES1
ES7
ES4
ES11
Nor8
Nor11
Nor17
Nor2
Nor3
Nor10
Nor13
ESC19
ESC17
ESC13
ESC3
ESC14
ESC27
ESC20
Nor16
Nor12
ES12
ES15
Nor5
0.0
0.1
0.2
0.3
0.4
