# Supplementary material for: Characterization of Esophageal Microbiota in Patients With Esophagitis and Esophageal Squamous Cell Carcinoma
Source: Front Cell Infect Microbiol. 2021 Nov 11;11:774330. doi: 10.3389/fcimb.2021.774330 (PMC8632060; doi:10.3389/fcimb.2021.774330)
Supplement: Supplementary file 2 [file DataSheet_1.zip › 16S_V3_V4-68╕÷╤∙▒╛JZD 2020.7.21/4.Beta_diversity_analysis/PCA/OTUs_PCA_notext.pdf]

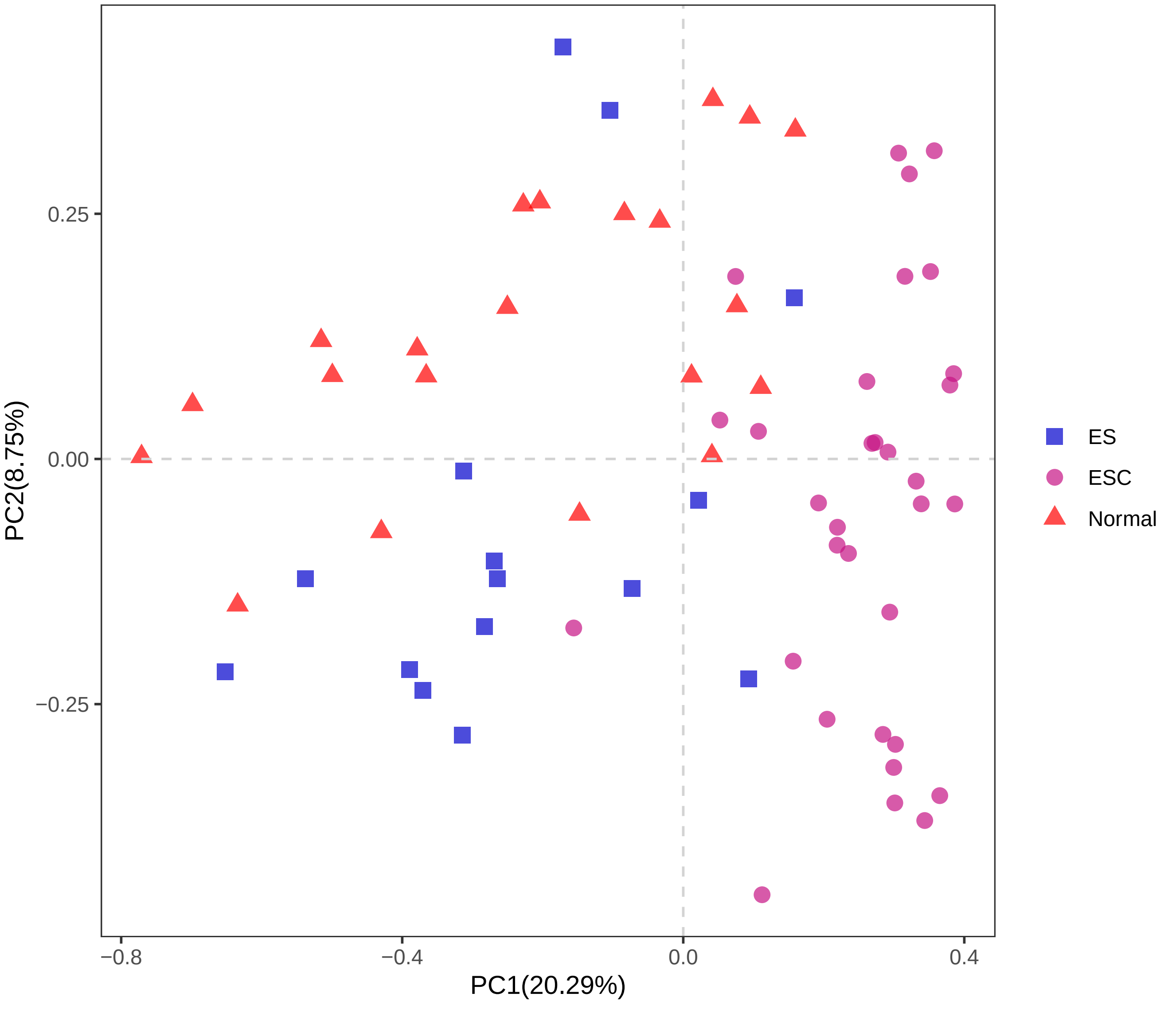
PC2(8.75%)
0.25
0.00
−0.25
−0.8
−0.4
0.0
0.4
PC1(20.29%)
ES
ESC
Normal
