# Supplementary material for: Characterization of Esophageal Microbiota in Patients With Esophagitis and Esophageal Squamous Cell Carcinoma
Source: Front Cell Infect Microbiol. 2021 Nov 11;11:774330. doi: 10.3389/fcimb.2021.774330 (PMC8632060; doi:10.3389/fcimb.2021.774330)
Supplement: Supplementary file 2 [file DataSheet_1.zip › 16S_V3_V4-68╕÷╤∙▒╛JZD 2020.7.21/4.Beta_diversity_analysis/PCA/OTUs_PCA_text.pdf]

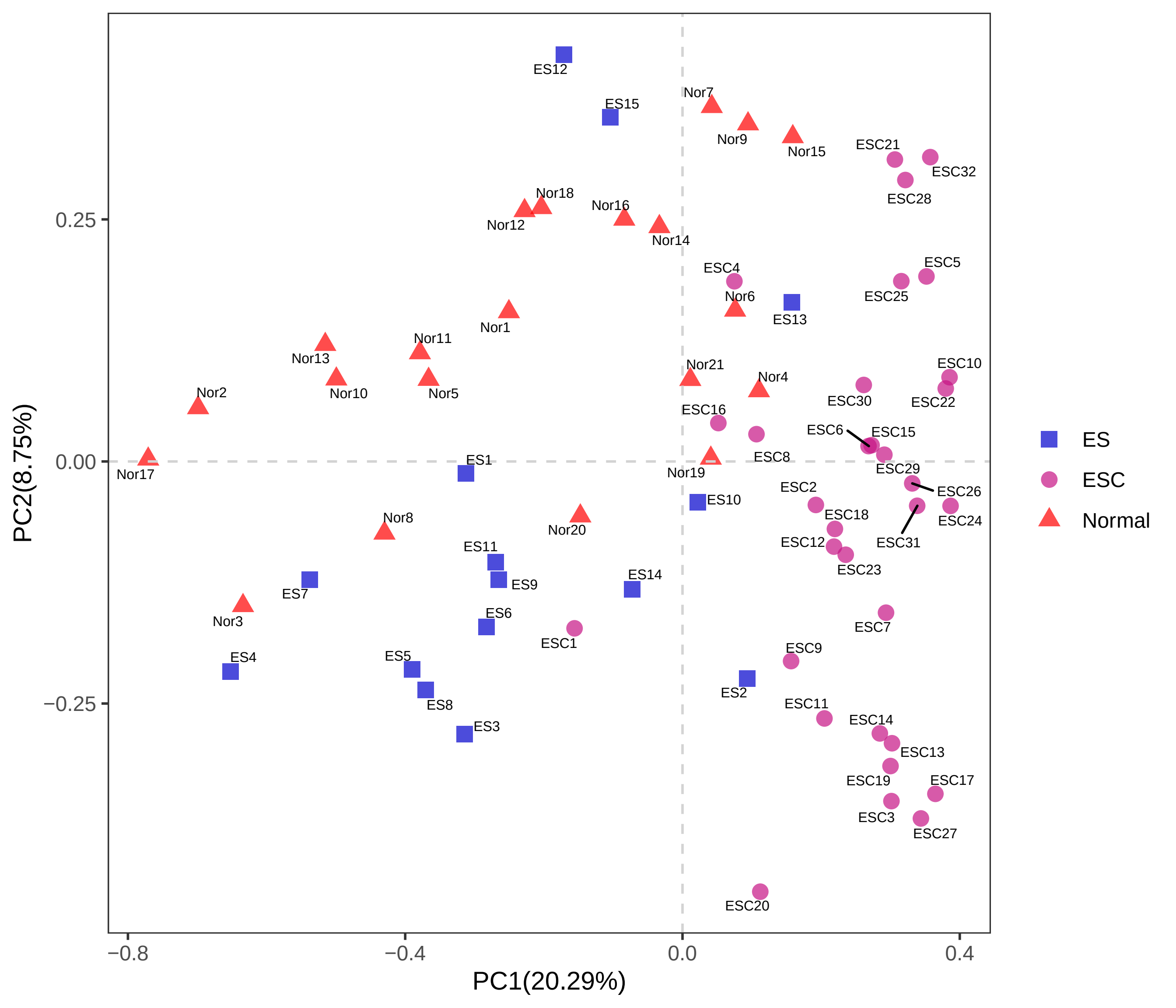

PC1(20.29%)
PC2(8.75%)
−0.8
−0.4
0.0
0.4
0.25
0.00
−0.25
ES
ESC
Normal
ES12
ES15
Nor7
Nor9
Nor15
ESC21
ESC32
ESC28
Nor18
Nor12
Nor16
Nor14
ESC4
ESC5
ESC25
Nor6
ES13
Nor1
Nor11
Nor13
Nor10
Nor5
Nor21
Nor4
ESC10
ESC30
ESC22
Nor2
ESC16
ESC8
ESC6
ESC15
ESC29
Nor17
ES1
Nor19
ESC2
ESC26
ES10
ESC18
ESC24
ESC31
ESC12
ESC23
Nor8
Nor20
ES11
ES9
ES7
ES14
Nor3
ES6
ESC1
ESC7
ESC9
ES4
ES5
ES8
ES2
ESC11
ESC14
ESC13
ES3
ESC19
ESC17
ESC3
ESC27
ESC20
