# Supplementary material for: Characterization of Esophageal Microbiota in Patients With Esophagitis and Esophageal Squamous Cell Carcinoma
Source: Front Cell Infect Microbiol. 2021 Nov 11;11:774330. doi: 10.3389/fcimb.2021.774330 (PMC8632060; doi:10.3389/fcimb.2021.774330)
Supplement: Supplementary file 2 [file DataSheet_1.zip › 16S_V3_V4-68╕÷╤∙▒╛JZD 2020.7.21/4.Beta_diversity_analysis/PLS-DA/OTUs_PLS-DA_text.pdf]

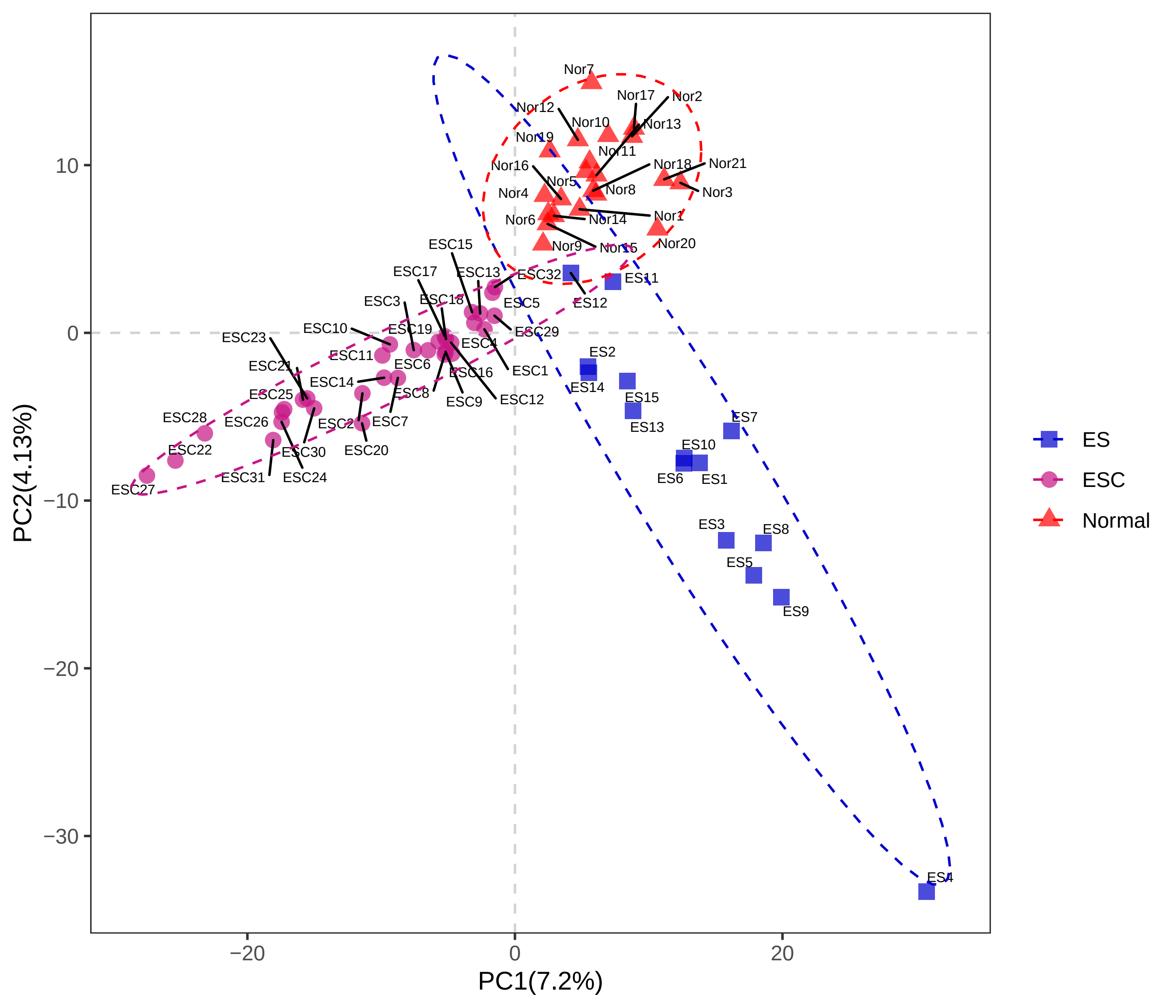

PC1(7.2%)
PC2(4.13%)
−20
0
20
10
−10
−30
ES
ESC
Normal
Nor7
Nor12
Nor17
Nor2
Nor10
Nor13
Nor19
Nor11
Nor16
Nor18
Nor21
Nor5
Nor4
Nor8
Nor3
Nor6
Nor14
Nor1
Nor9
Nor15
Nor20
ESC15
ESC17
ESC13
ESC32
ES11
ESC3
ESC18
ESC5
ES12
ESC10
ESC19
ESC29
ESC23
ESC4
ESC11
ESC6
ESC1
ESC21
ESC16
ESC14
ESC8
ESC9
ESC12
ESC25
ESC28
ESC26
ESC2
ESC7
ESC22
ESC30
ESC20
ESC31
ESC24
ESC27
ES2
ES14
ES15
ES13
ES7
ES10
ES6
ES1
ES3
ES8
ES5
ES9
ES4
