# Supplementary material for: Characterization of Esophageal Microbiota in Patients With Esophagitis and Esophageal Squamous Cell Carcinoma
Source: Front Cell Infect Microbiol. 2021 Nov 11;11:774330. doi: 10.3389/fcimb.2021.774330 (PMC8632060; doi:10.3389/fcimb.2021.774330)
Supplement: Supplementary file 2 [file DataSheet_1.zip › 16S_V3_V4-68╕÷╤∙▒╛JZD 2020.7.21/5.Statistical_test_analysis/Lefse/LDA.pdf]

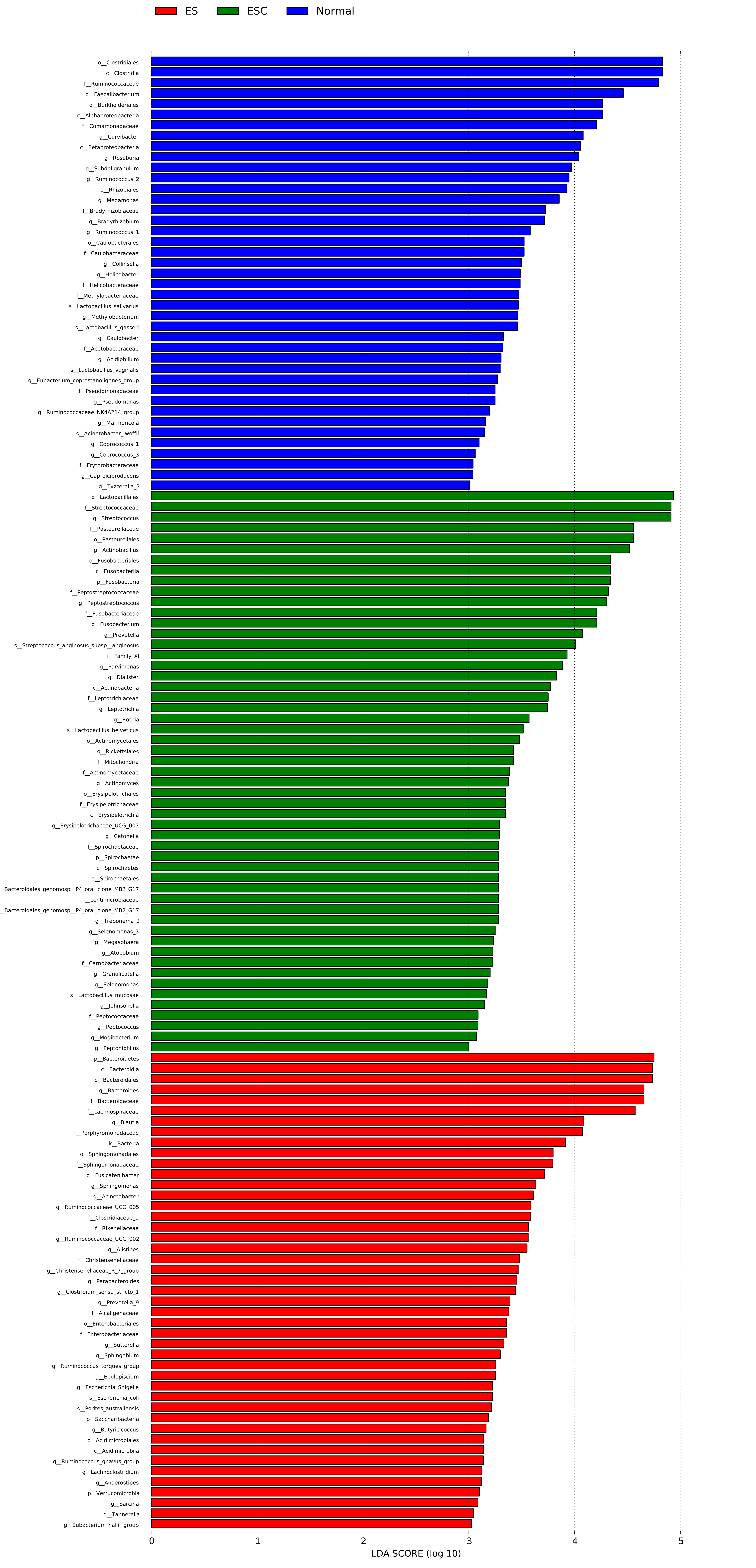
ES
ESC
Normal
o__Clostridiales
c__Clostridia
f__Ruminococcaceae
g__Faecalibacterium
o__Burkholderiales
c__Alphaproteobacteria
f__Comamonadaceae
g__Curvibacter
c__Betaproteobacteria
g__Roseburia
g__Subdoligranulum
g__Ruminococcus_2
o__Rhizobiales
g__Megamonas
f__Bradyrhizobiaceae
g__Bradyrhizobium
g__Ruminococcus_1
o__Caulobacterales
f__Caulobacteraceae
g__Collinsella
g__Helicobacter
f__Helicobacteraceae
f__Methylobacteriaceae
s__Lactobacillus_salivarius
g__Methylobacterium
s__Lactobacillus_gasseri
g__Caulobacter
f__Acetobacteraceae
g__Acidiphilium
s__Lactobacillus_vaginalis
g__Eubacterium_coprostanoligenes_group
f__Pseudomonadaceae
g__Pseudomonas
g__Ruminococcaceae_NK4A214_group
g__Marmoricola
s__Acinetobacter_lwoffii
g__Coprococcus_1
g__Coprococcus_3
f__Erythrobacteraceae
g__Caproiciproducens
g__Tyzzerella_3
o__Lactobacillales
f__Streptococcaceae
g__Streptococcus
f__Pasteurellaceae
o__Pasteurellales
g__Actinobacillus
o__Fusobacteriales
c__Fusobacteriia
p__Fusobacteria
f__Peptostreptococcaceae
g__Peptostreptococcus
f__Fusobacteriaceae
g__Fusobacterium
g__Prevotella
s__Streptococcus_anginosus_subsp__anginosus
f__Family_XI
g__Parvimonas
g__Dialister
c__Actinobacteria
f__Leptotrichiaceae
g__Leptotrichia
g__Rothia
s__Lactobacillus_helveticus
o__Actinomycetales
o__Rickettsiales
f__Mitochondria
f__Actinomycetaceae
g__Actinomyces
o__Erysipelotrichales
f__Erysipelotrichaceae
c__Erysipelotrichia
g__Erysipelotrichaceae_UCG_007
g__Catonella
f__Spirochaetaceae
p__Spirochaetae
c__Spirochaetes
o__Spirochaetales
Bacteroidales_genomosp__P4_oral_clone_MB2_G17
f__Lentimicrobiaceae
Bacteroidales_genomosp__P4_oral_clone_MB2_G17
g__Treponema_2
g__Selenomonas_3
g__Megasphaera
g__Atopobium
f__Carnobacteriaceae
g__Granulicatella
g__Selenomonas
s__Lactobacillus_mucosae
g__Johnsonella
f__Peptococcaceae
g__Peptococcus
g__Mogibacterium
g__Peptoniphilus
p__Bacteroidetes
c__Bacteroidia
o__Bacteroidales
g__Bacteroides
f__Bacteroidaceae
f__Lachnospiraceae
g__Blautia
f__Porphyromonadaceae
k__Bacteria
o__Sphingomonadales
f__Sphingomonadaceae
g__Fusicatenibacter
g__Sphingomonas
g__Acinetobacter
g__Ruminococcaceae_UCG_005
f__Clostridiaceae_1
f__Rikenellaceae
g__Ruminococcaceae_UCG_002
g__Alistipes
f__Christensenellaceae
g__Christensenellaceae_R_7_group
g__Parabacteroides
g__Clostridium_sensu_stricto_1
g__Prevotella_9
f__Alcaligenaceae
o__Enterobacteriales
f__Enterobacteriaceae
g__Sutterella
g__Sphingobium
g__Ruminococcus_torques_group
g__Epulopiscium
g__Escherichia_Shigella
s__Escherichia_coli
s__Porites_australiensis
p__Saccharibacteria
g__Butyricicoccus
o__Acidimicrobiales
c__Acidimicrobiia
g__Ruminococcus_gnavus_group
g__Lachnoclostridium
g__Anaerostipes
p__Verrucomicrobia
g__Sarcina
g__Tannerella
g__Eubacterium_hallii_group
0
1
2
3
4
5
LDA SCORE (log 10)
