# Supplementary material for: Characterization of Esophageal Microbiota in Patients With Esophagitis and Esophageal Squamous Cell Carcinoma
Source: Front Cell Infect Microbiol. 2021 Nov 11;11:774330. doi: 10.3389/fcimb.2021.774330 (PMC8632060; doi:10.3389/fcimb.2021.774330)
Supplement: Supplementary file 2 [file DataSheet_1.zip › 16S_V3_V4-68╕÷╤∙▒╛JZD 2020.7.21/5.Statistical_test_analysis/Lefse/cladogram.pdf]

Cladogram

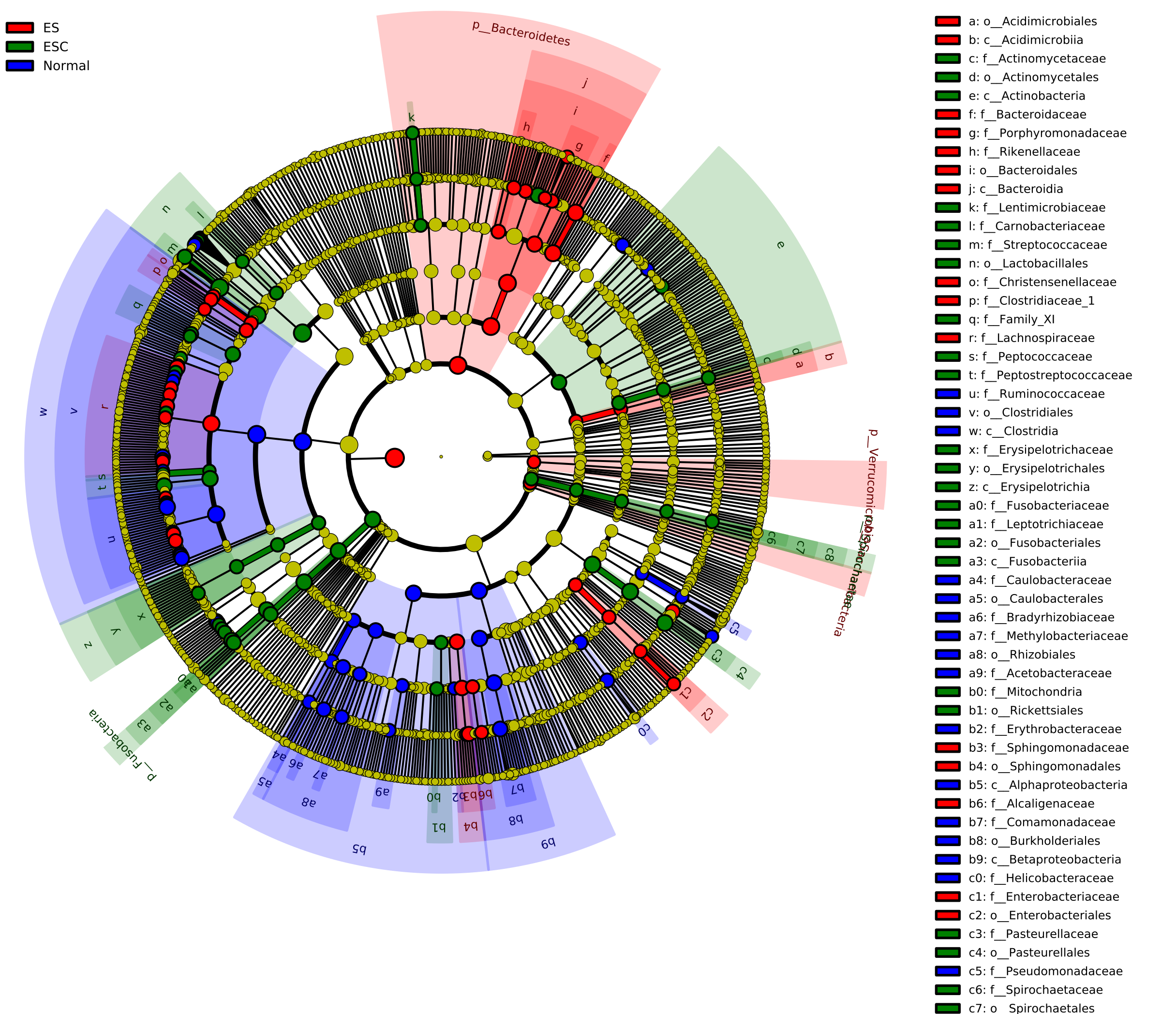

ES
ESC
Normal
p__Bacteroidetes
p__Fusobacteria
a: o__Acidimicrobiales
b: c__Acidimicrobiia
c: f__Actinomycetaceae
d: o__Actinomycetales
e: c__Actinobacteria
f: f__Bacteroidaceae
g: f__Porphyromonadaceae
h: f__Rikenellaceae
i: o__Bacteroidales
j: c__Bacteroidia
k: f__Lentimicrobiaceae
l: f__Carnobacteriaceae
m: f__Streptococcaceae
n: o__Lactobacillales
o: f__Christensenellaceae
p: f__Clostridiaceae_1
q: f__Family_XI
r: f__Lachnospiraceae
s: f__Peptococcaceae
t: f__Peptostreptococcaceae
u: f__Ruminococcaceae
v: o__Clostridiales
w: c__Clostridia
x: f__Erysipelotrichaceae
y: o__Erysipelotrichales
z: c__Erysipelotrichia
a0: f__Fusobacteriaceae
a1: f__Leptotrichiaceae
a2: o__Fusobacteriales
a3: c__Fusobacteriia
a4: f__Caulobacteraceae
a5: o__Caulobacterales
a6: f__Bradyrhizobiaceae
a7: f__Methylobacteriaceae
a8: o__Rhizobiales
a9: f__Acetobacteraceae
b0: f__Mitochondria
b1: o__Rickettsiales
b2: f__Erythrobacteraceae
b3: f__Sphingomonadaceae
b4: o__Sphingomonadales
b5: c__Alphaproteobacteria
b6: f__Alcaligenaceae
b7: f__Comamonadaceae
b8: o__Burkholderiales
b9: c__Betaproteobacteria
c0: f__Helicobacteraceae
c1: f__Enterobacteriaceae
c2: o__Enterobacteriales
c3: f__Pasteurellaceae
c4: o__Pasteurellales
c5: f__Pseudomonadaceae
c6: f__Spirochaetaceae
c7: o__Spirochaetales
