# Supplementary material for: Characterization of Esophageal Microbiota in Patients With Esophagitis and Esophageal Squamous Cell Carcinoma
Source: Front Cell Infect Microbiol. 2021 Nov 11;11:774330. doi: 10.3389/fcimb.2021.774330 (PMC8632060; doi:10.3389/fcimb.2021.774330)
Supplement: Supplementary file 2 [file DataSheet_1.zip › 16S_V3_V4-68╕÷╤∙▒╛JZD 2020.7.21/5.Statistical_test_analysis/Wilcoxon/Class/diff_Normal-ESC.txt.pdf]

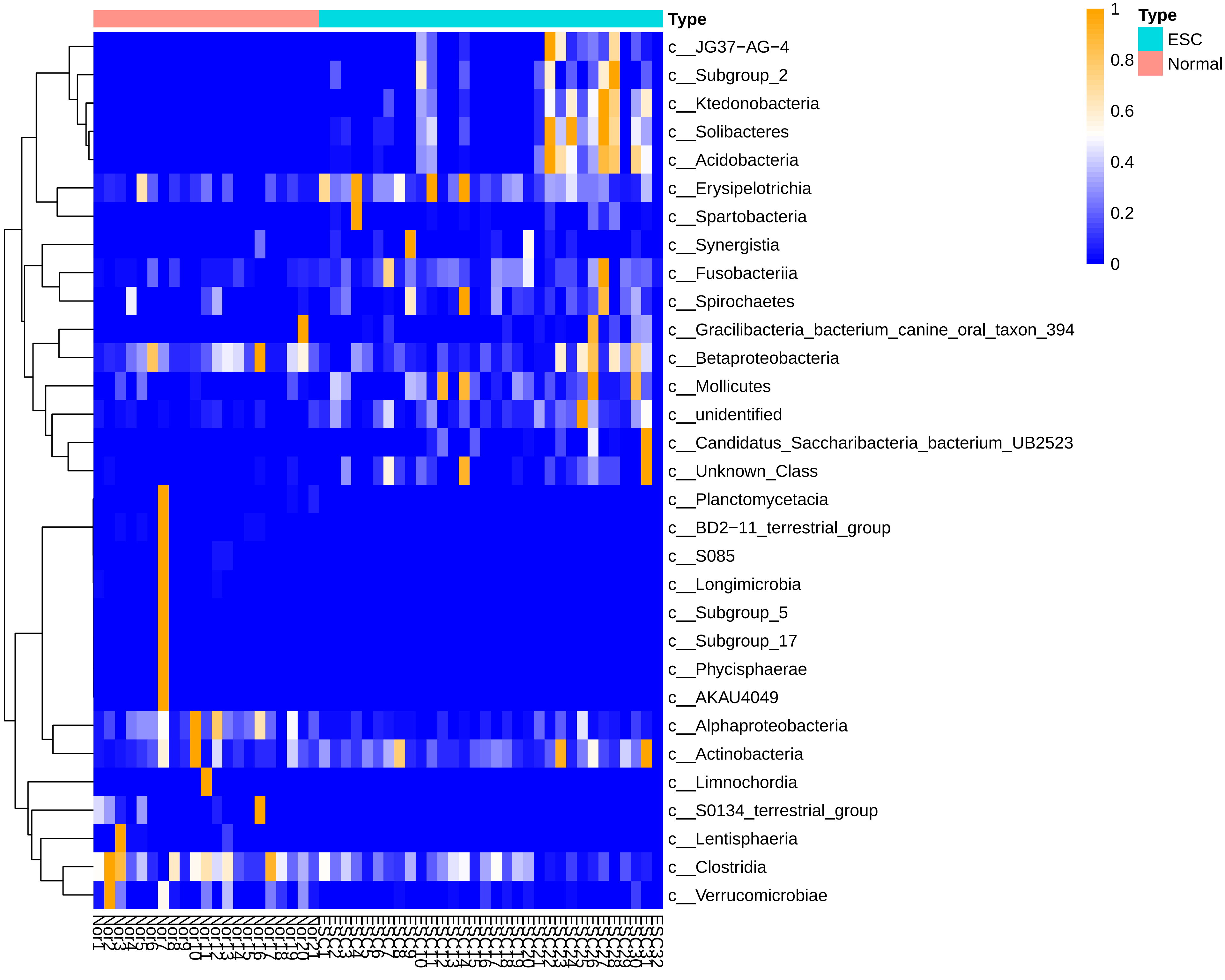

Type
c__JG37−AG−4
c__Subgroup_2
c__Ktedonobacteria
c__Solibacteres
c__Acidobacteria
c__Erysipelotrichia
c__Spartobacteria
c__Synergistia
c__Fusobacteriia
c__Spirochaetes
c__Gracilibacteria_bacterium_canine_oral_taxon_394
c__Betaproteobacteria
c__Mollicutes
c__unidentified
c__Candidatus_Saccharibacteria_bacterium_UB2523
c__Unknown_Class
c__Planctomycetacia
c__BD2−11_terrestrial_group
c__S085
c__Longimicrobia
c__Subgroup_5
c__Subgroup_17
c__Phycisphaerae
c__AKAU4049
c__Alphaproteobacteria
c__Actinobacteria
c__Limnochordia
c__S0134_terrestrial_group
c__Lentisphaeria
c__Clostridia
c__Verrucomicrobiae
1
0.8
0.6
0.4
0.2
0
Type
ESC
Normal
Nor1 Nor2 Nor3 Nor4 Nor5 Nor6 Nor7 Nor8 Nor9 Nor10 Nor11 Nor12 Nor13 Nor14 Nor15 Nor16 Nor17 Nor18 Nor19 Nor20 Nor21
ESC1 ESC2 ESC3 ESC4 ESC5 ESC6 ESC7 ESC8 ESC9 ESC10 ESC11 ESC12 ESC13 ESC14 ESC15 ESC16 ESC17 ESC18 ESC19 ESC20 ESC21 ESC22 ESC23 ESC24 ESC25 ESC26 ESC27 ESC28 ESC29 ESC30 ESC31 ESC32
