# Supplementary material for: Characterization of Esophageal Microbiota in Patients With Esophagitis and Esophageal Squamous Cell Carcinoma
Source: Front Cell Infect Microbiol. 2021 Nov 11;11:774330. doi: 10.3389/fcimb.2021.774330 (PMC8632060; doi:10.3389/fcimb.2021.774330)
Supplement: Supplementary file 2 [file DataSheet_1.zip › 16S_V3_V4-68╕÷╤∙▒╛JZD 2020.7.21/5.Statistical_test_analysis/Wilcoxon/Class/errbar_Normal-ESC_errbar.pdf]

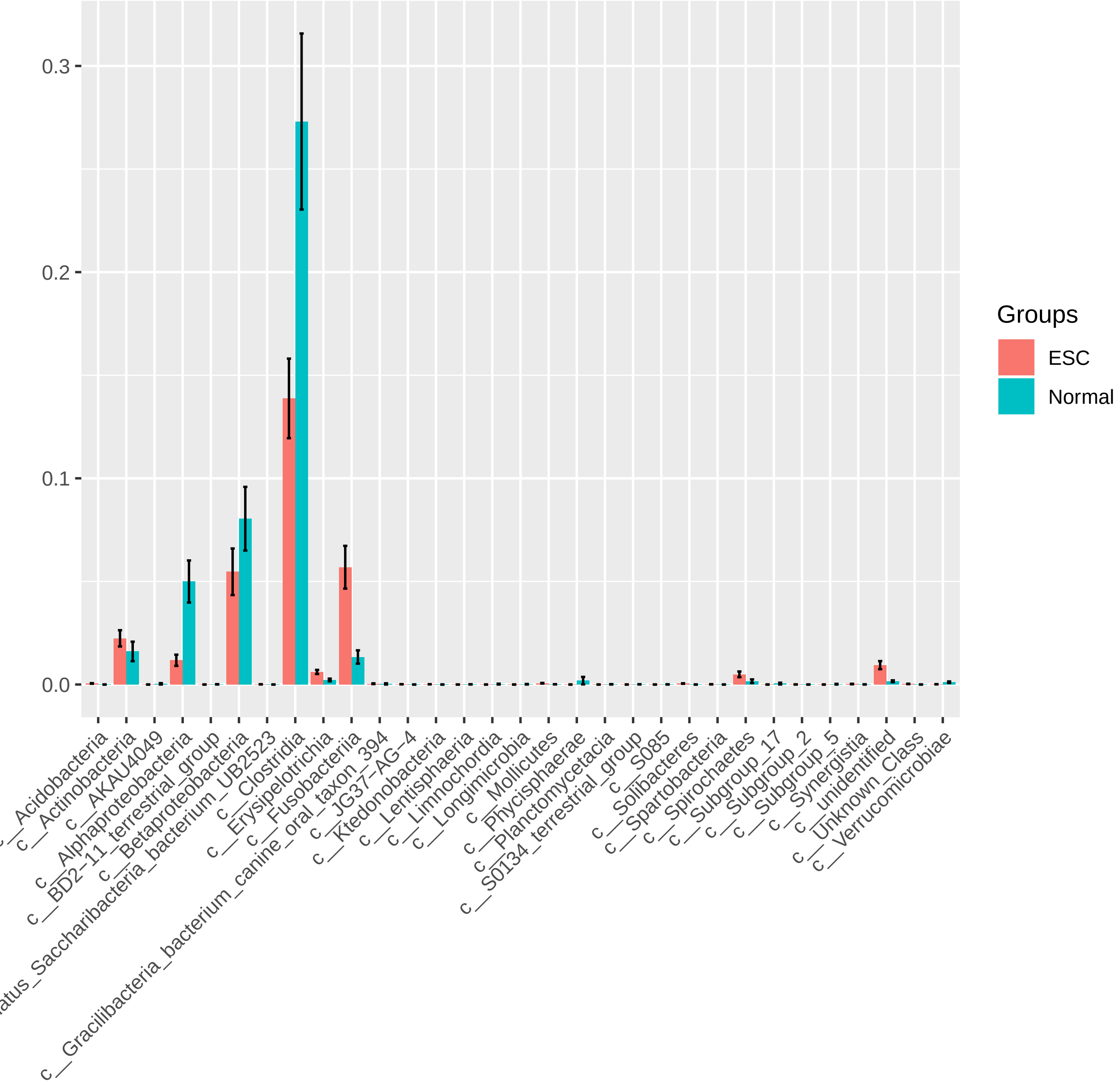

0.3
0.2
0.1
0.0
Groups
ESC
Normal
c__Acidobacteria
c__Actinobacteria
c__AKAU4049
c__Alphaproteobacteria
c__BD2-11_terrestrial_group
c__Betaproteobacteria
c__Clostridia
c__Erysipelotrichia
c__Fusobacteriia
c__Gracilibacteria_bacterium_canine_oral_taxon_394
c__JG37-AG-4
c__Ktedonobacteria
c__Lentisphaeria
c__Limnochordia
c__Longimicrobia
c__Mollicutes
c__Phycisphaerae
c__Planctomycetacia
c__S0134_terrestrial_group
c__S085
c__Solibacteres
c__Spartobacteria
c__Spirochaetes
c__Subgroup_17
c__Subgroup_2
c__Subgroup_5
c__Synergistia
c__unidentified
c__Unknown_Class
c__Verrucomicrobiae
