# Supplementary material for: Characterization of Esophageal Microbiota in Patients With Esophagitis and Esophageal Squamous Cell Carcinoma
Source: Front Cell Infect Microbiol. 2021 Nov 11;11:774330. doi: 10.3389/fcimb.2021.774330 (PMC8632060; doi:10.3389/fcimb.2021.774330)
Supplement: Supplementary file 2 [file DataSheet_1.zip › 16S_V3_V4-68╕÷╤∙▒╛JZD 2020.7.21/5.Statistical_test_analysis/Wilcoxon/Class/errbar_Normal-ES_errbar.pdf]

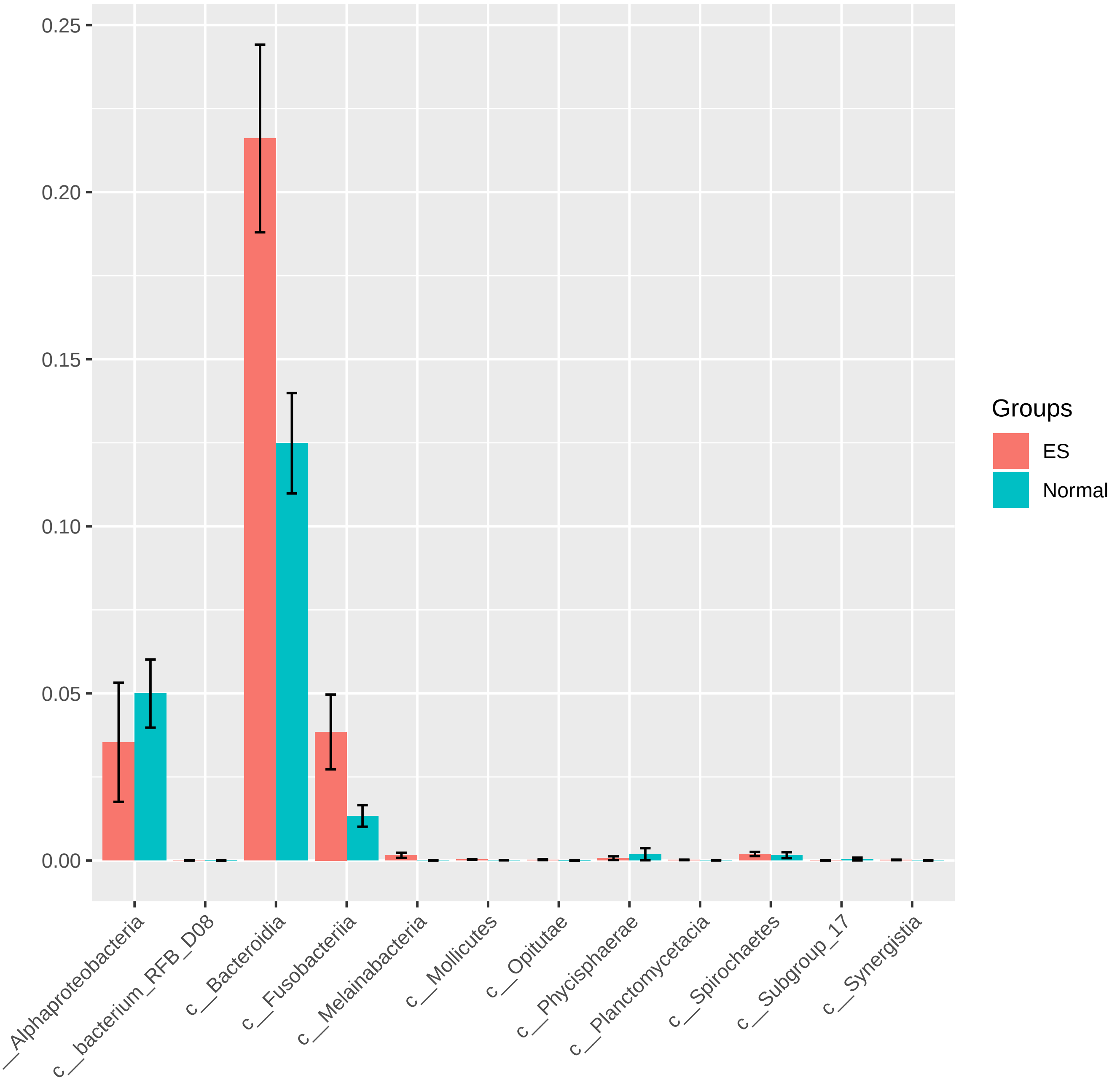

0.25
0.20
0.15
0.10
0.05
0.00
Groups
ES
Normal
__Alphaproteobacteria
c__bacterium_RFB_D08
c__Bacteroidia
c__Fusobacteriia
c__Melainabacteria
c__Mollicutes
c__Opitutae
c__Phycisphaerae
c__Planctomycetacia
c__Spirochaetes
c__Subgroup_17
c__Synergistia
