# Supplementary material for: Characterization of Esophageal Microbiota in Patients With Esophagitis and Esophageal Squamous Cell Carcinoma
Source: Front Cell Infect Microbiol. 2021 Nov 11;11:774330. doi: 10.3389/fcimb.2021.774330 (PMC8632060; doi:10.3389/fcimb.2021.774330)
Supplement: Supplementary file 2 [file DataSheet_1.zip › 16S_V3_V4-68╕÷╤∙▒╛JZD 2020.7.21/5.Statistical_test_analysis/Wilcoxon/Family/diff_ES-ESC.txt.pdf]

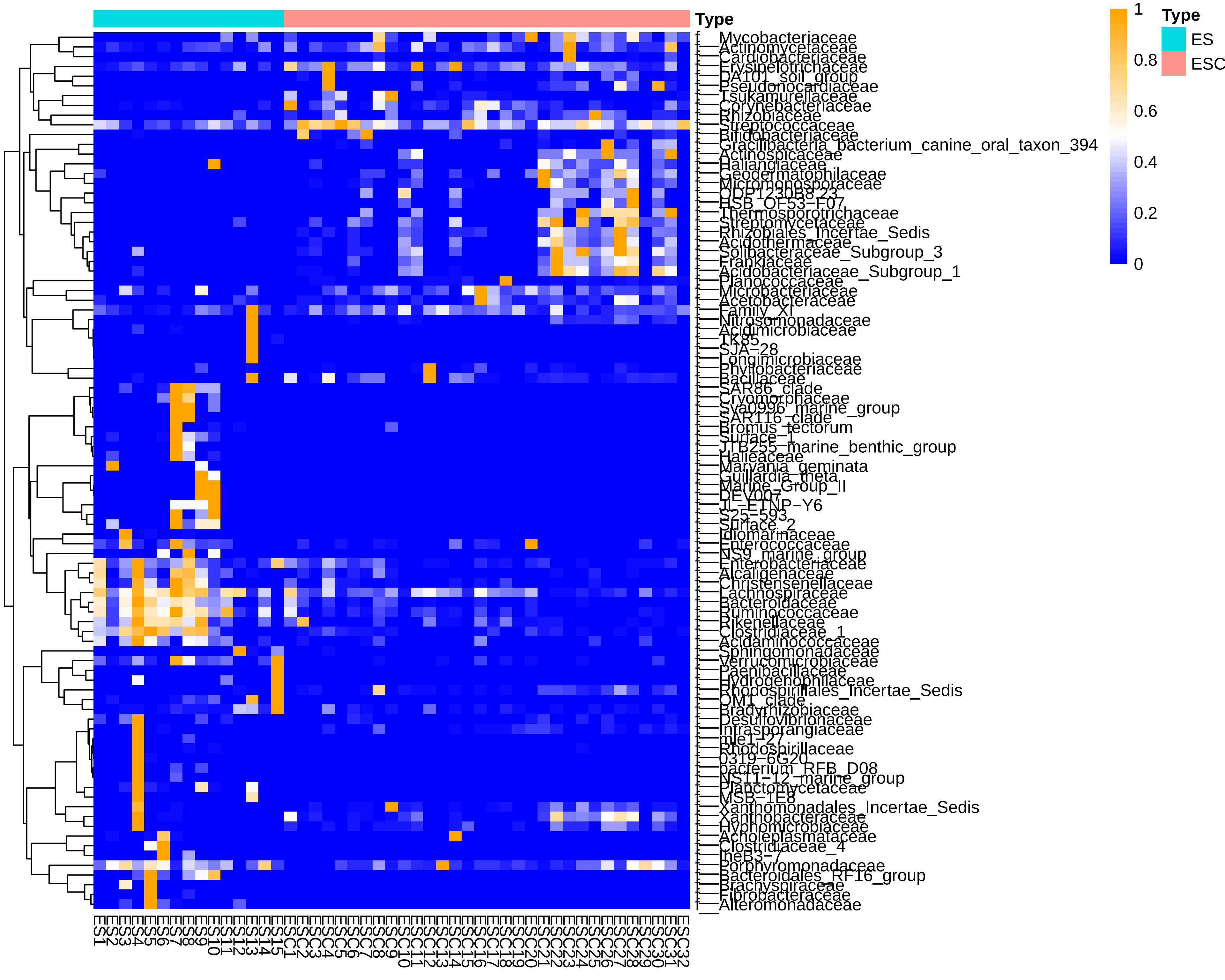
Type
1
0.8
0.6
0.4
0.2
0
Type
ES
ESC
Mycobacteriaceae
Actinomycetaceae
Cardiobacteriaceae
Erysipelotrichaceae
DA101_soil_group
Pseudonocardiaceae
Tsukamurellaceae
Corynebacteriaceae
Rhizobiaceae
Streptococcaceae
Bifidobacteriaceae
Gracilibacteria_bacterium_canine_oral_taxon_394
Actinospicaceae
Haliangiaceae
Geodermatophilaceae
Micromonosporaceae
ODP1230B8.23
HSB_OF53−F07
Thermosporotrichaceae
Streptomycetaceae
Rhizobiales_Incertae_Sedis
Acidothermaceae
Solibacteraceae_Subgroup_3
Frankiaceae
Acidobacteriaceae_Subgroup_1
Planococcaceae
Microbacteriaceae
Acetobacteraceae
Family_XI
Nitrosomonadaceae
Acidimicrobiaceae
TK85
SJA−28
Longimicrobiaceae
Phyllobacteriaceae
Bacillaceae
SAR86_clade
Cryomorphaceae
Sva0996_marine_group
SAR116_clade
Bromus_tectorum
Surface_1
JTB255−marine_benthic_group
Halieaceae
Marvania_geminata
Guillardia_theta
Marine_Group_II
DEV007
JL−ETNP−Y6
S25−593
Surface_2
Idiomarinaceae
Enterococcaceae
NS9_marine_group
Enterobacteriaceae
Alcaligenaceae
Christensenellaceae
Lachnospiraceae
Bacteroidaceae
Ruminococcaceae
Rikenellaceae
Clostridiaceae_1
Acidaminococcaceae
Sphingomonadaceae
Verrucomicrobiaceae
Paenibacillaceae
Hydrogenophilaceae
Rhodospirillales_Incertae_Sedis
OM1_clade
Bradyrhizobiaceae
Desulfovibrionaceae
Intrasporangiaceae
mle1−27
Rhodospirillaceae
0319−6G20
bacterium_RFB_D08
NS11−12_marine_group
Planctomycetaceae
MSB−1E8
Xanthomonadales_Incertae_Sedis
Xanthobacteraceae
Hyphomicrobiaceae
Acholeplasmataceae
Clostridiaceae_4
TheB3−7
Porphyromonadaceae
Bacteroidales_RF16_group
Brachyspiraceae
Fibrobacteraceae
Alteromonadaceae
ES1
ES2
ES3
ES4
ES5
ES6
ES7
ES8
ES9
ES10
ES11
ES12
ES13
ES14
ES15
ESC1
ESC2
ESC3
ESC4
ESC5
ESC6
ESC7
ESC8
ESC9
ESC10
ESC11
ESC12
ESC13
ESC14
ESC15
ESC16
ESC17
ESC18
ESC19
ESC20
ESC21
ESC22
ESC23
ESC24
ESC25
ESC26
ESC27
ESC28
ESC29
ESC30
ESC31
ESC32
