# Supplementary material for: Characterization of Esophageal Microbiota in Patients With Esophagitis and Esophageal Squamous Cell Carcinoma
Source: Front Cell Infect Microbiol. 2021 Nov 11;11:774330. doi: 10.3389/fcimb.2021.774330 (PMC8632060; doi:10.3389/fcimb.2021.774330)
Supplement: Supplementary file 2 [file DataSheet_1.zip › 16S_V3_V4-68╕÷╤∙▒╛JZD 2020.7.21/5.Statistical_test_analysis/Wilcoxon/Family/diff_Normal-ES.txt.pdf]

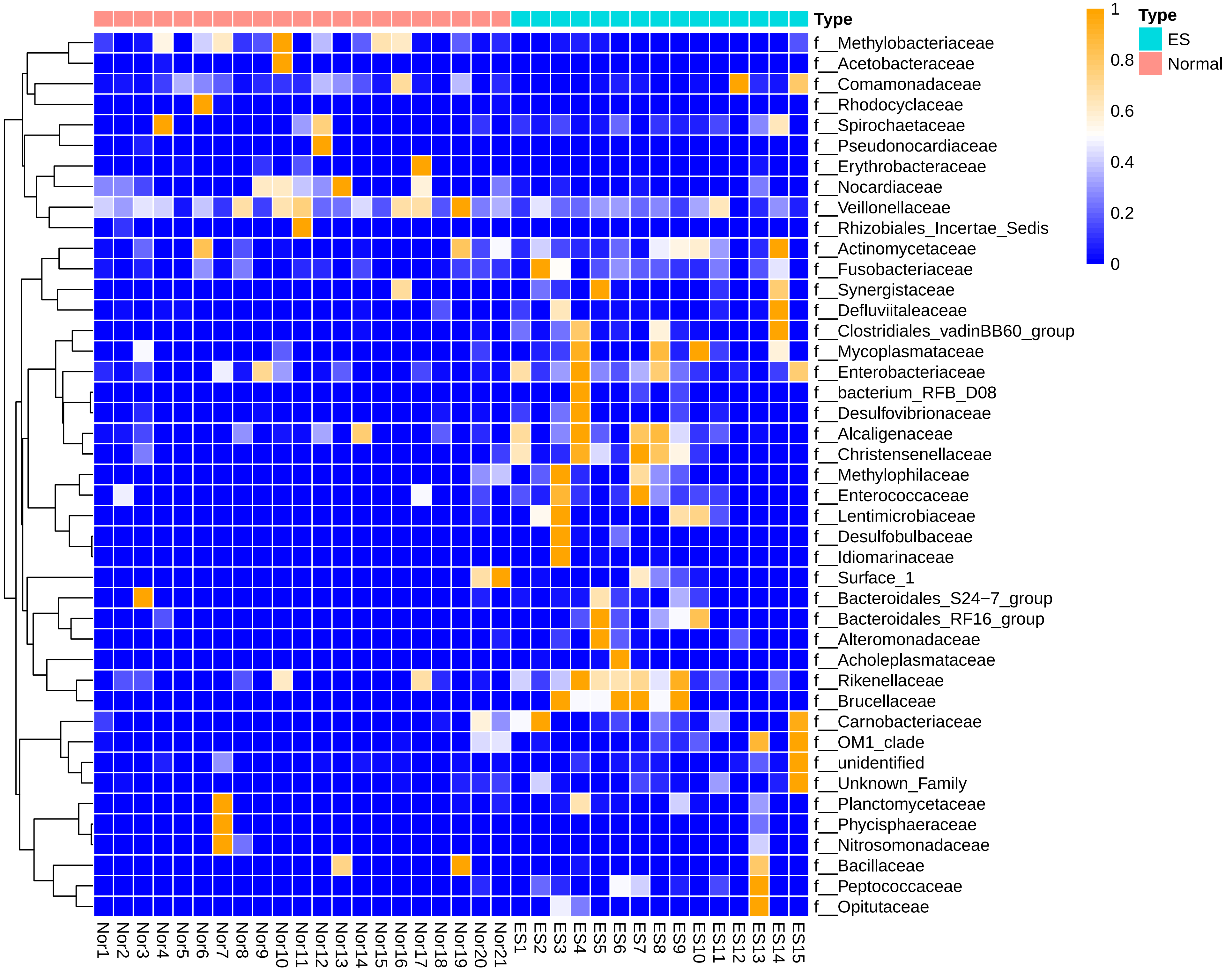
Type
f__Methylobacteriaceae
f__Acetobacteraceae
f__Comamonadaceae
f__Rhodocyclaceae
f__Spirochaetaceae
f__Pseudonocardiaceae
f__Erythrobacteraceae
f__Nocardiaceae
f__Veillonellaceae
f__Rhizobiales_Incertae_Sedis
f__Actinomycetaceae
f__Fusobacteriaceae
f__Synergistaceae
f__Defluviitaleaceae
f__Clostridiales_vadinBB60_group
f__Mycoplasmataceae
f__Enterobacteriaceae
f__bacterium_RFB_D08
f__Desulfovibrionaceae
f__Alcaligenaceae
f__Christensenellaceae
f__Methylophilaceae
f__Enterococcaceae
f__Lentimicrobiaceae
f__Desulfobulbaceae
f__Idiomarinaceae
f__Surface_1
f__Bacteroidales_S24−7_group
f__Bacteroidales_RF16_group
f__Alteromonadaceae
f__Acholeplasmataceae
f__Rikenellaceae
f__Brucellaceae
f__Carnobacteriaceae
f__OM1_clade
f__unidentified
f__Unknown_Family
f__Planctomycetaceae
f__Phycisphaeraceae
f__Nitrosomonadaceae
f__Bacillaceae
f__Peptococcaceae
f__Opitutaceae
Nor1
Nor2
Nor3
Nor4
Nor5
Nor6
Nor7
Nor8
Nor9
Nor10
Nor11
Nor12
Nor13
Nor14
Nor15
Nor16
Nor17
Nor18
Nor19
Nor20
Nor21
ES1
ES2
ES3
ES4
ES5
ES6
ES7
ES8
ES9
ES10
ES11
ES12
ES13
ES14
ES15
1
0.8
0.6
0.4
0.2
0
Type
ES
Normal
