# Supplementary material for: Characterization of Esophageal Microbiota in Patients With Esophagitis and Esophageal Squamous Cell Carcinoma
Source: Front Cell Infect Microbiol. 2021 Nov 11;11:774330. doi: 10.3389/fcimb.2021.774330 (PMC8632060; doi:10.3389/fcimb.2021.774330)
Supplement: Supplementary file 2 [file DataSheet_1.zip › 16S_V3_V4-68╕÷╤∙▒╛JZD 2020.7.21/5.Statistical_test_analysis/Wilcoxon/Family/diff_Normal-ESC.txt.pdf]

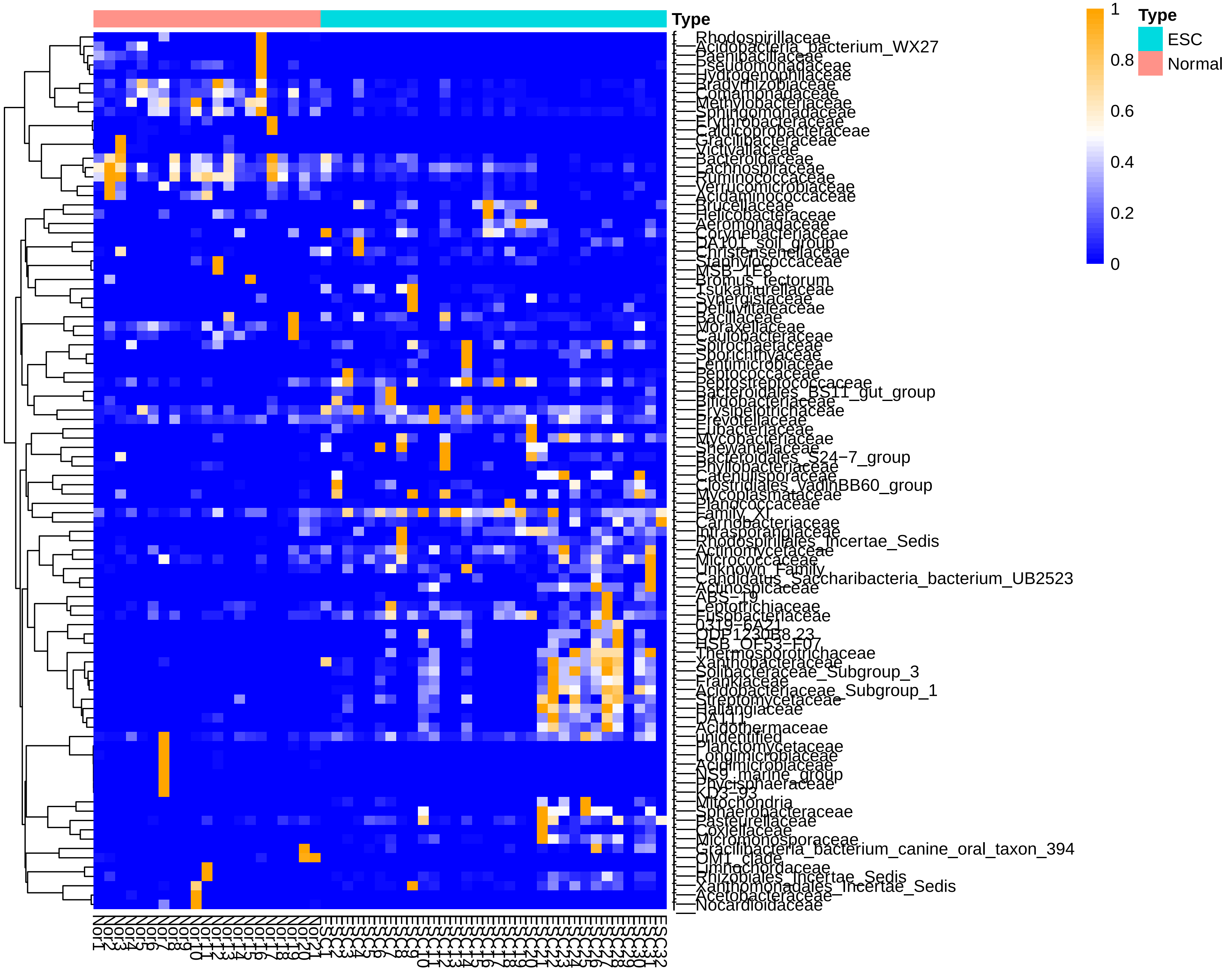

Type
Type
ESC
Normal
1
0.8
0.6
0.4
0.2
0
f__Rhodospirillaceae
f__Acidobacteria_bacterium_WX27
f__Paenibacillaceae
f__Pseudomonadaceae
f__Hydrogenophilaceae
f__Bradyrhizobiaceae
f__Comamonadaceae
f__Methylobacteriaceae
f__Sphingomonadaceae
f__Erythrobacteraceae
f__Caldicoprobacteraceae
f__Gracilibacteraceae
f__Victivallaceae
f__Bacteroidaceae
f__Lachnospiraceae
f__Ruminococcaceae
f__Verrucomicrobiaceae
f__Acidaminococcaceae
f__Brucellaceae
f__Helicobacteraceae
f__Aeromonadaceae
f__Corynebacteriaceae
f__DA101_soil_group
f__Christensenellaceae
f__Staphylococcaceae
f__MSB−1E8
f__Bromus_tectorum
f__Tsukamurellaceae
f__Synergistaceae
f__Defluviitaleaceae
f__Bacillaceae
f__Moraxellaceae
f__Caulobacteraceae
f__Spirochaetaceae
f__Sporichthyaceae
f__Lentimicrobiaceae
f__Peptococcaceae
f__Peptostreptococcaceae
f__Bacteroidales_BS11_gut_group
f__Bifidobacteriaceae
f__Erysipelotrichaceae
f__Prevotellaceae
f__Eubacteriaceae
f__Mycobacteriaceae
f__Shewanellaceae
f__Bacteroidales_S24−7_group
f__Phyllobacteriaceae
f__Catenulisporaceae
f__Clostridiales_vadinBB60_group
f__Mycoplasmataceae
f__Planococcaceae
f__Family_XI
f__Carnobacteriaceae
f__Intrasporangiaceae
f__Rhodospirillales_Incertae_Sedis
f__Actinomycetaceae
f__Micrococcaceae
f__Unknown_Family
f__Candidatus_Saccharibacteria_bacterium_UB2523
f__Actinospicaceae
f__ABS−19
f__Leptotrichiaceae
f__Fusobacteriaceae
f__0319−6A21
f__ODP1230B8.23
f__HSB_OF53−F07
f__Thermosporotrichaceae
f__Xanthobacteraceae
f__Solibacteraceae_Subgroup_3
f__Frankiaceae
f__Acidobacteriaceae_Subgroup_1
f__Streptomycetaceae
f__Haliangiaceae
f__DA111
f__Acidothermaceae
f__unidentified
f__Planctomycetaceae
f__Longimicrobiaceae
f__Acidimicrobiaceae
f__NS9_marine_group
f__Phycisphaeraceae
f__KD3−93
f__Mitochondria
f__Sphaerobacteraceae
f__Pasteurellaceae
f__Coxiellaceae
f__Micromonosporaceae
f__Gracilibacteria_bacterium_canine_oral_taxon_394
f__OM1_clade
f__Limnochordaceae
f__Rhizobiales_Incertae_Sedis
f__Xanthomonadales_Incertae_Sedis
f__Acetobacteraceae
f__Nocardioidaceae
Nor1 Nor2 Nor3 Nor4 Nor5 Nor6 Nor7 Nor8 Nor9 Nor10 Nor11 Nor12 Nor13 Nor14 Nor15 Nor16 Nor17 Nor18 Nor19 Nor20
ESC1 ESC2 ESC3 ESC4 ESC5 ESC6 ESC7 ESC8 ESC9 ESC10 ESC11 ESC12 ESC13 ESC14 ESC15 ESC16 ESC17 ESC18 ESC19 ESC20 ESC21 ESC22 ESC23 ESC24 ESC25 ESC26 ESC27 ESC28 ESC29 ESC30 ESC31 ESC32
