# Supplementary material for: Characterization of Esophageal Microbiota in Patients With Esophagitis and Esophageal Squamous Cell Carcinoma
Source: Front Cell Infect Microbiol. 2021 Nov 11;11:774330. doi: 10.3389/fcimb.2021.774330 (PMC8632060; doi:10.3389/fcimb.2021.774330)
Supplement: Supplementary file 2 [file DataSheet_1.zip › 16S_V3_V4-68╕÷╤∙▒╛JZD 2020.7.21/5.Statistical_test_analysis/Wilcoxon/Family/errbar_Normal-ES_errbar.pdf]

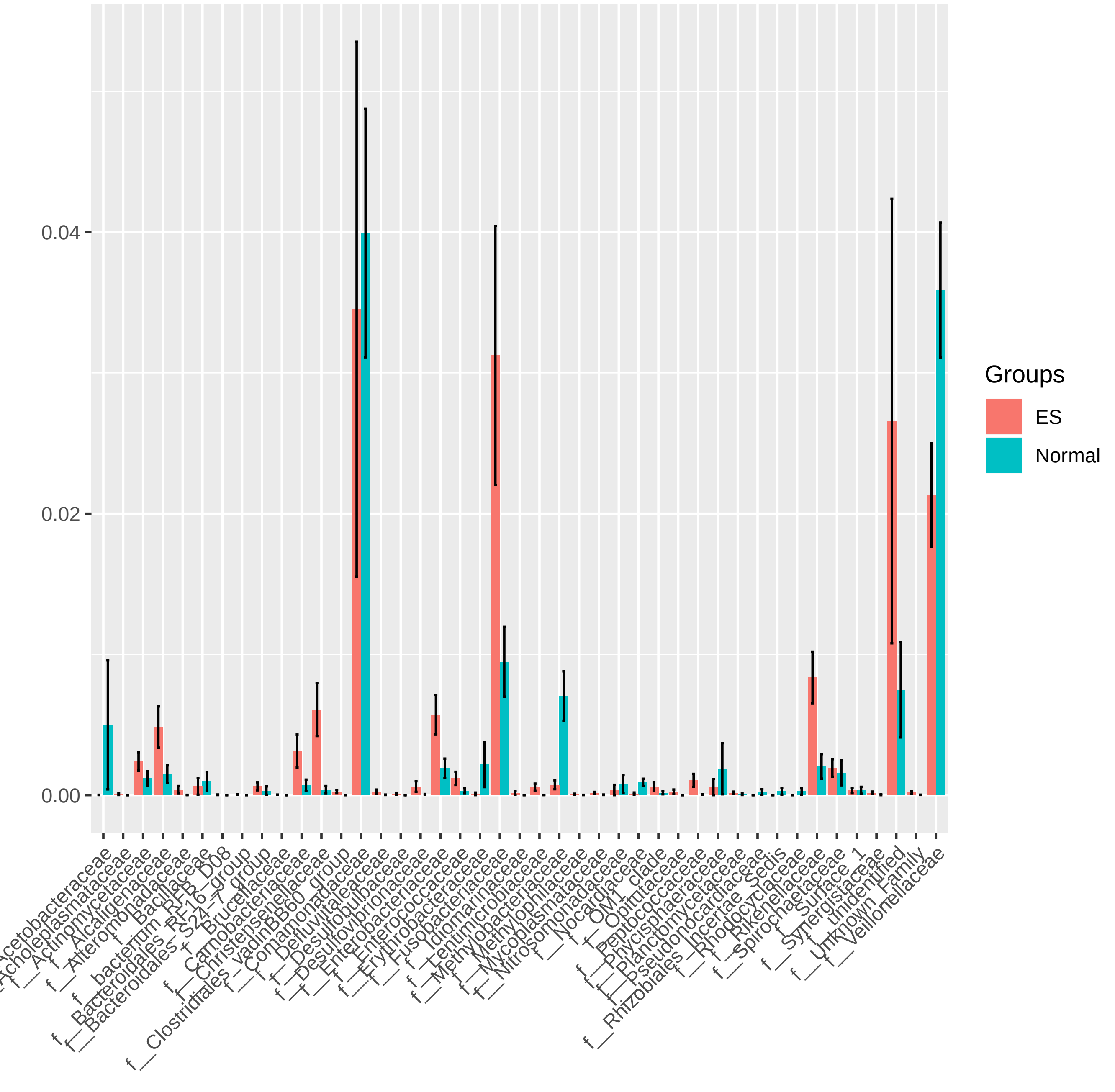

0.04
0.02
0.00
Groups
ES
Normal
f__Acetobacteraceae
f__Acholeplasmataceae
f__Actinomycetaceae
f__Alcaligenaceae
f__Alteromonadaceae
f__Bacillaceae
f__bacterium_RFB_D08
f__Bacteroidales_RF16_group
f__Bacteroidales_S24-7_group
f__Brucellaceae
f__Carnobacteriaceae
f__Christensenellaceae
f__Clostridiales_vadinBB60_group
f__Comamonadaceae
f__Defluviitaleaceae
f__Desulfobulbaceae
f__Desulfovibrionaceae
f__Enterobacteriaceae
f__Enterococcaceae
f__Erythrobacteraceae
f__Fusobacteriaceae
f__Idiomarinaceae
f__Lentimicrobiaceae
f__Methylobacteriaceae
f__Methylophilaceae
f__Mycoplasmataceae
f__Nitrosomonadaceae
f__Nocardiaceae
f__OM1_clade
f__Opitutaceae
f__Peptococcaceae
f__Phycisphaeraceae
f__Planctomycetaceae
f__Pseudonocardiaceae
f__Rhizobiales_Incertae_Sedis
f__Rhodocyclaceae
f__Rikenellaceae
f__Spirochaetaceae
f__Surface_1
f__Synergistaceae
f__unidentified
f__Unknown_Family
f__Veillonellaceae
