# Supplementary material for: Characterization of Esophageal Microbiota in Patients With Esophagitis and Esophageal Squamous Cell Carcinoma
Source: Front Cell Infect Microbiol. 2021 Nov 11;11:774330. doi: 10.3389/fcimb.2021.774330 (PMC8632060; doi:10.3389/fcimb.2021.774330)
Supplement: Supplementary file 2 [file DataSheet_1.zip › 16S_V3_V4-68╕÷╤∙▒╛JZD 2020.7.21/5.Statistical_test_analysis/Wilcoxon/Genus/diff_Normal-ES.txt.pdf]

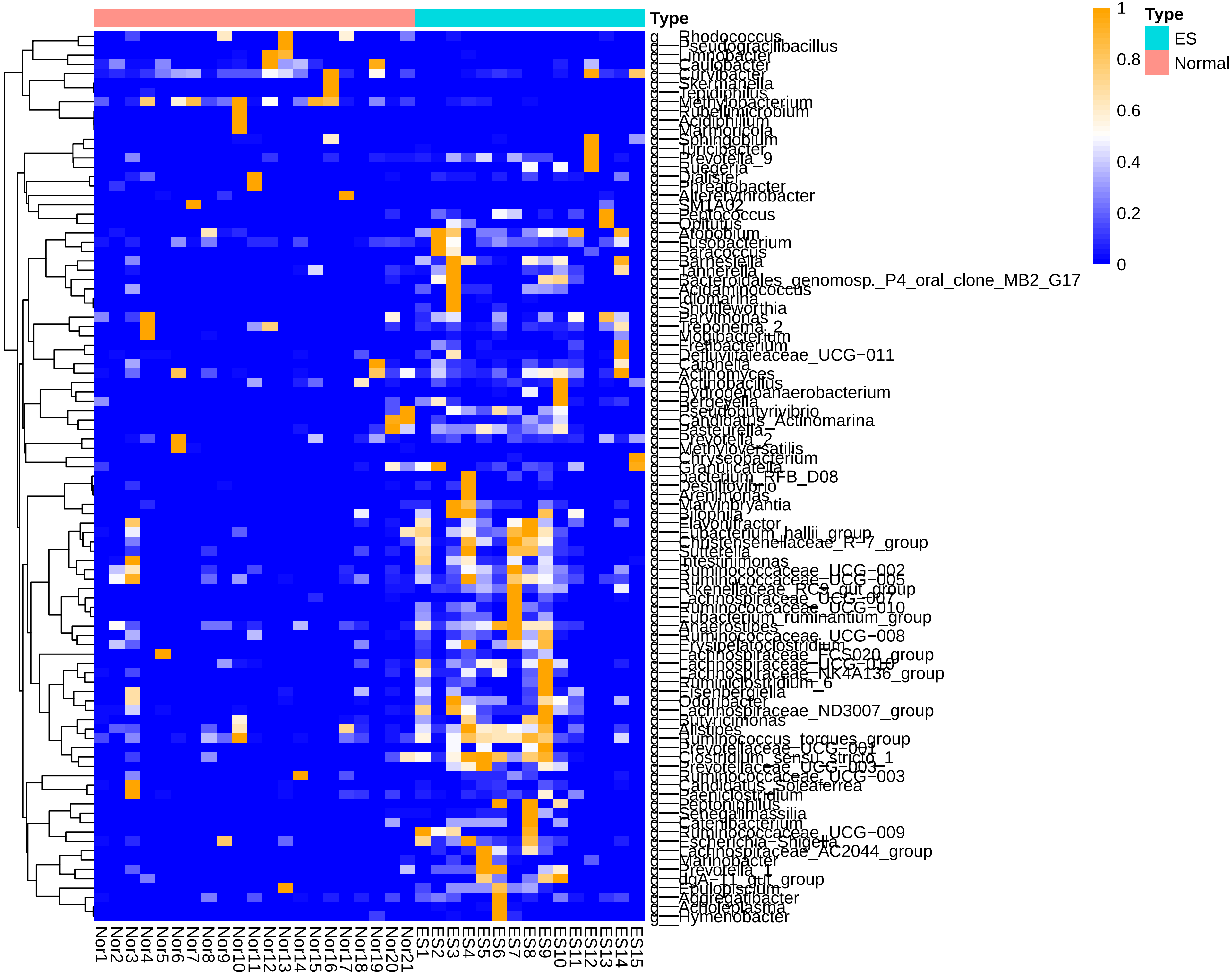
Type
1
0.8
0.6
0.4
0.2
0
Type
ES
Normal
g__Rhodococcus
g__Pseudogracilibacillus
g__Limnobacter
g__Caulobacter
g__Curvibacter
g__Skermanella
g__Tepidiphilus
g__Methylobacterium
g__Rubellimicrobium
g__Acidiphilium
g__Marmoricola
g__Sphingobium
g__Turicibacter
g__Prevotella_9
g__Ruegeria
g__Dialister
g__Phreatobacter
g__Altererythrobacter
g__SM1A02
g__Peptococcus
g__Opitutus
g__Atopobium
g__Fusobacterium
g__Paracoccus
g__Barnesiella
g__Tannerella
g__Bacteroidales_genomosp._P4_oral_clone_MB2_G17
g__Acidaminococcus
g__Idiomarina
g__Shuttleworthia
g__Parvimonas
g__Treponema_2
g__Mogibacterium
g__Fretibacterium
g__Defluviitaleaceae_UCG−011
g__Catonella
g__Actinomyces
g__Actinobacillus
g__Hydrogenoanaerobacterium
g__Bergeyella
g__Pseudobutyrivibrio
g__Candidatus_Actinomarina
g__Pasteurella
g__Prevotella_2
g__Methyloversatilis
g__Chryseobacterium
g__Granulicatella
g__bacterium_RFB_D08
g__Desulfovibrio
g__Arenimonas
g__Marvinbryantia
g__Bilophila
g__Flavonifractor
g__Eubacterium_hallii_group
g__Christensenellaceae_R−7_group
g__Sutterella
g__Intestinimonas
g__Ruminococcaceae_UCG−002
g__Ruminococcaceae_UCG−005
g__Rikenellaceae_RC9_gut_group
g__Lachnospiraceae_UCG−007
g__Ruminococcaceae_UCG−010
g__Eubacterium_ruminantium_group
g__Anaerostipes
g__Ruminococcaceae_UCG−008
g__Erysipelatoclostridium
g__Lachnospiraceae_FCS020_group
g__Lachnospiraceae_UCG−010
g__Lachnospiraceae_NK4A136_group
g__Ruminiclostridium_6
g__Eisenbergiella
g__Odoribacter
g__Lachnospiraceae_ND3007_group
g__Butyricimonas
g__Alistipes
g__Ruminococcus_torques_group
g__Prevotellaceae_UCG−001
g__Clostridium_sensu_stricto_1
g__Prevotellaceae_UCG−003
g__Ruminococcaceae_UCG−003
g__Candidatus_Soleaferrea
g__Paeniclostridium
g__Peptoniphilus
g__Senegalimassilia
g__Catenibacterium
g__Ruminococcaceae_UCG−009
g__Escherichia−Shigella
g__Lachnospiraceae_AC2044_group
g__Marinobacter
g__Prevotella_1
g__dgA−11_gut_group
g__Epulopiscium
g__Aggregatibacter
g__Acholeplasma
g__Hymenobacter
Nor1
Nor2
Nor3
Nor4
Nor5
Nor6
Nor7
Nor8
Nor9
Nor10
Nor11
Nor12
Nor13
Nor14
Nor15
Nor16
Nor17
Nor18
Nor19
Nor20
Nor21
ES1
ES2
ES3
ES4
ES5
ES6
ES7
ES8
ES9
ES10
ES11
ES12
ES13
ES14
ES15
