# Supplementary material for: Characterization of Esophageal Microbiota in Patients With Esophagitis and Esophageal Squamous Cell Carcinoma
Source: Front Cell Infect Microbiol. 2021 Nov 11;11:774330. doi: 10.3389/fcimb.2021.774330 (PMC8632060; doi:10.3389/fcimb.2021.774330)
Supplement: Supplementary file 2 [file DataSheet_1.zip › 16S_V3_V4-68╕÷╤∙▒╛JZD 2020.7.21/5.Statistical_test_analysis/Wilcoxon/OTUs/diff_ES-ESC.txt.pdf]

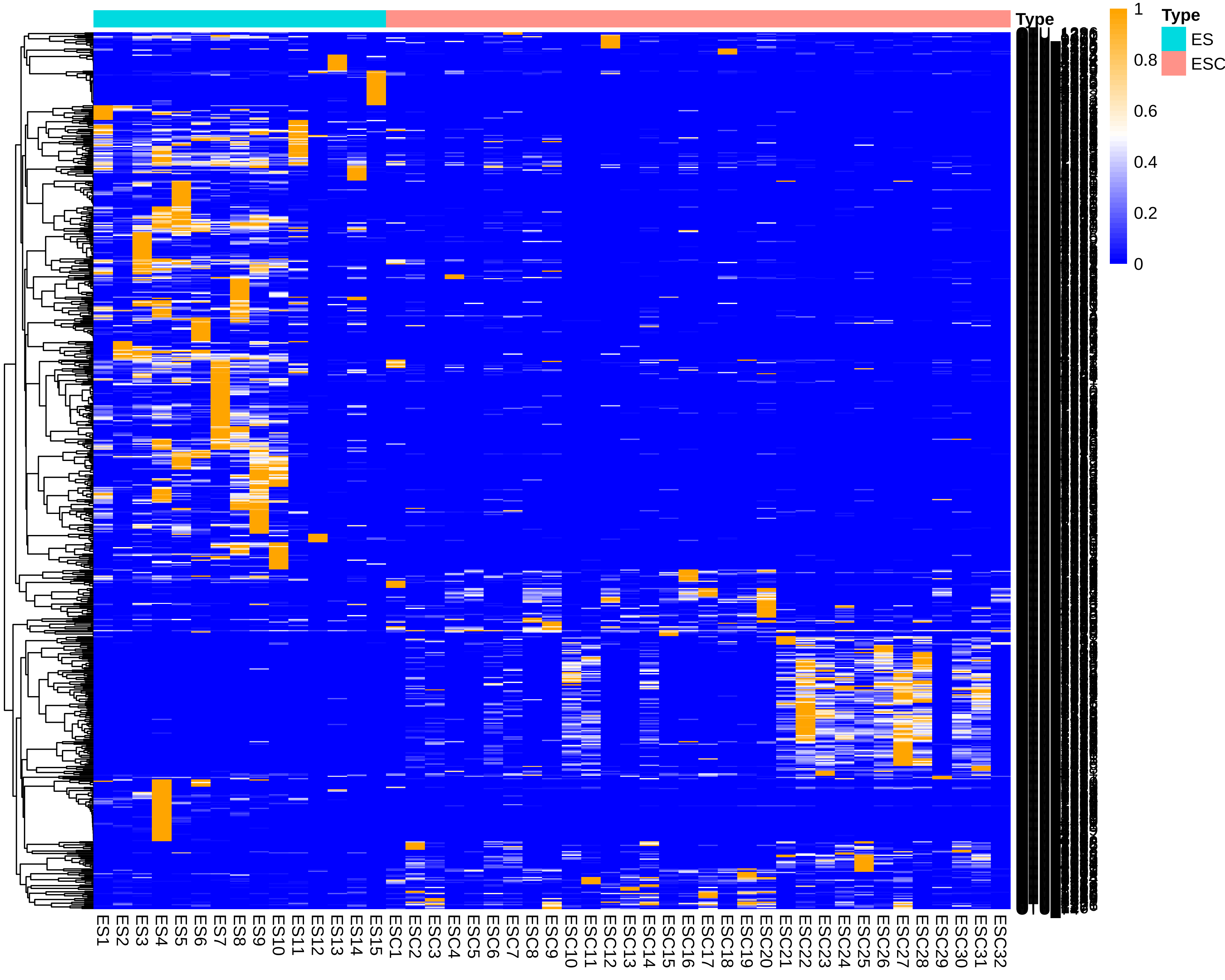

Type
1
0.8
0.6
0.4
0.2
0
Type
ES
ESC
ES1
ES2
ES3
ES4
ES5
ES6
ES7
ES8
ES9
ES10
ES11
ES12
ES13
ES14
ES15
ESC1
ESC2
ESC3
ESC4
ESC5
ESC6
ESC7
ESC8
ESC9
ESC10
ESC11
ESC12
ESC13
ESC14
ESC15
ESC16
ESC17
ESC18
ESC19
ESC20
ESC21
ESC22
ESC23
ESC24
ESC25
ESC26
ESC27
ESC28
ESC29
ESC30
ESC31
ESC32
