# Supplementary material for: Characterization of Esophageal Microbiota in Patients With Esophagitis and Esophageal Squamous Cell Carcinoma
Source: Front Cell Infect Microbiol. 2021 Nov 11;11:774330. doi: 10.3389/fcimb.2021.774330 (PMC8632060; doi:10.3389/fcimb.2021.774330)
Supplement: Supplementary file 2 [file DataSheet_1.zip › 16S_V3_V4-68╕÷╤∙▒╛JZD 2020.7.21/5.Statistical_test_analysis/Wilcoxon/OTUs/diff_Normal-ES.txt.pdf]

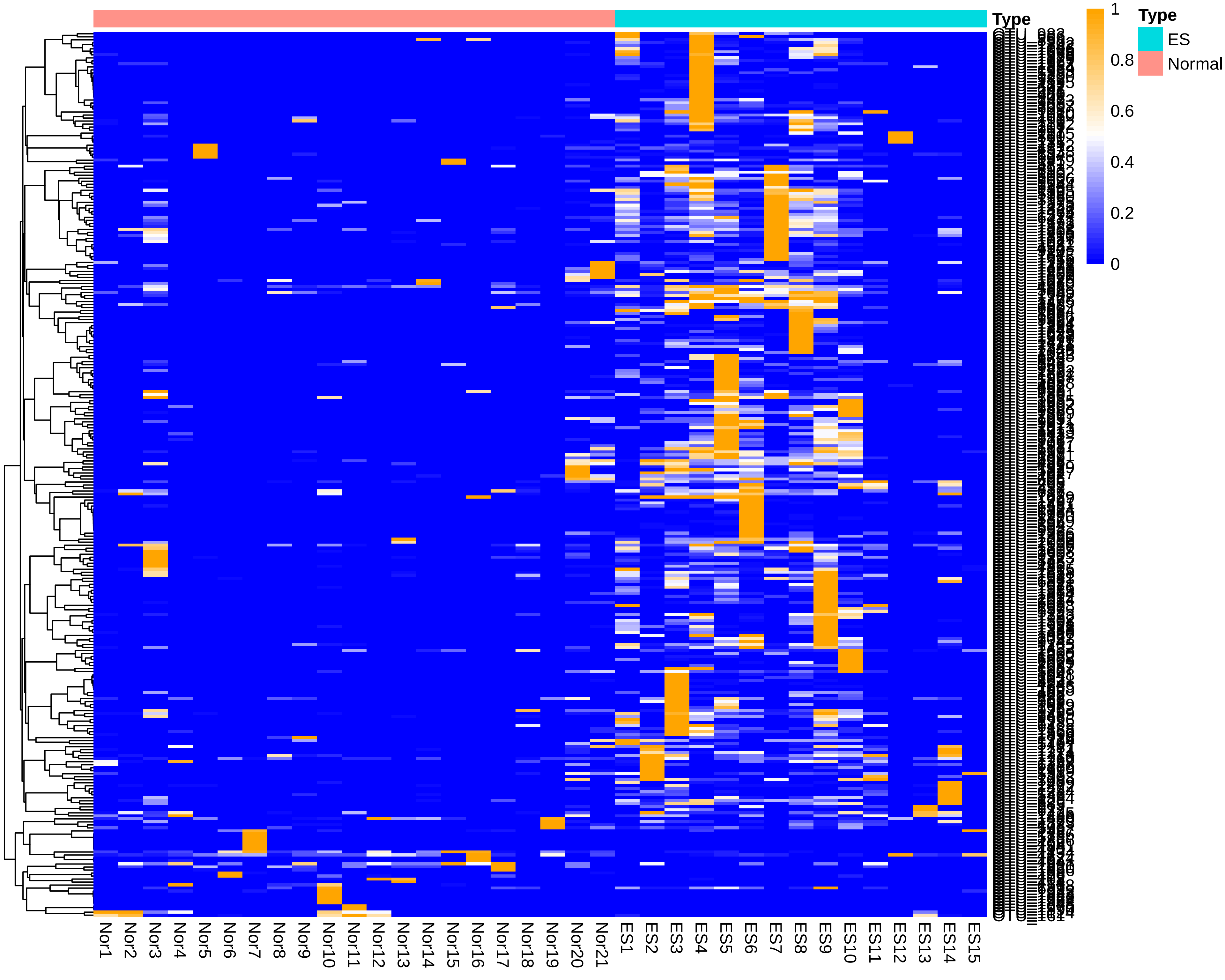
Type
Type
ES
Normal
1
0.8
0.6
0.4
0.2
0
Nor1
Nor2
Nor3
Nor4
Nor5
Nor6
Nor7
Nor8
Nor9
Nor10
Nor11
Nor12
Nor13
Nor14
Nor15
Nor16
Nor17
Nor18
Nor19
Nor20
Nor21
ES1
ES2
ES3
ES4
ES5
ES6
ES7
ES8
ES9
ES10
ES11
ES12
ES13
ES14
ES15
