# Supplementary material for: Characterization of Esophageal Microbiota in Patients With Esophagitis and Esophageal Squamous Cell Carcinoma
Source: Front Cell Infect Microbiol. 2021 Nov 11;11:774330. doi: 10.3389/fcimb.2021.774330 (PMC8632060; doi:10.3389/fcimb.2021.774330)
Supplement: Supplementary file 2 [file DataSheet_1.zip › 16S_V3_V4-68╕÷╤∙▒╛JZD 2020.7.21/5.Statistical_test_analysis/Wilcoxon/Order/diff_ES-ESC.txt.pdf]

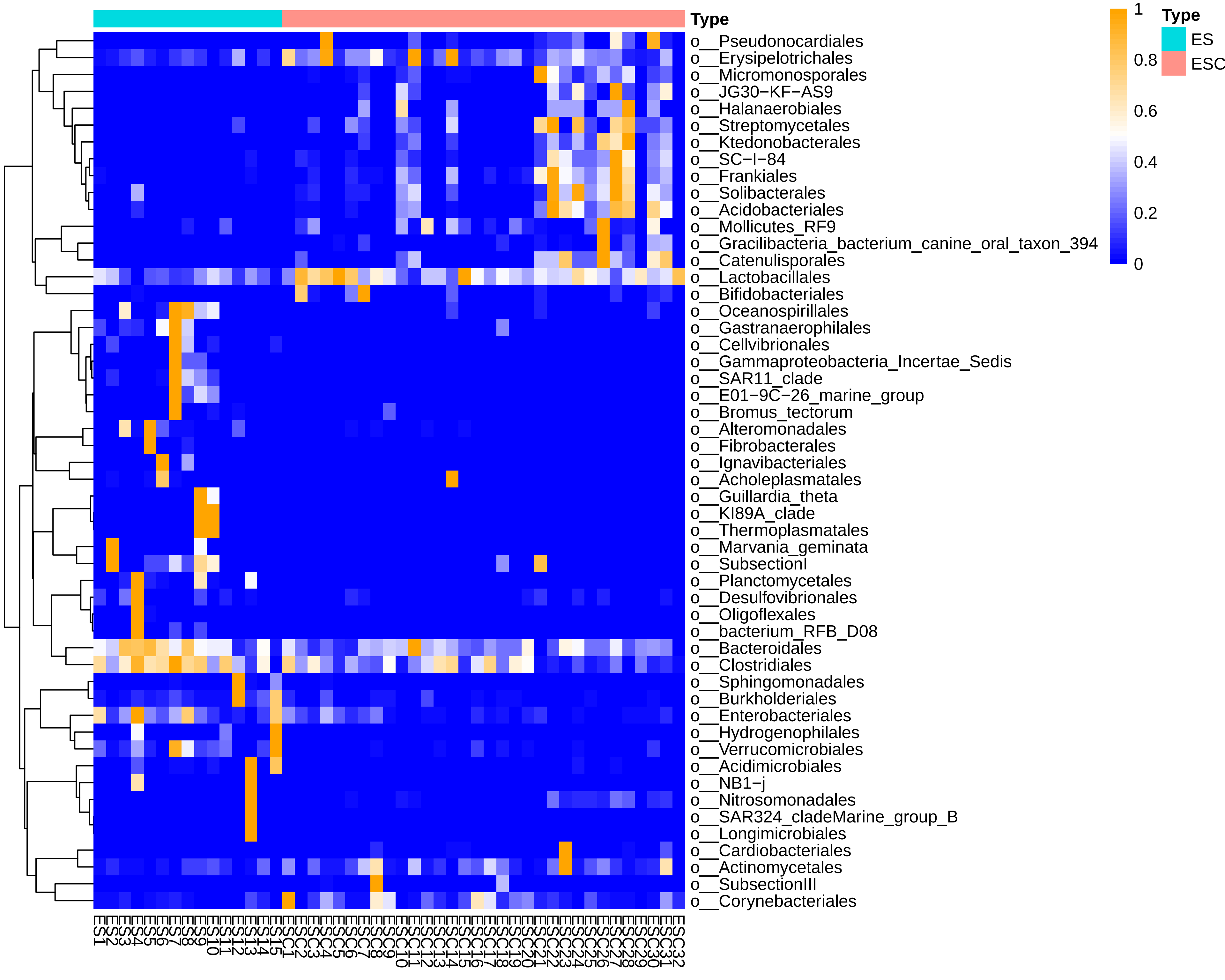
Type
1
0.8
0.6
0.4
0.2
0
Type
ES
ESC
o__Pseudonocardiales
o__Erysipelotrichales
o__Micromonosporales
o__JG30−KF−AS9
o__Halanaerobiales
o__Streptomycetales
o__Ktedonobacterales
o__SC−I−84
o__Frankiales
o__Solibacterales
o__Acidobacteriales
o__Mollicutes_RF9
o__Gracilibacteria_bacterium_canine_oral_taxon_394
o__Catenulisporales
o__Lactobacillales
o__Bifidobacteriales
o__Oceanospirillales
o__Gastranaerophilales
o__Cellvibrionales
o__Gammaproteobacteria_Incertae_Sedis
o__SAR11_clade
o__E01−9C−26_marine_group
o__Bromus_tectorum
o__Alteromonadales
o__Fibrobacterales
o__Ignavibacteriales
o__Acholeplasmatales
o__Guillardia_theta
o__KI89A_clade
o__Thermoplasmatales
o__Marvania_geminata
o__SubsectionI
o__Planctomycetales
o__Desulfovibrionales
o__Oligoflexales
o__bacterium_RFB_D08
o__Bacteroidales
o__Clostridiales
o__Sphingomonadales
o__Burkholderiales
o__Enterobacteriales
o__Hydrogenophilales
o__Verrucomicrobiales
o__Acidimicrobiales
o__NB1−j
o__Nitrosomonadales
o__SAR324_cladeMarine_group_B
o__Longimicrobiales
o__Cardiobacteriales
o__Actinomycetales
o__SubsectionIII
o__Corynebacteriales
ES1
ES2
ES3
ES4
ES5
ES6
ES7
ES8
ES9
ES10
ES11
ES12
ES13
ES14
ES15
ESC1
ESC2
ESC3
ESC4
ESC5
ESC6
ESC7
ESC8
ESC9
ESC10
ESC11
ESC12
ESC13
ESC14
ESC15
ESC16
ESC17
ESC18
ESC19
ESC20
ESC21
ESC22
ESC23
ESC24
ESC25
ESC26
ESC27
ESC28
ESC29
ESC30
ESC31
ESC32
