# Supplementary material for: Characterization of Esophageal Microbiota in Patients With Esophagitis and Esophageal Squamous Cell Carcinoma
Source: Front Cell Infect Microbiol. 2021 Nov 11;11:774330. doi: 10.3389/fcimb.2021.774330 (PMC8632060; doi:10.3389/fcimb.2021.774330)
Supplement: Supplementary file 2 [file DataSheet_1.zip › 16S_V3_V4-68╕÷╤∙▒╛JZD 2020.7.21/5.Statistical_test_analysis/Wilcoxon/Order/diff_Normal-ES.txt.pdf]

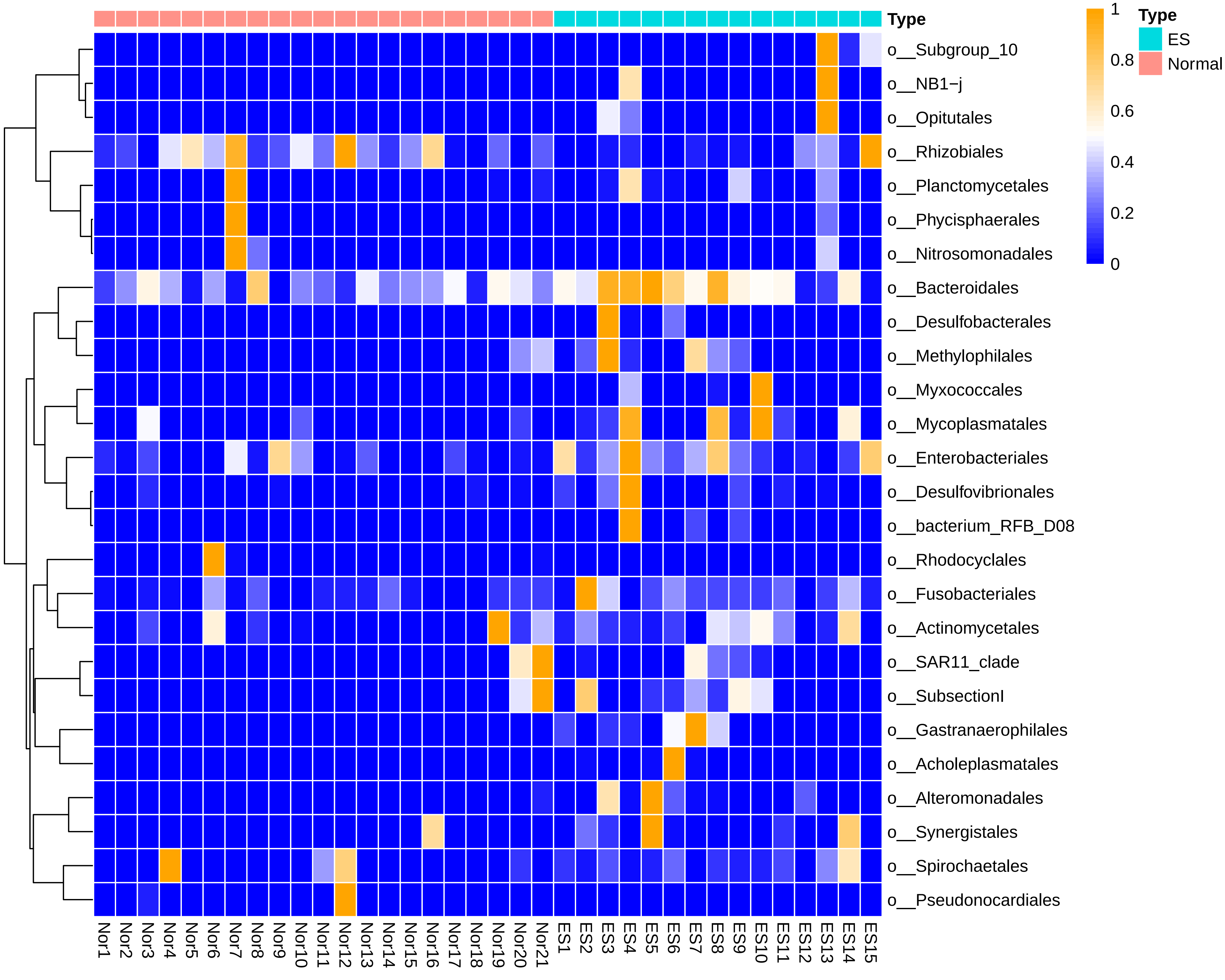
Type
o__Subgroup_10
o__NB1−j
o__Opitutales
o__Rhizobiales
o__Planctomycetales
o__Phycisphaerales
o__Nitrosomonadales
o__Bacteroidales
o__Desulfobacterales
o__Methylophilales
o__Myxococcales
o__Mycoplasmatales
o__Enterobacteriales
o__Desulfovibrionales
o__bacterium_RFB_D08
o__Rhodocyclales
o__Fusobacteriales
o__Actinomycetales
o__SAR11_clade
o__SubsectionI
o__Gastranaerophilales
o__Acholeplasmatales
o__Alteromonadales
o__Synergistales
o__Spirochaetales
o__Pseudonocardiales
Nor1 Nor2 Nor3 Nor4 Nor5 Nor6 Nor7 Nor8 Nor9 Nor10 Nor11 Nor12 Nor13 Nor14 Nor15 Nor16 Nor17 Nor18 Nor19 Nor20 Nor21
ES1 ES2 ES3 ES4 ES5 ES6 ES7 ES8 ES9 ES10 ES11 ES12 ES13 ES14 ES15
1
0.8
0.6
0.4
0.2
0
Type
ES
Normal
