# Supplementary material for: Characterization of Esophageal Microbiota in Patients With Esophagitis and Esophageal Squamous Cell Carcinoma
Source: Front Cell Infect Microbiol. 2021 Nov 11;11:774330. doi: 10.3389/fcimb.2021.774330 (PMC8632060; doi:10.3389/fcimb.2021.774330)
Supplement: Supplementary file 2 [file DataSheet_1.zip › 16S_V3_V4-68╕÷╤∙▒╛JZD 2020.7.21/5.Statistical_test_analysis/Wilcoxon/Order/diff_Normal-ESC.txt.pdf]

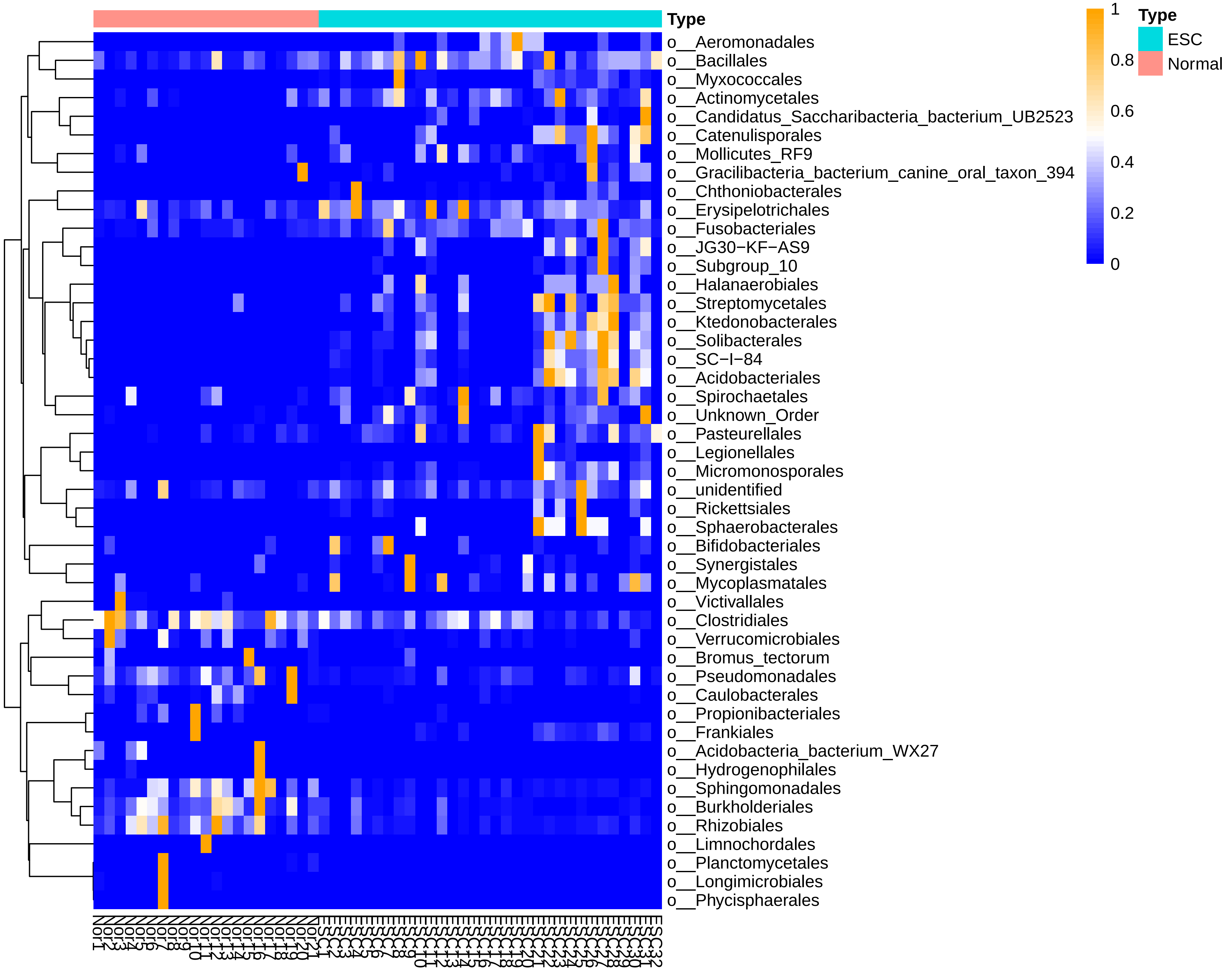
Type
ESC
Normal
1
0.8
0.6
0.4
0.2
0
o__Aeromonadales
o__Bacillales
o__Myxococcales
o__Actinomycetales
o__Candidatus_Saccharibacteria_bacterium_UB2523
o__Catenulisporales
o__Mollicutes_RF9
o__Gracilibacteria_bacterium_canine_oral_taxon_394
o__Chthoniobacterales
o__Erysipelotrichales
o__Fusobacteriales
o__JG30−KF−AS9
o__Subgroup_10
o__Halanaerobiales
o__Streptomycetales
o__Ktedonobacterales
o__Solibacterales
o__SC−I−84
o__Acidobacteriales
o__Spirochaetales
o__Unknown_Order
o__Pasteurellales
o__Legionellales
o__Micromonosporales
o__unidentified
o__Rickettsiales
o__Sphaerobacterales
o__Bifidobacteriales
o__Synergistales
o__Mycoplasmatales
o__Victivallales
o__Clostridiales
o__Verrucomicrobiales
o__Bromus_tectorum
o__Pseudomonadales
o__Caulobacterales
o__Propionibacteriales
o__Frankiales
o__Acidobacteria_bacterium_WX27
o__Hydrogenophilales
o__Sphingomonadales
o__Burkholderiales
o__Rhizobiales
o__Limnochordales
o__Planctomycetales
o__Longimicrobiales
o__Phycisphaerales
