# Supplementary material for: Characterization of Esophageal Microbiota in Patients With Esophagitis and Esophageal Squamous Cell Carcinoma
Source: Front Cell Infect Microbiol. 2021 Nov 11;11:774330. doi: 10.3389/fcimb.2021.774330 (PMC8632060; doi:10.3389/fcimb.2021.774330)
Supplement: Supplementary file 2 [file DataSheet_1.zip › 16S_V3_V4-68╕÷╤∙▒╛JZD 2020.7.21/5.Statistical_test_analysis/Wilcoxon/Order/errbar_ES-ESC_errbar.pdf]

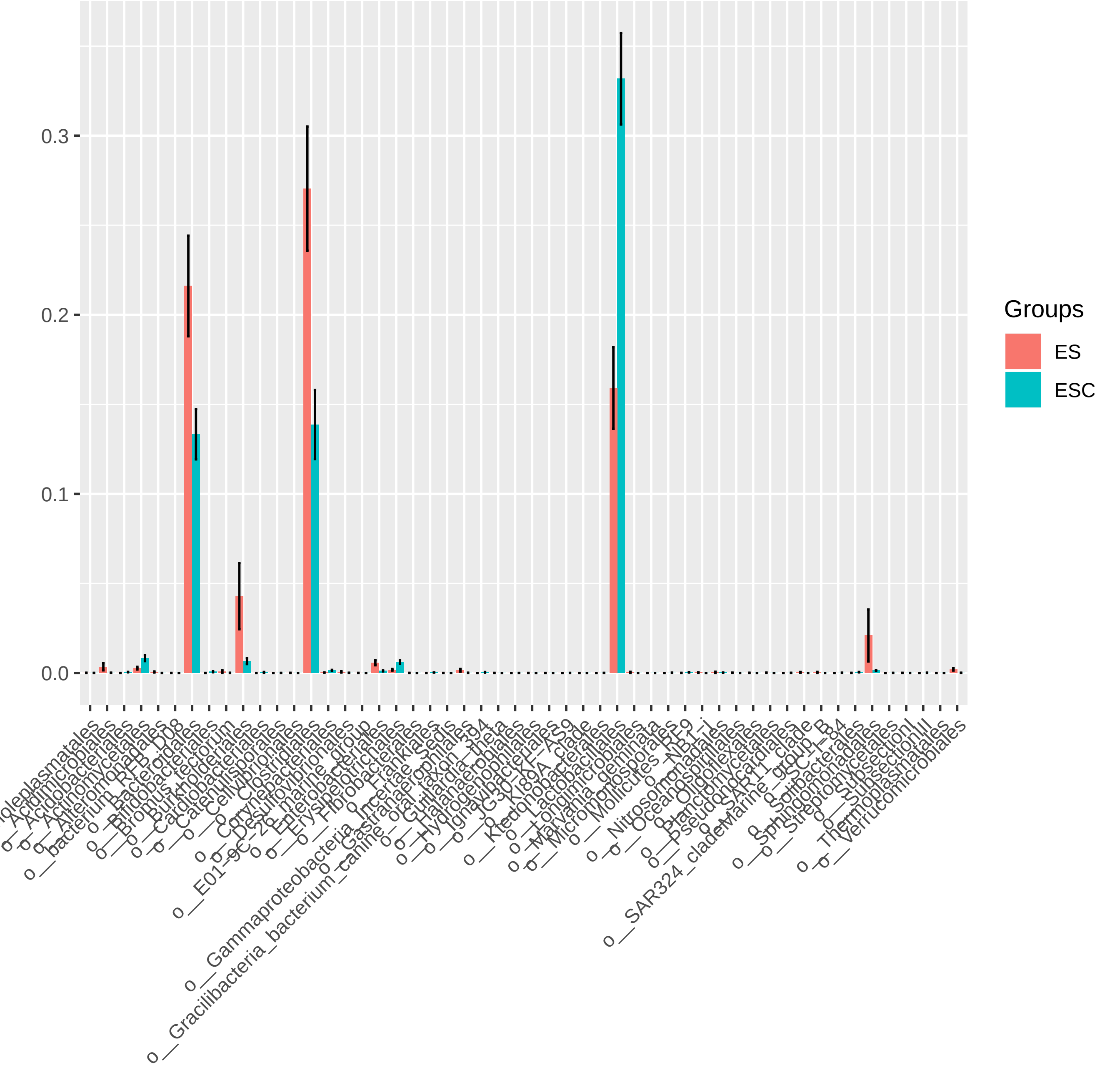

0.3
0.2
0.1
0.0
Groups
ES
ESC
o__Acidimicrobiales
o__Acidobacteriales
o__Actinomycetales
o__Alteromonadales
o__Bacteroidales
o__Bifidobacteriales
o__Burkholderiales
o__Clostridiales
o__Corynebacteriales
o__Desulfovibrionales
o__E01-9C-26_marine_group
o__Enterobacteriales
o__Erysipelotrichales
o__Fibrobacterales
o__Frankiales
o__Gammaproteobacteria_Incertae_Sedis
o__Gracilibacteria_bacterium_canine_oral_taxon_394
o__Lactobacillales
o__Nitrosomonadales
o__SAR324_clade(Marine_group_B)
o__Sphingomonadales
o__Streptomycetales
o__Thermoplasmatales
o__Verrucomicrobiales
