# Supplementary material for: Characterization of Esophageal Microbiota in Patients With Esophagitis and Esophageal Squamous Cell Carcinoma
Source: Front Cell Infect Microbiol. 2021 Nov 11;11:774330. doi: 10.3389/fcimb.2021.774330 (PMC8632060; doi:10.3389/fcimb.2021.774330)
Supplement: Supplementary file 2 [file DataSheet_1.zip › 16S_V3_V4-68╕÷╤∙▒╛JZD 2020.7.21/5.Statistical_test_analysis/Wilcoxon/Order/errbar_Normal-ESC_errbar.pdf]

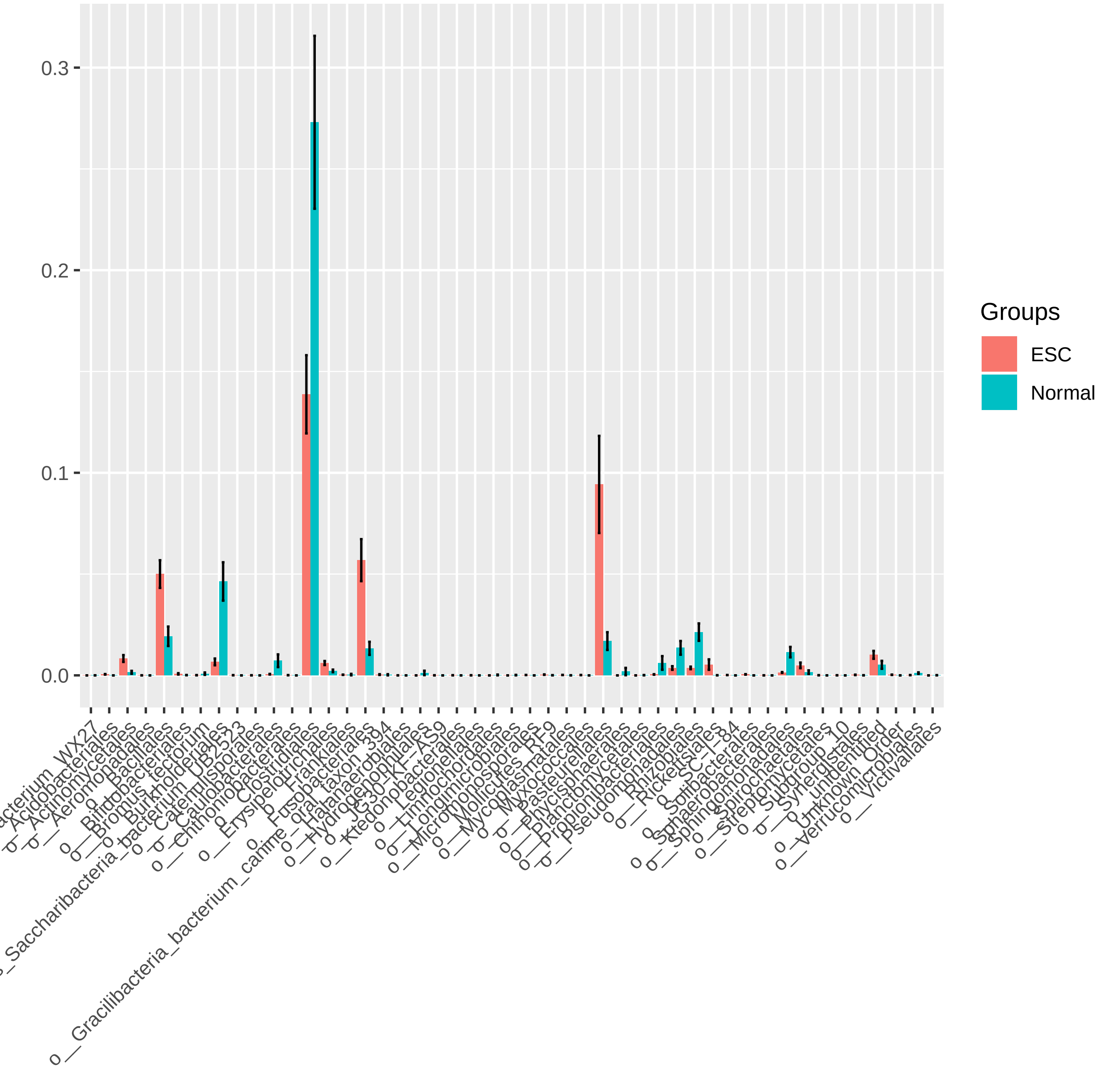
0.3
0.2
0.1
0.0
Groups
ESC
Normal
o__Acidobacteriales
o__Actinomycetales
o__Aeromonadales
o__Bacillales
o__Bifidobacteriales
o__Bromus_tectorum
o__Burkholderiales
o__Caternulisporales
o__Caulobacterales
o__Chthoniobacterales
o__Clostridiales
o__Erysipelotrichales
o__Frankiales
o__Fusobacteriales
o__Gracilibacteria_bacterium_canine_oral_taxon_394
o__Halanaerobiales
o__Hydrogenophilales
o__JG30-KF-AS9
o__Ktedonobacterales
o__Legionellales
o__Limnochordales
o__Longimicrobiales
o__Micromonosporales
o__Mollicutes_RF9
o__Mycoplasmatales
o__Myxococcales
o__Pasteurellales
o__Phycisphaerales
o__Planctomycetales
o__Propionibacteriales
o__Pseudomonadales
o__Rhizobiales
o__Rickettsiales
o__SC-I-84
o__Solibacterales
o__Sphaerobacterales
o__Sphingomonadales
o__Spirochaetales
o__Streptomycetales
o__Subgroup_10
o__Synergistales
o__unidentified
o__Unknown_Order
o__Verrucomicrobiales
o__Victivallales
