# Supplementary material for: Characterization of Esophageal Microbiota in Patients With Esophagitis and Esophageal Squamous Cell Carcinoma
Source: Front Cell Infect Microbiol. 2021 Nov 11;11:774330. doi: 10.3389/fcimb.2021.774330 (PMC8632060; doi:10.3389/fcimb.2021.774330)
Supplement: Supplementary file 2 [file DataSheet_1.zip › 16S_V3_V4-68╕÷╤∙▒╛JZD 2020.7.21/5.Statistical_test_analysis/Wilcoxon/Order/errbar_Normal-ES_errbar.pdf]

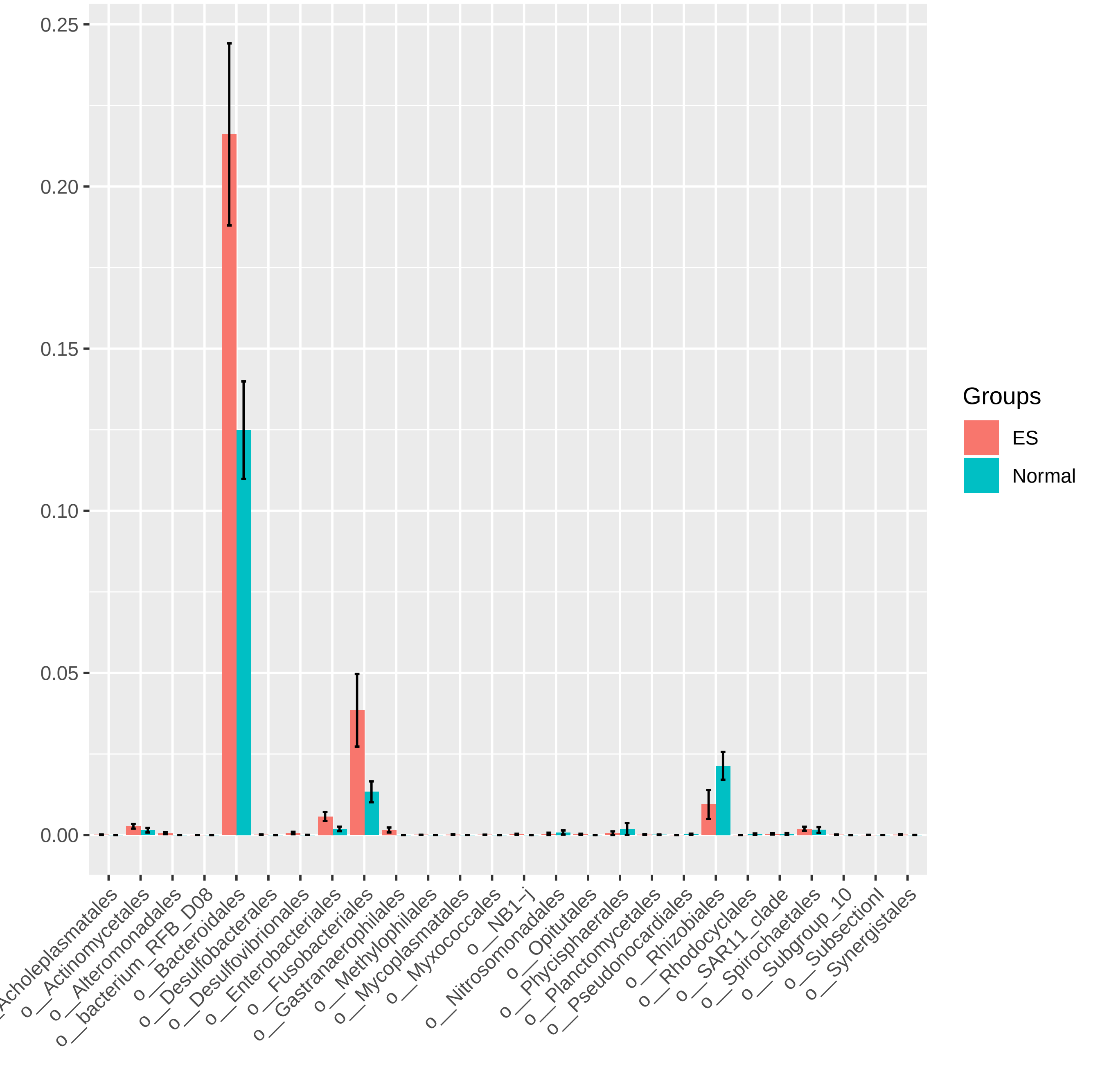

0.25
0.20
0.15
0.10
0.05
0.00
Groups
ES
Normal
o__Acholeplasmatales
o__Actinomycetales
o__Alteromonadales
o__bacterium_RFB_D08
o__Bacteroidales
o__Desulfobacterales
o__Desulfovibrionales
o__Enterobacteriales
o__Fusobacteriales
o__Gastranaerophilales
o__Methylophilales
o__Mycoplasmatales
o__Myxococcales
o__NB1−j
o__Nitrosomonadales
o__Opitutales
o__Phycisphaerales
o__Planctomycetales
o__Pseudonocardiales
o__Rhizobiales
o__Rhodocyclales
o__SAR11_clade
o__Spirochaetales
o__Subgroup_10
o__SubsectionI
o__Synergistales
