# Supplementary material for: Characterization of Esophageal Microbiota in Patients With Esophagitis and Esophageal Squamous Cell Carcinoma
Source: Front Cell Infect Microbiol. 2021 Nov 11;11:774330. doi: 10.3389/fcimb.2021.774330 (PMC8632060; doi:10.3389/fcimb.2021.774330)
Supplement: Supplementary file 2 [file DataSheet_1.zip › 16S_V3_V4-68╕÷╤∙▒╛JZD 2020.7.21/5.Statistical_test_analysis/Wilcoxon/Phylum/diff_ES-ESC.txt.pdf]

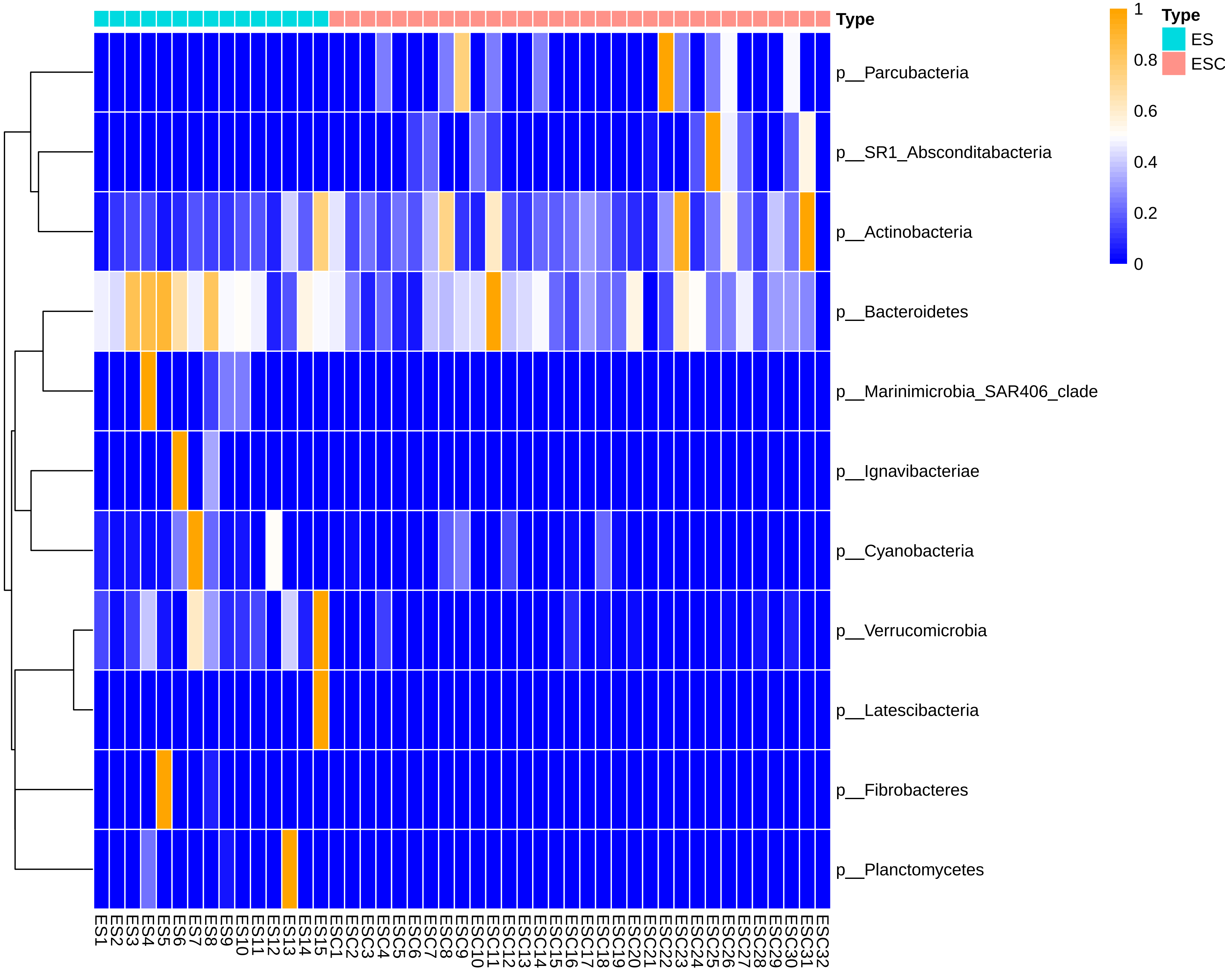

Type
ES
ESC
1
0.8
0.6
0.4
0.2
0
p__Parcubacteria
p__SR1_Absconditabacteria
p__Actinobacteria
p__Bacteroidetes
p__Marinimicrobia_SAR406_clade
p__Ignavibacteriae
p__Cyanobacteria
p__Verrucomicrobia
p__Latescibacteria
p__Fibrobacteres
p__Planctomycetes
ES1 ES2 ES3 ES4 ES5 ES6 ES7 ES8 ES9 ES10 ES11 ES12 ES13 ES14 ES15
ESC1 ESC2 ESC3 ESC4 ESC5 ESC6 ESC7 ESC8 ESC9 ESC10 ESC11 ESC12 ESC13 ESC14 ESC15 ESC16 ESC17 ESC18 ESC19 ESC20 ESC21 ESC22 ESC23 ESC24 ESC25 ESC26 ESC27 ESC28 ESC29 ESC30 ESC31 ESC32
