# Supplementary material for: Characterization of Esophageal Microbiota in Patients With Esophagitis and Esophageal Squamous Cell Carcinoma
Source: Front Cell Infect Microbiol. 2021 Nov 11;11:774330. doi: 10.3389/fcimb.2021.774330 (PMC8632060; doi:10.3389/fcimb.2021.774330)
Supplement: Supplementary file 2 [file DataSheet_1.zip › 16S_V3_V4-68╕÷╤∙▒╛JZD 2020.7.21/5.Statistical_test_analysis/Wilcoxon/Phylum/diff_Normal-ESC.txt.pdf]

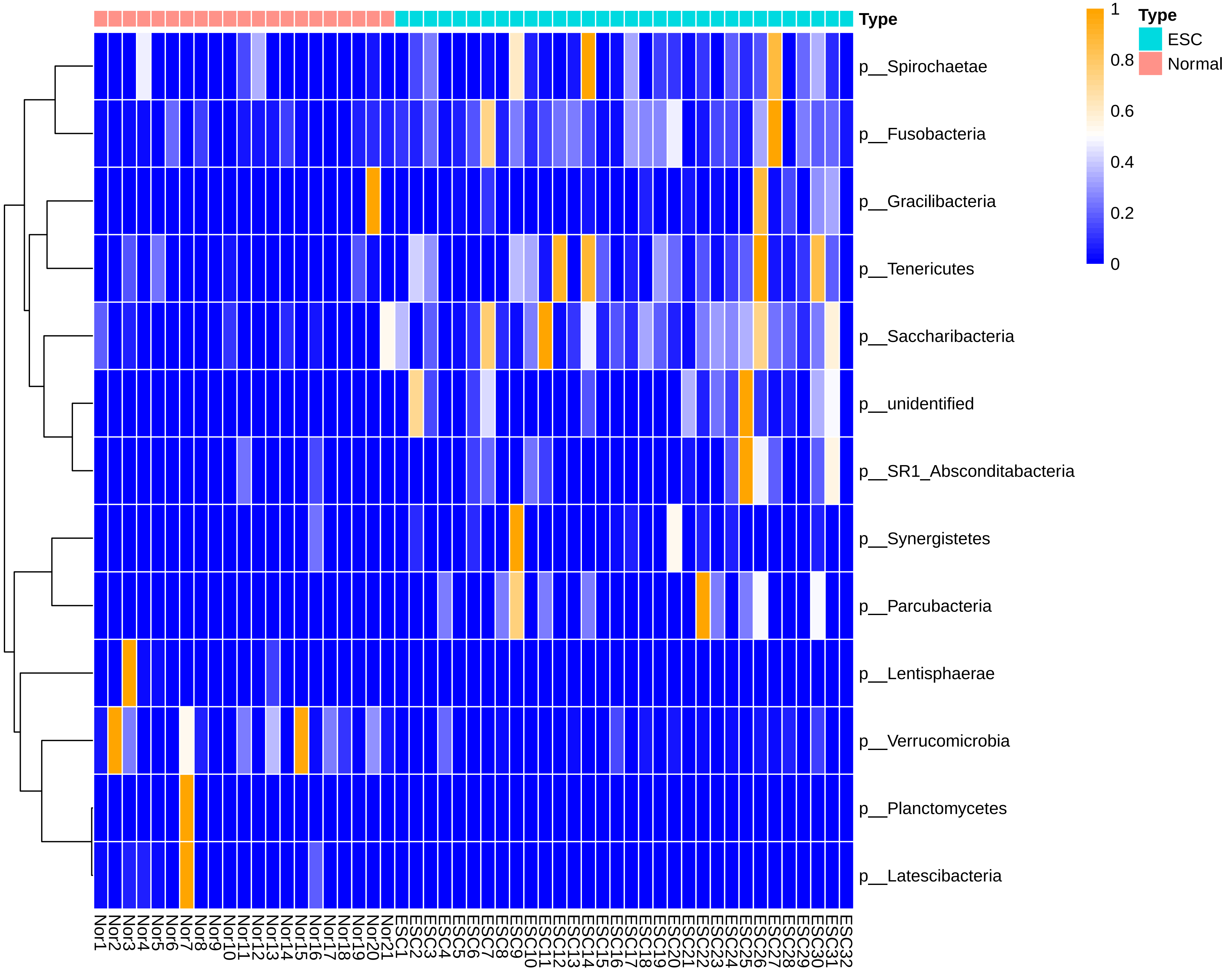

Type
ESC
Normal
1
0.8
0.6
0.4
0.2
0
p__Spirochaetae
p__Fusobacteria
p__Gracilibacteria
p__Tenericutes
p__Saccharibacteria
p__unidentified
p__SR1_Absconditabacteria
p__Synergistetes
p__Parcubacteria
p__Lentisphaerae
p__Verrucomicrobia
p__Planctomycetes
p__Latescibacteria
Nor1
Nor2
Nor3
Nor4
Nor5
Nor6
Nor7
Nor8
Nor9
Nor10
Nor11
Nor12
Nor13
Nor14
Nor15
Nor16
Nor17
Nor18
Nor19
Nor20
Nor21
ESC1
ESC2
ESC3
ESC4
ESC5
ESC6
ESC7
ESC8
ESC9
ESC10
ESC11
ESC12
ESC13
ESC14
ESC15
ESC16
ESC17
ESC18
ESC19
ESC20
ESC21
ESC22
ESC23
ESC24
ESC25
ESC26
ESC27
ESC28
ESC29
ESC30
ESC31
ESC32
