# Supplementary material for: Characterization of Esophageal Microbiota in Patients With Esophagitis and Esophageal Squamous Cell Carcinoma
Source: Front Cell Infect Microbiol. 2021 Nov 11;11:774330. doi: 10.3389/fcimb.2021.774330 (PMC8632060; doi:10.3389/fcimb.2021.774330)
Supplement: Supplementary file 2 [file DataSheet_1.zip › 16S_V3_V4-68╕÷╤∙▒╛JZD 2020.7.21/5.Statistical_test_analysis/Wilcoxon/Phylum/errbar_Normal-ESC_errbar.pdf]

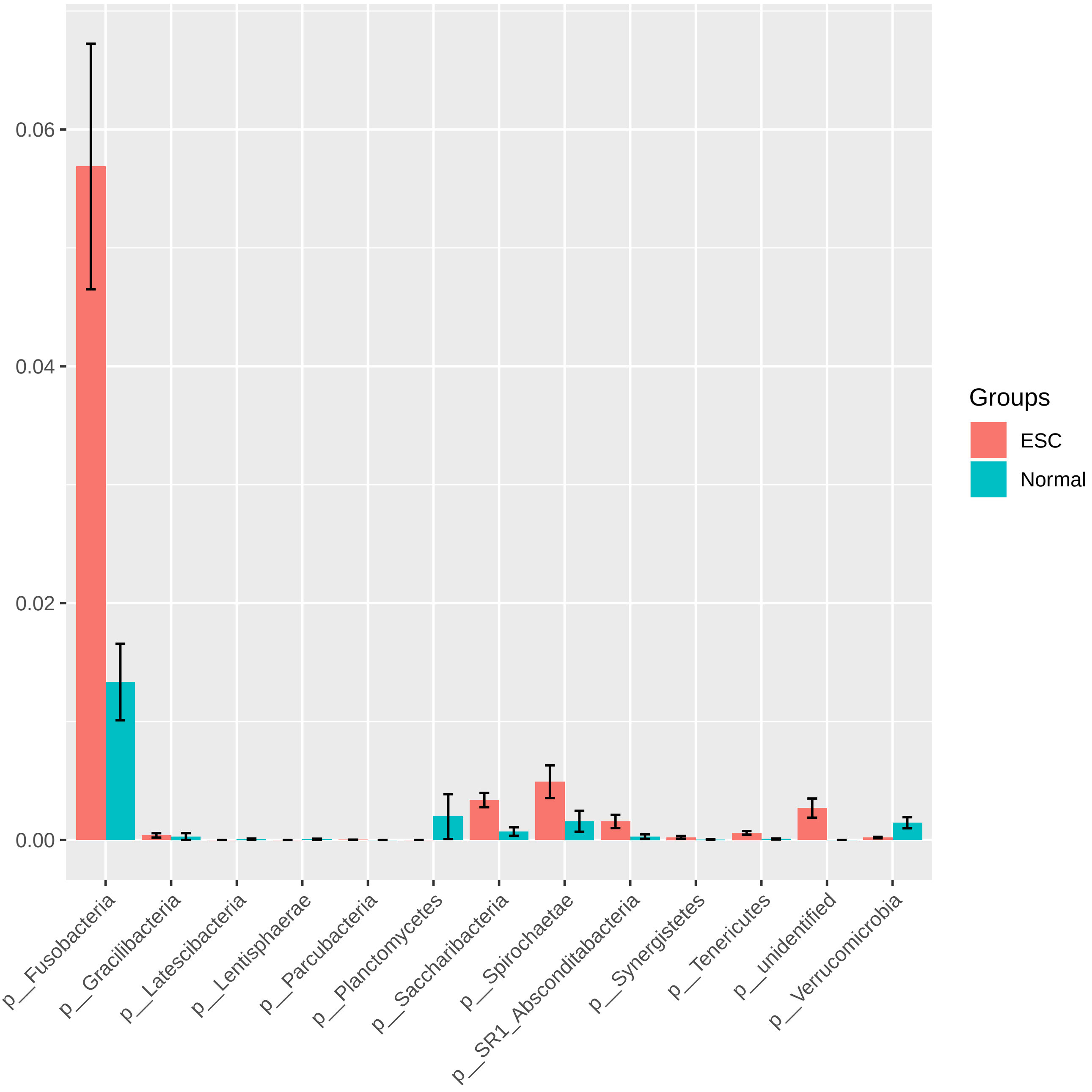

0.06
0.04
0.02
0.00
Groups
ESC
Normal
p__Fusobacteria
p__Gracilibacteria
p__Latescibacteria
p__Lentisphaerae
p__Parcubacteria
p__Planctomycetes
p__Saccharibacteria
p__Spirochaetae
p__SR1_Absconditabacteria
p__Synergistetes
p__Tenericutes
p__unidentified
p__Verrucomicrobia
