# Supplementary material for: Characterization of Esophageal Microbiota in Patients With Esophagitis and Esophageal Squamous Cell Carcinoma
Source: Front Cell Infect Microbiol. 2021 Nov 11;11:774330. doi: 10.3389/fcimb.2021.774330 (PMC8632060; doi:10.3389/fcimb.2021.774330)
Supplement: Supplementary file 2 [file DataSheet_1.zip › 16S_V3_V4-68╕÷╤∙▒╛JZD 2020.7.21/5.Statistical_test_analysis/Wilcoxon/Phylum/errbar_Normal-ES_errbar.pdf]

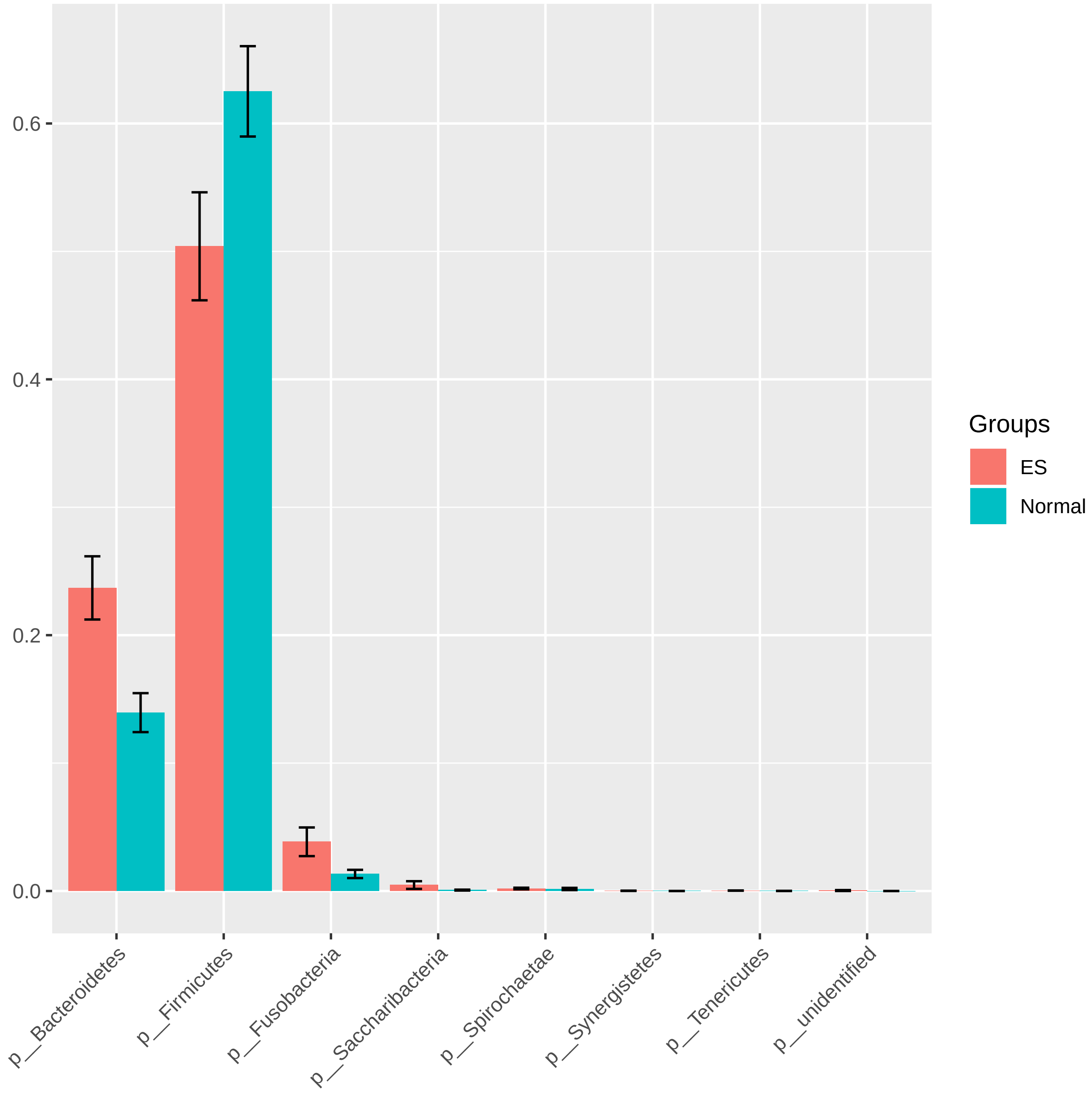
0.6
0.4
0.2
0.0
p__Bacteroidetes
p__Firmicutes
p__Fusobacteria
p__Saccharibacteria
p__Spirochaetae
p__Synergistetes
p__Tenericutes
p__unidentified
Groups
ES
Normal
