# Supplementary material for: Characterization of Esophageal Microbiota in Patients With Esophagitis and Esophageal Squamous Cell Carcinoma
Source: Front Cell Infect Microbiol. 2021 Nov 11;11:774330. doi: 10.3389/fcimb.2021.774330 (PMC8632060; doi:10.3389/fcimb.2021.774330)
Supplement: Supplementary file 2 [file DataSheet_1.zip › 16S_V3_V4-68╕÷╤∙▒╛JZD 2020.7.21/6.Correlation_analysis/Network/p_network.pdf]

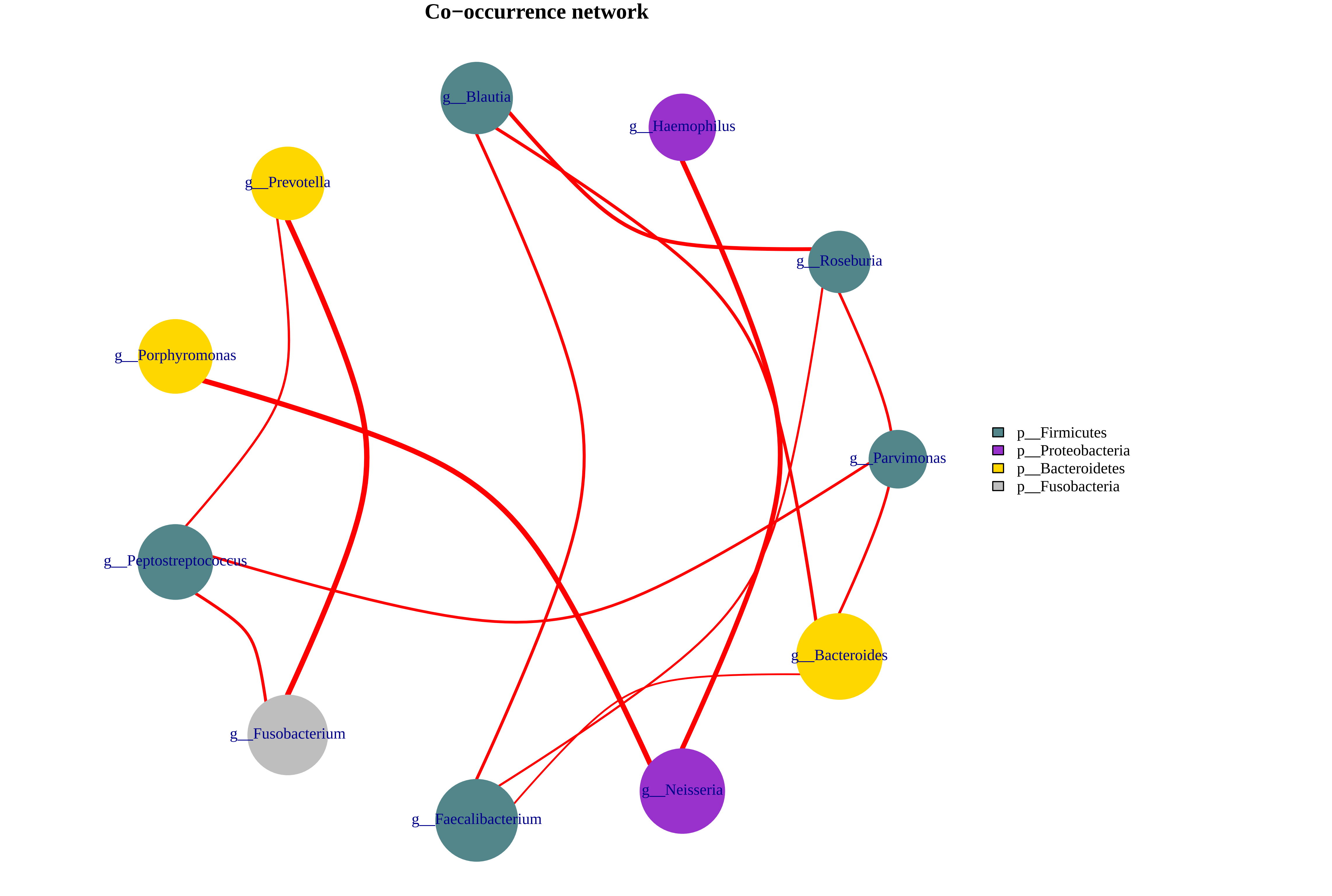
Co−occurrence network
g__Blautia
g__Haemophilus
g__Prevotella
g__Roseburia
g__Porphyromonas
g__Parvimonas
g__Peptostreptococcus
g__Bacteroides
g__Fusobacterium
g__Neisseria
g__Faecalibacterium
p__Firmicutes
p__Proteobacteria
p__Bacteroidetes
p__Fusobacteria
