# Supplementary material for: Characterization of Esophageal Microbiota in Patients With Esophagitis and Esophageal Squamous Cell Carcinoma
Source: Front Cell Infect Microbiol. 2021 Nov 11;11:774330. doi: 10.3389/fcimb.2021.774330 (PMC8632060; doi:10.3389/fcimb.2021.774330)
Supplement: Supplementary file 2 [file DataSheet_1.zip › 16S_V3_V4-68╕÷╤∙▒╛JZD 2020.7.21/Report/mic_report.html]

微生物多样性信息分析结题报告


项目基本信息

实验流程

数据质控及OTU基础分析

Alpha多样性分析

物种注释

Beta多样性分析

多元统计分析

相关性分析

附录

# 项目基本信息/Basic project information

- all
- 项目摘要
- 分析流程

## 1. 项目摘要

项目编号&项目名：20200717-微生物测序

项目类型：16S\_V3-V4

## 2. 分析流程

为使分析结果更加准确、可靠，测序原始数据下机后，首先进行数据质控，通过序列拼接、过滤和去嵌合体后得到优化序列，然后进行OTU聚类及注释。基于聚类结果，可以进行Alpha多样性分析和Beta多样性分析；基于注释结果，可以得到各水平的分类信息，从而进行样本组成及样本间群落结构差异相关分析。整个分析流程参照下图：

图1 微生物多样性信息分析流程示意图

## 1. 项目摘要

项目编号&项目名：20200717-微生物测序

项目类型：16S\_V3-V4

## 2. 分析流程

为使分析结果更加准确、可靠，测序原始数据下机后，首先进行数据质控，通过序列拼接、过滤和去嵌合体后得到优化序列，然后进行OTU聚类及注释。基于聚类结果，可以进行Alpha多样性分析和Beta多样性分析；基于注释结果，可以得到各水平的分类信息，从而进行样本组成及样本间群落结构差异相关分析。整个分析流程参照下图：

图1 微生物多样性信息分析流程示意图

# 实验流程/Experimental procedure

- all
- 基因DNA的提取
- PCR扩增
- Miseq文库构建
- Miseq上机测序

## 实验过程如下图所示：

图2 微生物实验流程示意图

## 1. 基因DNA的提取

完成基因组DNA抽提后，利用1%琼脂糖凝胶电泳检测抽提的基因组DNA。

## 2. PCR扩增

按指定测序区域，合成带有barcode的特异引物，或合成带有错位碱基的融合引物。

为保证后续数据分析的准确性及可靠性，需满足两个条件:

1) 尽可能使用低循环数扩增；2) 保证每个样本扩增的循环数一致。随机选取具有代表性的样本进行预实验，确保在最低循环数中使绝大多数样本能够扩增出浓度合适的产物。

PCR 产物使用 1%琼脂糖凝胶电泳检测扩增目的条带大小，并用Agencourt AMPure XP 核酸纯化试剂盒纯化

## 3. Miseq文库构建

1)连接“Y”字形接头；

2)使用磁珠筛选去除接头自连片段；

3)利用PCR扩增进行文库模板的富集；

4)氢氧化钠变性，产生单链DNA片段。

## 4. Miseq上机测序

1)DNA片段的一端与引物碱基互补，固定在芯片上；

2)另一端随机与附近的另外一个引物互补，也被固定住，形成“桥 (bridge)”；

3)PCR扩增，产生DNA簇；

4)DNA扩增子线性化成为单链；

5)加入改造过的DNA聚合酶和带有4种荧光标记的dNTP，每次循环只合成一个碱基；

6)用激光扫描反应板表面，读取每条模板序列第一轮反应所聚合上去的核苷酸种类；

7)将“荧光基团”和“终止基团”化学切割，恢复3'端粘性，继续聚合第二个核苷酸；

8)统计每轮收集到的荧光信号结果，获知模板DNA片段的序列。

## 1. 基因DNA的提取

完成基因组DNA抽提后，利用1%琼脂糖凝胶电泳检测抽提的基因组DNA。

## 2. PCR扩增

按指定测序区域，合成带有barcode的特异引物，或合成带有错位碱基的融合引物。

为保证后续数据分析的准确性及可靠性，需满足两个条件:

1) 尽可能使用低循环数扩增；2) 保证每个样本扩增的循环数一致。随机选取具有代表性的样本进行预实验，确保在最低循环数中使绝大多数样本能够扩增出浓度合适的产物。

PCR 产物使用 1%琼脂糖凝胶电泳检测扩增目的条带大小，并用Agencourt AMPure XP 核酸纯化试剂盒纯化

## 3. Miseq文库构建

1)连接“Y”字形接头；

2)使用磁珠筛选去除接头自连片段；

3)利用PCR扩增进行文库模板的富集；

4)氢氧化钠变性，产生单链DNA片段。

## 4. Miseq上机测序

1)DNA片段的一端与引物碱基互补，固定在芯片上；

2)另一端随机与附近的另外一个引物互补，也被固定住，形成“桥 (bridge)”；

3)PCR扩增，产生DNA簇；

4)DNA扩增子线性化成为单链；

5)加入改造过的DNA聚合酶和带有4种荧光标记的dNTP，每次循环只合成一个碱基；

6)用激光扫描反应板表面，读取每条模板序列第一轮反应所聚合上去的核苷酸种类；

7)将“荧光基团”和“终止基团”化学切割，恢复3'端粘性，继续聚合第二个核苷酸；

8)统计每轮收集到的荧光信号结果，获知模板DNA片段的序列。

# 数据质控及OTU基础分析/QC/Basic analysis

- all
- 测序结果格式介绍
- 数据处理过程和参数
- 有效数据统计
- 优质序列长度分布
- OTU聚类分析
- OTU分布Venn图
- OTU分布花瓣图

## 1. 测序结果格式介绍

原始数据下机后，结果以Fastq格式存储。Fastq文件包含每条测序序列的名称、碱基序列以及其对应的测序质量信息。每条read包含4行信息，其中第一行和第三行由文件识别标志和读段名（ID）组成（第一行以“@”开头，第三行以“+” 开头；第三行中 ID可以省略但“+”不能省略），第二行为碱基序列，而第四行是第二行中的序列内容每个碱基所对应的测序质量值。为方便保存和共享各实验室产生的高通量测序数据，NCBI数据中心建立了数据库SRA（Sequence Read Archive，http://www.ncbi.nlm.nih.gov/Traces/sra）来存放共享的原始测序数据。Fastq格式示例如下：

@M03506:24:000000000-AM4K7:1:1101:14185:1000 1:N:0:58

AACTCCTACGGGAGGCAGCAGTGAGGAATATTGGTCAATGGACGAGAGTCTG

+

FFEFGGGGGGCGEECEGEGGGGGGG=DGEFGGGGGG6BEF:EGGCEGGGGFGGG

## 2. 数据处理过程和参数

Miseq测序得到的是Pair-End(PE)双端序列数据，对测得的Fastq数据进行质控处理，最终得到优质的Fasta数据。

具体参数如下：

1)利用Trimmomatic、Pear对Fastq数据进行质控。对于trimmomatic采用滑动窗口策略,窗口大小设置50bp，平均质量值为20，最小保留序列长度为120，Pear用于去除具有N的序列；

2)利用Flash、Pear根据PE的overlap关系对两端序列进行拼接(merge)处理，最小overlap设置10bp，错配率为0.1，得到Fasta序列；

3)根据已知数据库用uchime方法比对去除Fasta序列的嵌合体，对于未知数据库使用自比对(denovo)方法进行去除，同时去除不合要求的短序列。

软件：Trimmomatic(v0.36)、Pear(v0.9.6)、Flash(v1.20)、Vsearch(v2.7.1)

## 3. 有效数据统计

下机数据（Raw PE），在去除barcode和primer并拼接后得到raw\_tags，raw\_tags经进一步去除嵌合体、短序列后得到优质序列clean\_tags。具体结果统计如下表所示：

##### 表1 拼接结果统计

| #SampleID | raw\_tags | clean\_tags |
| --- | --- | --- |
| ES10 | 47284 | 41456 |
| ES11 | 48846 | 43610 |
| ES12 | 74011 | 71810 |
| ES13 | 47358 | 43873 |
| ES14 | 58118 | 54124 |
| ES15 | 47790 | 46199 |
| ES1 | 86767 | 77848 |
| ES2 | 77584 | 66629 |
| ES3 | 66515 | 54682 |
| ES4 | 84023 | 71558 |
| ES5 | 66956 | 56597 |
| ES6 | 102840 | 86376 |
| ES7 | 43608 | 38360 |
| ES8 | 74502 | 62916 |
| ES9 | 34648 | 31048 |
| ESC10 | 31994 | 30636 |
| ESC11 | 27621 | 25559 |
| ESC12 | 31719 | 30141 |
| ESC13 | 52282 | 49791 |
| ESC14 | 69992 | 67778 |
| ESC15 | 38995 | 36495 |
| ESC16 | 40212 | 38335 |
| ESC17 | 44355 | 41488 |
| ESC18 | 43350 | 41372 |
| ESC19 | 41933 | 39518 |
| ESC1 | 35818 | 34178 |
| ESC20 | 49886 | 47082 |
| ESC21 | 68666 | 65328 |
| ESC22 | 31064 | 29100 |
| ESC23 | 69678 | 66226 |
| ESC24 | 49318 | 46852 |
| ESC25 | 74101 | 71402 |
| ESC26 | 34011 | 31932 |
| ESC27 | 29209 | 27026 |
| ESC28 | 29761 | 28117 |
| ESC29 | 45130 | 43764 |
| ESC2 | 53613 | 50768 |
| ESC30 | 68129 | 64072 |
| ESC31 | 48508 | 45527 |
| ESC32 | 54054 | 51769 |
| ESC3 | 59738 | 57560 |
| ESC4 | 42594 | 41038 |
| ESC5 | 36917 | 35821 |
| ESC6 | 51781 | 50200 |
| ESC7 | 59229 | 54184 |
| ESC8 | 42336 | 39484 |
| ESC9 | 27015 | 25536 |
| Nor10 | 58576 | 52837 |
| Nor11 | 61342 | 55577 |
| Nor12 | 45880 | 43077 |
| Nor13 | 58441 | 53932 |
| Nor14 | 45795 | 43570 |
| Nor15 | 42950 | 41033 |
| Nor16 | 48858 | 46217 |
| Nor17 | 83132 | 73870 |
| Nor18 | 44013 | 41331 |
| Nor19 | 65499 | 61467 |
| Nor1 | 52407 | 47628 |
| Nor20 | 50333 | 42987 |
| Nor21 | 39579 | 35030 |
| Nor2 | 55899 | 51072 |
| Nor3 | 79428 | 73910 |
| Nor4 | 41858 | 38231 |
| Nor5 | 39634 | 37381 |
| Nor6 | 39912 | 38350 |
| Nor7 | 52739 | 50335 |
| Nor8 | 44907 | 41454 |
| Nor9 | 42046 | 40794 |

 *##### 注: raw\_tags为经过过滤低质量的fastq数据拼接得到的结果，clean\_tags为进一步对拼接结果去除嵌合体、短序列后得到的结果。*

## 4. 优质序列长度分布

#### 结果说明:

结果目录:**1.QC\_and\_Basic\_analysis/Clean\_Tags\_Distribution**

经数据预处理最终得到的优质序列分布统计，如下表所示：

##### 表2 优质序列分布统计

| Length(bp) | Sequences |
| --- | --- |
| distribute | reads |
| 0-200 | 0 |
| 200-260 | 5897 |
| 260-320 | 11259 |
| 320-360 | 576 |
| 360-380 | 225 |
| 380-400 | 48985 |
| 400-420 | 1070532 |
| 420-440 | 2126949 |
| 440-460 | 271 |
| 460-480 | 152 |
| 480-500 | 159 |
| 500-520 | 221 |
| 520-540 | 22 |
| 540-560 | 0 |
| 560-600 | 0 |

  

##### 图3 序列分布图

 *##### 注：横坐标为序列长度梯度，纵坐标为tags数。* 


## 5. OTUs聚类分析

OTUs(Operational Taxonomic Units)是在系统发生学或群体遗传学研究中，为了便于进行分析，人为给某一个分类单元（品系，属，种、分组等）设置的同一标志。要了解一个样品测序结果中的菌种、菌属等数目信息，就需要对序列进行归类操作（cluster）。通过归类操作，将序列按照彼此的相似性分归为许多小组，一个小组就是一个OTU。可根据不同的相似度水平，对所有序列进行OTU划分，通常对97%相似水平下的OTU进行生物信息统计分析。

#### 平台及软件

Qiime（Version 1.8.0http://qiime.org/)

vsearch(2.7.1 https://github.com/torognes/vsearch)

#### 分析方法

将clean tags用聚类(或降噪)方式生成OTUs。聚类方法可选 uparse[1],uclust[2],cdhit[3],ref参照数据库等方法;降噪法为unoise3[4]方法。默认为uparse聚类法。

#### 结果说明:

结果目录：**1.QC\_and\_Basic\_analysis/OTUs**

共产生 2157
个 OTU,经过抽平处理剩余 2134
,详细单个样本OTU数目见下表

##### 表3 单个样本的OTU数目统计

| SampleID | Final\_tags | OTUs |
| --- | --- | --- |
| Nor1 | 23656 | 303 |
| Nor2 | 23656 | 245 |
| Nor3 | 23656 | 304 |
| Nor4 | 23656 | 220 |
| Nor5 | 23656 | 214 |
| Nor6 | 23656 | 183 |
| Nor7 | 23656 | 224 |
| Nor8 | 23656 | 227 |
| Nor9 | 23656 | 156 |
| Nor10 | 23656 | 315 |
| Nor11 | 23656 | 230 |
| Nor12 | 23656 | 245 |
| Nor13 | 23656 | 238 |
| Nor14 | 23656 | 239 |
| Nor15 | 23656 | 190 |
| Nor16 | 23656 | 200 |
| Nor17 | 23656 | 188 |
| Nor18 | 23656 | 207 |
| Nor19 | 23656 | 128 |
| Nor20 | 23656 | 388 |
| Nor21 | 23656 | 295 |
| ES1 | 23656 | 358 |
| ES2 | 23656 | 376 |
| ES3 | 23656 | 446 |
| ES4 | 23656 | 520 |
| ES5 | 23656 | 431 |
| ES6 | 23656 | 402 |
| ES7 | 23656 | 332 |
| ES8 | 23656 | 454 |
| ES9 | 23656 | 415 |
| ES10 | 23656 | 382 |
| ES11 | 23656 | 325 |
| ES12 | 23656 | 49 |
| ES13 | 23656 | 291 |
| ES14 | 23656 | 271 |
| ES15 | 23656 | 197 |
| ESC1 | 23656 | 213 |
| ESC2 | 23656 | 322 |
| ESC3 | 23656 | 269 |
| ESC4 | 23656 | 222 |
| ESC5 | 23656 | 218 |
| ESC6 | 23656 | 337 |
| ESC7 | 23656 | 313 |
| ESC8 | 23656 | 288 |
| ESC9 | 23656 | 304 |
| ESC10 | 23656 | 289 |
| ESC11 | 23656 | 288 |
| ESC12 | 23656 | 252 |
| ESC13 | 23656 | 277 |
| ESC14 | 23656 | 335 |
| ESC15 | 23656 | 306 |
| ESC16 | 23656 | 294 |
| ESC17 | 23656 | 279 |
| ESC18 | 23656 | 265 |
| ESC19 | 23656 | 276 |
| ESC20 | 23656 | 364 |
| ESC21 | 23656 | 411 |
| ESC22 | 23656 | 449 |
| ESC23 | 23656 | 387 |
| ESC24 | 23656 | 437 |
| ESC25 | 23656 | 376 |
| ESC26 | 23656 | 456 |
| ESC27 | 23656 | 463 |
| ESC28 | 23656 | 432 |
| ESC29 | 23656 | 224 |
| ESC30 | 23656 | 399 |
| ESC31 | 23656 | 455 |
| ESC32 | 23656 | 200 |

 *##### 注:第一列为样本名称，第二列为最终OTU表中每个样本对应的tag数目，第三列为每个样本最终得到的OTU数目。*

  

#### 参考文献

**[1]** Edgar, R.C. (2013) UPARSE: Highly accurate OTU sequences from microbial amplicon reads, Nature Methods [Pubmed:23955772, dx.doi.org/10.1038/nmeth.2604].

**[2]**Edgar, R.C. (2010) Search and clustering orders of magnitude faster than BLAST, Bioinformatics 26(19), 2460-2461.doi: 10.1093/bioinformatics/btq461.

**[3]**Li et al (2006) Cd-hit: a fast program for clustering and comparing large sets of protein or nucleotide sequences.

**[4]**Rognes T, Flouri T, Nichols B, Quince C, Mahé F. (2016) VSEARCH: a versatile open source tool for metagenomics. PeerJ 4:e2584. doi: 10.7717/peerj.2584

## 6. OTU分布Venn图

#### 分析方法:

Venn图 ^[1]^ 可用于统计多个样本中所共有和独有的OTU数目，可以比较直观的表现环境样本的OTU数目组及其样本或分组之间的重叠情况。

  

软件：R语言工具统计和作图。

  

#### 结果说明:

结果目录：**1.QC\_and\_Basic\_analysis/OTUs\_Venn**

\*.png ：韦恩图。

  

- >

##### 图4 韦恩图

##### *注：不同的颜色代表不同的样本，不同颜色的圆圈重叠的区域是为交集，即为重叠几个颜色圈的相同OTU，相对于不重叠的部分则为独有OTU，韦恩图只能做2~7个圈，即2~7个分组或样本。*

#### 参考文献

**[1]**Fouts DE, Szpakowski S, Purushe J, Torralba M, Waterman RC, et al. (2012) Next Generation Sequencing to Define Prokaryotic and Fungal Diversity in the Bovine Rumen. PLoS ONE 7(11): e48289. doi:10.1371/journal.pone.0048289

## 7. OTU分布花瓣图

花瓣图是另一种表示样本/组间特有和共有OTU数目的展示方式。

#### 结果说明:

结果目录：**1.QC\_and\_Basic\_analysis/OTUs\_Flower**

##### 图5 花瓣图

##### *花瓣图以每个花瓣代表一个样品/组，中间的core数字代表的是所有样品共有的OTU数目，花瓣上的数字代表该样品/组特有的OTU数目。*

## 1. 测序结果格式介绍

原始数据下机后，结果以Fastq格式存储。Fastq文件包含每条测序序列的名称、碱基序列以及其对应的测序质量信息。每条read包含4行信息，其中第一行和第三行由文件识别标志和读段名（ID）组成（第一行以“@”开头，第三行以“+” 开头；第三行中 ID可以省略但“+”不能省略），第二行为碱基序列，而第四行是第二行中的序列内容每个碱基所对应的测序质量值。为方便保存和共享各实验室产生的高通量测序数据，NCBI数据中心建立了数据库SRA（Sequence Read Archive，http://www.ncbi.nlm.nih.gov/Traces/sra）来存放共享的原始测序数据。Fastq格式示例如下：

@M03506:24:000000000-AM4K7:1:1101:14185:1000 1:N:0:58

AACTCCTACGGGAGGCAGCAGTGAGGAATATTGGTCAATGGACGAGAGTCTG

+

FFEFGGGGGGCGEECEGEGGGGGGG=DGEFGGGGGG6BEF:EGGCEGGGGFGGG

## 2. 数据处理过程和参数

Miseq测序得到的是Pair-End(PE)双端序列数据，对测得的Fastq数据进行质控处理，最终得到优质的Fasta数据。

具体参数如下：

1)利用Trimmomatic、Pear对Fastq数据进行质控。对于trimmomatic采用滑动窗口策略,窗口大小设置50bp，平均质量值为20，最小保留序列长度为120，Pear用于去除具有N的序列；

2)利用Flash、Pear根据PE的overlap关系对两端序列进行拼接(merge)处理，最小overlap设置10bp，错配率为0.1，得到Fasta序列；

3)根据已知数据库用uchime方法比对去除Fasta序列的嵌合体，对于未知数据库使用自比对(denovo)方法进行去除，同时去除不合要求的短序列。

软件：Trimmomatic(v0.36)、Pear(v0.9.6)、Flash(v1.20)、Vsearch(v2.7.1)

## 3. 有效数据统计

下机数据（Raw PE），在去除barcode和primer并拼接后得到raw\_tags，raw\_tags经进一步去除嵌合体、短序列后得到优质序列clean\_tags。具体结果统计如下表所示：

##### 表1 拼接结果统计

| #SampleID | raw\_tags | clean\_tags |
| --- | --- | --- |
| ES10 | 47284 | 41456 |
| ES11 | 48846 | 43610 |
| ES12 | 74011 | 71810 |
| ES13 | 47358 | 43873 |
| ES14 | 58118 | 54124 |
| ES15 | 47790 | 46199 |
| ES1 | 86767 | 77848 |
| ES2 | 77584 | 66629 |
| ES3 | 66515 | 54682 |
| ES4 | 84023 | 71558 |
| ES5 | 66956 | 56597 |
| ES6 | 102840 | 86376 |
| ES7 | 43608 | 38360 |
| ES8 | 74502 | 62916 |
| ES9 | 34648 | 31048 |
| ESC10 | 31994 | 30636 |
| ESC11 | 27621 | 25559 |
| ESC12 | 31719 | 30141 |
| ESC13 | 52282 | 49791 |
| ESC14 | 69992 | 67778 |
| ESC15 | 38995 | 36495 |
| ESC16 | 40212 | 38335 |
| ESC17 | 44355 | 41488 |
| ESC18 | 43350 | 41372 |
| ESC19 | 41933 | 39518 |
| ESC1 | 35818 | 34178 |
| ESC20 | 49886 | 47082 |
| ESC21 | 68666 | 65328 |
| ESC22 | 31064 | 29100 |
| ESC23 | 69678 | 66226 |
| ESC24 | 49318 | 46852 |
| ESC25 | 74101 | 71402 |
| ESC26 | 34011 | 31932 |
| ESC27 | 29209 | 27026 |
| ESC28 | 29761 | 28117 |
| ESC29 | 45130 | 43764 |
| ESC2 | 53613 | 50768 |
| ESC30 | 68129 | 64072 |
| ESC31 | 48508 | 45527 |
| ESC32 | 54054 | 51769 |
| ESC3 | 59738 | 57560 |
| ESC4 | 42594 | 41038 |
| ESC5 | 36917 | 35821 |
| ESC6 | 51781 | 50200 |
| ESC7 | 59229 | 54184 |
| ESC8 | 42336 | 39484 |
| ESC9 | 27015 | 25536 |
| Nor10 | 58576 | 52837 |
| Nor11 | 61342 | 55577 |
| Nor12 | 45880 | 43077 |
| Nor13 | 58441 | 53932 |
| Nor14 | 45795 | 43570 |
| Nor15 | 42950 | 41033 |
| Nor16 | 48858 | 46217 |
| Nor17 | 83132 | 73870 |
| Nor18 | 44013 | 41331 |
| Nor19 | 65499 | 61467 |
| Nor1 | 52407 | 47628 |
| Nor20 | 50333 | 42987 |
| Nor21 | 39579 | 35030 |
| Nor2 | 55899 | 51072 |
| Nor3 | 79428 | 73910 |
| Nor4 | 41858 | 38231 |
| Nor5 | 39634 | 37381 |
| Nor6 | 39912 | 38350 |
| Nor7 | 52739 | 50335 |
| Nor8 | 44907 | 41454 |
| Nor9 | 42046 | 40794 |

 *##### 注: raw\_tags为经过过滤低质量的fastq数据拼接得到的结果，clean\_tags为进一步对拼接结果去除嵌合体、短序列后得到的结果。*

## 4. 优质序列长度分布

#### 结果说明:

结果目录:**1.QC\_and\_Basic\_analysis/Clean\_Tags\_Distribution**

经数据预处理最终得到的优质序列分布统计，如下表所示：

##### 表2 优质序列分布统计

| Length(bp) | Sequences |
| --- | --- |
| distribute | reads |
| 0-200 | 0 |
| 200-260 | 5897 |
| 260-320 | 11259 |
| 320-360 | 576 |
| 360-380 | 225 |
| 380-400 | 48985 |
| 400-420 | 1070532 |
| 420-440 | 2126949 |
| 440-460 | 271 |
| 460-480 | 152 |
| 480-500 | 159 |
| 500-520 | 221 |
| 520-540 | 22 |
| 540-560 | 0 |
| 560-600 | 0 |

  

##### 图3 序列分布图

 *##### 注：横坐标为序列长度梯度，纵坐标为tags数。*

## 5. OTUs聚类分析

OTUs(Operational Taxonomic Units)是在系统发生学或群体遗传学研究中，为了便于进行分析，人为给某一个分类单元（品系，属，种、分组等）设置的同一标志。要了解一个样品测序结果中的菌种、菌属等数目信息，就需要对序列进行归类操作（cluster）。通过归类操作，将序列按照彼此的相似性分归为许多小组，一个小组就是一个OTU。可根据不同的相似度水平，对所有序列进行OTU划分，通常对97%相似水平下的OTU进行生物信息统计分析。

#### 平台及软件

Qiime（Version 1.8.0http://qiime.org/)

vsearch(2.7.1 https://github.com/torognes/vsearch)

#### 分析方法

将clean tags用聚类(或降噪)方式生成OTUs。聚类方法可选 uparse[1],uclust[2],cdhit[3],ref参照数据库等方法;降噪法为unoise3[4]方法。默认为uparse聚类法。

#### 结果说明:

结果目录：**1.QC\_and\_Basic\_analysis/OTUs**

共产生 2157
个 OTU,经过抽平处理剩余 2134
,详细单个样本OTU数目见下表

##### 表3 单个样本的OTU数目统计

| SampleID | Final\_tags | OTUs |
| --- | --- | --- |
| Nor1 | 23656 | 303 |
| Nor2 | 23656 | 245 |
| Nor3 | 23656 | 304 |
| Nor4 | 23656 | 220 |
| Nor5 | 23656 | 214 |
| Nor6 | 23656 | 183 |
| Nor7 | 23656 | 224 |
| Nor8 | 23656 | 227 |
| Nor9 | 23656 | 156 |
| Nor10 | 23656 | 315 |
| Nor11 | 23656 | 230 |
| Nor12 | 23656 | 245 |
| Nor13 | 23656 | 238 |
| Nor14 | 23656 | 239 |
| Nor15 | 23656 | 190 |
| Nor16 | 23656 | 200 |
| Nor17 | 23656 | 188 |
| Nor18 | 23656 | 207 |
| Nor19 | 23656 | 128 |
| Nor20 | 23656 | 388 |
| Nor21 | 23656 | 295 |
| ES1 | 23656 | 358 |
| ES2 | 23656 | 376 |
| ES3 | 23656 | 446 |
| ES4 | 23656 | 520 |
| ES5 | 23656 | 431 |
| ES6 | 23656 | 402 |
| ES7 | 23656 | 332 |
| ES8 | 23656 | 454 |
| ES9 | 23656 | 415 |
| ES10 | 23656 | 382 |
| ES11 | 23656 | 325 |
| ES12 | 23656 | 49 |
| ES13 | 23656 | 291 |
| ES14 | 23656 | 271 |
| ES15 | 23656 | 197 |
| ESC1 | 23656 | 213 |
| ESC2 | 23656 | 322 |
| ESC3 | 23656 | 269 |
| ESC4 | 23656 | 222 |
| ESC5 | 23656 | 218 |
| ESC6 | 23656 | 337 |
| ESC7 | 23656 | 313 |
| ESC8 | 23656 | 288 |
| ESC9 | 23656 | 304 |
| ESC10 | 23656 | 289 |
| ESC11 | 23656 | 288 |
| ESC12 | 23656 | 252 |
| ESC13 | 23656 | 277 |
| ESC14 | 23656 | 335 |
| ESC15 | 23656 | 306 |
| ESC16 | 23656 | 294 |
| ESC17 | 23656 | 279 |
| ESC18 | 23656 | 265 |
| ESC19 | 23656 | 276 |
| ESC20 | 23656 | 364 |
| ESC21 | 23656 | 411 |
| ESC22 | 23656 | 449 |
| ESC23 | 23656 | 387 |
| ESC24 | 23656 | 437 |
| ESC25 | 23656 | 376 |
| ESC26 | 23656 | 456 |
| ESC27 | 23656 | 463 |
| ESC28 | 23656 | 432 |
| ESC29 | 23656 | 224 |
| ESC30 | 23656 | 399 |
| ESC31 | 23656 | 455 |
| ESC32 | 23656 | 200 |

 *##### 注:第一列为样本名称，第二列为最终OTU表中每个样本对应的tag数目，第三列为每个样本最终得到的OTU数目。*

  

#### 参考文献

**[1]** Edgar, R.C. (2013) UPARSE: Highly accurate OTU sequences from microbial amplicon reads, Nature Methods [Pubmed:23955772, dx.doi.org/10.1038/nmeth.2604].

**[2]**Edgar, R.C. (2010) Search and clustering orders of magnitude faster than BLAST, Bioinformatics 26(19), 2460-2461.doi: 10.1093/bioinformatics/btq461.

**[3]**Li et al (2006) Cd-hit: a fast program for clustering and comparing large sets of protein or nucleotide sequences.

**[4]**Rognes T, Flouri T, Nichols B, Quince C, Mahé F. (2016) VSEARCH: a versatile open source tool for metagenomics. PeerJ 4:e2584. doi: 10.7717/peerj.2584

## 6. OTU分布Venn图

#### 分析方法:

Venn图 ^[1]^ 可用于统计多个样本中所共有和独有的OTU数目，可以比较直观的表现环境样本的OTU数目组及其样本或分组之间的重叠情况。

  

软件：R语言工具统计和作图。

  

#### 结果说明:

结果目录：**1.QC\_and\_Basic\_analysis/OTUs\_Venn**

\*.png ：韦恩图。

  

- >

##### 图4 韦恩图

##### *注：不同的颜色代表不同的样本，不同颜色的圆圈重叠的区域是为交集，即为重叠几个颜色圈的相同OTU，相对于不重叠的部分则为独有OTU，韦恩图只能做2~7个圈，即2~7个分组或样本。*

#### 参考文献

**[1]**Fouts DE, Szpakowski S, Purushe J, Torralba M, Waterman RC, et al. (2012) Next Generation Sequencing to Define Prokaryotic and Fungal Diversity in the Bovine Rumen. PLoS ONE 7(11): e48289. doi:10.1371/journal.pone.0048289

## 7. OTU分布花瓣图

花瓣图是另一种表示样本/组间特有和共有OTU数目的展示方式。

#### 结果说明:

结果目录：**1.QC\_and\_Basic\_analysis/OTUs\_Flower**

##### 图5 花瓣图

##### *花瓣图以每个花瓣代表一个样品/组，中间的core数字代表的是所有样品共有的OTU数目，花瓣上的数字代表该样品/组特有的OTU数目。*

# Alpha多样性分析/Alpha diversity analysis

- all
- 稀释性曲线
- Shannon-Wiener
- Rank abundance曲线
- Specaccum物种累积
- alpha多样性指数

## 1. 稀释性曲线（Rarefaction curve)

#### 分析方法

稀释性曲线  ^[1]^  是从样本中随机抽取一定数量的个体，统计这些个体所代表的物种数目，并以个体数与物种数来构建曲线。它可以用来比较测序数据量不同的样本中物种的丰富度，也可以用来说明样本的测序数据量是否合理。采用对序列进行随机抽样的方法，以抽到的序列数与它们所能代表OTU的数目构建rarefaction curve，当曲线趋向平坦时，说明测序数据量合理，更多的数据量只会产生少量新的OTU，反之则表明继续测序还可能产生较多新的OTU。因此，通过作稀释性曲线，可得出样品的测序深度情况。

  

软件：基于OTU聚类结果利用mothur做rarefaction分析，利用R语言工具制作曲线图。

#### 结果说明

结果目录： **2.Alpha\_diversity\_analysis/Rarefaction**

otu.\*.rarefaction:每个样品在不同取样值的OTU数目表 Numsampled：随机取样数 （预设为从1开始每增加50计算一次直到本次该样品取样数）

  

##### 图6 样品稀释曲线图

##### *注：横坐标：随机抽取的测序数据量；纵坐标：观测到的OTU数量。*

  

#### 参考文献

**[1]** Katherine R Amato. et al. Habitat degradation impacts black howler monkey (Alouatta pigra) gastrointestinal microbiomes. The ISME Journal (2013) 7, 1344–1353; doi:10.1038/ismej.

## 2. Shannon-Wiener曲线

#### 分析方法:

Shannon-Wiener  ^[1]^  是反映样本中微生物多样性的指数，利用各样本的测序量在不同测序深度时的微生物多样性指数构建曲线，以此反映各样本在不同测序数量时的微生物多样性。当曲线趋向平坦时，说明测序数据量足够大，可以反映样本中绝大多数的微生物信息。

  

软件：基于OTU聚类结果利用mothur计算不同随机抽样下的shannon值，利用R语言工具制作曲线图。

  

#### 结果说明:

结果目录： **2.Alpha\_diversity\_analysis/Shannon**

otu.\*.shannon：每个样本在不同取样值的shannon指数表

  

- >

图7 Shannon-Wiener曲线图

##### *注：横坐标为测序深度，纵坐标为shannon指数*

## 3. Rank-Abundance曲线

#### 分析方法:

Rank-abundance  ^[1]^  曲线是分析多样性的一种方式。构建方法是统计单一样本中，每一个OTU所含的序列数，将OTUs按丰度（所含有的序列条数）由大到小等级排序，再以OTU等级为横坐标，以每个OTU中所含的序列数（也可用OTU中序列数的相对百分含量）为纵坐标做图。

Rank-abundance曲线可用来解释多样性的两个方面，即物种丰度和物种均匀度。在水平方向，物种的丰度由曲线的宽度来反映，物种的丰度越高，曲线在横轴上的范围越大；曲线的形状（平滑程度）反映了样本中物种的均度，曲线越平缓，物种分布越均匀。

  

软件：R语言工具统计和作图。

  

#### 结果说明:

结果目录： **2.Alpha\_diversity\_analysis/Rank\_abundance**

abundance.pdf：多样本曲线图

  
  

### Rank\_abundance

- >

##### 图8 Rank abundance图

##### *注：横坐标：OTU等级，“10^3”代表样本中按照丰度排列第1000位的OTU；纵坐标：该等级OTU中序列数的相对百分含量，即属于该OTU的序列数除以总序列数，纵坐标轴上数字，例如“10^-2”代表相对丰度为0.01，“10^-1”代表相对丰度为0.1，依次类推。*

  

#### 参考文献

**[1]**Scott T Bates, Jose C Clemente, et al. Global biogeography of highly diverse protistan communities in soil. The ISME Journal (2013) 7, 652–659; doi:10.1038/ismej.2012.147

## 4. Specaccum物种累积曲线

#### 分析方法:

物种累积曲线  ^[1]^  ( species accumulation curves) 是用于描述随着样本量的加大物种增加的状况，是调查样本的物种组成和预测样本中物种丰度的有效工具，在生物多样性和群落调查中，被广泛用于样本量是否充分的判断以及物种丰富度( species richness) 的估计。因此，通过物种累积曲线不仅可以判断样本量是否充分，在样本量充分的前提下，运用物种累积曲线还可以对物种丰富度进行预测。

  

#### 结果说明:

结果目录： **2.Alpha\_diversity\_analysis/Specaccum**

  

图9 Specaccum物种累积曲线

##### *注：横坐标：样本量；纵坐标：抽样后OTU数目。结果反映了持续抽样下新OTU（新物种）出现的速率。在一定范围内，随着样本量的加大，若曲线表现为急剧上升则表示群落中有大量物种被发现；当曲线趋于平缓，则表示此环境中的物种并不会随样本量的增加而显著增多。利用物种累积曲线可以作为对样本量是否充分的判断，曲线急剧上升表明样本量不足，需要增加抽样量；反之，则表明抽样充分，可以进行数据分析，一般10个样本以上才有意义。*

  

#### 参考文献

**[1]**Maughan H, Wang PW, Diaz Caballero J, Fung P, Gong Y, et al. (2012) Analysis of the Cystic Fibrosis Lung Microbiota via Serial Illumina Sequencing of Bacterial 16S rRNA Hypervariable Regions. PLoS ONE 7(10): e45791. doi:10.1371/journal.pone.0045791

## 5. alpha多样性指数

多样性指数及其稀释曲线可通过以下链接查看： alpha多样性指数

经过最低序列数的样本数进行抽平分析，其多样性指数如下列表所示：

##### 表4 alpha多样性指数统计表

|  |  |  |  |  |  |  |
| --- | --- | --- | --- | --- | --- | --- |
| SampleID | chao1 | goods\_coverage | observed\_species | PD\_whole\_tree | shannon | simpson |
| ES1 | 380.57 | 1.00 | 357.90 | 26.20 | 5.42 | 0.91 |
| ES10 | 545.68 | 1.00 | 382.00 | 32.53 | 5.54 | 0.91 |
| ES11 | 416.29 | 1.00 | 325.00 | 27.96 | 5.52 | 0.94 |
| ES12 | 62.20 | 1.00 | 49.00 | 7.28 | 3.65 | 0.90 |
| ES13 | 387.01 | 1.00 | 290.80 | 28.69 | 4.40 | 0.84 |
| ES14 | 361.59 | 1.00 | 271.00 | 25.02 | 6.01 | 0.97 |
| ES15 | 282.65 | 1.00 | 197.00 | 21.42 | 5.52 | 0.96 |
| ES2 | 481.62 | 1.00 | 376.00 | 29.59 | 5.38 | 0.93 |
| ES3 | 631.00 | 1.00 | 446.00 | 34.17 | 6.47 | 0.97 |
| ES4 | 660.59 | 1.00 | 520.00 | 40.81 | 6.87 | 0.98 |
| ES5 | 568.97 | 1.00 | 431.00 | 32.30 | 6.42 | 0.97 |
| ES6 | 607.15 | 0.99 | 402.00 | 33.06 | 6.34 | 0.97 |
| ES7 | 421.00 | 1.00 | 332.00 | 27.86 | 6.57 | 0.98 |
| ES8 | 642.90 | 1.00 | 454.00 | 42.07 | 6.72 | 0.98 |
| ES9 | 478.03 | 1.00 | 415.00 | 33.72 | 6.43 | 0.95 |
| ESC1 | 304.50 | 1.00 | 213.00 | 21.18 | 5.78 | 0.97 |
| ESC10 | 436.87 | 1.00 | 288.90 | 35.78 | 3.49 | 0.81 |
| ESC11 | 416.23 | 1.00 | 287.80 | 44.33 | 4.77 | 0.93 |
| ESC12 | 339.57 | 1.00 | 252.00 | 22.43 | 5.33 | 0.94 |
| ESC13 | 456.08 | 1.00 | 277.00 | 25.68 | 4.95 | 0.94 |
| ESC14 | 472.28 | 1.00 | 335.00 | 59.02 | 5.20 | 0.94 |
| ESC15 | 457.67 | 1.00 | 306.00 | 26.18 | 3.65 | 0.77 |
| ESC16 | 419.16 | 1.00 | 294.00 | 26.71 | 4.68 | 0.87 |
| ESC17 | 421.50 | 1.00 | 279.00 | 24.47 | 5.13 | 0.93 |
| ESC18 | 350.58 | 1.00 | 265.00 | 26.32 | 5.20 | 0.94 |
| ESC19 | 345.00 | 1.00 | 276.00 | 23.05 | 5.07 | 0.93 |
| ESC2 | 448.83 | 1.00 | 322.00 | 122.61 | 3.55 | 0.69 |
| ESC20 | 471.33 | 1.00 | 364.00 | 30.06 | 5.06 | 0.93 |
| ESC21 | 558.00 | 0.99 | 411.00 | 85.98 | 2.39 | 0.61 |
| ESC22 | 584.84 | 0.99 | 449.00 | 70.55 | 3.87 | 0.82 |
| ESC23 | 546.20 | 0.99 | 387.00 | 75.49 | 4.83 | 0.93 |
| ESC24 | 674.11 | 0.99 | 436.90 | 87.13 | 4.18 | 0.81 |
| ESC25 | 531.77 | 0.99 | 376.00 | 120.04 | 4.35 | 0.86 |
| ESC26 | 622.98 | 0.99 | 456.00 | 72.93 | 4.59 | 0.88 |
| ESC27 | 612.13 | 0.99 | 463.00 | 63.06 | 5.16 | 0.93 |
| ESC28 | 627.74 | 0.99 | 432.00 | 78.33 | 3.35 | 0.80 |
| ESC29 | 280.89 | 1.00 | 224.00 | 21.73 | 4.46 | 0.85 |
| ESC3 | 388.38 | 1.00 | 269.00 | 67.83 | 3.83 | 0.84 |
| ESC30 | 581.30 | 0.99 | 398.90 | 102.98 | 4.95 | 0.91 |
| ESC31 | 641.29 | 0.99 | 455.00 | 98.43 | 5.31 | 0.93 |
| ESC32 | 292.50 | 1.00 | 200.00 | 18.27 | 2.17 | 0.65 |
| ESC4 | 304.97 | 1.00 | 222.00 | 21.24 | 4.09 | 0.77 |
| ESC5 | 355.52 | 1.00 | 218.00 | 22.08 | 2.87 | 0.61 |
| ESC6 | 472.42 | 1.00 | 336.90 | 68.00 | 3.83 | 0.76 |
| ESC7 | 403.73 | 1.00 | 313.00 | 93.82 | 5.11 | 0.93 |
| ESC8 | 376.67 | 1.00 | 288.00 | 25.95 | 4.77 | 0.87 |
| ESC9 | 405.25 | 1.00 | 304.00 | 26.02 | 4.89 | 0.89 |
| Nor1 | 428.08 | 1.00 | 303.00 | 26.91 | 4.39 | 0.83 |
| Nor10 | 400.31 | 1.00 | 315.00 | 28.41 | 5.55 | 0.96 |
| Nor11 | 284.17 | 1.00 | 230.00 | 22.58 | 5.56 | 0.96 |
| Nor12 | 317.62 | 1.00 | 245.00 | 24.45 | 5.14 | 0.96 |
| Nor13 | 331.95 | 1.00 | 238.00 | 21.88 | 5.33 | 0.96 |
| Nor14 | 321.85 | 1.00 | 238.90 | 24.49 | 3.78 | 0.75 |
| Nor15 | 273.09 | 1.00 | 190.00 | 20.28 | 3.43 | 0.70 |
| Nor16 | 276.24 | 1.00 | 200.00 | 21.52 | 4.98 | 0.95 |
| Nor17 | 233.49 | 1.00 | 188.00 | 17.95 | 5.09 | 0.94 |
| Nor18 | 226.38 | 1.00 | 207.00 | 18.93 | 3.51 | 0.72 |
| Nor19 | 170.27 | 1.00 | 128.00 | 15.74 | 5.54 | 0.97 |
| Nor2 | 379.61 | 1.00 | 245.00 | 29.93 | 5.10 | 0.94 |
| Nor20 | 471.69 | 1.00 | 388.00 | 34.12 | 5.53 | 0.94 |
| Nor21 | 387.57 | 1.00 | 295.00 | 28.29 | 4.63 | 0.85 |
| Nor3 | 360.00 | 1.00 | 304.00 | 26.91 | 6.21 | 0.97 |
| Nor4 | 365.70 | 1.00 | 220.00 | 23.81 | 4.57 | 0.91 |
| Nor5 | 298.52 | 1.00 | 213.90 | 25.46 | 4.59 | 0.93 |
| Nor6 | 260.90 | 1.00 | 183.00 | 17.65 | 4.53 | 0.90 |
| Nor7 | 321.75 | 1.00 | 224.00 | 23.59 | 4.57 | 0.87 |
| Nor8 | 305.96 | 1.00 | 227.00 | 21.26 | 5.33 | 0.95 |
| Nor9 | 243.14 | 1.00 | 156.00 | 17.28 | 1.91 | 0.39 |

##### 图10 alpha多样性指数box图

##### *箱形图主要包含5个数据节点，将一组数据从大到小排列，分别计算出他的上边缘，上四分位数，中位数，下四分位数，下边缘。横坐标为样本/分组名称，纵坐标为Alpha指数。*

#### 分析方法

群落生态学中研究微生物多样性，通过单样品的多样性分析（Alpha多样性）可以反映微生物群落的丰度和多样性，包括一系列统计学分析指数估计环境群落的物种丰度和多样性。

#### 各指数算法如下：

**Chao1**:即菌种丰富度指数，用以估计群落中的OTU数目。其公式如下：

Schao1=Sobs+n1(n1-1)/2(n2+1)

其中Schao1为估计的OTU数，Sobs为观测到的OTU数，n1为只有一条序列的OTU数目，n2为只有两条序列的OTU数目。

**Simpson**:用来估算样品中微生物多样性指数之一，结果中交付为1-D值。其公式如下：

1-D=1－∑(Ni/N)^2

Simpson指数兼顾丰富度和均匀度，其中的D值反映的是在同一个样本中随机的抽取2个个体，这两个个体来自同一个类的概率。即D值是发现的某一特定物种的个体的总数（Ni）除以发现的个体总数（N）。

D值在0-1之间，0表示无限多样，1表示没有多样性。所以1-D值越大，说明群落多样性越高。

**observed\_species**：随测序深度的增加，实际观测到OTU的个数。

**goods\_coverage**：观测深度，其公式为：

C=1-n1/N

其中，n1为只有含一条序列的OTU数目，N为抽样中出现的总的序列数目。该指数表征各样本文库的覆盖率，其数值越高，则样本中序列被测出的概率越高，而没有被测出的概率越低。该指数反映本次测序结果是否代表了样本中微生物的真实情况。

**PD\_whole\_tree**:谱系多样性,是兼顾考虑了物种丰度以及进化距离的多样性指数。是基于系统发育树来计算的一种多样性指数，它用各个样品中OTUs的代表序列构建出系统发育树的距离，将某一个样品中的所有代表序列的枝长加和，从而得到的数值。数值越大，群落多样性越高。

**Shannon**：用来估算样品中微生物多样性指数之一。其公式如下：

H=－∑（Pi）（㏑Pi）

其中Pi为样品中属于第i种的个体的比例，如样品总个体数为N，第i种个体数为ni，则Pi=ni/N 各种之间，个体分配越均匀，H值就越大。如果每一个体都属于不同的种，多样性指数就最大； 如果每一个体都属于同一种，则其多样性指数就最小。所以shannon值越大，说明群落多样性越高。

#### 分析软件：

qiime  ^[1]^  version v.1.8.0http://qiime.org/scripts/alpha\_rarefaction.html)，分析时通常对数据进行抽平处理，以该数据中样本数据最少的数据量作计算，并且通常采用0.97的相似度进行分析。

#### 结果说明:

结果目录： **2.Alpha\_diversity\_analysis/Alpha\_diversity\_index**

  

#### 参考文献

**[1]** Miller G E, Engen P A, Gillevet P M, et al. Lower neighborhood socioeconomic status associated with reduced diversity of the colonic microbiota in healthy adults[J]. PLoS One, 2016, 11(2): e0148952.

## 1. 稀释性曲线（Rarefaction curve)

#### 分析方法

稀释性曲线  ^[1]^  是从样本中随机抽取一定数量的个体，统计这些个体所代表的物种数目，并以个体数与物种数来构建曲线。它可以用来比较测序数据量不同的样本中物种的丰富度，也可以用来说明样本的测序数据量是否合理。采用对序列进行随机抽样的方法，以抽到的序列数与它们所能代表OTU的数目构建rarefaction curve，当曲线趋向平坦时，说明测序数据量合理，更多的数据量只会产生少量新的OTU，反之则表明继续测序还可能产生较多新的OTU。因此，通过作稀释性曲线，可得出样品的测序深度情况。

  

软件：基于OTU聚类结果利用mothur做rarefaction分析，利用R语言工具制作曲线图。

#### 结果说明

结果目录： **2.Alpha\_diversity\_analysis/Rarefaction**

otu.\*.rarefaction:每个样品在不同取样值的OTU数目表 Numsampled：随机取样数 （预设为从1开始每增加50计算一次直到本次该样品取样数）

  

##### 图6 样品稀释曲线图

##### *注：横坐标：随机抽取的测序数据量；纵坐标：观测到的OTU数量。*

  

#### 参考文献

**[1]** Katherine R Amato. et al. Habitat degradation impacts black howler monkey (Alouatta pigra) gastrointestinal microbiomes. The ISME Journal (2013) 7, 1344–1353; doi:10.1038/ismej.

## 2. Shannon-Wiener曲线

#### 分析方法:

Shannon-Wiener  ^[1]^  是反映样本中微生物多样性的指数，利用各样本的测序量在不同测序深度时的微生物多样性指数构建曲线，以此反映各样本在不同测序数量时的微生物多样性。当曲线趋向平坦时，说明测序数据量足够大，可以反映样本中绝大多数的微生物信息。

  

软件：基于OTU聚类结果利用mothur计算不同随机抽样下的shannon值，利用R语言工具制作曲线图。

  

#### 结果说明:

结果目录： **2.Alpha\_diversity\_analysis/Shannon**

otu.\*.shannon：每个样本在不同取样值的shannon指数表

  

- >

图7 Shannon-Wiener曲线图

##### *注：横坐标为测序深度，纵坐标为shannon指数*

## 3. Rank-Abundance曲线

#### 分析方法:

Rank-abundance  ^[1]^  曲线是分析多样性的一种方式。构建方法是统计单一样本中，每一个OTU所含的序列数，将OTUs按丰度（所含有的序列条数）由大到小等级排序，再以OTU等级为横坐标，以每个OTU中所含的序列数（也可用OTU中序列数的相对百分含量）为纵坐标做图。

Rank-abundance曲线可用来解释多样性的两个方面，即物种丰度和物种均匀度。在水平方向，物种的丰度由曲线的宽度来反映，物种的丰度越高，曲线在横轴上的范围越大；曲线的形状（平滑程度）反映了样本中物种的均度，曲线越平缓，物种分布越均匀。

  

软件：R语言工具统计和作图。

  

#### 结果说明:

结果目录： **2.Alpha\_diversity\_analysis/Rank\_abundance**

abundance.pdf：多样本曲线图

  
  

### Rank\_abundance

- >

##### 图8 Rank abundance图

##### *注：横坐标：OTU等级，“10^3”代表样本中按照丰度排列第1000位的OTU；纵坐标：该等级OTU中序列数的相对百分含量，即属于该OTU的序列数除以总序列数，纵坐标轴上数字，例如“10^-2”代表相对丰度为0.01，“10^-1”代表相对丰度为0.1，依次类推。*

  

#### 参考文献

**[1]**Scott T Bates, Jose C Clemente, et al. Global biogeography of highly diverse protistan communities in soil. The ISME Journal (2013) 7, 652–659; doi:10.1038/ismej.2012.147

## 4. Specaccum物种累积曲线

#### 分析方法:

物种累积曲线  ^[1]^  ( species accumulation curves) 是用于描述随着样本量的加大物种增加的状况，是调查样本的物种组成和预测样本中物种丰度的有效工具，在生物多样性和群落调查中，被广泛用于样本量是否充分的判断以及物种丰富度( species richness) 的估计。因此，通过物种累积曲线不仅可以判断样本量是否充分，在样本量充分的前提下，运用物种累积曲线还可以对物种丰富度进行预测。

  

#### 结果说明:

结果目录： **2.Alpha\_diversity\_analysis/Specaccum**

  

图9 Specaccum物种累积曲线

##### *注：横坐标：样本量；纵坐标：抽样后OTU数目。结果反映了持续抽样下新OTU（新物种）出现的速率。在一定范围内，随着样本量的加大，若曲线表现为急剧上升则表示群落中有大量物种被发现；当曲线趋于平缓，则表示此环境中的物种并不会随样本量的增加而显著增多。利用物种累积曲线可以作为对样本量是否充分的判断，曲线急剧上升表明样本量不足，需要增加抽样量；反之，则表明抽样充分，可以进行数据分析，一般10个样本以上才有意义。*

  

#### 参考文献

**[1]**Maughan H, Wang PW, Diaz Caballero J, Fung P, Gong Y, et al. (2012) Analysis of the Cystic Fibrosis Lung Microbiota via Serial Illumina Sequencing of Bacterial 16S rRNA Hypervariable Regions. PLoS ONE 7(10): e45791. doi:10.1371/journal.pone.0045791

## 5. alpha多样性指数

多样性指数及其稀释曲线可通过以下链接查看： alpha多样性指数

经过最低序列数的样本数进行抽平分析，其多样性指数如下列表所示：

##### 表4 alpha多样性指数统计表

|  |  |  |  |  |  |  |
| --- | --- | --- | --- | --- | --- | --- |
| SampleID | chao1 | goods\_coverage | observed\_species | PD\_whole\_tree | shannon | simpson |
| ES1 | 380.57 | 1.00 | 357.90 | 26.20 | 5.42 | 0.91 |
| ES10 | 545.68 | 1.00 | 382.00 | 32.53 | 5.54 | 0.91 |
| ES11 | 416.29 | 1.00 | 325.00 | 27.96 | 5.52 | 0.94 |
| ES12 | 62.20 | 1.00 | 49.00 | 7.28 | 3.65 | 0.90 |
| ES13 | 387.01 | 1.00 | 290.80 | 28.69 | 4.40 | 0.84 |
| ES14 | 361.59 | 1.00 | 271.00 | 25.02 | 6.01 | 0.97 |
| ES15 | 282.65 | 1.00 | 197.00 | 21.42 | 5.52 | 0.96 |
| ES2 | 481.62 | 1.00 | 376.00 | 29.59 | 5.38 | 0.93 |
| ES3 | 631.00 | 1.00 | 446.00 | 34.17 | 6.47 | 0.97 |
| ES4 | 660.59 | 1.00 | 520.00 | 40.81 | 6.87 | 0.98 |
| ES5 | 568.97 | 1.00 | 431.00 | 32.30 | 6.42 | 0.97 |
| ES6 | 607.15 | 0.99 | 402.00 | 33.06 | 6.34 | 0.97 |
| ES7 | 421.00 | 1.00 | 332.00 | 27.86 | 6.57 | 0.98 |
| ES8 | 642.90 | 1.00 | 454.00 | 42.07 | 6.72 | 0.98 |
| ES9 | 478.03 | 1.00 | 415.00 | 33.72 | 6.43 | 0.95 |
| ESC1 | 304.50 | 1.00 | 213.00 | 21.18 | 5.78 | 0.97 |
| ESC10 | 436.87 | 1.00 | 288.90 | 35.78 | 3.49 | 0.81 |
| ESC11 | 416.23 | 1.00 | 287.80 | 44.33 | 4.77 | 0.93 |
| ESC12 | 339.57 | 1.00 | 252.00 | 22.43 | 5.33 | 0.94 |
| ESC13 | 456.08 | 1.00 | 277.00 | 25.68 | 4.95 | 0.94 |
| ESC14 | 472.28 | 1.00 | 335.00 | 59.02 | 5.20 | 0.94 |
| ESC15 | 457.67 | 1.00 | 306.00 | 26.18 | 3.65 | 0.77 |
| ESC16 | 419.16 | 1.00 | 294.00 | 26.71 | 4.68 | 0.87 |
| ESC17 | 421.50 | 1.00 | 279.00 | 24.47 | 5.13 | 0.93 |
| ESC18 | 350.58 | 1.00 | 265.00 | 26.32 | 5.20 | 0.94 |
| ESC19 | 345.00 | 1.00 | 276.00 | 23.05 | 5.07 | 0.93 |
| ESC2 | 448.83 | 1.00 | 322.00 | 122.61 | 3.55 | 0.69 |
| ESC20 | 471.33 | 1.00 | 364.00 | 30.06 | 5.06 | 0.93 |
| ESC21 | 558.00 | 0.99 | 411.00 | 85.98 | 2.39 | 0.61 |
| ESC22 | 584.84 | 0.99 | 449.00 | 70.55 | 3.87 | 0.82 |
| ESC23 | 546.20 | 0.99 | 387.00 | 75.49 | 4.83 | 0.93 |
| ESC24 | 674.11 | 0.99 | 436.90 | 87.13 | 4.18 | 0.81 |
| ESC25 | 531.77 | 0.99 | 376.00 | 120.04 | 4.35 | 0.86 |
| ESC26 | 622.98 | 0.99 | 456.00 | 72.93 | 4.59 | 0.88 |
| ESC27 | 612.13 | 0.99 | 463.00 | 63.06 | 5.16 | 0.93 |
| ESC28 | 627.74 | 0.99 | 432.00 | 78.33 | 3.35 | 0.80 |
| ESC29 | 280.89 | 1.00 | 224.00 | 21.73 | 4.46 | 0.85 |
| ESC3 | 388.38 | 1.00 | 269.00 | 67.83 | 3.83 | 0.84 |
| ESC30 | 581.30 | 0.99 | 398.90 | 102.98 | 4.95 | 0.91 |
| ESC31 | 641.29 | 0.99 | 455.00 | 98.43 | 5.31 | 0.93 |
| ESC32 | 292.50 | 1.00 | 200.00 | 18.27 | 2.17 | 0.65 |
| ESC4 | 304.97 | 1.00 | 222.00 | 21.24 | 4.09 | 0.77 |
| ESC5 | 355.52 | 1.00 | 218.00 | 22.08 | 2.87 | 0.61 |
| ESC6 | 472.42 | 1.00 | 336.90 | 68.00 | 3.83 | 0.76 |
| ESC7 | 403.73 | 1.00 | 313.00 | 93.82 | 5.11 | 0.93 |
| ESC8 | 376.67 | 1.00 | 288.00 | 25.95 | 4.77 | 0.87 |
| ESC9 | 405.25 | 1.00 | 304.00 | 26.02 | 4.89 | 0.89 |
| Nor1 | 428.08 | 1.00 | 303.00 | 26.91 | 4.39 | 0.83 |
| Nor10 | 400.31 | 1.00 | 315.00 | 28.41 | 5.55 | 0.96 |
| Nor11 | 284.17 | 1.00 | 230.00 | 22.58 | 5.56 | 0.96 |
| Nor12 | 317.62 | 1.00 | 245.00 | 24.45 | 5.14 | 0.96 |
| Nor13 | 331.95 | 1.00 | 238.00 | 21.88 | 5.33 | 0.96 |
| Nor14 | 321.85 | 1.00 | 238.90 | 24.49 | 3.78 | 0.75 |
| Nor15 | 273.09 | 1.00 | 190.00 | 20.28 | 3.43 | 0.70 |
| Nor16 | 276.24 | 1.00 | 200.00 | 21.52 | 4.98 | 0.95 |
| Nor17 | 233.49 | 1.00 | 188.00 | 17.95 | 5.09 | 0.94 |
| Nor18 | 226.38 | 1.00 | 207.00 | 18.93 | 3.51 | 0.72 |
| Nor19 | 170.27 | 1.00 | 128.00 | 15.74 | 5.54 | 0.97 |
| Nor2 | 379.61 | 1.00 | 245.00 | 29.93 | 5.10 | 0.94 |
| Nor20 | 471.69 | 1.00 | 388.00 | 34.12 | 5.53 | 0.94 |
| Nor21 | 387.57 | 1.00 | 295.00 | 28.29 | 4.63 | 0.85 |
| Nor3 | 360.00 | 1.00 | 304.00 | 26.91 | 6.21 | 0.97 |
| Nor4 | 365.70 | 1.00 | 220.00 | 23.81 | 4.57 | 0.91 |
| Nor5 | 298.52 | 1.00 | 213.90 | 25.46 | 4.59 | 0.93 |
| Nor6 | 260.90 | 1.00 | 183.00 | 17.65 | 4.53 | 0.90 |
| Nor7 | 321.75 | 1.00 | 224.00 | 23.59 | 4.57 | 0.87 |
| Nor8 | 305.96 | 1.00 | 227.00 | 21.26 | 5.33 | 0.95 |
| Nor9 | 243.14 | 1.00 | 156.00 | 17.28 | 1.91 | 0.39 |

##### 图10 alpha多样性指数box图

##### *箱形图主要包含5个数据节点，将一组数据从大到小排列，分别计算出他的上边缘，上四分位数，中位数，下四分位数，下边缘。横坐标为样本/分组名称，纵坐标为Alpha指数。*

#### 分析方法

群落生态学中研究微生物多样性，通过单样品的多样性分析（Alpha多样性）可以反映微生物群落的丰度和多样性，包括一系列统计学分析指数估计环境群落的物种丰度和多样性。

#### 各指数算法如下：

**Chao1**:即菌种丰富度指数，用以估计群落中的OTU数目。其公式如下：

Schao1=Sobs+n1(n1-1)/2(n2+1)

其中Schao1为估计的OTU数，Sobs为观测到的OTU数，n1为只有一条序列的OTU数目，n2为只有两条序列的OTU数目。

**Simpson**:用来估算样品中微生物多样性指数之一，结果中交付为1-D值。其公式如下：

1-D=1－∑(Ni/N)^2

Simpson指数兼顾丰富度和均匀度，其中的D值反映的是在同一个样本中随机的抽取2个个体，这两个个体来自同一个类的概率。即D值是发现的某一特定物种的个体的总数（Ni）除以发现的个体总数（N）。

D值在0-1之间，0表示无限多样，1表示没有多样性。所以1-D值越大，说明群落多样性越高。

**observed\_species**：随测序深度的增加，实际观测到OTU的个数。

**goods\_coverage**：观测深度，其公式为：

C=1-n1/N

其中，n1为只有含一条序列的OTU数目，N为抽样中出现的总的序列数目。该指数表征各样本文库的覆盖率，其数值越高，则样本中序列被测出的概率越高，而没有被测出的概率越低。该指数反映本次测序结果是否代表了样本中微生物的真实情况。

**PD\_whole\_tree**:谱系多样性,是兼顾考虑了物种丰度以及进化距离的多样性指数。是基于系统发育树来计算的一种多样性指数，它用各个样品中OTUs的代表序列构建出系统发育树的距离，将某一个样品中的所有代表序列的枝长加和，从而得到的数值。数值越大，群落多样性越高。

**Shannon**：用来估算样品中微生物多样性指数之一。其公式如下：

H=－∑（Pi）（㏑Pi）

其中Pi为样品中属于第i种的个体的比例，如样品总个体数为N，第i种个体数为ni，则Pi=ni/N 各种之间，个体分配越均匀，H值就越大。如果每一个体都属于不同的种，多样性指数就最大； 如果每一个体都属于同一种，则其多样性指数就最小。所以shannon值越大，说明群落多样性越高。

#### 分析软件：

qiime  ^[1]^  version v.1.8.0http://qiime.org/scripts/alpha\_rarefaction.html)，分析时通常对数据进行抽平处理，以该数据中样本数据最少的数据量作计算，并且通常采用0.97的相似度进行分析。

#### 结果说明:

结果目录： **2.Alpha\_diversity\_analysis/Alpha\_diversity\_index**

  

#### 参考文献

**[1]** Miller G E, Engen P A, Gillevet P M, et al. Lower neighborhood socioeconomic status associated with reduced diversity of the colonic microbiota in healthy adults[J]. PLoS One, 2016, 11(2): e0148952.

# 物种注释/Taxonomic Analysis

- all
- OTUs注释
- 物种组成分析
- Core microbiome分析
- 属水平进化发生树
- 样本聚类柱状图
- Krona物种展示
- OTU及其分类学heatmap分析

## 1. OTUs注释

#### 分析方法

为了得到每个OTU对应的物种分类信息，采用RDP Classifier算法（默认）或blast、uclust consensus taxonomy assigner等方法对OTU代表序列进行比对分析，并在各个水平(kingdom，phylum，class，order，family，genus，species)注释其群落的物种信息。

  

#### 数据库选择

#### 16s细菌和古菌核糖体数据库（不指定则默认silva128）：

Silva  ^[1]^  (Release128/132 http://www.arb-silva.de)

RDP  ^[2]^  (V16 http://rdp.cme.msu.edu/)

Greengene  ^[3]^  (Release 13.5 http://greengenes.secondgenome.com/)

#### 18S

Silva  ^[4]^  (Release128/132 http://www.arb-silva.de)

#### 口腔专用

HOMD (Release15.1 http://www.homd.org/)

#### ITS真菌：

Unite  ^[5]^  (Release 7.2 http://unite.ut.ee/index.php)的真菌数据库。

#### 其他：

NCBI Nucleotide(https://www.ncbi.nlm.nih.gov/)数据库

blast ^[6]^ (2.6.0+ http://blast.ncbi.nlm.nih.gov) ,evalue设置为1e-5

#### 软件及算法添加如下：

Qiime平台 （ http://qiime.org/scripts/assign\_taxonomy.html）

Uclust Consensus Taxon Assigner  ^[7]^  (UCLUST v1.2.22q http://www.drive5.com/uclust/downloads1\_2\_22q.html)，置信度阈值为0.9

RDP Classifier（默认算法）  ^[8]^  (http://sourceforge.net/projects/rdp-classifier/)，置信度阈值为0.7

#### 参考文献

**[1]** Quast C, Pruesse E, Yilmaz P, Gerken J, Schweer T, Yarza P, Peplies J, Glöckner FO . The SILVA ribosomal RNA gene database project: improved data processing and web-based tools. Nucl. Acids Res. 41 (D1): D590-D596(2013)

**[2]** Cole, J. R., Q. Wang, E. Cardenas, J. Fish, B. Chai, R. J. Farris, A. S. Kulam-Syed-Mohideen, D. M. McGarrell, T. Marsh, G. M. Garrity, and J. M. Tiedje. The Ribosomal Database Project: improved alignments and new tools for rRNA analysis. Nucleic Acids Res. 37 (Database issue): D141-145;doi: 10.1093/nar/gkn879] [PMID: 19004872] (2009)

**[3]** DeSantis, T. Z., P. Hugenholtz, N. Larsen, M. Rojas, E. L. Brodie, K. Keller, T. Huber, D. Dalevi, P. Hu, and G. L. Andersen. 2006. Greengenes, a Chimera-Checked 16S rRNA Gene Database and Workbench Compatible with ARB. Appl Environ Microbiol 72:5069-72.

**[4]** Urmas Kõljalg, R. Henrik Nilsson, Kessy Abarenkov, Leho Tedersoo, et al. Towards a unified paradigm for sequence-based identification of fungi. Molecular Ecology (2013)22,5271–5277DOI: 10.1111/mec.12481

**[5]** Altschul S F, Madden T L, Schäffer A A, et al. Gapped BLAST and PSI-BLAST: a new generation of protein database search programs[J]. Nucleic acids research, 1997, 25(17): 3389-3402.

**[6]** Edgar R C. Search and clustering orders of magnitude faster than BLAST[J]. Bioinformatics, 2010, 26(19): 2460-2461.

**[7]** Wang Q, Garrity GM, Tiedje JM, Cole JR. 2007. Naive Bayesian classifier for rapid assignment of rRNA sequences into the new bacterial taxonomy.Appl Environ Microb 73(16): 5261-5267.

## 2. Core microbiome分析

#### 分析方法:

Core microbiome分析，是通过qiime平台算出覆盖样本的微生物组情况，起初以50%的样本数再以5%累加递增，求其百分数覆盖下的样本数目所包含共有的OTU情况。这里我们统计所有样本下共有的OTU，并鉴别分类水平将其注释。

  

软件：qiime

  

#### 结果说明:

结果目录： **3.Taxonomic\_analysis/Core\_microbiome**

  

##### 图11 Core microbiome

##### *注：共有OTU随着样本数目的增加情况。横坐标为样本百分数，纵坐标为共有OTU数。*

  
  

##### 表5 所有样本共有OTU表

|  |  |  |
| --- | --- | --- |
| OTUs | level | taxonomy |
| OTU\_23 | species | s\_\_Streptococcus\_pneumoniae |
| OTU\_47 | genus | g\_\_Curvibacter |

##### *注：上表是共有OTU及其注释情况。当共有OTU过多时，仅显示其20个，详细见附件。*

## 3. 物种组成分析

#### 分析方法

根据分类学分析结果，可以得知一个或多个样本在各分类水平上的分类学比对情况。在结果中，包含了两个信息：

  

1. 样本中含有何种微生物

2. 样本中各微生物的序列数，即各微生物的相对丰度。

  

因此，可以使用统计学的分析方法，观测样本在不同分类水平上的群落结构。将多个样本的群落结构分析放在一起对比时，还可以观测其变化情况。这里我们用柱状图可视化观察不同样本(或者分组)的物种组成情况  ^[1]^ ，用饼图可视化观察同一个样本（或者统一分组）的菌种含量情况，这里仅显示一组门水平的柱状图和饼图，其他水平和分组详见附件。 我们从门、纲、目、科、属等五个水平上作柱状图和饼图。*注：单个样本只作饼图分析*

  

#### 软件：R语言作图工具

  

#### 结果说明

结果目录：**3.Taxonomic\_analysis/Barplot**

  

##### 图12 物种组成分析柱状图

##### *横坐标为样本名称，纵坐标为物种在该样本中的相对丰度。图中显示的是相对丰度1%以上的物种信息。*

  

#### 参考文献

**[1]**Lisa Oberauner, Christin Zachow, Stefan Lackner, et al. The ignored diversity: complex bacterial communities in intensive care units revealed by 16S pyrosequencing. SCIENTIFIC REPORTS. 3 :1413 .DOI: 10.1038/srep01413.(2013)

## 4. 属水平进化发生树

#### 分析方法:

软件及算法：mafft进行序列对齐，fasttree进行建树。并根据python语言把其物种（属）的丰度和进化关系进行可视化展示。

#### 结果说明:

结果目录： **3.Taxonomic\_analysis/Genus\_phylogeny**

##### 图14 属水平进化发生树

##### *以属为单位选取对应丰度最大的OTU的代表序列进行建树。图标第一列为属水平所对应的门水平，第二列为样本或分组信息。以属为单位选取对应丰度最大的OTU的代表序列进行建树，进化树外圈展示每个属在不同样本/分组中的相对丰度。色块的长度代表相对丰度的高低。*

## 5. 样本聚类柱状图

#### 分析方法:

基于Unweighted Unifrac距离矩阵，UPGMA方法聚类建树，并将聚类结果与各样品在门、纲、目、科、属水平上的物种相对丰度整合展示。

#### 结果说明:

结果目录： **3.Taxonomic\_analysis/Levels\_Bar\_tree**

##### 图15 样本聚类柱状图

##### *左边为样本间层次聚类分析，右边为样品的群落结构柱状图。横坐标为物种在该样本中的相对丰度，纵坐标为样本名称。*

## 6. Krona物种展示

#### 分析方法:

使用Krona^[1]^对物种注释结果进行可视化展示,展示结果中,圆圈从内到外依次代表不同的分类级别,扇形的大小代表不同OTU注释结果的相对比例。详细介绍见下图

  

软件：KronaTools

  

#### 结果说明:

结果目录： **3.Taxonomic\_analysis/Krona**

  

##### 图16 Krona样例图展示

结果链接如下所示(点击查看)：

krona.html

  

#### 参考文献

**[1]**Interactive metagenomic visualization in a Web browser, Brian D Ondov, Nicholas H Bergman and Adam M Phillippy, BMC Bioinformatics201112:385, DOI: 10.1186/1471-2105-12-385.

## 7. OTU及其分类学heatmap分析

#### 分析方法:

Heatmap  ^[1]^  可以用颜色变化来反映二维矩阵或表格中的数据信息，它可以直观地将数据值的大小以定义的颜色深浅表示出来。常根据需要将数据进行物种或样本间丰>度相似性聚类，将聚类后数据表示在heatmap图上，可将高丰度和低丰度的物种分块聚集，通过颜色梯度及相似程度来反映多个样本在各分类水平上群落组成的相似性和差异性。

  

软件及算法：R语言vegan包，vegdist和hclust进行距离计算和聚类分析；距离算法：Bray-Curtis，聚类方法：complete。图中颜色梯度可自定为两种或两种以上颜色渐变色。样本间和物种间聚类树枝可自定是否画出。

#### 结果说明:

结果目录： **3.Taxonomic\_analysis/Heatmap**

图17 OTU及其分类学水平heatmap图

##### *左边为物种/OTU的关系聚类分析。横坐标为样本名称，纵坐标为OTU/物种，颜色深浅代表OTU/物种丰度的高低。这里仅显示排行靠前的优势物种。*

  

#### 参考文献

**[1]**Elie Jami, Adi Israel, et al. Exploring the bovine rumen bacterial community from birth to adulthood. The ISME Journal advance online publication, 21 February 2013; doi:10.1038/ismej.2013.2

## 1. OTUs注释

#### 分析方法

为了得到每个OTU对应的物种分类信息，采用RDP Classifier算法（默认）或blast、uclust consensus taxonomy assigner等方法对OTU代表序列进行比对分析，并在各个水平(kingdom，phylum，class，order，family，genus，species)注释其群落的物种信息。

  

#### 数据库选择

#### 16s细菌和古菌核糖体数据库（不指定则默认silva128）：

Silva  ^[1]^  (Release128/132 http://www.arb-silva.de)

RDP  ^[2]^  (V16 http://rdp.cme.msu.edu/)

Greengene  ^[3]^  (Release 13.5 http://greengenes.secondgenome.com/)

#### 18S

Silva  ^[4]^  (Release128/132 http://www.arb-silva.de)

#### 口腔专用

HOMD (Release15.1 http://www.homd.org/)

#### ITS真菌：

Unite  ^[5]^  (Release 7.2 http://unite.ut.ee/index.php)的真菌数据库。

#### 其他：

NCBI Nucleotide(https://www.ncbi.nlm.nih.gov/)数据库

blast ^[6]^ (2.6.0+ http://blast.ncbi.nlm.nih.gov) ,evalue设置为1e-5

#### 软件及算法添加如下：

Qiime平台 （ http://qiime.org/scripts/assign\_taxonomy.html）

Uclust Consensus Taxon Assigner  ^[7]^  (UCLUST v1.2.22q http://www.drive5.com/uclust/downloads1\_2\_22q.html)，置信度阈值为0.9

RDP Classifier（默认算法）  ^[8]^  (http://sourceforge.net/projects/rdp-classifier/)，置信度阈值为0.7

#### 参考文献

**[1]** Quast C, Pruesse E, Yilmaz P, Gerken J, Schweer T, Yarza P, Peplies J, Glöckner FO . The SILVA ribosomal RNA gene database project: improved data processing and web-based tools. Nucl. Acids Res. 41 (D1): D590-D596(2013)

**[2]** Cole, J. R., Q. Wang, E. Cardenas, J. Fish, B. Chai, R. J. Farris, A. S. Kulam-Syed-Mohideen, D. M. McGarrell, T. Marsh, G. M. Garrity, and J. M. Tiedje. The Ribosomal Database Project: improved alignments and new tools for rRNA analysis. Nucleic Acids Res. 37 (Database issue): D141-145;doi: 10.1093/nar/gkn879] [PMID: 19004872] (2009)

**[3]** DeSantis, T. Z., P. Hugenholtz, N. Larsen, M. Rojas, E. L. Brodie, K. Keller, T. Huber, D. Dalevi, P. Hu, and G. L. Andersen. 2006. Greengenes, a Chimera-Checked 16S rRNA Gene Database and Workbench Compatible with ARB. Appl Environ Microbiol 72:5069-72.

**[4]** Urmas Kõljalg, R. Henrik Nilsson, Kessy Abarenkov, Leho Tedersoo, et al. Towards a unified paradigm for sequence-based identification of fungi. Molecular Ecology (2013)22,5271–5277DOI: 10.1111/mec.12481

**[5]** Altschul S F, Madden T L, Schäffer A A, et al. Gapped BLAST and PSI-BLAST: a new generation of protein database search programs[J]. Nucleic acids research, 1997, 25(17): 3389-3402.

**[6]** Edgar R C. Search and clustering orders of magnitude faster than BLAST[J]. Bioinformatics, 2010, 26(19): 2460-2461.

**[7]** Wang Q, Garrity GM, Tiedje JM, Cole JR. 2007. Naive Bayesian classifier for rapid assignment of rRNA sequences into the new bacterial taxonomy.Appl Environ Microb 73(16): 5261-5267.

## 3. 物种组成分析

#### 分析方法

根据分类学分析结果，可以得知一个或多个样本在各分类水平上的分类学比对情况。在结果中，包含了两个信息：

  

1. 样本中含有何种微生物

2. 样本中各微生物的序列数，即各微生物的相对丰度。

  

因此，可以使用统计学的分析方法，观测样本在不同分类水平上的群落结构。将多个样本的群落结构分析放在一起对比时，还可以观测其变化情况。这里我们用柱状图可视化观察不同样本(或者分组)的物种组成情况  ^[1]^ ，用饼图可视化观察同一个样本（或者统一分组）的菌种含量情况，这里仅显示一组门水平的柱状图和饼图，其他水平和分组详见附件。 我们从门、纲、目、科、属等五个水平上作柱状图和饼图。*注：单个样本只作饼图分析*

  

#### 软件：R语言作图工具

  

#### 结果说明

结果目录：**3.Taxonomic\_analysis/Barplot**

  

##### 图12 物种组成分析柱状图

##### *横坐标为样本名称，纵坐标为物种在该样本中的相对丰度。图中显示的是相对丰度1%以上的物种信息。*

  

#### 参考文献

**[1]**Lisa Oberauner, Christin Zachow, Stefan Lackner, et al. The ignored diversity: complex bacterial communities in intensive care units revealed by 16S pyrosequencing. SCIENTIFIC REPORTS. 3 :1413 .DOI: 10.1038/srep01413.(2013)

## 2. Core microbiome分析

#### 分析方法:

Core microbiome分析，是通过qiime平台算出覆盖样本的微生物组情况，起初以50%的样本数再以5%累加递增，求其百分数覆盖下的样本数目所包含共有的OTU情况。这里我们统计所有样本下共有的OTU，并鉴别分类水平将其注释。

  

软件：qiime

  

#### 结果说明:

结果目录： **3.Taxonomic\_analysis/Core\_microbiome**

  

##### 图11 Core microbiome

##### *注：共有OTU随着样本数目的增加情况。横坐标为样本百分数，纵坐标为共有OTU数。*

  
  

##### 表5 所有样本共有OTU表

|  |  |  |
| --- | --- | --- |
| OTUs | level | taxonomy |
| OTU\_23 | species | s\_\_Streptococcus\_pneumoniae |
| OTU\_47 | genus | g\_\_Curvibacter |

##### *注：上表是共有OTU及其注释情况。当共有OTU过多时，仅显示其20个，详细见附件。*

## 4. 属水平进化发生树

#### 分析方法:

软件及算法：mafft进行序列对齐，fasttree进行建树。并根据python语言把其物种（属）的丰度和进化关系进行可视化展示。

#### 结果说明:

结果目录： **3.Taxonomic\_analysis/Genus\_phylogeny**

##### 图14 属水平进化发生树

##### *以属为单位选取对应丰度最大的OTU的代表序列进行建树。图标第一列为属水平所对应的门水平，第二列为样本或分组信息。以属为单位选取对应丰度最大的OTU的代表序列进行建树，进化树外圈展示每个属在不同样本/分组中的相对丰度。色块的长度代表相对丰度的高低。*

## 5. 样本聚类柱状图

#### 分析方法:

基于Unweighted Unifrac距离矩阵，UPGMA方法聚类建树，并将聚类结果与各样品在门、纲、目、科、属水平上的物种相对丰度整合展示。

#### 结果说明:

结果目录： **3.Taxonomic\_analysis/Levels\_Bar\_tree**

##### 图15 样本聚类柱状图

##### *左边为样本间层次聚类分析，右边为样品的群落结构柱状图。横坐标为物种在该样本中的相对丰度，纵坐标为样本名称。*

## 6. Krona物种展示

#### 分析方法:

使用Krona^[1]^对物种注释结果进行可视化展示,展示结果中,圆圈从内到外依次代表不同的分类级别,扇形的大小代表不同OTU注释结果的相对比例。详细介绍见下图

  

软件：KronaTools

  

#### 结果说明:

结果目录： **3.Taxonomic\_analysis/Krona**

  

##### 图16 Krona样例图展示

结果链接如下所示(点击查看)：

krona.html

  

#### 参考文献

**[1]**Interactive metagenomic visualization in a Web browser, Brian D Ondov, Nicholas H Bergman and Adam M Phillippy, BMC Bioinformatics201112:385, DOI: 10.1186/1471-2105-12-385.

## 7. OTU及其分类学heatmap分析

#### 分析方法:

Heatmap  ^[1]^  可以用颜色变化来反映二维矩阵或表格中的数据信息，它可以直观地将数据值的大小以定义的颜色深浅表示出来。常根据需要将数据进行物种或样本间丰>度相似性聚类，将聚类后数据表示在heatmap图上，可将高丰度和低丰度的物种分块聚集，通过颜色梯度及相似程度来反映多个样本在各分类水平上群落组成的相似性和差异性。

  

软件及算法：R语言vegan包，vegdist和hclust进行距离计算和聚类分析；距离算法：Bray-Curtis，聚类方法：complete。图中颜色梯度可自定为两种或两种以上颜色渐变色。样本间和物种间聚类树枝可自定是否画出。

#### 结果说明:

结果目录： **3.Taxonomic\_analysis/Heatmap**

图17 OTU及其分类学水平heatmap图

##### *左边为物种/OTU的关系聚类分析。横坐标为样本名称，纵坐标为OTU/物种，颜色深浅代表OTU/物种丰度的高低。这里仅显示排行靠前的优势物种。*

  

#### 参考文献

**[1]**Elie Jami, Adi Israel, et al. Exploring the bovine rumen bacterial community from birth to adulthood. The ISME Journal advance online publication, 21 February 2013; doi:10.1038/ismej.2013.2

# Beta多样性分析/Beta diversity analysis

- all
- 基于unifrac的heatmap
- 基于unifrac的PCoA分析
- Hcluster分析
- PCA分析
- PLS-DA分析
- NMDS分析

## 1. 基于unifrac 的 heatmap

为了更好的显示多个样本之间的距离关系，将加权与未加权的unifrac样本距离矩阵用热力图表现出来，如下图所示

#### 结果说明:

结果目录：**4.Beta\_diversity\_analysis/(Un)Weighted\_Unifrac\_distance\_heatmap**

  

图18 基于unirfac heatmap图

##### *左侧和上侧为基于unifrac计算样本与样本间的距离关系。横、纵坐标均为样本名称，颜色深浅代表样本与样本间相似性的高低。*

对于有生物学重复的多样本距离矩阵，计算它们组内的距离关系绘制箱线图如下所示:

图19 基于unifrac distance 箱线图

## 2. 基于unifrac及bray-curtis的PCoA分析

#### 分析方法:

PCoA分析，即主坐标分析（principal co-ordinates analysis），也是一种非约束性的数据降维分析方法，可用来研究样本群落组成的相似性或差异性，与PCA分析类似；主要区别在于，PCA基于欧氏距离，PCoA基于除欧氏距离以外的其它距离，通过降维找出影响样本群落组成差异的潜在主成分。

PCoA分析，首先对一系列的特征值和特征向量进行排序，然后选择排在前几位的最主要特征值，并将其表现在坐标系里，结果相当于是距离矩阵的一个旋转，它没有改变样本点之间的相互位置关系，只是改变了坐标系统。基于bray-curtis的PCoA用二维图进行绘图，基于加权与非加权的PCOA以网页版三维图进行展示，其结果如下

  

软件：R、qiime

  

#### 结果说明:

结果目录： **4.Beta\_diversity\_analysis/Bray-Curtis\_PCoA**

  

加权PCoA分析： 3D\_weighted\_PCoA\_index.html

非加权PCoA分析： 3D\_unweighted\_PCoA\_index.html

图20 基于bray-curtis的PCoA

##### *注：图中不同颜色或形状的点代表不同的分组情况，横、纵坐标轴的刻度是相对距离，无实际意义。PC1、PC2分别代表对于两组样本微生物组成发生偏移的疑似影响因素，需要结合样本特征信息归纳总结。*

## 3. Hcluster分析

#### 分析方法:

利用树枝结构描述和比较多个样本间的相似性和差异关系。首先使用描述群落组成关系和结构的算法计算样本间的距离，即根据beta多样性距离矩阵进行层次聚类（Hierarchical cluatering）分析  ^[1]^  ，使用非加权组平均法UPGMA（Unweighted pair group method with arithmetic mean）算法构建树状结构，得到树状关系形式用于可视化分析。

  

软件：Qiime计算beta多样性距离矩阵，计算距离矩阵的算法为bray curtis:

SA,i = 表示A样本中第i个OTU所含的序列数；

SB,i = 表示B样本中第i个OTU所含的序列数。

  

#### 结果说明:

结果目录： **4.Beta\_diversity\_analysis/Hcluster**

图21 基于bray curtis多样本聚类树

##### *注：树枝长度代表样本间的距离，越是相似的样本越能聚到一起。*

  

#### 参考文献

**[1]**Xiao-Tao Jiang ,Xin Peng, et al.Illumina Sequencing of 16S rRNA Tag Revealed Spatial Variations of Bacterial Communities in a Mangrove Wetland. Microb Ecol (2013) 66:96–104.DOI 10.1007/s00248-013-0238-8.

## 4. PCA分析

#### 分析方法:

PCA分析  ^[1]^  (Principal Component Analysis)，即主成分分析，是一种对数据进行简化分析的技术，这种方法可以有效的找出数据中最“主要”的元素和结构，去除噪音和冗余，将原有的复杂数据降维，揭示隐藏在复杂数据背后的简单结构。其优点是简单且无参数限制。通过分析不同样本OTU（97%相似性）组成可以反映样本间的差异和距离，PCA运用方差分解，将多组数据的差异反映在二维坐标图上，坐标轴取能够最大反映方差值的两个特征值。如样本组成越相似，反映在PCA图中的距离越近。不同环境间的样本可能表现出分散和聚集的分布情况，PCA结果中对样本差异性解释度最高的两个成分可以用于对假设因素进行验证。

  

软件：R语言PCA统计分析和作图。

  

#### 结果说明:

结果目录： **4.Beta\_diversity\_analysis/PCA**

##### 图22 基于OTU水平的PCA分析

##### *注：不同颜色或形状的点代表不同的样本分组情况，横、纵坐标轴的刻度是相对距离，无实际意义。贡献率则是对其解释程度的大小，可以对假设因素进行评估验证。*

  

#### 参考文献

**[1]**Yu Wang, Hua-Fang Sheng, et al. Comparison of the Levels of Bacterial Diversity in Freshwater,Intertidal Wetland, and Marine Sediments by Using Millions of Illumina Tags. Appl. Environ. Microbiol. 2012, 78(23):8264. DOI: 10.1128/AEM.01821-12

## 5. PLS-DA分析

#### 分析方法:

PLS-DA分析  ^[1]^ 偏最小二乘法判别分析（Partial Least Squares Discrimination Analysis简称PLS-DA）是一种用于判别分析的多变量统计分析方法。判别分析是一种根据观察或测量到的若干变量值，来判断研究对象如何分类的常用统计分析方法。不同于主成分分析（ PCA）法，它是一种有监督的判别分析统计方法。该方法运用PLS-DA建立微生物含量与样品类别之间的关系模型，来实现对样品类别的预测。

  

软件：R语言作图。

  

#### 结果说明:

结果目录： **4.Beta\_diversity\_analysis/PLS-DA**

##### 图23 基于OTU的偏最小二乘法判别分析

## 6. NMDS分析

#### 分析方法:

非度量多维尺度法（NMDS）  ^[1]^  是一种将多维空间的研究对象（样本或变量）简化到低维空间进行定位、分析和归类，同时又保留对象间原始关系的数据分析方法。适用于无法获得研究对象间精确的相似性或相异性数据，仅能得到他们之间等级关系数据的情形。其基本特征是将对象间的相似性或相异性数据看成点间距离的单调函数，在保持原始数据次序关系的基础上，用新的相同次序的数据列替换原始数据进行度量型多维尺度分析。换句话说，当资料不适合直接进行变量型多维尺度分析时，对其进行变量变换，再采用变量型多维尺度分析，对原始资料而言，就称之为非度量型多维尺度分析。其特点是根据样本中包含的物种信息，以点的形式反映在多维空间上，而对不同样本间的差异程度，则是通过点与点间的距离体现的，最终获得样本的空间定位点图。

  

软件：R语言vegan与ggplot2等软件包进行数据计算NMDS分析和作图。

  

#### 结果说明:

结果目录： **4.Beta\_diversity\_analysis/NMDS**

图24 OTU水平NMDS分析

##### *注：一般说来同组样本之间均在一个圈内说明组间差异不明显，而组与组之间圈无交集则说明各组之间有一定差异性。*

#### 参考文献

**[1]**Magali Noval Rivas, PhD, Oliver T. Burton, et al. A microbita signature associated with experimental food allergy promotes allergic senitization and anaphylaxis. The Journal of Allergy and Clinical Immunology.Volume 131, Issue 1 , Pages 201-212, January 2013

## 1. 基于unifrac 的 heatmap

为了更好的显示多个样本之间的距离关系，将加权与未加权的unifrac样本距离矩阵用热力图表现出来，如下图所示

#### 结果说明:

结果目录：**4.Beta\_diversity\_analysis/(Un)Weighted\_Unifrac\_distance\_heatmap**

  

图18 基于unirfac heatmap图

##### *左侧和上侧为基于unifrac计算样本与样本间的距离关系。横、纵坐标均为样本名称，颜色深浅代表样本与样本间相似性的高低。*

对于有生物学重复的多样本距离矩阵，计算它们组内的距离关系绘制箱线图如下所示:

图19 基于unifrac distance 箱线图

## 2. 基于unifrac及bray-curtis的PCoA分析

#### 分析方法:

PCoA分析，即主坐标分析（principal co-ordinates analysis），也是一种非约束性的数据降维分析方法，可用来研究样本群落组成的相似性或差异性，与PCA分析类似；主要区别在于，PCA基于欧氏距离，PCoA基于除欧氏距离以外的其它距离，通过降维找出影响样本群落组成差异的潜在主成分。

PCoA分析，首先对一系列的特征值和特征向量进行排序，然后选择排在前几位的最主要特征值，并将其表现在坐标系里，结果相当于是距离矩阵的一个旋转，它没有改变样本点之间的相互位置关系，只是改变了坐标系统。基于bray-curtis的PCoA用二维图进行绘图，基于加权与非加权的PCOA以网页版三维图进行展示，其结果如下

  

软件：R、qiime

  

#### 结果说明:

结果目录： **4.Beta\_diversity\_analysis/Bray-Curtis\_PCoA**

  

加权PCoA分析： 3D\_weighted\_PCoA\_index.html

非加权PCoA分析： 3D\_unweighted\_PCoA\_index.html

图20 基于bray-curtis的PCoA

##### *注：图中不同颜色或形状的点代表不同的分组情况，横、纵坐标轴的刻度是相对距离，无实际意义。PC1、PC2分别代表对于两组样本微生物组成发生偏移的疑似影响因素，需要结合样本特征信息归纳总结。*

## 3. Hcluster分析

#### 分析方法:

利用树枝结构描述和比较多个样本间的相似性和差异关系。首先使用描述群落组成关系和结构的算法计算样本间的距离，即根据beta多样性距离矩阵进行层次聚类（Hierarchical cluatering）分析  ^[1]^  ，使用非加权组平均法UPGMA（Unweighted pair group method with arithmetic mean）算法构建树状结构，得到树状关系形式用于可视化分析。

  

软件：Qiime计算beta多样性距离矩阵，计算距离矩阵的算法为bray curtis:

SA,i = 表示A样本中第i个OTU所含的序列数；

SB,i = 表示B样本中第i个OTU所含的序列数。

  

#### 结果说明:

结果目录： **4.Beta\_diversity\_analysis/Hcluster**

图21 基于bray curtis多样本聚类树

##### *注：树枝长度代表样本间的距离，越是相似的样本越能聚到一起。*

  

#### 参考文献

**[1]**Xiao-Tao Jiang ,Xin Peng, et al.Illumina Sequencing of 16S rRNA Tag Revealed Spatial Variations of Bacterial Communities in a Mangrove Wetland. Microb Ecol (2013) 66:96–104.DOI 10.1007/s00248-013-0238-8.

## 4. PCA分析

#### 分析方法:

PCA分析  ^[1]^  (Principal Component Analysis)，即主成分分析，是一种对数据进行简化分析的技术，这种方法可以有效的找出数据中最“主要”的元素和结构，去除噪音和冗余，将原有的复杂数据降维，揭示隐藏在复杂数据背后的简单结构。其优点是简单且无参数限制。通过分析不同样本OTU（97%相似性）组成可以反映样本间的差异和距离，PCA运用方差分解，将多组数据的差异反映在二维坐标图上，坐标轴取能够最大反映方差值的两个特征值。如样本组成越相似，反映在PCA图中的距离越近。不同环境间的样本可能表现出分散和聚集的分布情况，PCA结果中对样本差异性解释度最高的两个成分可以用于对假设因素进行验证。

  

软件：R语言PCA统计分析和作图。

  

#### 结果说明:

结果目录： **4.Beta\_diversity\_analysis/PCA**

##### 图22 基于OTU水平的PCA分析

##### *注：不同颜色或形状的点代表不同的样本分组情况，横、纵坐标轴的刻度是相对距离，无实际意义。贡献率则是对其解释程度的大小，可以对假设因素进行评估验证。*

  

#### 参考文献

**[1]**Yu Wang, Hua-Fang Sheng, et al. Comparison of the Levels of Bacterial Diversity in Freshwater,Intertidal Wetland, and Marine Sediments by Using Millions of Illumina Tags. Appl. Environ. Microbiol. 2012, 78(23):8264. DOI: 10.1128/AEM.01821-12

## 5. PLS-DA分析

#### 分析方法:

PLS-DA分析  ^[1]^ 偏最小二乘法判别分析（Partial Least Squares Discrimination Analysis简称PLS-DA）是一种用于判别分析的多变量统计分析方法。判别分析是一种根据观察或测量到的若干变量值，来判断研究对象如何分类的常用统计分析方法。不同于主成分分析（ PCA）法，它是一种有监督的判别分析统计方法。该方法运用PLS-DA建立微生物含量与样品类别之间的关系模型，来实现对样品类别的预测。

  

软件：R语言作图。

  

#### 结果说明:

结果目录： **4.Beta\_diversity\_analysis/PLS-DA**

##### 图23 基于OTU的偏最小二乘法判别分析

## 6. NMDS分析

#### 分析方法:

非度量多维尺度法（NMDS）  ^[1]^  是一种将多维空间的研究对象（样本或变量）简化到低维空间进行定位、分析和归类，同时又保留对象间原始关系的数据分析方法。适用于无法获得研究对象间精确的相似性或相异性数据，仅能得到他们之间等级关系数据的情形。其基本特征是将对象间的相似性或相异性数据看成点间距离的单调函数，在保持原始数据次序关系的基础上，用新的相同次序的数据列替换原始数据进行度量型多维尺度分析。换句话说，当资料不适合直接进行变量型多维尺度分析时，对其进行变量变换，再采用变量型多维尺度分析，对原始资料而言，就称之为非度量型多维尺度分析。其特点是根据样本中包含的物种信息，以点的形式反映在多维空间上，而对不同样本间的差异程度，则是通过点与点间的距离体现的，最终获得样本的空间定位点图。

  

软件：R语言vegan与ggplot2等软件包进行数据计算NMDS分析和作图。

  

#### 结果说明:

结果目录： **4.Beta\_diversity\_analysis/NMDS**

图24 OTU水平NMDS分析

##### *注：一般说来同组样本之间均在一个圈内说明组间差异不明显，而组与组之间圈无交集则说明各组之间有一定差异性。*

#### 参考文献

**[1]**Magali Noval Rivas, PhD, Oliver T. Burton, et al. A microbita signature associated with experimental food allergy promotes allergic senitization and anaphylaxis. The Journal of Allergy and Clinical Immunology.Volume 131, Issue 1 , Pages 201-212, January 2013

# 多元化统计分析/Multivariate statistical analysis

- all
- metastats 组间显著性差异分析
- Kruskal-Wallis 检验
- Amova 分析
- Wilcoxon检验
- Anosim 分析
- LEfSe 分析

## 1.metastats组间显著性差异分析

#### 分析方法:

Metastats分析，即组间显著性差异分析，该分析通过对比两组条件下的多个样品，找出两组中具有显著差异的微生物类型。分析多个样品时，如果样品分属于两个组，则可以进行Metastats分析，若样品分为两组则直接进行对比分析，分为多组时，则需分别对每两组进行对比分析。

分析软件：mothur

#### 结果说明：

结果目录： **5.Statistical\_test\_analysis/Metastats**


## 2. Kruskal-Wallis检验

#### 分析方法

Kruskal-Wallis秩和检验是一个关于三组或更多数据的非参数性测试，可以计算多组独立数据均值之间的差异存在性问题。它只针对在图形上显示的数据的波动性进行处理，与 ANOVA 不同，所谓的非参数性测试不进行关于数据分布的假设。通过Kruskal-Wallis秩和检验我们可以从多个生物学重复的多组数据中得到OTU水平某些OTU是否具有差异，或分类学某些菌种是否具有差异性。这里我们认为p\_value小于0.05即视为有差异的OTU或物种。

分析软件：R及其qvalue包

#### 结果说明：

结果目录：**5.Statistical\_test\_analysis/Kruskal-wallis**

## 3.Amova方差分析

#### 分析方法:

分子方差分析^[1]^分子方差分析（Analysis of molecular variance）是一种传统方差分析的非参数模拟，与ANOVA类似。分子方差分析是基于加权或非加权Unifrac距离矩阵，检验不同组间差异显著性的非参数分析方法。一般基于Unifrac距离，使用mothur软件amova函数进行组间差异分析。

分析软件：mothur

#### 结果说明：

结果目录：**5.Statistical\_test\_analysis/Amova**

  

#### 参考文献

**[1]** Clarke, K. R. (1993). Non-parametric multivariate analysis of changes in community structure. \_Australian Journal of Ecology\_ 18, 117-143.


## 4. Wilcoxon检验

#### 分析方法:

Wilcoxon检验指的是秩和检验或签名秩检验，它是一个非参数检验，用于比较两个配对组。该测试基本上计算每组对之间的差异并分析这些差异。 Wilcoxon秩和检验可用于检验两个种群具有相同连续分布的无效假设。使用这种测试方法所必需的基本假设是数据来自同一群体并且配对，数据可以至少在一个区间范围内进行测量，并且数据是随机独立选择的。

分析软件：R及其stats包

#### 结果说明：

结果目录：**5.Statistical\_test\_analysis/Wilcoxon**

图25 Wilcoxon检验

#### 参考文献

**[1]** Clarke, K. R. (1993). Non-parametric multivariate analysis of changes in community structure. \_Australian Journal of Ecology\_ 18, 117-143.

## 5. Anosim分析

#### 分析方法:

Anosim^[1]^相似性分析是一种非参数检验，用来检验组间（两组或多组）的差异是否显著大于组内差异，从而判断分组是否有意义，这里计算两两组之间的相似性关系，并对所有分组进行计算作图。

  

软件：R，Vegan package:

  

##### 表6 两两组计算结果如下表

|  |  |  |  |  |
| --- | --- | --- | --- | --- |
| Method name | R statistic | p-value | Number of permutations | group |
| ANOSIM | 0.1049 | 0.030 | 999 | Normal-ES |
| ANOSIM | 0.4119 | 0.001 | 999 | Normal-ESC |
| ANOSIM | 0.4307 | 0.001 | 999 | ES-ESC |
| ANOSIM | 0.3570 | 0.001 | 999 | all |

  

#### 参考文献

**[1]**Clarke, K. R. (1993). Non-parametric multivariate analysis of changes in community structure. \_Australian Journal of Ecology\_ 18, 117-143.

##### 注：说明：R-value介于（-1，1）之间，R-value大于0，说明组间差异显著。R-value小于0，说明组内差异大于组间差异，统计分析的可信度用 P-value 表示，P< 0.05 表示统计具有显著性。


## 6. LEfSe分析

LDA Effect Size ^[1]^ 分析，可以实现多个分组之间的比较，还进行分组比较的内部进行亚组比较分析，从而找到组间在丰度上有显著差异的物种（即biomaker），其分析原理有如下三步：

1)首先在多组样本中采用的参数检验ANOVA检验检测不同分组间丰度差异显著的物种，阈值设定0.05；

2)然后在上一步中获得的显著差异物种，用成组的Wilcoxon秩和检验来进行组间差异分析，阈值设定0.05；

3)最后用线性判别分析（LDA）对数据进行降维和评估差异显著的物种的影响力（即LDA score），阈值设定3。

#### 结果说明:

结果目录： **5.Statistical\_test\_analysis/Lefse**

图26 基于分类信息的LEfSe分析的LDA分布柱状图

##### *注:展示了LDA score大于设定值有差异的物种，即具有统计学差异的biomaker。展现不同组中丰度有显著差异的物种，柱状图的长度代表显著差异物种的影响大小；*

图27 基于分类信息的LEfSe分析的进化分支图

##### *注:由内至外辐射的圆圈代表了由门至属（或种）的分类级别。在不同分类级别上的每一个小圆圈代表该水平下的一个分类，小圆圈直径大小与相对丰度大小呈正比。 着色原则：无显著差异的物种统一着色为黄色，差异物种 Biomarker跟随组进行着色，红色节点表示在红色组别中起到重要作用的微生物类群，绿色节点表示在绿色组别中起到重要作用的微生物类群，其它圈颜色意义类同。图中英文字母表示的物种名称在右侧图例中进行展示。*

#### 参考文献

**[1]**Segata N, Izard J, Waldron L, et al. Metagenomic biomarker discovery and explanation[J]. Genome Biol, 2011, 12(6): R60.

## 1.metastats组间显著性差异分析

#### 分析方法:

Metastats分析，即组间显著性差异分析，该分析通过对比两组条件下的多个样品，找出两组中具有显著差异的微生物类型。分析多个样品时，如果样品分属于两个组，则可以进行Metastats分析，若样品分为两组则直接进行对比分析，分为多组时，则需分别对每两组进行对比分析。

分析软件：mothur

#### 结果说明：

结果目录： **5.Statistical\_test\_analysis/Metastats**

## 2. Kruskal-Wallis检验

#### 分析方法

Kruskal-Wallis秩和检验是一个关于三组或更多数据的非参数性测试，可以计算多组独立数据均值之间的差异存在性问题。它只针对在图形上显示的数据的波动性进行处理，与 ANOVA 不同，所谓的非参数性测试不进行关于数据分布的假设。通过Kruskal-Wallis秩和检验我们可以从多个生物学重复的多组数据中得到OTU水平某些OTU是否具有差异，或分类学某些菌种是否具有差异性。这里我们认为p\_value小于0.05即视为有差异的OTU或物种。

分析软件：R及其qvalue包

#### 结果说明：

结果目录：**5.Statistical\_test\_analysis/Kruskal-wallis**

## 3.Amova方差分析

#### 分析方法:

分子方差分析^[1]^分子方差分析（Analysis of molecular variance）是一种传统方差分析的非参数模拟，与ANOVA类似。分子方差分析是基于加权或非加权Unifrac距离矩阵，检验不同组间差异显著性的非参数分析方法。一般基于Unifrac距离，使用mothur软件amova函数进行组间差异分析。

分析软件：mothur

#### 结果说明：

结果目录：**5.Statistical\_test\_analysis/Amova**

  

#### 参考文献

**[1]** Clarke, K. R. (1993). Non-parametric multivariate analysis of changes in community structure. \_Australian Journal of Ecology\_ 18, 117-143.

## 4. Wilcoxon检验

#### 分析方法:

Wilcoxon检验指的是秩和检验或签名秩检验，它是一个非参数检验，用于比较两个配对组。该测试基本上计算每组对之间的差异并分析这些差异。 Wilcoxon秩和检验可用于检验两个种群具有相同连续分布的无效假设。使用这种测试方法所必需的基本假设是数据来自同一群体并且配对，数据可以至少在一个区间范围内进行测量，并且数据是随机独立选择的。

分析软件：R及其stats包

#### 结果说明：

结果目录：**5.Statistical\_test\_analysis/Wilcoxon**

#### 参考文献

**[1]** Clarke, K. R. (1993). Non-parametric multivariate analysis of changes in community structure. \_Australian Journal of Ecology\_ 18, 117-143.

## 5. Anosim分析

#### 分析方法:

Anosim^[1]^相似性分析是一种非参数检验，用来检验组间（两组或多组）的差异是否显著大于组内差异，从而判断分组是否有意义，这里计算两两组之间的相似性关系，并对所有分组进行计算作图。

  

软件：R，Vegan package:

  

##### 表6 两两组计算结果如下表

|  |  |  |  |  |
| --- | --- | --- | --- | --- |
| Method name | R statistic | p-value | Number of permutations | group |
| ANOSIM | 0.1049 | 0.030 | 999 | Normal-ES |
| ANOSIM | 0.4119 | 0.001 | 999 | Normal-ESC |
| ANOSIM | 0.4307 | 0.001 | 999 | ES-ESC |
| ANOSIM | 0.3570 | 0.001 | 999 | all |

  

#### 参考文献

**[1]**Clarke, K. R. (1993). Non-parametric multivariate analysis of changes in community structure. \_Australian Journal of Ecology\_ 18, 117-143.

##### 注：说明：R-value介于（-1，1）之间，R-value大于0，说明组间差异显著。R-value小于0，说明组内差异大于组间差异，统计分析的可信度用 P-value 表示，P< 0.05 表示统计具有显著性。

## 6. LEfSe分析

LDA Effect Size ^[1]^ 分析，可以实现多个分组之间的比较，还进行分组比较的内部进行亚组比较分析，从而找到组间在丰度上有显著差异的物种（即biomaker），其分析原理有如下三步：

1)首先在多组样本中采用的参数检验ANOVA检验检测不同分组间丰度差异显著的物种，阈值设定0.05；

2)然后在上一步中获得的显著差异物种，用成组的Wilcoxon秩和检验来进行组间差异分析，阈值设定0.05；

3)最后用线性判别分析（LDA）对数据进行降维和评估差异显著的物种的影响力（即LDA score），阈值设定3。

#### 结果说明:

结果目录： **5.Statistical\_test\_analysis/Lefse**

图26 基于分类信息的LEfSe分析的LDA分布柱状图

##### *注:展示了LDA score大于设定值有差异的物种，即具有统计学差异的biomaker。展现不同组中丰度有显著差异的物种，柱状图的长度代表显著差异物种的影响大小；*

图27 基于分类信息的LEfSe分析的进化分支图

##### *注:由内至外辐射的圆圈代表了由门至属（或种）的分类级别。在不同分类级别上的每一个小圆圈代表该水平下的一个分类，小圆圈直径大小与相对丰度大小呈正比。 着色原则：无显著差异的物种统一着色为黄色，差异物种 Biomarker跟随组进行着色，红色节点表示在红色组别中起到重要作用的微生物类群，绿色节点表示在绿色组别中起到重要作用的微生物类群，其它圈颜色意义类同。图中英文字母表示的物种名称在右侧图例中进行展示。*

#### 参考文献

**[1]**Segata N, Izard J, Waldron L, et al. Metagenomic biomarker discovery and explanation[J]. Genome Biol, 2011, 12(6): R60.

# 相关性分析/References

- all
- 网络互作图

## 1.NetWork分析

#### 分析方法:

通过spearman检验方法，选取所有样本绝对丰度前20的属结果进行相互关联性分析，并以对应的门作为legend，计算所得结果过滤掉p值大于0.05的或相关值|R|<0.4的进行绘图。

  

软件及算法:R及其igraph包，psych包。

#### 结果说明:

结果目录： **6.Correlation\_analysis/Network**

  

##### 图28 网络互作图

##### *注：点的大小代表丰度的大小，线的粗细代表相关性大小；点的颜色代表所属门，线为红色表示呈正相关，蓝色表示呈负相关。*

## 1.NetWork分析

#### 分析方法:

通过spearman检验方法，选取所有样本绝对丰度前20的属结果进行相互关联性分析，并以对应的门作为legend，计算所得结果过滤掉p值大于0.05的或相关值|R|<0.4的进行绘图。

  

软件及算法:R及其igraph包，psych包。

#### 结果说明:

结果目录： **6.Correlation\_analysis/Network**

  

##### 图28 网络互作图

##### *注：点的大小代表丰度的大小，线的粗细代表相关性大小；点的颜色代表所属门，线为红色表示呈正相关，蓝色表示呈负相关。*

# 附录/References

- all
- 附录-目录结果
- 附录-软件列表

## 1. 附录-目录结果

目录结构说明： **Readme.txt**

## 2. 附录-软件列表

|  |  |
| --- | --- |
| 工作类型 | 软件或算法 |
| 分析平台 | Qiime |
| 原始下机数据质控 | Trimmomatic、Pear |
| 序列拼接 | Flash、Pear |
| Tag序列质控 | mothur、uchime |
| OTU聚类 | uclust、uparse |
| 比对 | RDP classifier、blast、uclust |
| 其他数据计算及其作图 | Qiime自带脚本、内部开发程序、R等 |

## 1. 附录-目录结果

目录结构说明： **Readme.txt**

## 2. 附录-软件列表

|  |  |
| --- | --- |
| 工作类型 | 软件或算法 |
| 分析平台 | Qiime |
| 原始下机数据质控 | Trimmomatic、Pear |
| 序列拼接 | Flash、Pear |
| Tag序列质控 | mothur、uchime |
| OTU聚类 | uclust、uparse |
| 比对 | RDP classifier、blast、uclust |
| 其他数据计算及其作图 | Qiime自带脚本、内部开发程序、R等 |
